# Electrically conductive charge-segregated pseudo-polymorphs comprising highly planar expanded π-electronic cations†

**DOI:** 10.1039/d4sc07576e

**Published:** 2025-02-19

**Authors:** Yohei Haketa, Ryoya Nakajima, Yuto Maruyama, Hiroki Tanaka, Wookjin Choi, Shu Seki, Shunsuke Sato, Hitomi Baba, Yoshiki Ishii, Go Watanabe, Kirill Bulgarevich, Kazuo Takimiya, Kenzo Deguchi, Shinobu Ohki, Kenjiro Hashi, Takashi Nakanishi, Yukihide Ishibashi, Tsuyoshi Asahi, Kazuchika Ohta, Hiromitsu Maeda

**Affiliations:** a Department of Applied Chemistry, College of Life Sciences, Ritsumeikan University Kusatsu 525-8577 Japan maedahir@ph.ritsumei.ac.jp; b Department of Molecular Engineering, Graduate School of Engineering, Kyoto University Kyoto 615-8510 Japan; c Department of Physics, Graduate School of Science, Kitasato University Sagamihara 252-0373 Japan; d Department of Data Science, School of Frontier Engineering, Kitasato University Sagamihara 252-0373 Japan; e Center for Emergent Matter Science (CEMS), RIKEN Wako 351-0198 Japan; f Department of Chemistry, Graduate School of Science, Tohoku University Sendai 980-8578 Japan; g Advanced Institute for Materials Research (AIMR), Tohoku University Sendai 980-8577 Japan; h Research Network and Facility Services Division, National Institute for Materials Science (NIMS) Tsukuba 305-0003 Japan; i Center for Basic Research on Materials, National Institute for Materials Science (NIMS) Tsukuba 305-0003 Japan; j Research Center for Materials Nanoarchitectonics (MANA), National Institute for Materials Science (NIMS) Tsukuba 305-0044 Japan; k Department of Applied Chemistry, Graduate School of Science and Engineering, Ehime University Matsuyama 790-8577 Japan; l Interdisciplinary Graduate School of Science and Technology, Shinshu University Ueda 386-8567 Japan

## Abstract

Independently stacked positively and negatively charged π-electronic systems in charge-segregated columnar structures are desired for electronic properties derived from their electron-deficient and -rich assembling states, respectively. An expanded π-electronic cation, benzoporphyrin Au^III^ complex, was synthesized as the component of ion pairs in combination with counteranions. In contrast to benzoporphyrin, which is known for its insolubility in organic solvents, the ion pairs with bulky anions in this study are soluble in common organic solvents. The ion pairs formed charge-segregated assemblies as two pseudo-polymorphs of single-crystal and less-crystalline (LeC) states based on the stacking of the benzoporphyrin Au^III^ complex. XRD and solid-state NMR measurements, along with molecular dynamics (MD) simulation, revealed that the LeC states were formed by a less-ordered arrangement of constituting ions induced by bulky counteranions. The electric conductivity properties were observed in the single-crystal and LeC charge-segregated assemblies.

## Abstract 2

Ion pairs of a benzoporphyrin Au^III^ complex as a planar expanded π-electronic cation exhibited anion-dependent charge-segregated assemblies in pseudo-polymorphs of single-crystal and less-crystalline states, exhibiting electric conductivity properties.

## Graphical abstract

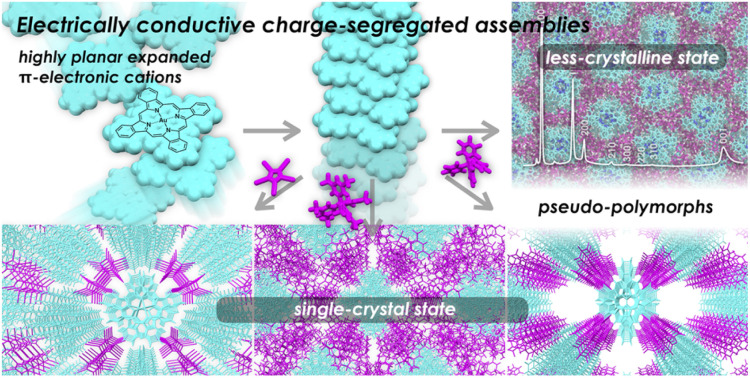

## Introduction

**Fig. 1:**
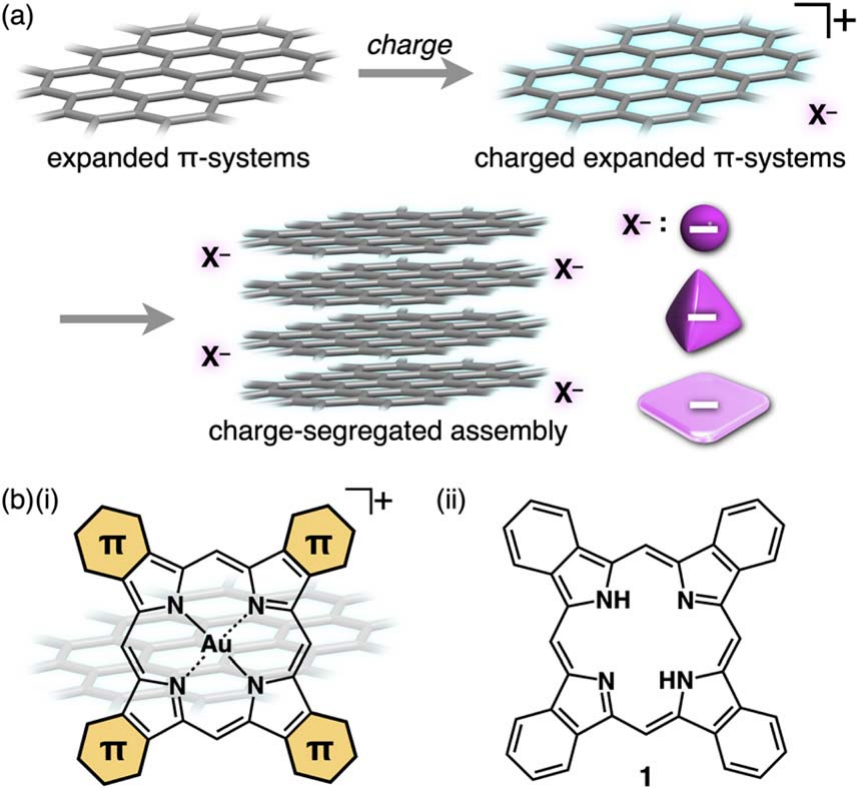
(a) Expanded π-electronic systems that have no peripheral substituents (top left) and their charged and ion-pairing states (top right, represented as cations), forming a charge-segregated assembly by stacking (bottom) and (b) (i) π-expanded porphyrin Au^III^ complexes as expanded π-electronic cations that can be used in (a) and (ii) benzoporphyrin 1. The positive signs were omitted in the charge-segregated assembly in (a).

The ordered arrangement of π-electronic systems is crucial for charge-carrier transport properties.^1^ Expanded π-planes are adequate for achieving high performance in organic semiconductive materials.^1*g*^ Since substituents affect the electronic states of molecules and their arrangement, π-electronic systems that have no substituents are in great demand.^1*e*^ However, such systems have low solubility (high crystallinity), making it difficult to arrange the constituents to form assembled structures (Fig. 1a top left). A promising strategy is the preparation of ion pairs of charged π-electronic systems by combining them with counterions that improve solubility (Fig. 1a top right).^2^ An appropriate combination of charged constituents enables facile handling of π-electronic systems to form counterion-dependent assemblies for applications (Fig. 1a bottom). In particular, independently stacked positively and negatively charged π-electronic systems are fascinating because of their ability to form electron-deficient and -rich assembling states, which can function as n- and p-type semiconductive pathways, respectively, in charge-segregated columnar structures.^3^ Expanded π-systems contribute to influential dispersion forces that overcome electrostatic repulsion in charged columns. The potential positively charged π-systems are porphyrin Au^III^ complexes, which have been included in various ion-pairing assemblies in the form of crystals, supramolecular gels and liquid crystals depending on the substituents.^4,5^ Expansion of π-electronic systems can be achieved by modifications at the pyrrole β-positions (Fig. 1b(i)). Numerous porphyrin derivatives that have been synthesized to date offered the choice to use benzoporphyrin^6^1 (Fig. 1b(ii)) as a framework to provide highly planar charged expanded π-electronic systems. Large planes of benzoporphyrin-based cations are suitable for stacking to form charge-segregated assemblies, whose packing structures can be controlled by coexisting counteranions. The arrangement of substituent-free planar cations and bulky anions can be modulated by crystallization conditions. This study shows π-expanded cation-based ion-pairing assemblies in single-crystal and pseudo-polymorph less-crystalline (LeC) states and their electric conductivity properties derived from the charge-segregated assemblies.

## Results and discussion

### Synthesis and characterization of expanded π-electronic cations

**Fig. 2:**
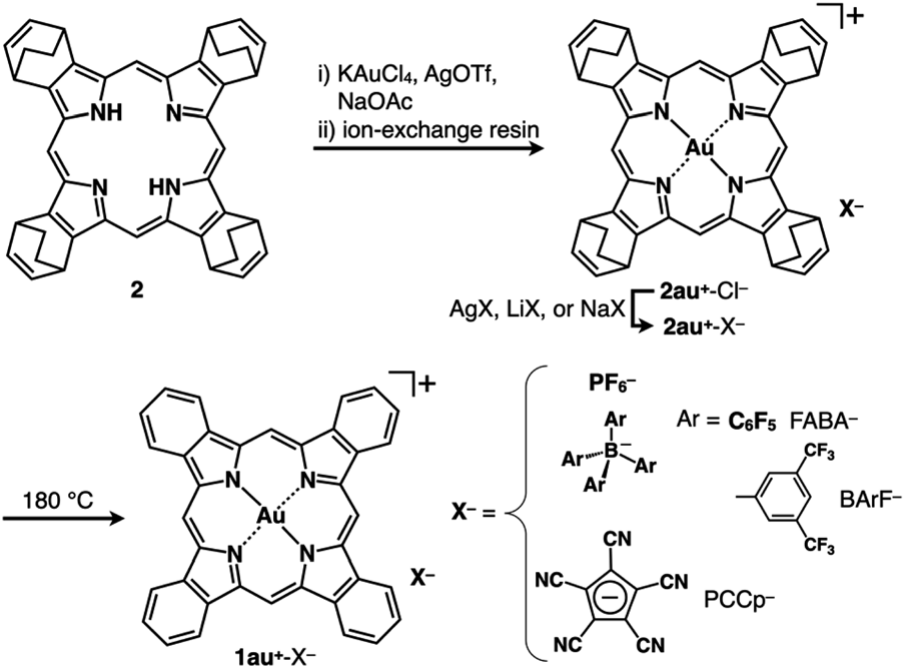
Synthesis of benzoporphyrin Au^III^ complex ion pairs 1au^+^-X^−^ (X^−^ = PF_6_^−^, FABA^−^, BArF^−^ and PCCp^−^) *via* the corresponding bicycloporphyrin Au^III^ complex ion pairs 2au^+^-X^−^.

**Fig. 3:**
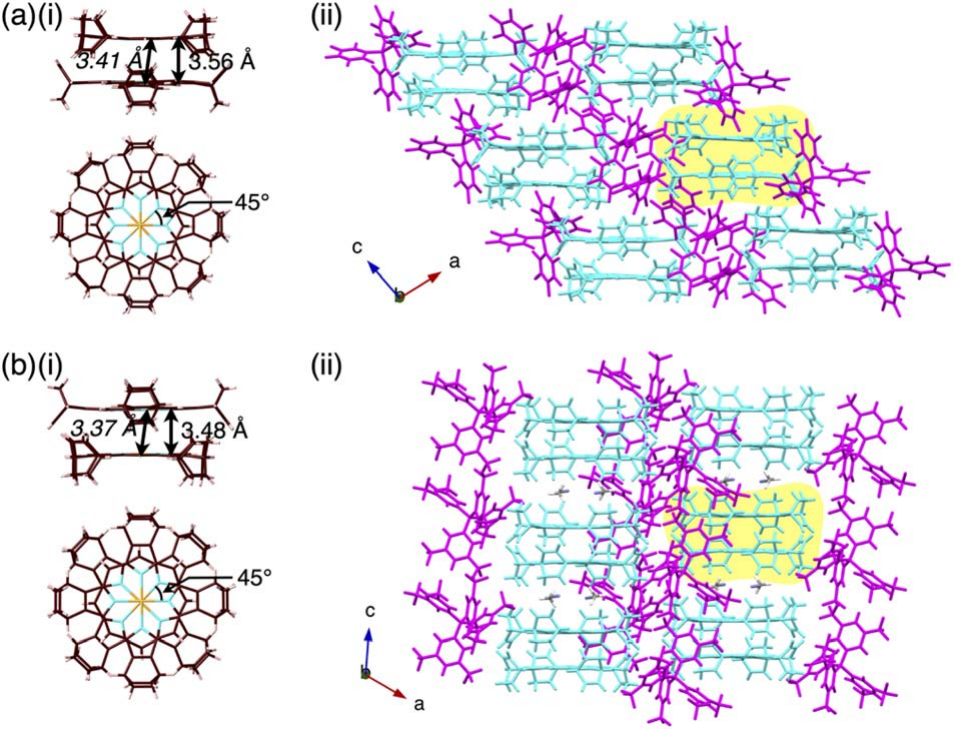
Single-crystal X-ray structures of (a) 2au^+^-FABA^−^ and (b) 2au^+^-BArF^−^: (i) side and top views of stacked dimers with stacking and Au⋯Au (italic) distances and (ii) packing structures with the yellow parts corresponding to the stacked dimers in (i). Atom colour codes in (i): brown, pink, blue and orange refer to carbon, hydrogen, nitrogen and gold, respectively. Colour codes in (ii): cyan and magenta refer to cation and anion parts, respectively. Solvent molecules are omitted in (a), whereas CH_3_CN molecules are shown in (b).

Benzoporphyrins, including metal complexes, can be synthesized from bicyclo[2.2.2]octadiene precursors *via* retro-Diels–Alder reactions.^6*f*^ In this study, Au^III^ complexation was conducted for bicycloporphyrin 2 to afford the Au^III^ complex 2au^+^ mainly as a triflate (OTf^−^) ion pair by treatment with KAuCl_4_ in the presence of AgOTf and NaOAc (Fig. 2 top). The ion pair 2au^+^-OTf^−^ was converted to the Cl^−^ ion pair 2au^+^-Cl^−^ in 28% yield (two steps) using an ion-exchange resin (Amberlite: IRA402BL Cl). As the selection of the counteranions was crucial in this study, the ion-pair metathesis of 2au^+^-Cl^−^ with AgPF_6_, LiB(C_6_F_5_)_4_ (LiFABA), NaB(3,5-(CF_3_)_2_C_6_H_3_)_4_ (NaBArF) and NaPCCp (PCCp^−^: pentacyanocyclopentadienide)^7^ afforded the corresponding ion pairs 2au^+^-X^−^ (X^−^ = PF_6_^−^, FABA^−^, BArF^−^ and PCCp^−^) in 66–76% yields. The obtained ion pairs were characterized by ^1^H, ^13^C and ^19^F NMR and ESI-TOF-MS. DMSO solutions of 2au^+^ ion pairs (4 μM) exhibited Soret and Q-bands at 392 and 507/542 nm, respectively, indicating that 2au^+^ exists as a monomeric state with a negligible counteranion effect on the electronic properties of 2au^+^ (Fig. S12†).^8^2au^+^-FABA^−^ and 2au^+^-BArF^−^ were also characterized by X-ray analysis for single crystals prepared by vapour diffusion from CH_3_CN/water (Fig. 3, S18 and S19†). In the solid-state structures, the cation 2au^+^, refined as one of the stereoisomers, formed stacked dimers with stacking/Au⋯Au distances of 3.56/3.41 and 3.48/3.37 Å, respectively, and a rotation of ∼45° owing to dispersion forces at the core planes and β-bicyclo units (Fig. 3a and b(i)). The stacking of 2au^+^ is visualized by Hirshfeld surface analysis, which shows a bow-tie arrangement of red and blue triangles in the shape-index property and flat regions in the curvedness property (Fig. S24 and S25†).^9^ Mean-plane deviations of 0.052/0.068 and 0.036 Å for stacking 2au^+^ planes (core 25 atoms), respectively, indicate slightly curved 2au^+^ planes, owing to the Au⋯Au distances being less than the stacking distances. Such remarkably close Au⋯Au distances are also ascribable to the orbital interactions arising from overlap of the 5d_*z*^2^_ and 6p_*z*_ orbitals of the adjacent Au^III^.^10^ The stacked 2au^+^ dimers in ion pairs are aligned along the *c*-axis with an offset, which is smaller in 2au^+^-BArF^−^ with a longer distance between the stacked dimers by including two CH_3_CN molecules (Fig. 3a and b(ii)). In either case, counteranions are located at the side of the stacked 2au^+^ dimers. In particular, BArF^−^ anions are located proximally at the side of the stacked 2au^+^ dimers, forming a pseudohexagonally arranged packing structure along the *c*-axis (Fig. S19a†).

Stacking of 2au^+^ by overcoming electrostatic repulsion was also observed in the solution state. In CD_3_CN, the ^1^H NMR of 2au^+^-FABA^−^ showed broad signals at 9.96, 8.37/6.94, 5.98 and 3.49–1.30 ppm ascribable to *meso*-CH, bicyclo-sp^2^-CH, bicyclo-bridged methine-CH and bicyclo-sp^3^-CH, respectively, for the stacked dimer.^11^ Concentration-dependent UV/vis absorption spectra of 2au^+^-FABA^−^ in CH_3_CN exhibited a blue shift of *λ*_max_ from 389 to 374 nm when increasing the concentration from 1.0 × 10^−6^ to 1.0 × 10^−5^ M, suggesting the formation of an H-like stacked dimer at the higher concentration (Fig. S71†). The transition dipole moments of 2au^+^ in the optimized structure of the stacked dimer 2au^+^_2_ based on PCM-GD3BJ-B3LYP/6-31+G(d,p) with LanL2DZ for Au (CH_3_CN) are arranged at ∼45°, suggesting that the H-like stacking mode induces a blue shift (Fig. S61a†).^12^ In addition, TD-DFT calculation of the optimized 2au^+^_2_ revealed an absorption at 375 nm, which is blue-shifted by 16 nm compared to the monomer state (Fig. S46†).

**Fig. 4:**
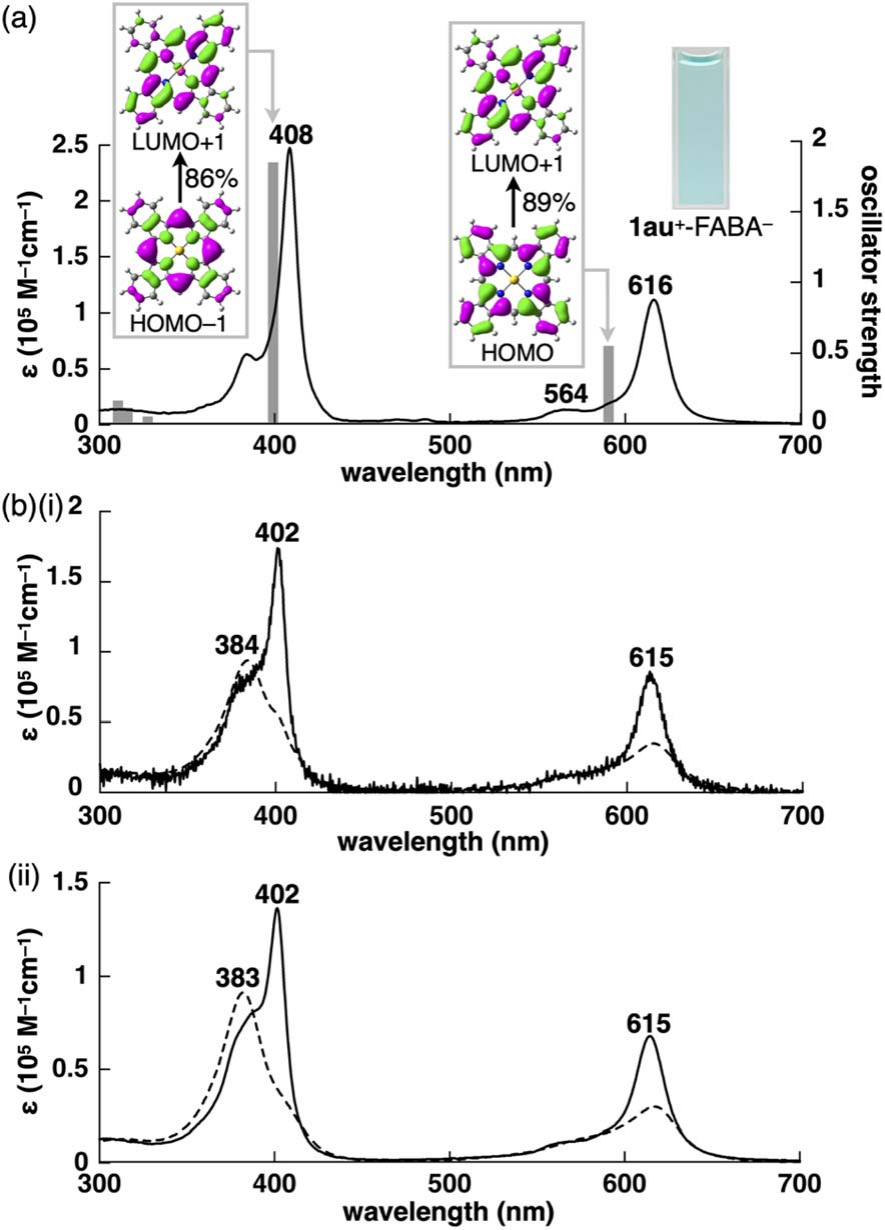
(a) UV/vis absorption spectrum of 1au^+^-FABA^−^ in DMSO (4 μM) (inset: photograph of the DMSO solution (4 μM)) and TD-DFT-based UV/vis absorption stick spectrum (grey bar) of 1au^+^ at PCM-B3LYP-GD3BJ/6-31+G(d,p) with LanL2DZ for Au (DMSO) and (b) UV/vis absorption spectra of 1au^+^-FABA^−^ in CH_3_CN according to (i) concentrations (solid line: 0.18 μM and broken line: 20 μM) and (ii) temperatures (solid line: 70 °C and broken line: −40 °C).

According to the synthetic procedure for benzoporphyrin 1,^6*f*^2au^+^-X^−^ (X^−^ = PF_6_^−^, FABA^−^, BArF^−^ and PCCp^−^) were quantitatively transformed to the corresponding benzoporphyrin ion pairs 1au^+^-X^−^ by heating at 180 °C for 20–60 min in the absence of solvent (Fig. 2 bottom). In contrast to 1,^6*f*^ which is insoluble in most organic solvents, the obtained 1au^+^-X^−^ showed enhanced solubility. For example, 1au^+^-FABA^−^ was soluble in acetone, DMF, CH_3_CN and DMSO. In contrast, another ion pair 1au^+^-Cl^−^, which was synthesized from 2au^+^-Cl^−^, was not soluble in these solvents.^13^ It is noteworthy that 1au^+^ is soluble with facile handling in the form of the ion pairs with appropriate counteranions, although the optimized structure of 1au^+^ estimated at B3LYP/6-31+G(d,p) with LanL2DZ for Au^12^ exhibits completely planar geometry with a mean-plane deviation of 0.00 Å (Fig. S29†). ^1^H NMR of 1au^+^-FABA^−^ in DMSO-*d*_6_ (1.0 mM), as an example, at r.t. exhibited broad signals at 10.21, 9.18 and 8.11 ppm, suggesting soluble but aggregated structures as also indicated by concentration-dependent ^1^H NMR (Fig. S74†).^14^ Such ^1^H NMR signals in the downfield region suggested the aromatic ring current effect of 1au^+^, which was further supported by nucleus-independent chemical shift (NICS) and the anisotropy of the current induced density (ACID) calculations (Fig. S54 and S55†). Interestingly, ^19^F NMR in the same solvent shows sharp signals derived from FABA^−^ in the dispersed state. The UV/vis absorption spectrum of 1au^+^-FABA^−^ in DMSO (4 μM), as a monomer state, exhibits Soret and Q bands of 408 and 564/616 nm, respectively (Fig. 4a), which are more red-shifted than those of 2au^+^-FABA^−^. The TD-DFT-based UV/vis absorption stick spectrum of 1au^+^ in DMSO shows that these absorptions are mainly derived from the HOMO−1-to-LUMO+1 and HOMO-to-LUMO+1 transitions, respectively (Fig. 4a inset, S49†).

The ^1^H NMR of 1au^+^-FABA^−^ in CD_3_CN (1.0 mM) shows broader signals than those in DMSO-*d*_6_, suggesting more aggregated structures in the less polar solvent (Fig. S9†). Similar to 2au^+^-FABA^−^, 1au^+^-FABA^−^ shows a *λ*_max_ blue-shift from 402 to 384 nm upon increasing the concentration from 1.8 × 10^−7^ to 2.0 × 10^−5^ M in CH_3_CN, suggesting the formation of H-like stacked structures (Fig. 4b(i) and S72†). Such a blue shift of the *λ*_max_ is also observed in variable-temperature (VT)-UV/vis absorption spectra at lower temperatures (Fig. 4b(ii)). TD-DFT calculation of the optimized structure for the stacked dimer 1au^+^_2_ at PCM-B3LYP-GD3BJ/6-31+G(d,p) with LanL2DZ for Au (CH_3_CN) suggests a slightly blue-shifted Soret band compared to that of the monomeric state (Fig. S51†). The dimerization constant (*K*_dim_) of 1au^+^ for 1au^+^-FABA^−^ is estimated to be 5 × 10^6^ M^−1^ in CH_3_CN at r.t. from concentration-dependent UV/vis absorption spectral changes (Fig. S72†). π-Expansion of positively charged π-electronic systems induces influential dispersion forces, which enable the stacking of identically charged π-electronic systems.

### Single-crystal-state charge-segregated assemblies

**Fig. 5:**
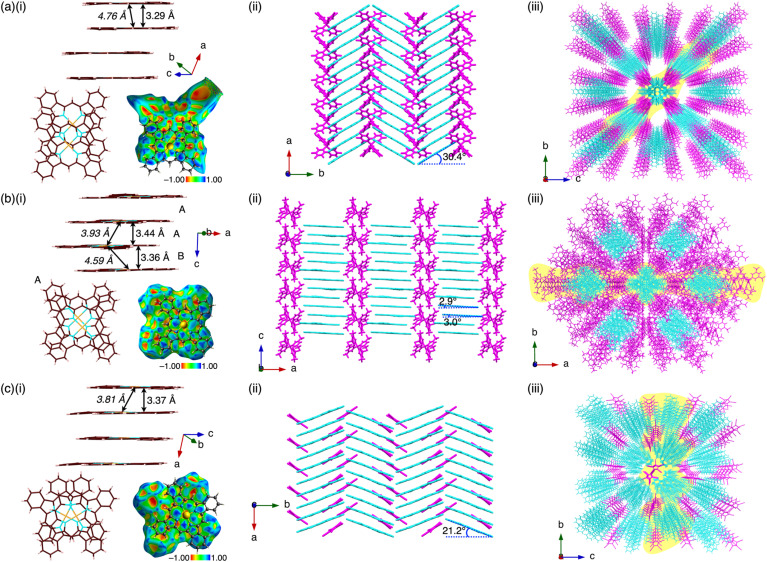
Single-crystal X-ray structures of (a) 1au^+^-FABA^−^, (b) 1au^+^-BArF^−^ and (c) 1au^+^-PCCp^−^: (i) side views of the columnar structures of stacked 1au^+^ with stacking and Au⋯Au (italic) distances and top views along with Hirshfeld surface mapped over the shape-index property of the stacked dimer, (ii) packing structures of columns of stacked 1au^+^ and counteranions and (iii) packing structures with the yellow parts corresponding to the packing structures in (ii). In (b), the different types of stacking arrangements are indicated by A and B (stacking arrangement A is shown as a representative). Atom colour codes in (i): brown, pink, blue and orange refer to carbon, hydrogen, nitrogen and gold, respectively. Colour codes in (ii and iii): cyan and magenta refer to cation and anion parts, respectively.

Prism-shaped single crystals of the ion pairs 1au^+^-X^−^ (X^−^ = FABA^−^, BArF^−^ and PCCp^−^), obtained from CH_3_CN/CHCl_3_,^15^ 1,1,1-trichloroethane/*n*-heptane and DMF/*o*-dichlorobenzene, respectively, were suitable for X-ray analysis, revealing the exact structures of the ion pairs and their assembled structures (Fig. 5). In these structures, the cation 1au^+^, showing planar geometry with mean-plane deviations (core 25 atoms) of 0.016, 0.031/0.011 and 0.023 Å, respectively, forms closely stacked columnar structures with stacking distances of 3.29, 3.36/3.44 and 3.37 Å, respectively, in the charge-segregated mode (Fig. 5a–c(i)). The stacked parts of 1au^+^ are clearly shown by Hirshfeld surface analysis, exhibiting a bow-tie arrangement of red and blue triangles in the shape-index property and flat regions in the curvedness property (Fig. S26–S28†).^9^ The Au⋯Au distances are 4.76, 3.93/4.59 and 3.81 Å, respectively, suggesting that the offset stacking of the cations is larger for 1au^+^-FABA^−^. The angles of 44.1°, 48.4°/60.1° and 62.1° are estimated, respectively, for the lines passing through two Au atoms of stacked 1au^+^ to the corresponding 41-atom mean planes. In these ion pairs, counteranions FABA^−^, BArF^−^ and PCCp^−^ are located at the side of the columnar structures (Fig. 5a–c(ii) and (iii)). The ion-pair crystals 1au^+^-FABA^−^, 1au^+^-BArF^−^ and 1au^+^-PCCp^−^ formed orthorhombic, monoclinic and orthorhombic packing, respectively, with the columns of stacked 1au^+^ aligned along the *a*-, *c*- and *a*-axes, respectively, which are the long axes of the prism crystals (Fig. S23†). Notably, the stacked 1au^+^ in the columns are tilted, with angles of 30.4°, 2.9°/3.0° and 21.2°, respectively, relative to the stacking axis (Fig. 5a–c(ii)). Interestingly, in the crystal packing of 1au^+^-FABA^−^, an ion-pair framework composed of columnar cation structures and counteranions forms two tubular spaces per unit cell, with a volume of 5.56 nm^3^, containing disordered solvent molecules. The solvent molecules in the single crystal are not removed after heating at 100 °C under vacuum, as revealed by the X-ray analysis.

**Fig. 6:**
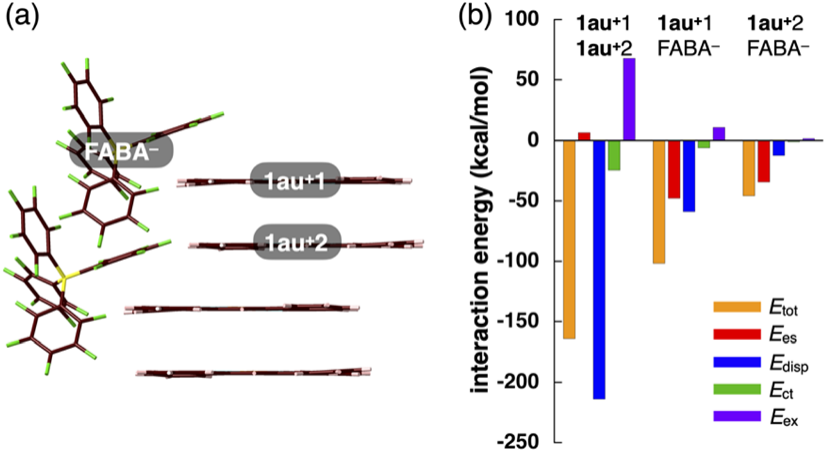
Decomposition of the total intermolecular interaction energies (*E*_tot_) of 1au^+^-FABA^−^ for (a) the single-crystal X-ray structure and (b) estimated interaction energies (kcal mol^−1^) according to EDA based on the FMO2-MP2 method using a basis set of NOSeC-V-DZP with MCP with NOSeC-V-TZP with MCP for Au (see Table S4† for the complete data list). Colour codes in (i): brown, pink, blue, yellow, green and orange refer to carbon, hydrogen, nitrogen, boron, fluorine and gold, respectively.

To evaluate the stacking behaviour of 1au^+^ in the crystal structures, energy decomposition analysis (EDA)^16,17^ was performed using the fragment molecular orbital (FMO) method (FMO2-MP2) with mixed basis sets including NOSeC-V-DZP with MCP with NOSeC-V-TZP with MCP for Au.^18,19^ The EDA calculations using FMO yielded *E*_es_, *E*_disp_, *E*_ct_ and *E*_ex_ (energies for electrostatic, dispersion, charge-transfer forces and exchange repulsion, respectively) and *E*_tot_ (total energy). In the columnar structure of 1au^+^ in 1au^+^-FABA^−^, an *E*_tot_ of −164.2 kcal mol^−1^ is observed, whereas *E*_disp_ and *E*_es_ are −214.0 and 6.5 kcal mol^−1^, respectively, indicating that *E*_disp_ is a major force in the stacking structure (Fig. 6 and S58†). EDA calculations for 1au^+^-BArF^−^ and 1au^+^-PCCp^−^ also elucidated similar energy balances for neighbouring π-electronic ions (Fig. S59 and S60†).

**Fig. 7:**
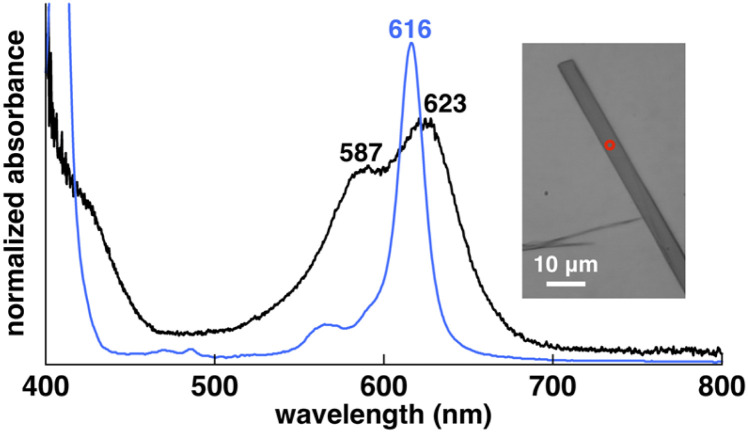
Solid-state UV/vis absorption spectra of 1au^+^-FABA^−^ in the single crystal (black line) and in DMSO (4 μM) (blue line) as a reference (inset: photograph of the single crystal (red circle indicates the position of measurement)).

Crystal-state absorptions of the ion pairs 1au^+^-FABA^−^, 1au^+^-BArF^−^ and 1au^+^-PCCp^−^ were evaluated *via* optical microscopy for spectroscopic examination (Fig. S75 and S76†). In 1au^+^-FABA^−^, the absorptions at 587 and 623 nm, which are slightly blue- and red-shifted, respectively, compared to those of the monomer in DMSO (616 nm), are ascribable to the exciton coupling in a predominantly J-like arrangement and also very weak coupling for orthogonally arranged transition dipole moments (Fig. 7 and S62†). These behaviours are derived from the *D*_4h_ geometry of 1au^+^. Similar to 1au^+^-FABA^−^, the crystal-state absorption of 1au^+^-PCCp^−^ shows absorptions at 588 and 623 nm, which are ascribable to the exciton coupling of stacked 1au^+^. On the other hand, 1au^+^-BArF^−^ mainly exhibits a blue-shifted broad absorption at 585 nm with a shoulder at 654 nm. The blue-shifted absorption can be attributed to the larger contribution of the H-like arrangement of transition dipoles in the stacked 1au^+^ (Fig. S62†).

**Fig. 8:**
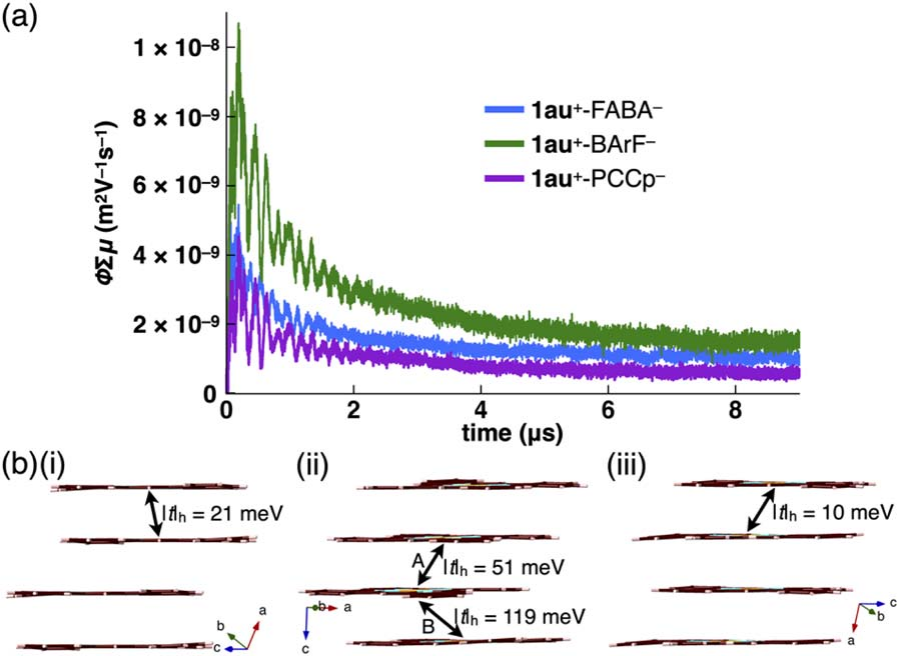
(a) Photoconductivity transients observed upon excitation at 355 nm, 9.1 × 10^15^ photons per cm^2^ per pulse, for the long axis of the single crystals of 1au^+^-FABA^−^ (blue), 1au^+^-BArF^−^ (green) and 1au^+^-PCCp^−^ (purple) and (b) stacked 1au^+^ in the single crystal structures of (i) 1au^+^-FABA^−^, (ii) 1au^+^-BArF^−^ and (iii) 1au^+^-PCCp^−^ and hole transfer integrals (|*t*|_h_) estimated at PW91/TZP. Stacking modes A and B in (ii) correspond to Fig. 5b(i).

Charge-segregated assemblies of π-electronic ion pairs show fascinating electric conductivity properties. The electric conductivity properties of stacked 1au^+^ in the ion pairs (1au^+^-FABA^−^, 1au^+^-BArF^−^ and 1au^+^-PCCp^−^) were evaluated by flash-photolysis time-resolved microwave conductivity (FP-TRMC) measurements (Fig. 8a and S91†).^20^ Electric conductivity (*ϕ*Σ*μ*) values of 4.6 × 10^−9^, 9.2 × 10^−9^ and 2.9 × 10^−9^ m^2^ V^−1^ s^−1^ were observed for the longer axes of the respective single crystals. Clear anisotropic electric conductivity was shown in 1au^+^-BArF^−^ (7.9 × 10^−9^ m^2^ V^−1^ s^−1^ for the shorter axis). These values are comparable to and greater than those of previously reported charge-segregated assemblies.^3^ The order of the *ϕ*Σ*μ* values, 1au^+^-BArF^−^ > 1au^+^-FABA^−^ > 1au^+^-PCCp^−^, is consistent with the theoretically estimated transfer integrals *t* at PW91/TZP for the stacked 1au^+^ units in the crystal structures, showing hole transfer integrals |*t*|_h_ of 118.5/50.5, 21.3 and 9.8 meV, respectively (Fig. 8b and S63†).^21^ Furthermore, theoretically estimated HOMO band dispersions using the tight-binding approximation^22^ for the stacked 1au^+^ in the single-crystal structures of 1au^+^-FABA^−^, 1au^+^-BArF^−^ and 1au^+^-PCCp^−^ exhibited one-dimensional band structures, which are consistent with the stacking structures of 1au^+^ (Fig. S64†). The Fermi levels lie in the middle of the band gaps, suggesting that charge-segregated assemblies comprising stacked 1au^+^ exhibit semiconductive behaviours. In the discussed ion pairs, decreased on-site Coulomb repulsion between stacked π-electronic cations with expanded π-electronic systems would induce hole transport rather than electron transport. The conductivity transients recorded under an SF_6_ atmosphere with negligible quenching of charge carriers suggest a major contribution from holes photo-injected into the assemblies (Fig. S92†).^23^

### Charge-segregated assemblies in pseudo-polymorph less-crystalline states

**Fig. 9:**
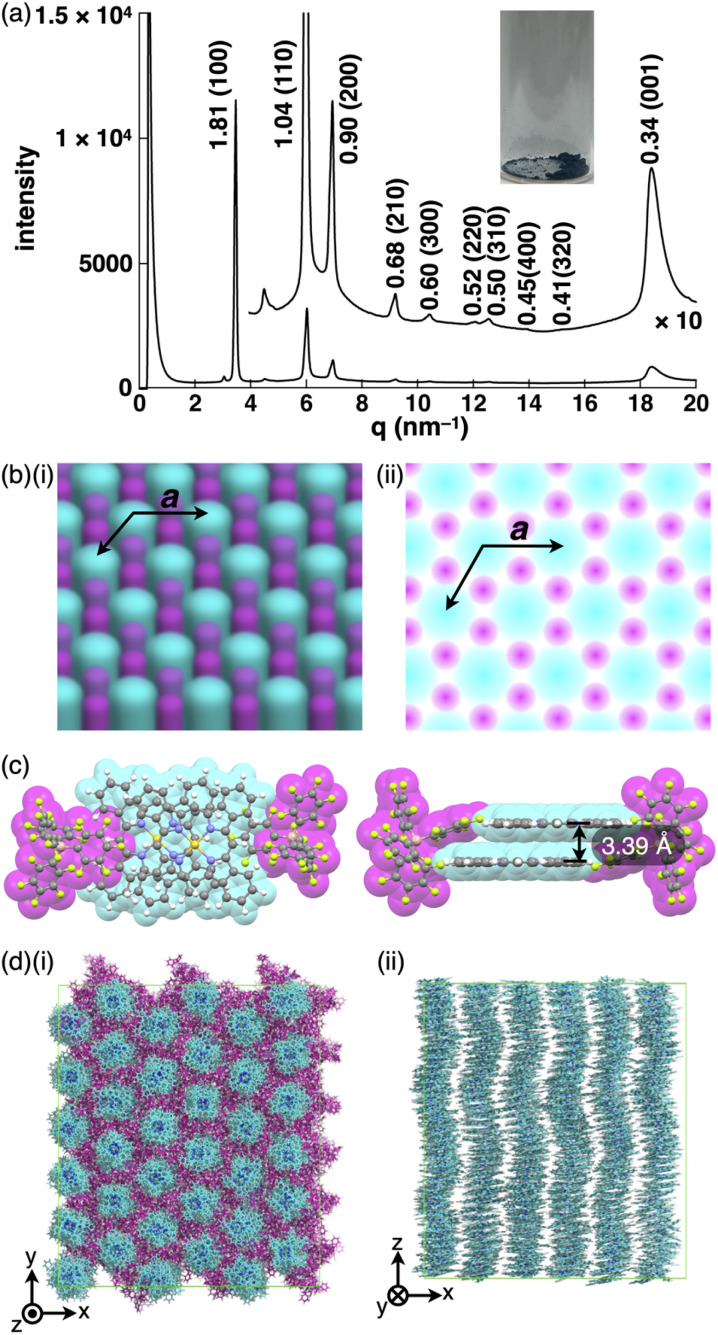
(a) Synchrotron XRD pattern of 1au^+^-FABA^−^_P_ at 25 °C for the sample obtained by precipitation from acetone/*n*-hexane (inset: photograph of the precipitates), (b) schematic representation for the Col_h_ packing structure: (i) packing diagram and (ii) top view of Col_h_ (1au^+^ and FABA^−^ are represented in cyan and magenta, respectively), (c) top and side views of the optimized structure of 1au^+^-FABA^−^ as a dimer using B3LYP-GD3BJ/6-31+G(d,p) with LanL2DZ for Au and (d) snapshot of the MD simulation result for 1au^+^-FABA^−^_P_ after 100 ns of the equilibration run at 25 °C showing (i) the top view of the packing diagram and (ii) the side view of 1au^+^ columns extracted from the packing diagram (1au^+^ and FABA^−^ are represented in cyan and magenta, respectively). In (b) (ii), magenta circles suggest the possible locations of FABA^−^ with a 50% occupancy rate on average in a cross section, whereas cyan circles show the diameter of 1au^+^ columns in slipped stacking.

Substituent-free benzoporphyrins in the forms of free base and metal complexes have been known to show highly crystalline states after heating the corresponding bicyclo[2.2.2]octadiene precursors in the film state.^6*h*^ In contrast, 1au^+^ as ion pairs can form bulk materials that are not single crystals *via* recrystallization from appropriate solvents. Precipitation of 1au^+^-FABA^−^ from acetone/*n*-hexane provided a material (labelled as 1au^+^-FABA^−^_P_) that appeared to differ from the single crystals (Fig. 9a inset).^24^ The synchrotron XRD of 1au^+^-FABA^−^_P_ at 25 °C exhibited broad peaks instead of crystalline diffraction, suggesting the formation of an LeC state for the obtained precipitates. The diffraction peaks of 1.81, 1.04, 0.90, 0.68, 0.60, 0.52, 0.50, 0.45, 0.41 and 0.34 nm, showing Debye–Scherrer rings, were assigned to the *hkl* parameters derived from a hexagonal pattern (100, 110, 200, 210, 300, 220, 310, 400 and 320) as *a* = 2.09 nm (Fig. 9a and S81†).^25^ The intense peak at 0.34 nm was assigned to 001 as the stacking distance of 1au^+^ in the hexagonal columnar (Col_h_) structure (*Z* = 1 for *ρ* = 1.79 g cm^−3^) (Fig. 9b). Interestingly, heating the powder sample of 2au^+^-FABA^−^ ^26^ at 190 °C (labelled as 1au^+^-FABA^−^_H_) also formed a Col_h_ structure identical to that of 1au^+^-FABA^−^_P_ (Fig. S79–S82†). In contrast to the tilted 1au^+^ plane along the *a*-axis in the single crystal of 1au^+^-FABA^−^, the 1au^+^ plane in 1au^+^-FABA^−^_P_ should be arranged perpendicularly to the stacking axis, as indicated by the intracolumnar stacking period of 0.34 nm. On the basis of the Col_h_ structure with *a* = 2.09 nm and the sizes of 1au^+^ and FABA^−^, columnar structures comprising less-ordered stacking of 1au^+^, as indicated by cyan circles, are located close to FABA^−^, as indicated by magenta circles (Fig. 9b(ii)).

The proximal location of 1au^+^ and FABA^−^ is also suggested by the optimized structure of dimeric 1au^+^-FABA^−^ using B3LYP-GD3BJ/6-31+G(d,p) with LanL2DZ for Au (Fig. 9c). FABA^−^ should be paired with several 1au^+^ in the proximal location, although the XRD pattern suggests that the location of FABA^−^ is less clear, probably indicating an amorphous-like state. In light of the stoichiometry of the constituents and their contrasting planar and bulky shapes, FABA^−^ would be observed in three sites on average among the 1au^+^ columns, and, in another cross section according to the 1au^+^ planes, the anions should be located in the other three sites. As a result, FABA^−^ can be hexagonally arranged around the 1au^+^ columns. The Col_h_ structure suggested by the XRD pattern is clearly demonstrated by all-atom molecular dynamics (MD) simulations at 25 °C after 100 ns of the equilibration run (Fig. 9d and S66†). Notably, as the initial structure for the MD simulation, the columns comprising tilted 1au^+^ units, as observed in the single-crystal structure, are transformed to a structure with barely tilted 1au^+^. The combination of planar 1au^+^, suitable for stacking, and bulky FABA^−^, with a less-ordered arrangement *via* noncovalent interactions induces the LeC state.^27,28^ The less-ordered FABA^−^ interferes with the ordered packing of 1au^+^ for crystallization. 1au^+^-FABA^−^_P_ maintains the Col_h_ structure up to 195 °C and is converted to a complicated crystalline state at higher temperatures, with decomposition at 355 °C. This behaviour is also supported by differential scanning calorimetry (DSC) analysis (Fig. S78†). The appearance of 1au^+^-FABA^−^_P_ as a dark blue powder in polarized optical microscopy (POM) was maintained through the heating process. The condition-dependent assembly (single-crystal and LeC states) as pseudo-polymorphism^29^ is fascinating for tuneable properties according to the arrangement of building blocks. The Col_h_ LeC state, in the absence of aliphatic chains, is rare^28^ but can be achieved by pairing the planar π-electronic cation with a bulky counteranion.^30^

**Fig. 10:**
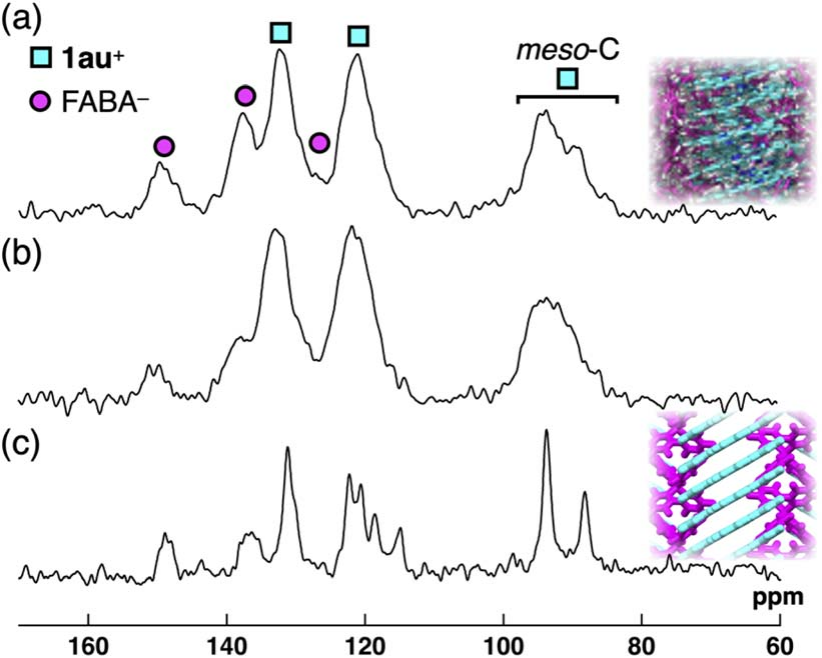
Solid-state ^13^C CPMAS NMR spectra of (a) 1au^+^-FABA^−^_P_, (b) 1au^+^-FABA^−^_H_ and (c) 1au^+^-FABA^−^ as single crystals recorded at 20 kHz MAS spinning frequency at r.t. with the corresponding packing diagrams.

The details of the structures of 1au^+^-FABA^−^ were investigated by solid-state NMR (SSNMR) spectroscopy. ^13^C NMR was performed using cross-polarization magic-angle spinning (CPMAS) (Fig. 10 and S86†). Dipolar dephasing experiments were conducted to support the signal assignment for the ion pair (Fig. S86 and S88†). The broad signals at 132.2, 120.8, 93.4 and 89.3 ppm were assigned to 1au^+^, whereas those at 149.7, 137.7 and 126.0 ppm were assigned to FABA^−^ (Fig. 10a). ^13^C CPMAS NMR of 1au^+^-FABA^−^_H_ showed slightly broader signals than 1au^+^-FABA^−^_P_, suggesting the formation of a more disordered arrangement of constituting ions (Fig. 10b). In contrast, ^13^C CPMAS NMR for 1au^+^-FABA^−^ as single crystals showed narrower signals than those of 1au^+^-FABA^−^_P_ and 1au^+^-FABA^−^_H_ (Fig. 10c), suggesting that the broader signals of 1au^+^-FABA^−^_P_ and 1au^+^-FABA^−^_H_ indicated a less-ordered arrangement of ions that form Col_h_ structures. Similar signal broadening was observed for ^19^F and ^11^B MAS NMR of 1au^+^-FABA^−^_P_ and 1au^+^-FABA^−^_H_ (Fig. S89 and S90†). Moreover, the characteristic up-field split signals at 93.4 and 89.3 ppm in 1au^+^-FABA^−^_P_ are derived from unsubstituted *meso*-carbons,^31^ as also suggested by theoretically estimated NMR signals for 1au^+^ using B3LYP/6-311+G(d,p) with SDD for Au (Fig. S65†).^12^ Such signal splitting is also observed in the ^13^C CPMAS NMR of the single crystals due to slipped stacking of 1au^+^ observed in the crystal structure of 1au^+^-FABA^−^ (Fig. 5a(i)).^32^ These observations, along with the XRD analysis and MD simulation, support a less-ordered slipped stacking structure for 1au^+^ in a column of Col_h_ LeC states, wherein the 1au^+^ planes are more perpendicularly arranged to the column compared to those in the single crystal.^33^

The formation of such an LeC state was also observed in the precipitate of 1au^+^-BArF^−^_P_ prepared from acetone/*n*-hexane.^34^ Similar to 1au^+^-FABA^−^_P_, 1au^+^-BArF^−^_P_ shows a Col_h_ LeC state (*a* = 2.28 nm, *c* = 0.34 nm, and *Z* = 1 for *ρ* = 1.71 g cm^−3^) (Fig. S84 and S85†), also exhibiting condition-dependent pseudo-polymorphs. Col_h_ LeC states in the precipitates were observed for 1au^+^-FABA^−^ and 1au^+^-BArF^−^, which formed orthorhombic and monoclinic single-crystal packing structures, respectively. Such pseudo-polymorphic phenomena are fascinating because stacking of 1au^+^ can be easily controlled by the assembly conditions. The electric conductivity (*ϕ*Σ*μ*) values of LeC-state 1au^+^-FABA^−^_P_, 1au^+^-FABA^−^_H_ and 1au^+^-BArF^−^_P_ were estimated to be 1.2 × 10^−9^, 1.0 × 10^−9^ and 3.4 × 10^−9^ m^2^ V^−1^ s^−1^, respectively, suggesting that electrically conductive pathways also exist in the columnar structures of LeC materials, although the values are smaller than those of the corresponding single crystals (Fig. S93†).

## Conclusions

Ion-pairing assemblies in charge-segregated modes were constructed from a highly planar expanded π-electronic cation in combination with counteranions. Charge-segregated assemblies were formed with both planar and bulky counteranions by means of stable stacked structures of the expanded π-electronic cation. The stacking arrangement and resulting absorption spectra in the single crystals were modulated by coexisting anions. Depending on crystallizing solvent conditions, ion pairs with bulky borate anions also provided LeC states as pseudo-polymorphs of their single crystals. Both single crystals and LeC states exhibited electric conductive properties due to stacking of the π-expanded porphyrin Au^III^ complex.^35^ It is noteworthy that the discussed planar π-electronic cation, benzoporphyrin Au^III^ complex, is soluble in organic solvents in the ion-pairing states. Ion pairing is an effective strategy to increase the solubility of planar π-electronic systems for their facile handling and the fabrication of assembled structures with ordered arrangements. Further modifications of charged π-electronic systems would lead to ion-pairing assemblies that can be applied for functional electronic materials and devices.

## Author contributions

H. M. designed and conducted the project. Y. H., R. N., Y. M. and H. T. carried out the synthesis, characterization and property examinations. W. C. and S. Se. evaluated the electric conductivities. S. Sa., H. B., Y. I. and G. W. conducted the MD calculations. K. B. and K. T. conducted the transfer integral calculations. K. D., S. O., K. H. and T. N. evaluated the SSNMR. Y. I. and T. A. recorded the solid-state absorption spectra. K. O. supported the discussion on the assemblies.

## Conflicts of interest

There are no conflicts of interest to declare.

## Supplementary Material

SC-016-D4SC07576E-s001

SC-016-D4SC07576E-s002

## Data Availability

Data supporting the work in this publication are available *via* the ESI and associated crystallographic data.
