# Supplementary material for: Electrically conductive charge-segregated pseudo-polymorphs comprising highly planar expanded π-electronic cations
Source: Chem Sci. 2025 Feb 19;16(12):4998–5006. doi: 10.1039/d4sc07576e (PMC11873929; doi:10.1039/d4sc07576e)
Supplement: SC-016-D4SC07576E-s001 [file SC-016-D4SC07576E-s001.pdf]

Supplementary Information (SI) for Chemical Science.
This journal is © The Royal Society of Chemistry 2025

Electronic Supplementary Information

Electrically conductive charge-segregated pseudo-polymorphs comprising highly planar expanded π-electronic cation

Yohei Haketa, Ryoya Nakajima, Yuto Maruyama, Hiroki Tanaka, Wookjin Choi, Shu Seki, Shunsuke Sato, Hitomi Baba, Yoshiki Ishii, Go Watanabe, Kirill Bulgarevich, Kazuo Takimiya, Kenzo Deguchi, Shinobu Ohki, Kenjiro Hashi, Takashi Nakanishi, Yukihide Ishibashi, Tsuyoshi Asahi, Kazuchika Ohta and Hiromitsu Maeda*

Department of Applied Chemistry, College of Life Sciences, Ritsumeikan University, Kusatsu 525–8577, Japan, Fax: +81 77 561 2659; Tel: +81 77 561 5969; E-mail: maedahir@ph.ritsumei.ac.jp, Department of Molecular Engineering, Graduate School of Engineering, Kyoto University, Kyoto 615–8510, Japan, Department of Physics, Graduate School of Science, Kitasato University, Sagamihara 252–0373, Japan, Department of Data Science, School of Frontier Engineering, Kitasato University, Sagamihara 252–0373, Japan, Center for Emergent Matter Science (CEMS), RIKEN, Wako 351–0198, Japan, Department of Chemistry, Graduate School of Science, Tohoku University, Sendai 980–8578, Japan, Advanced Institute for Materials Research (AIMR), Tohoku University, Sendai 980–8577, Japan, Research Network and Facility Services Division, National Institute for Materials Science (NIMS), Tsukuba 305–0003, Japan, Center for Basic Research on Materials, National Institute for Materials Science (NIMS), Tsukuba 305–0003, Japan, Research Center for Materials Nanoarchitectonics (MANA), National Institute for Materials Science (NIMS), Tsukuba 305–0044, Japan, Department of Applied Chemistry, Graduate School of Science and Engineering, Ehime University, Matsuyama 790–8577, Japan, and Interdisciplinary Graduate School of Science and Technology, Shinshu University, Ueda 386–8567, Japan

Table of contents

1. Synthetic procedures and spectroscopic data S3
Fig. S1 Synthesis of benzoporphyrin Au^{III} complex ion pairs. S3
Fig. S2–11 ^{1}H, ^{13}C, and ^{19}F NMR spectra. S6
Fig. S12 UV/vis absorption spectra. S20
2. X-ray crystallographic data S21
Fig. S13–17 Ortep drawings. S22
Fig. S18–23 Packing diagrams. S25
Fig. S24–30 Hirshfeld surface analysis. S30
3. Theoretical studies S36
Fig. S29–31 Optimized structures. S37
Fig. S32 ESP mapping. S38
Fig. S33–52 Molecular orbitals and TD-DFT calculations. S39
Fig. S53,54 Theoretical calculation for NICS. S52
Fig. S55 Theoretical calculation for ACID. S54
Fig. S56–60 Single-crystal X-ray structures for EDA. S54
Fig. S61,62 Theoretical calculations and evaluation for the transition dipole moments. S56
Fig. S63 Packing structures for transfer integrals. S58
Fig. S64 Theoretically estimated band dispersions. S59
Fig. S65 Simulated ^{13}C NMR. S60
Fig. S66 MD simulations for LeC. S61
Cartesian coordination of optimized structures S61
4. Solution-state assembled behaviors S73
Fig. S67–72 UV/vis absorption spectra. S73
Fig. S73,74 Variable-concentration ^{1}H NMR spectra. S76
5. Solid-state assembled behaviors S78
Fig. S75,76 Optical microscopic image and UV/vis absorption spectra of single crystals. S78
Fig. S77,78 DSC. S79
Fig. S79–85 XRD patterns and proposed packing models. S81
Fig. S86–90 Solid-state NMR. S88
6. Electric conductivity properties S91

Fig. S91–93 Electric conductivity. S92

1. Synthetic procedures and spectroscopic data

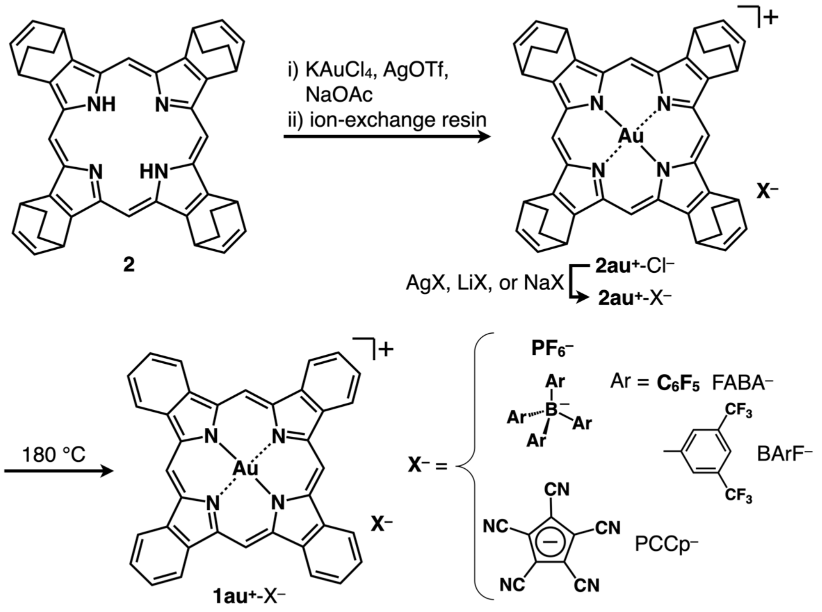

Fig. S1 Synthesis of benzoporphyrin Au^{III} complex ion pairs.

General Procedures. Starting materials were purchased from FUJIFILM Wako Pure Chemical Corp., Nacalai Tesque Inc., and Sigma-Aldrich Co. and were used without further purification unless otherwise stated. NMR spectra used in the characterization of products were recorded on a JEOL ECA-600 600 MHz spectrometers. All NMR spectra were referenced to solvent. UV-visible absorption spectra were recorded on a Hitachi U-3500 spectrometer. High-resolution (HR) electrospray ionization mass spectrometry (ESI-MS) was recorded on a BRUKER microTOF using ESI-TOF method. TLC analyses were carried out on aluminum sheets coated with silica gel 60 (Merck 5554). Column chromatography was performed on Wakogel C-300.

Bicycloporphyrin Au^{III} complex as a Cl$^-$ ion pair, 2au$^+$-Cl$^-$. Following the literature procedure,[S1] to a solution of bicyclooctadiene-fused porphyrin (bicycloporphyrin)[S2] **2** (100.6 mg, 0.163 mmol) in CH_2Cl_2 (23 mL) were added NaOAc (214 mg, 2.61 mmol), $KAuCl_4$ (244 mg, 0.646 mmol), and AgOTf (0.644 g, 2.51 mmol) in a mixed solution of THF (31.6 mL) and water (4.4 mL). After the mixture was stirred at 0 °C for 1 h, the solvent was removed. The residue, mainly containing a $CF_3SO_3^-$ ion pair **2au**$^+$-$CF_3SO_3^-$, was chromatographed over ion-exchanged resin (Amberlite IRA402BL Cl, eluent: MeOH) and was purified by silica gel column chromatography (Wakogel C-300; eluent: 5% acetone/CH_2Cl_2 to 5% MeOH/CH_2Cl_2 and subsequently 10% MeOH/CH_2Cl_2), followed by recrystallization from CH_2Cl_2/*n*-hexane to afford **2au**$^+$-Cl$^-$ (39.5 mg, 46.3 μmol, 28%) as a red solid. R_f = 0.23 (10% MeOH/CH_2Cl_2). ^{1}H NMR (600 MHz, CD_3CN, 20 °C): δ(ppm) 10.03/9.96 (br, 4H, *meso*-CH), 8.35/6.93 (br, 8H, CH=CH), 6.03–5.87 (m, 8H, CH), 3.8–1.3 (separated broad signals derived from partially associated isomers, 16H, CH_2CH_2). $^{13}C\{^1H\}$ NMR (151 MHz, CD_3CN, 20 °C): δ(ppm) 148.66, 138.11, 127.64, 100.11, 37.38, 28.20. UV/vis (CH_2Cl_2, λ_{max}[nm] (ε, 10^5 $M^{-1}cm^{-1}$)): 392 (1.9), 507 (0.13), 542 (0.13). ESI-TOF-MS: *m/z* 817.2600. Calcd for $C_{44}H_{36}AuN_4$ ([M − Cl]$^+$): 817.2600.

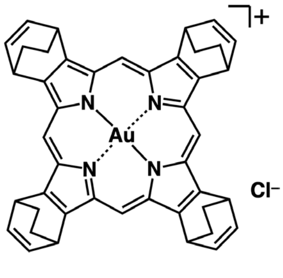

Bicycloporphyrin Au^{III} complex as a PF_6^- ion pair, 2au$^+$-PF_6^-. $AgPF_6$ (15.3 mg, 60.5 μmol) in MeOH (2.0 mL) was added to **2au**$^+$-Cl$^-$ (19.9 mg, 23.3 μmol) in MeOH (8.0 mL), and the reaction mixture was stirred for 15 min. The mixture was purified by silica gel column chromatography (Wakogel C-300; eluent: 3% MeOH/CH_2Cl_2) and was recrystallized from CH_2Cl_2/MeOH to afford **2au**$^+$-PF_6^- (15.9 mg, 16.5 μmol, 71%) as a red solid. R_f = 0.29 (3% MeOH/CH_2Cl_2). ^{1}H NMR (600 MHz, CD_3CN, 20 °C): δ(ppm) 9.97 (br, 4H, *meso*-CH), 8.35/6.94 (br, 8H, CH=CH), 6.04–5.87 (m, 8H, CH), 3.8–1.3 (separated broad signals derived from partially associated isomers, 16H, CH_2CH_2). $^{13}C\{^1H\}$ NMR (151 MHz, DMSO-d_6, 20 °C): δ (ppm) 147.28, 137.11, 126.08, 99.30, 35.82, 27.40. ^{19}F NMR (564 MHz, CD_3CN, 20 °C): δ(ppm) −73.08 (d, $J_{19F-31P}$ = 705.0 Hz, 6F). UV/vis (CH_2Cl_2, λ_{max}[nm] (ε, 10^5 $M^{-1}cm^{-1}$)): 392 (2.1), 507 (0.15), 542 (0.15). ESI-TOF-MS: (pos) *m/z* 817.2600. Calcd for $C_{44}H_{36}AuN_4$ ([M – F_6P]$^+$): 817.2600.

(neg) *m/z* 144.9647. Calcd for F_6P ([M – $C_{44}H_{36}AuN_4$]$^-$): 144.9647.

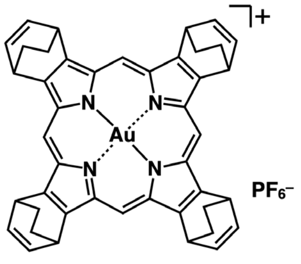

Bicycloporphyrin Au^{III} complex as a FABA$^-$ ($B(C_6F_5)_4^-$) ion pair, 2au$^+$-FABA$^-$. $LiB(C_6F_5)_4$ (LiFABA) (38.2 mg, 55.7 µmol) in CH_2Cl_2 (4.0 mL) was added to **2au**$^+$-Cl$^-$ (31.1 mg, 36.5 µmol) in CH_2Cl_2 (16.0 mL), and the reaction mixture was stirred for 10 min. The mixture was purified by silica gel column chromatography (Wakogel C-300; eluent: 25% *n*-hexane/CH_2Cl_2) and was recrystallized from CH_2Cl_2/*n*-hexane to afford **2au**$^+$-FABA$^-$ (40.6 mg, 27.1 µmol, 74%) as a red solid. R_f = 0.39 (25% *n*-hexane/CH_2Cl_2). ^{1}H NMR (600 MHz, CD_3CN, 20 °C): δ(ppm) 10.05/9.96 (br, 4H, *meso*-CH), 8.37/6.94 (br, 8H, CH=CH), 6.04–5.87 (m, 8H, CH), 3.8–1.3 (separated broad signals derived from partially associated isomers, 16H, CH_2CH_2). ^{13}C{^{1}H} NMR (151 MHz, CD_3CN, 20 °C): δ(ppm) 148.95 (d, $J_{13C-19F}$ = 239.9 Hz), 148.52, 139.18 (d, $J_{13C-19F}$ = 245.8 Hz), 137.61, 137.19 (d, $J_{13C-19F}$ = 238.6 Hz), 127.62, 124.90, 99.97, 37.31, 28.26. ^{19}F NMR (564 MHz, CD_3CN, 20 °C): δ(ppm) –133.85 (br, 8F), –163.95 (t, J = 19.7 Hz, 4F), –168.39 (br, 8F). UV/vis (CH_2Cl_2, λ_{max}[nm] (ε, 10^5 $M^{-1}cm^{-1}$)): 392 (1.9), 507 (0.14), 542 (0.13). ESI-TOF-MS: (pos) *m/z* 817.2601. Calcd for $C_{44}H_{36}AuN_4$ ([M – $C_{24}BF_{20}$]$^+$): 817.2600. (neg) *m/z* 678.9778. Calcd for $C_{24}BF_{20}$ ([M – $C_{44}H_{36}AuN_4$]$^-$): 678.9779. This compound was further characterized by single-crystal X-ray diffraction analysis.

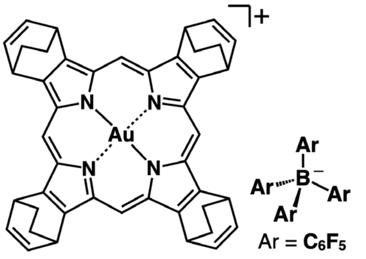

Bicycloporphyrin Au^{III} complex as a BArF$^-$ (tetrakis(3,5-bis(trifluoromethyl)phenyl)borate) ion pair, 2au$^+$-BArF$^-$. NaBArF (18.6 mg, 21.0 µmol) in MeOH (2.4 mL) was added to **2au**$^+$-Cl$^-$ (10.0 mg, 11.7 µmol) in MeOH (9.6 mL), and the reaction mixture was stirred for 15 min. The mixture was purified by silica gel column chromatography (Wakogel C-300; eluent: 3% MeOH/CH_2Cl_2) and was recrystallized from CH_2Cl_2/*n*-hexane to afford **2au**$^+$-BArF$^-$ (15.0 mg, 8.92 µmol, 76%) as a red solid. R_f = 0.43 (25% *n*-hexane/CH_2Cl_2). ^{1}H NMR (600 MHz, CD_3CN, 20 °C): δ(ppm) 10.02/9.95 (br, 4H, *meso*-CH), 8.36/6.93 (br, 8H, CH=CH), 7.69–7.66 (m, 12H, BArF-CH), 6.04–5.86 (m, 8H, CH), 3.8–1.3 (separated broad signals derived from partially associated isomers, 16H, CH_2CH_2). ^{13}C{^{1}H} NMR (151 MHz, CD_3CN, 20 °C): δ (ppm) 162.60 ($J_{13C-11B}$ = 50.1 Hz), 148.54, 138.22, 135.65, 129.90 ($J_{13C-19F}$ = 32.3 Hz), 127.54, 125.45 ($J_{13C-19F}$ = 271.8 Hz), 118.68, 100.00, 37.34, 28.26. ^{19}F NMR (564 MHz, CD_3CN, 20 °C): δ (ppm) –63.29 (s, 24F). UV/vis (CH_2Cl_2, λ_{max}[nm] (ε, 10^5 $M^{-1}cm^{-1}$)): 392 (2.1), 507 (0.15), 541 (0.14). ESI-TOF-MS: (pos) *m/z* 817.2602. Calcd for $C_{44}H_{36}AuN_4$ ([M – $C_{32}H_{12}BF_{24}$]$^+$): 817.2600. (neg) *m/z* 863.0654. Calcd for $C_{32}H_{12}BF_{24}$ ([M – $C_{44}H_{36}AuN_4$]$^-$): 863.0654. This compound was further characterized by single-crystal X-ray diffraction analysis.

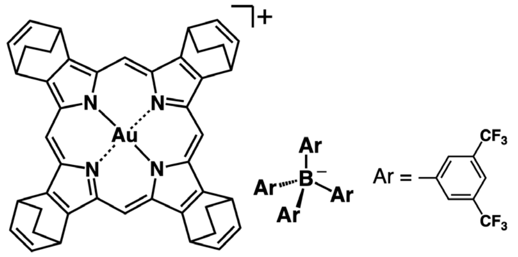

Bicycloporphyrin Au^{III} complex as a PCCp$^-$ ion pair, 2au$^+$-PCCp$^-$. Pentacyanocyclopentadienide as a Na^+ salt (NaPCCp)[S3] (10.8 mg, 50.7 µmol) in MeOH (2.8 mL) was added to **2au**$^+$-Cl$^-$ (42.7 mg, 50.0 µmol) in MeOH (11.2 mL), and the reaction mixture was stirred for 10 min. The mixture was purified by silica gel column chromatography (Wakogel C-300; eluent: 1% MeOH/CH_2Cl_2) and was recrystallized from CH_2Cl_2/MeOH to afford **2au**$^+$-PCCp$^-$ (33.2 mg, 32.8 µmol, 66%) as a red solid. R_f = 0.39 (1% MeOH/CH_2Cl_2). ^{1}H NMR (600 MHz, DMSO-d_6, 20 °C): δ(ppm) 10.28/10.09 (br, 4H, *meso*-CH), 8.41/8.17/6.95 (br, 8H, CH=CH), 6.12/6.00 (br, 8H, CH), 3.8–1.3 (separated broad signals derived from partially associated isomers, 16H, CH_2CH_2). ^{13}C{^{1}H} NMR (151 MHz, DMSO-d_6, 20 °C): δ (ppm) 147.22, 136.95, 126.06, 112.67, 101.30, 99.21, 35.85, 27.37. UV/vis (CH_2Cl_2, λ_{max}[nm] (ε, 10^5 $M^{-1}cm^{-1}$)): 392 (2.0), 507 (0.14), 541 (0.14). ESI-TOF-MS: (pos) *m/z* 817.2602. Calcd for $C_{44}H_{36}AuN_4$ ([M – $C_{10}N_5$]$^+$): 817.2600. (neg) *m/z* 190.0159. Calcd for $C_{10}N_5$ ([M – $C_{44}H_{36}AuN_4$]$^-$): 190.0159.

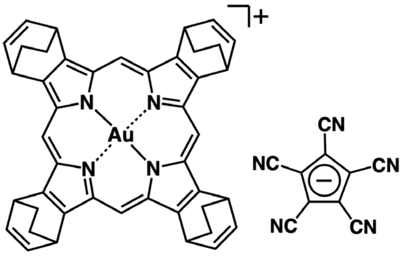

Benzoporphyrin Au^{III} complex as a PF_6^- ion pair, 1au$^+$-PF_6^-. **2au**$^+$-PF_6^- (15.9 mg, 16.5 µmol) was heated at 180 °C for 20 min under vacuum. Crystallization of the residue from CH_2Cl_2/*n*-hexane in the presence of a small amount of CH_3CN afforded **1au**$^+$-PF_6^- (8.5 mg, 10 µmol, 61%) as a blue solid. ^{1}H NMR (600 MHz, DMSO-d_6, 20 °C): δ (ppm) 10.21 (br, 4H, *meso*-CH), 9.19 (br, 8H, benzo-CH), 8.11 (br, 8H, benzo-CH) (The observed signals were very broad due to the aggregation). ^{13}C{^{1}H}

NMR spectrum could not be obtained due to inadequate solubility required for ^{13}C NMR measurement. ^{19}F NMR (564 MHz, DMSO-d_6, 20 °C): δ (ppm) –69.97 (d, $J_{19F-31P}$ = 710.6 Hz, 6F). UV/vis (DMSO, λ_{max}[nm] (ε, 10^5 $M^{-1}cm^{-1}$)): 384 (0.60), 408 (2.4), 616 (1.1). ESI-TOF-MS: (pos) *m/z* 705.1348. Calcd for $C_{44}H_{36}AuN_4$ ([M – F_6P]$^+$): 705.1348. (neg) *m/z* 144.9647. Calcd for F_6P ([M – $C_{44}H_{36}AuN_4$]$^-$): 144.9647.

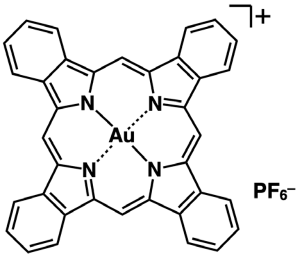

Benzoporphyrin Au^{III} complex as a FABA$^-$ ion pair, 1au$^+$-FABA$^-$. **2au**$^+$-FABA$^-$ (9.59 mg, 6.4 μmol) was heated at 180 °C for 1 h under vacuum. Crystallization of the residue from CH_2Cl_2/*n*-hexane afforded **1au**$^+$-FABA$^-$ (7.90 mg, 5.7 μmol, 89%) as a blue solid. ^{1}H NMR (600 MHz, DMSO-d_6, 20 °C): δ (ppm) 10.21 (br, 4H, *meso*-CH), 9.18 (br, 8H, benzo-CH), 8.11 (br, 8H, benzo-CH) (The observed signals were very broad due to the aggregation). ^{13}C{^{1}H} NMR (151 MHz, DMSO-d_6, 20 °C): δ(ppm) 147.56 ($J_{13C-19F}$ = 235.7 Hz), 137.68 ($J_{13C-19F}$ = 243.0 Hz), 135.64 ($J_{13C-19F}$ = 242.8 Hz), 131.20, 128.58, 126.89, 123.22, 120.71, 94.23. ^{19}F NMR (564 MHz, DMSO-d_6, 20 °C): δ (ppm) –122.12 (br, 8F), –150.90 (t, J = 22.0 Hz, 4F), –155.53 (t, J = 19.2 Hz, 8F). UV/vis (DMSO, λ_{max}[nm] (ε, 10^5 $M^{-1}cm^{-1}$)): 384 (0.63), 408 (2.5), 616 (1.1). ESI-TOF-MS: (pos) *m/z* 705.1348. Calcd for $C_{44}H_{36}AuN_4$ ([M – $C_{24}BF_{20}$]$^+$): 705.1348. (neg) *m/z* 678.9779. Calcd for $C_{24}BF_{20}$ ([M – $C_{44}H_{36}AuN_4$]$^-$): 678.9779. This compound was further characterized by single-crystal X-ray diffraction analysis.

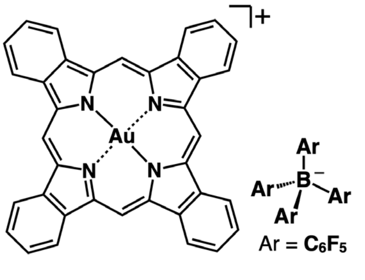

Benzoporphyrin Au^{III} complex as a BArF$^-$ ion pair, 1au$^+$-BArF$^-$. **2au**$^+$-BArF$^-$ (9.7 mg, 5.8 μmol) was heated at 180 °C for 1 h under vacuum. Crystallization of the residue from acetone/*n*-hexane afforded **1au**$^+$-BArF$^-$ (7.6 mg, 4.8 μmol, 83%) as a blue solid. R_f = 0.14 (10% MeOH/CH_2Cl_2). ^{1}H NMR (600 MHz, DMSO-d_6, 20 °C): δ (ppm) 10.27 (br, 4H, *meso*-CH), 9.21 (br, 8H, benzo-CH), 8.12 (br, 8H, benzo-CH), 7.74 (s, 4H, BArF-CH), 7.62 (m, 8H, BArF-CH) (The observed signals were very broad due to the aggregation). ^{13}C{^{1}H} NMR (151 MHz, DMSO-d_6, 80 °C): δ (ppm) 160.68 (q, $J_{13C-11B}$ = 49.7 Hz, BArF-C), 133.73 (s, BArF-C), 131.80, 128.81, 128.55–127.91 (m, BArF-C), 128.06, 123.70 (q, $J_{13C-19F}$ = 273.3 Hz, BArF-C), 120.97, 117.15, 94.66. ^{19}F NMR (564 MHz, DMSO-d_6, 20 °C): δ (ppm) –61.44 (s, 24F). UV/vis (DMSO, λ_{max}[nm] (ε, 10^5 $M^{-1}cm^{-1}$)): 384 (0.61), 408 (2.4), 616 (1.1). ESI-TOF-MS: (pos) *m/z* 705.1348. Calcd for $C_{44}H_{36}AuN_4$ ([M – $C_{32}H_{12}BF_{24}$]$^+$): 705.1348. (neg) *m/z* 863.0654. Calcd for $C_{32}H_{12}BF_{24}$ ([M – $C_{44}H_{36}AuN_4$]$^-$): 863.0654. This compound was further characterized by single-crystal X-ray diffraction analysis.

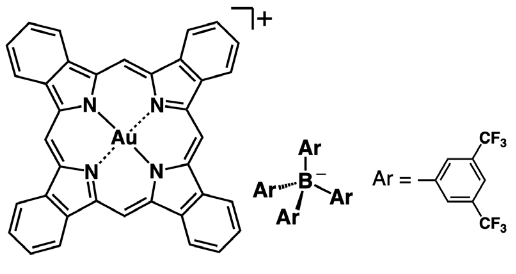

Benzoporphyrin Au^{III} complex as a PCCp$^-$ ion pair, 1au$^+$-PCCp$^-$. **2au**$^+$-PCCp$^-$ (7.09 mg, 7.0 μmol) was heated at 180 °C for 1 h under vacuum. Crystallization of the residue from CH_2Cl_2/DMF/*n*-hexane afforded **1au**$^+$-PCCp$^-$ (4.19 mg, 4.7 μmol, 67%) as a blue solid. ^{1}H NMR (600 MHz, DMSO-d_6, 20 °C): δ (ppm) 10.40 (br, 4H, *meso*-CH), 9.31 (br, 8H, benzo-CH), 8.17 (br, 8H, benzo-CH) (The observed signals were very broad due to the aggregation). ^{13}C{^{1}H} NMR (151 MHz, DMSO-d_6, 80 °C): δ (ppm) 132.32, 128.99, 128.28, 121.24, 112.54, 101.43, 95.07. UV/vis (DMSO, λ_{max}[nm] (ε, 10^5 $M^{-1}cm^{-1}$)): 384 (0.62), 408 (2.5), 616 (1.0). ESI-TOF-MS: (pos) *m/z* 705.1348. Calcd for $C_{44}H_{36}AuN_4$ ([M – $C_{10}N_5$]$^+$): 705.1348. (neg) *m/z* 190.0159. Calcd for $C_{10}N_5$ ([M – $C_{44}H_{36}AuN_4$]$^-$): 190.0159. This compound was further characterized by single-crystal X-ray diffraction analysis.

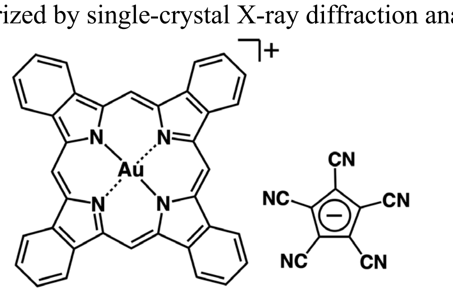

[S1] (a) Y. Haketa, Y. Bando, Y. Sasano, H. Tanaka, N. Yasuda, I. Hisaki and H. Maeda, *iScience*, 2019, **14**, 241–256; (b) H. Tanaka, Y. Haketa, N. Yasuda and H. Maeda, *Chem. Asian J.*, 2019, **14**, 2129–2137; (c) H. Tanaka, Y. Kobayashi, K. Furukawa, Y. Okayasu, S. Akine, N. Yasuda and H. Maeda, *J. Am. Chem. Soc.*, 2022, **144**, 21710–21718; (d) Y. Maruyama, K. Harano, H. Kanai, Y. Ishida, H. Tanaka, S. Sugiura and H. Maeda, *Angew. Chem., Int. Ed.*, 2025, **64**, e202415135.

[S2] S. Ito, T. Murashima and N. Ono, *J. Chem. Soc., Perkin Trans. 1*, 1997, 3161–3165.

[S3] (a) O. W. Webster, *J. Am. Chem. Soc.*, 1965, **87**, 1820–1821; (b) T. Sakai, S. Seo, J. Matsuoka and Y. Mori, *J. Org. Chem.*, 2013, **78**, 10978–10985; (c) Y. Bando, Y. Haketa, T. Sakurai, W. Matsuda, S. Seki, H. Takaya and H. Maeda, *Chem. Eur. J.*, 2016, **22**, 7843–7850.

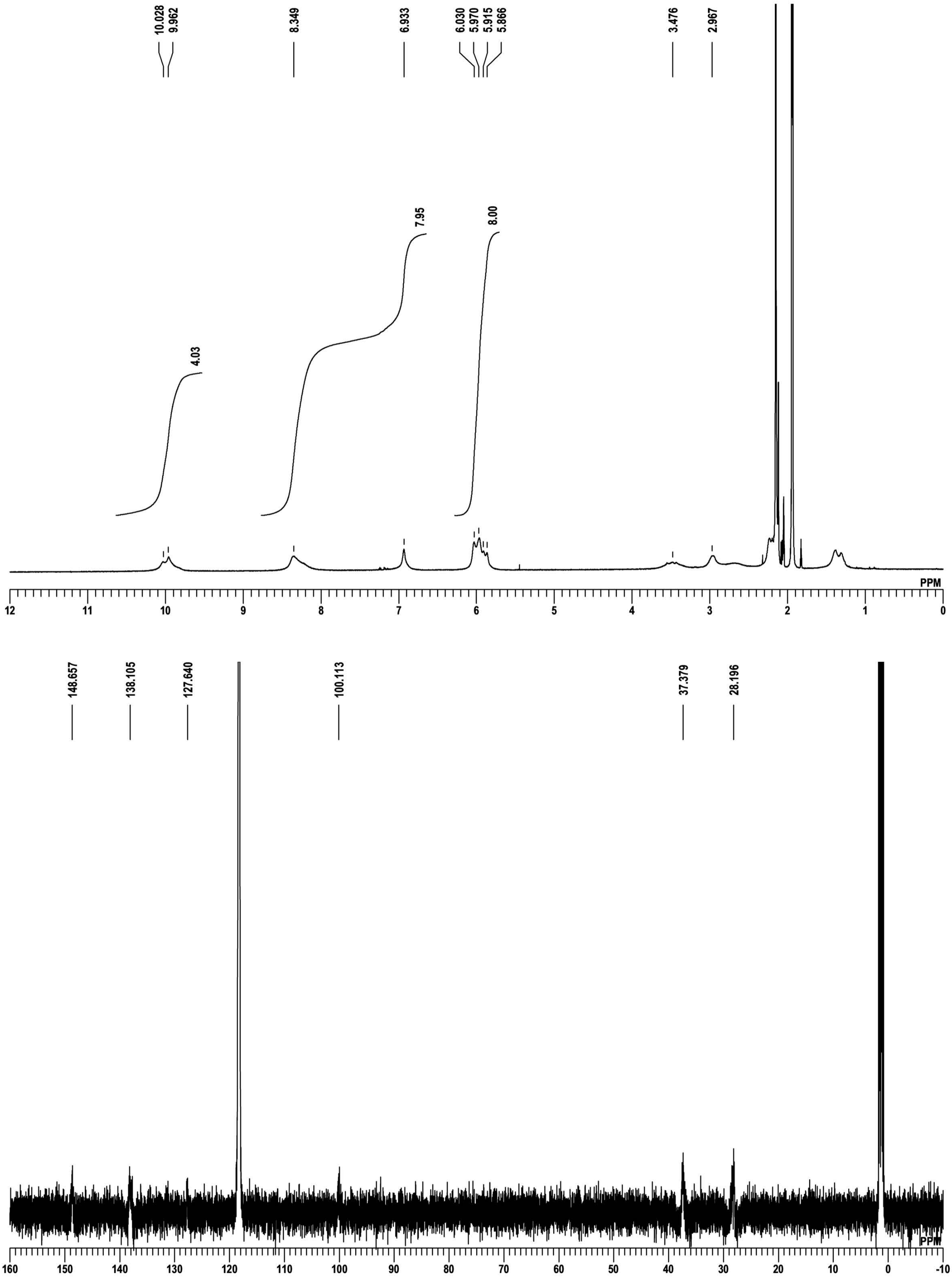

Fig. S2 ^{1}H NMR (top) and $^{13}C\{^1H\}$ NMR (bottom) spectra of **2au**$^+$-Cl$^-$ in CD_3CN at 20 °C. Broad ^{1}H NMR signals are due to the equilibrium between monomers and stacked dimers with several stereoisomers at the bicyclo units. The UV/vis absorption spectra in CH_3CN also suggested the formation of stacked dimer (Fig. S67).

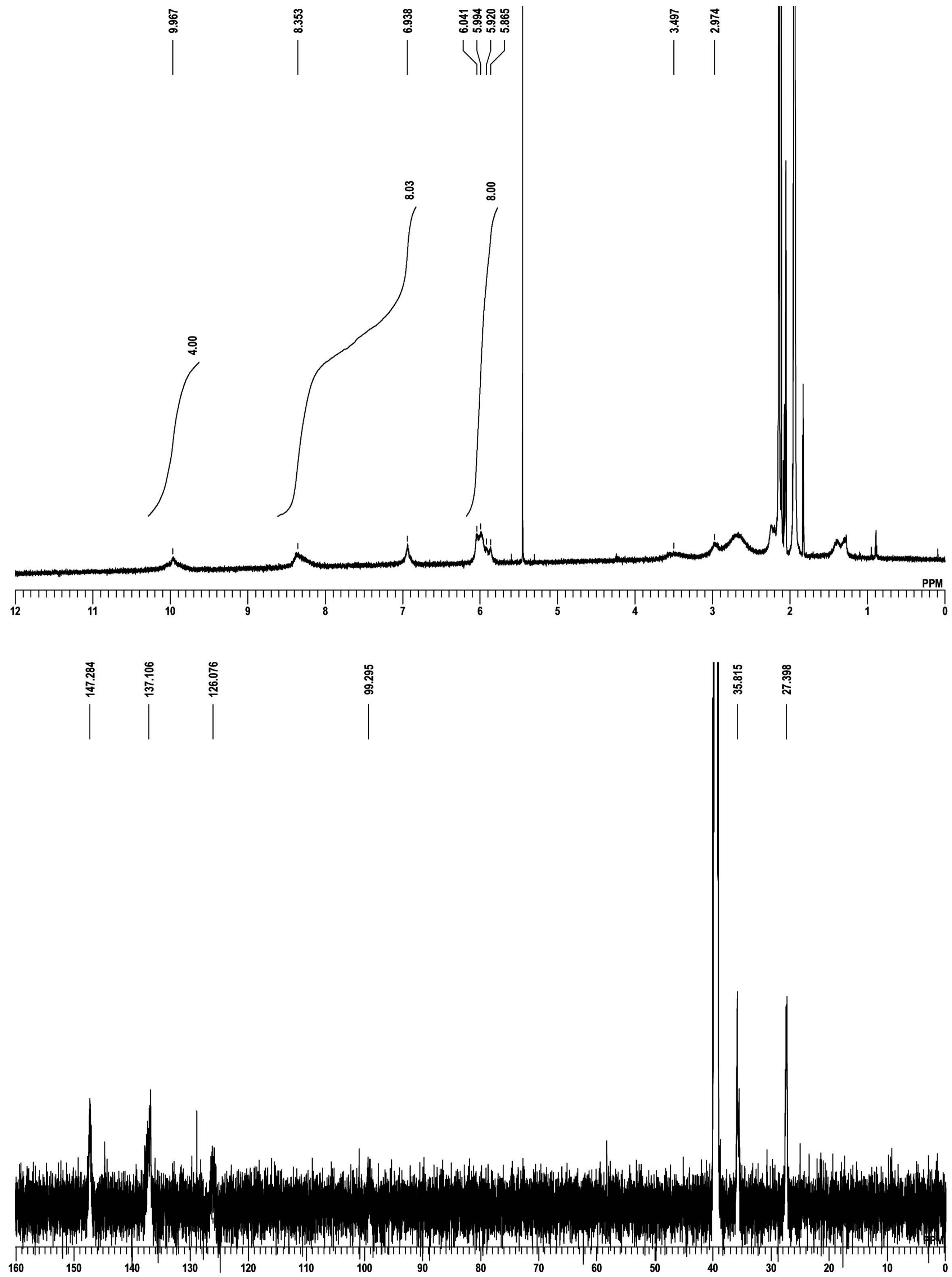

Fig. S3 ^{1}H NMR (top), $^{13}\{^{1}H\}$C NMR (middle), and ^{19}F NMR (bottom) spectra of **2au**$^{+}$-PF_6^{-} in CD_3CN (^{1}H and ^{19}F NMR) and DMSO-d_6 (^{13}C NMR) at 20 °C. Broad ^{1}H NMR signals are due to the equilibrium between monomers and stacked dimers with several stereoisomers at the bicyclo units. The UV/vis absorption spectra in CH_3CN also suggested the formation of stacked dimer (Fig. S67).

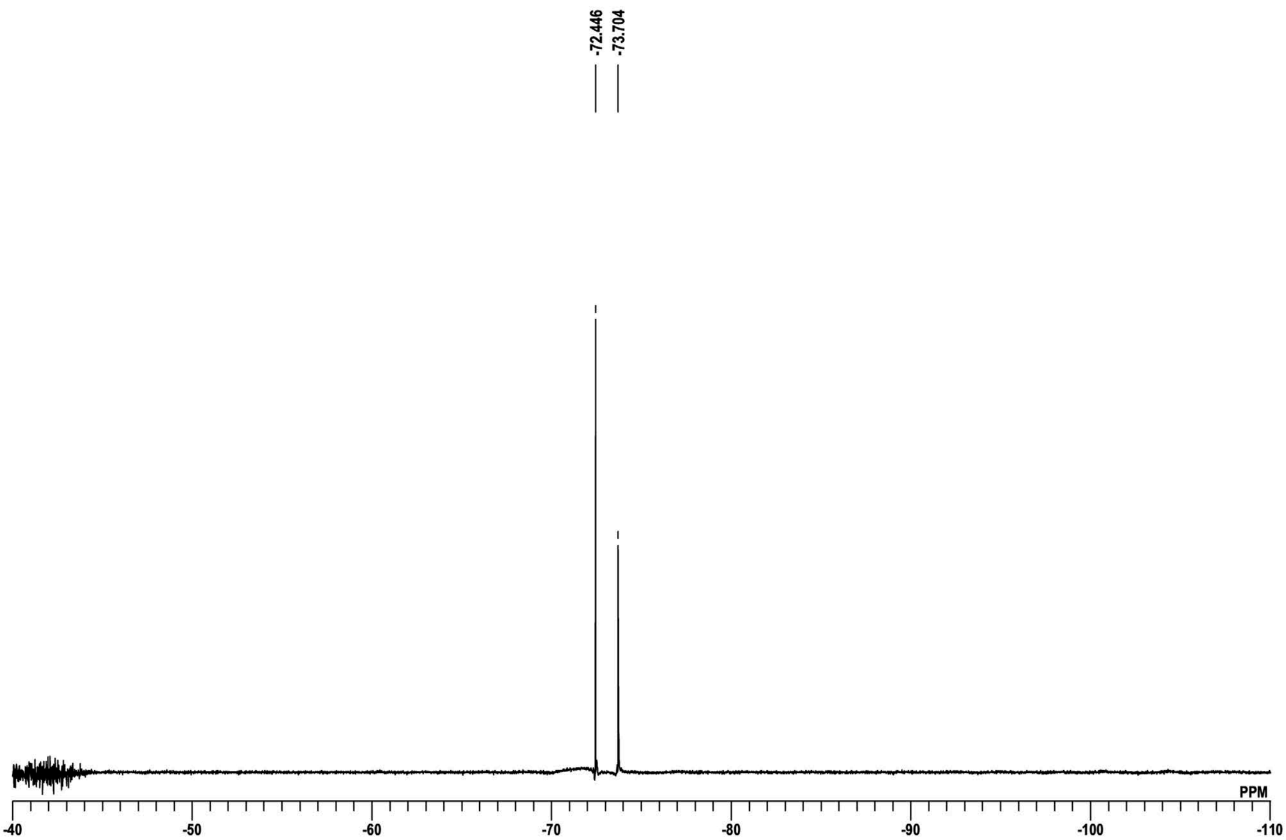

Fig. S3 (Continued)

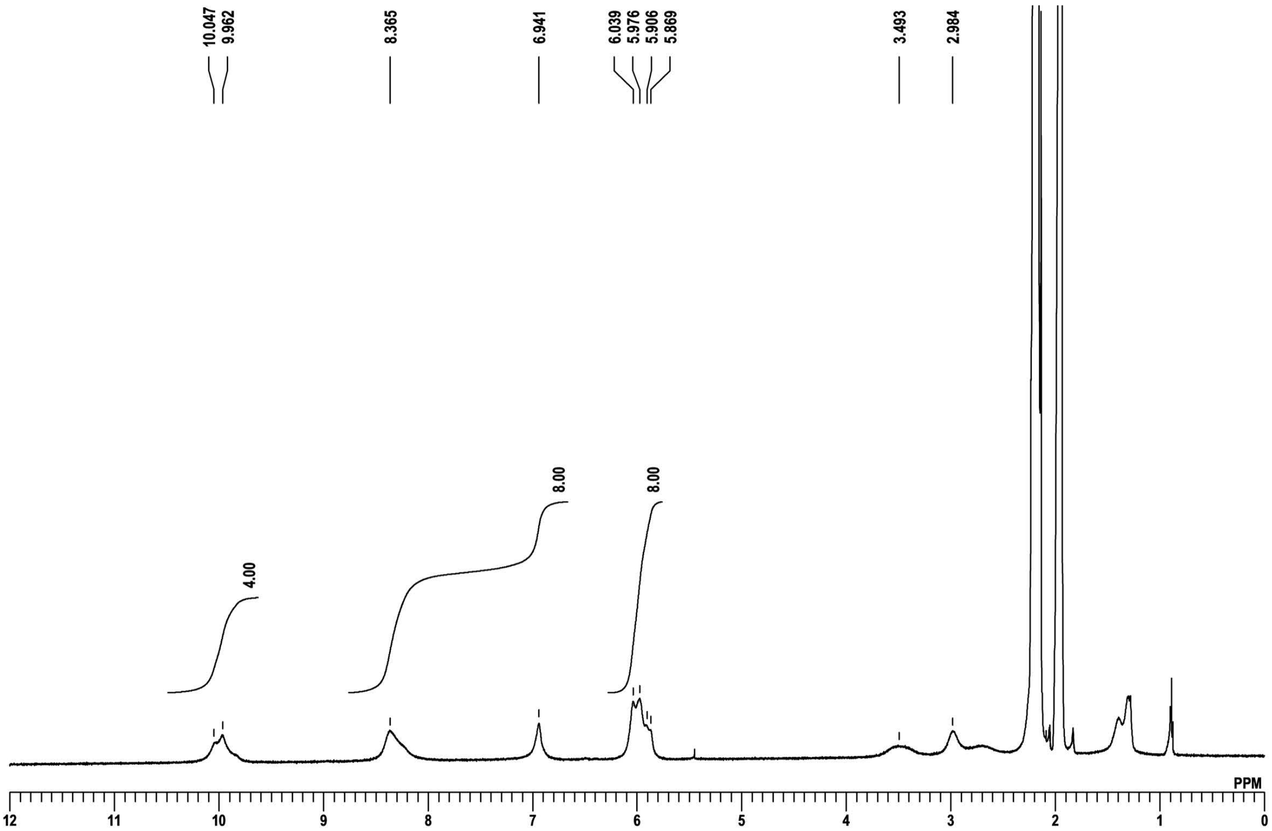

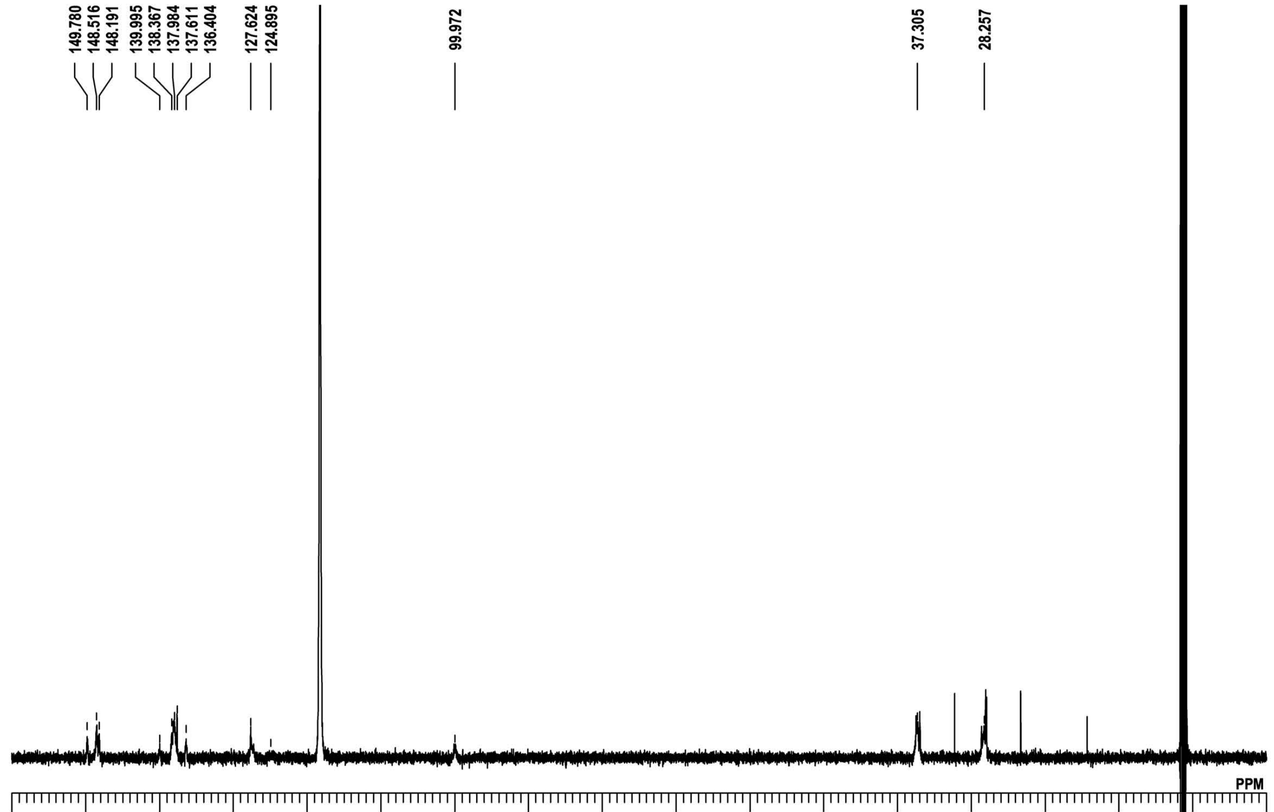

Fig. S4 ^{1}H NMR (top), ^{13}C{^{1}H} NMR (middle), and ^{19}F NMR (bottom) spectra of **2au**$^{+}$-FABA^{-} in CD_3CN at 20 °C. Broad ^{1}H NMR signals are due to the equilibrium of monomers and stacked dimers with several stereoisomers at the bicyclo units. The UV/vis absorption spectra in CH_3CN also suggested the formation of stacked dimer (Fig. S67,69,71).

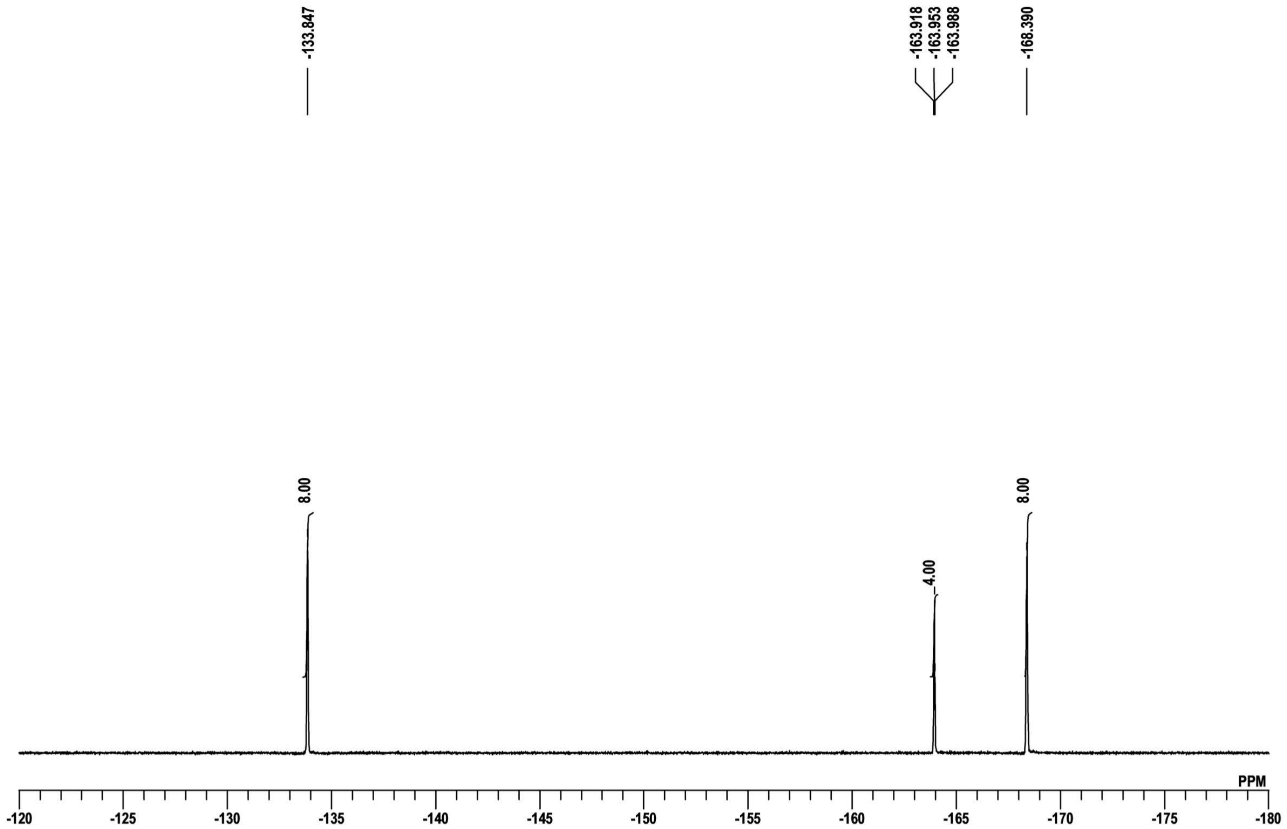

Fig. S4 (Continued)

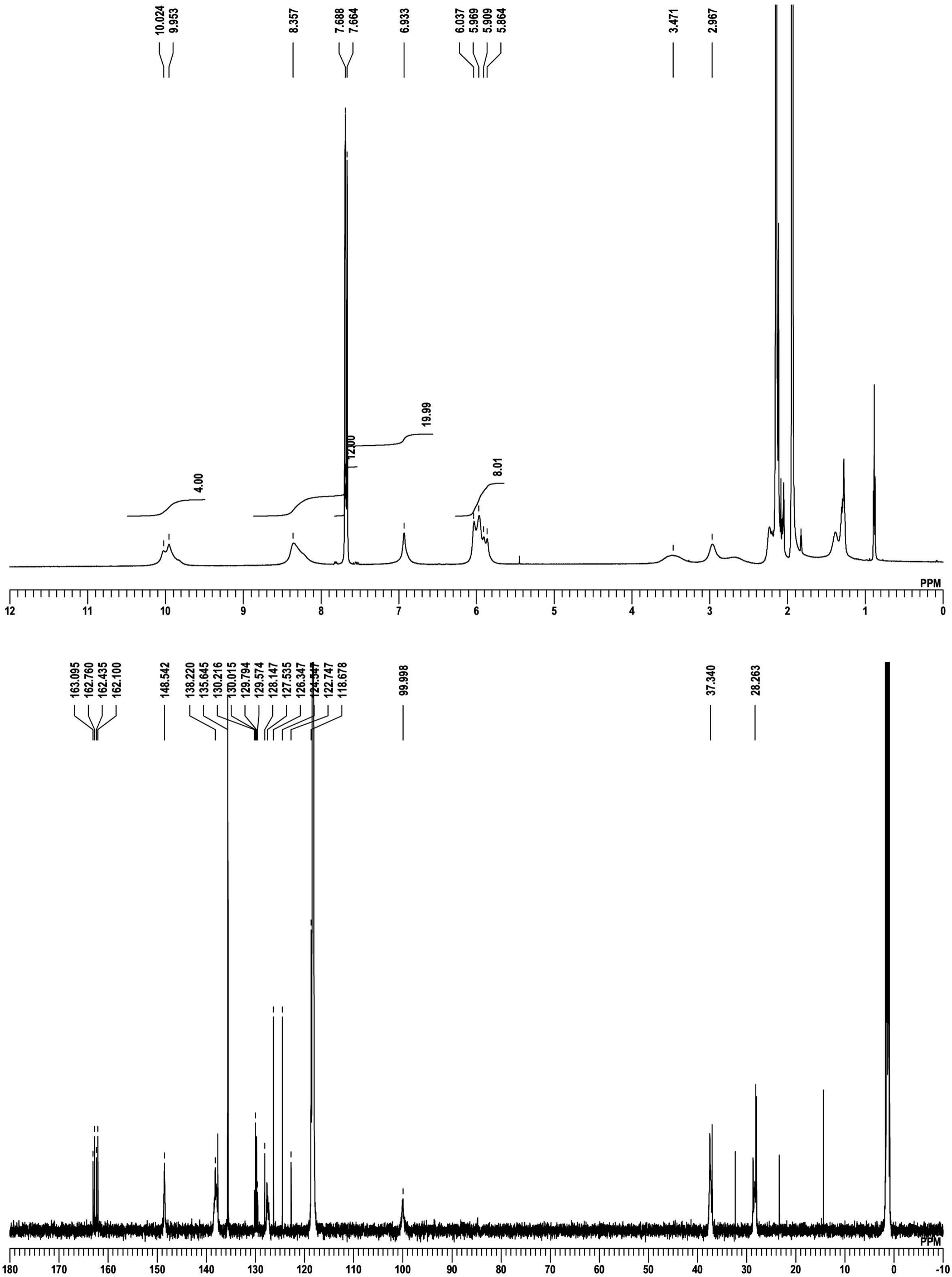

Fig. S5 ^{1}H NMR (top), ^{13}C{^{1}H} NMR (middle), and ^{19}F NMR (bottom) spectra of **2au**$^{+}$-BArF^{-} in CD_3CN at 20 °C. Broad ^{1}H NMR signals are due to the equilibrium between monomers and stacked dimers with several stereoisomers at the bicyclo units. The UV/vis absorption spectra in CH_3CN also suggested the formation of stacked dimer (Fig. S67).

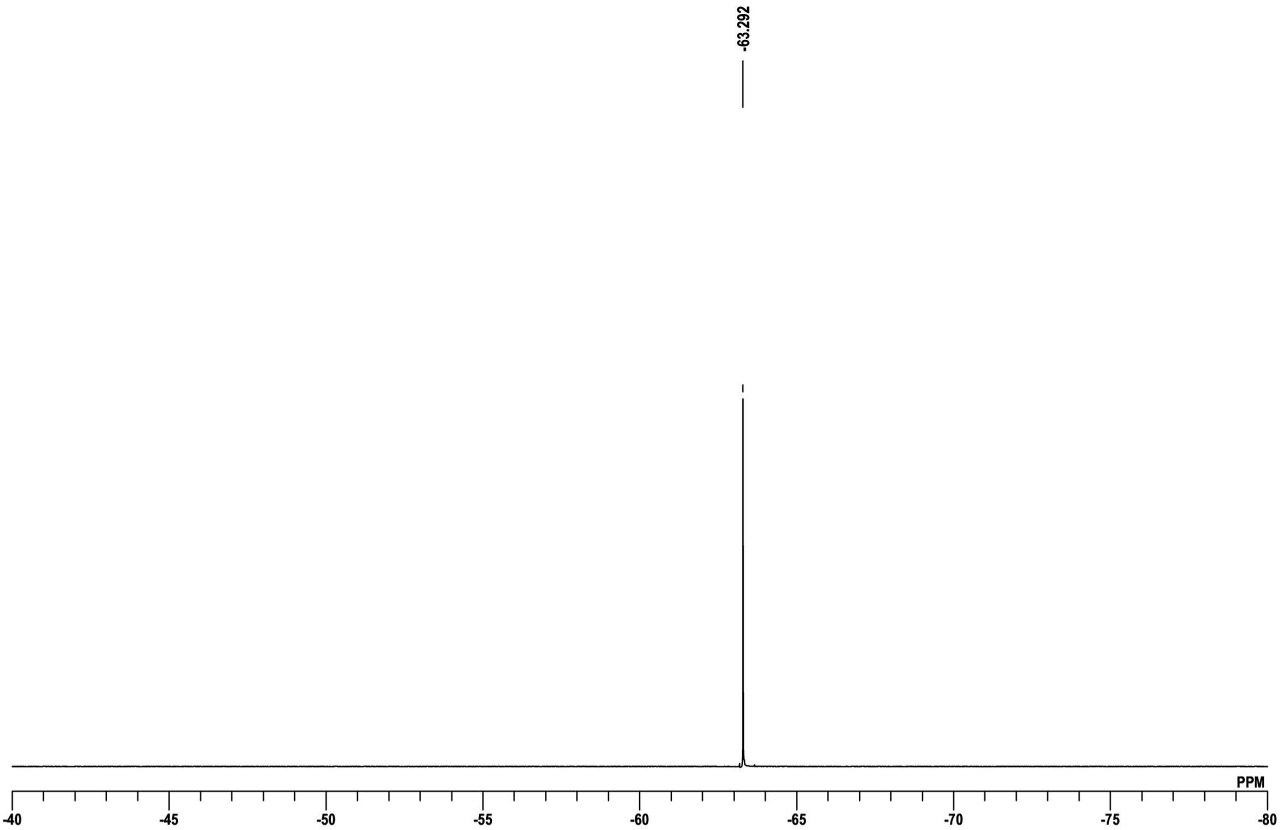

Fig. S5 (Continued)

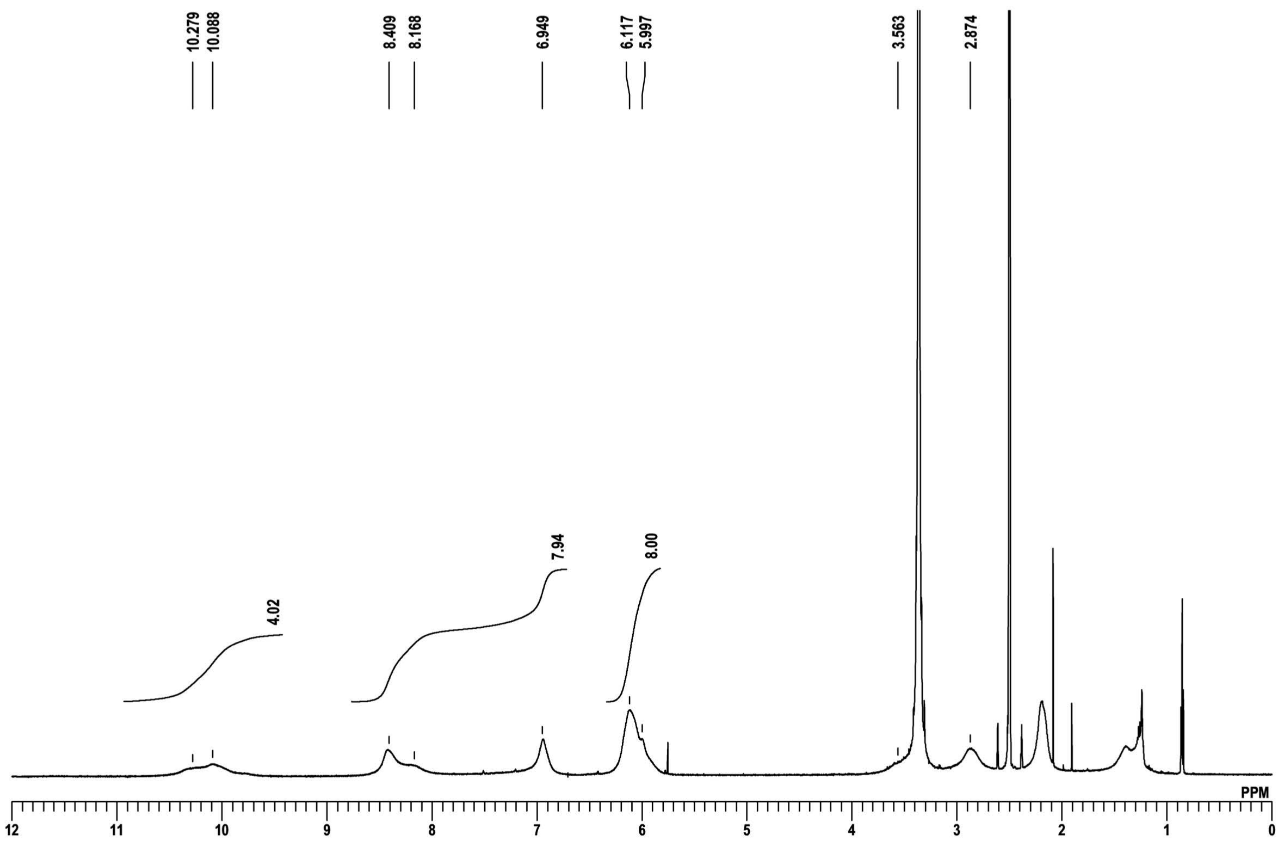

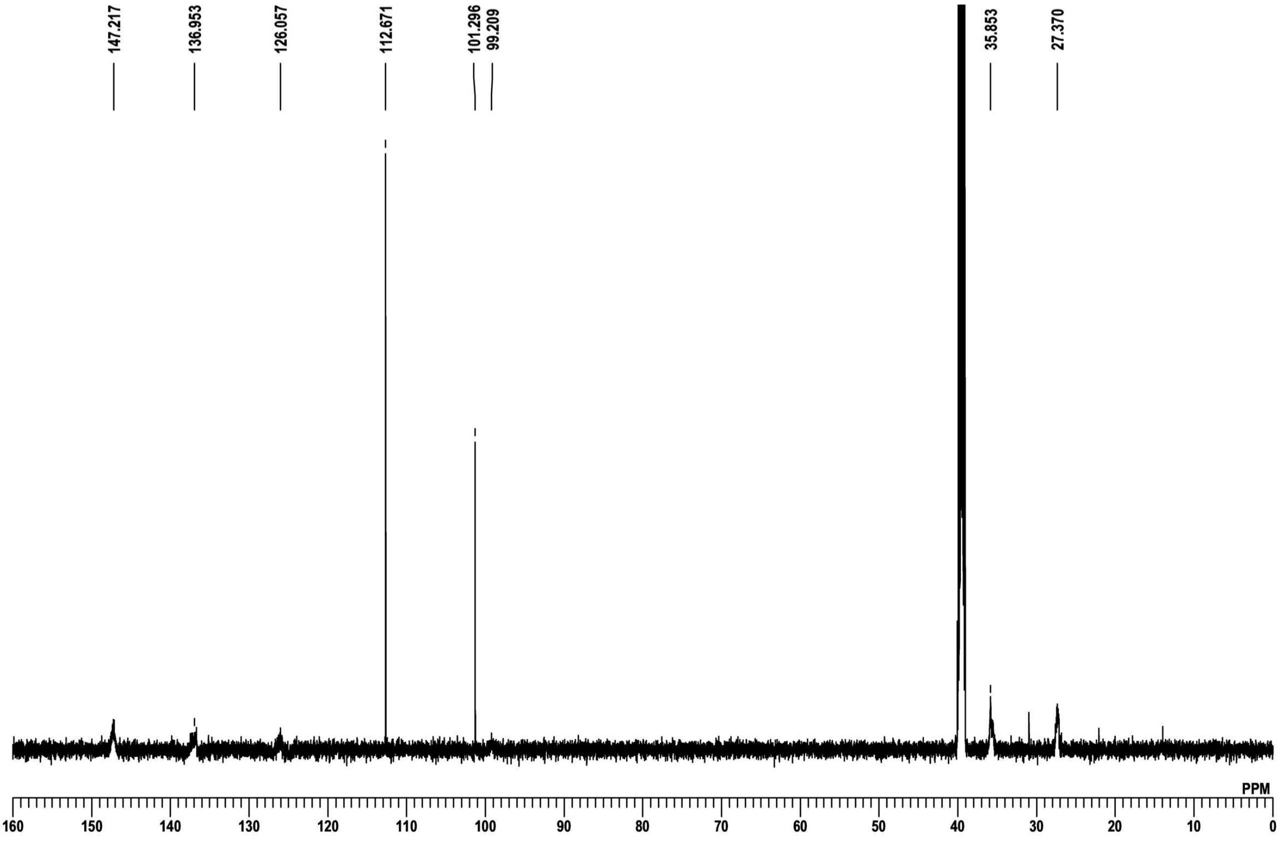

Fig. S6 ^{1}H NMR (top) and $^{13}C\{^{1}H\}$ NMR (bottom) spectra of **2au**$^{+}$-PCCp^{-} in DMSO-d_6 at 20 °C. Broad ^{1}H NMR signals are due to the equilibrium between monomers and stacked dimers with several stereoisomers at the bicyclo units. Such stacked dimers were not formed in the dilute conditions as observed in the UV/vis absorption spectra (Fig. S67).

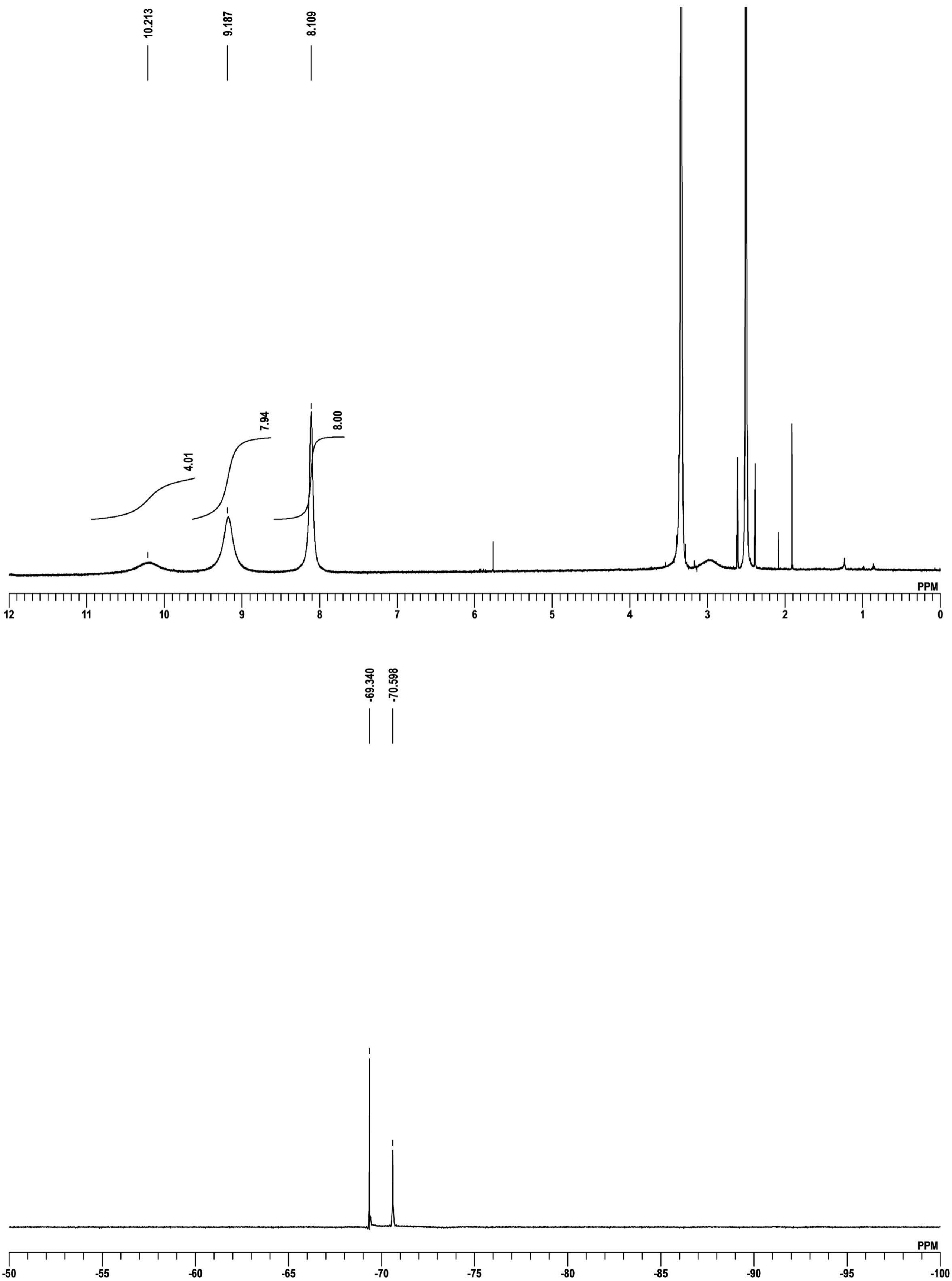

Fig. S7 ^{1}H NMR (top) and ^{19}F NMR (bottom) spectra of **1au**$^+$-PF_6^- in DMSO-*d*6 at 20 °C. Broad ^{1}H NMR signals are due to the equilibrium between monomers and stacked dimers (oligomers). The UV/vis absorption spectra in DMSO also suggested the formation of stacked dimers (oligomers) (Fig. S68).

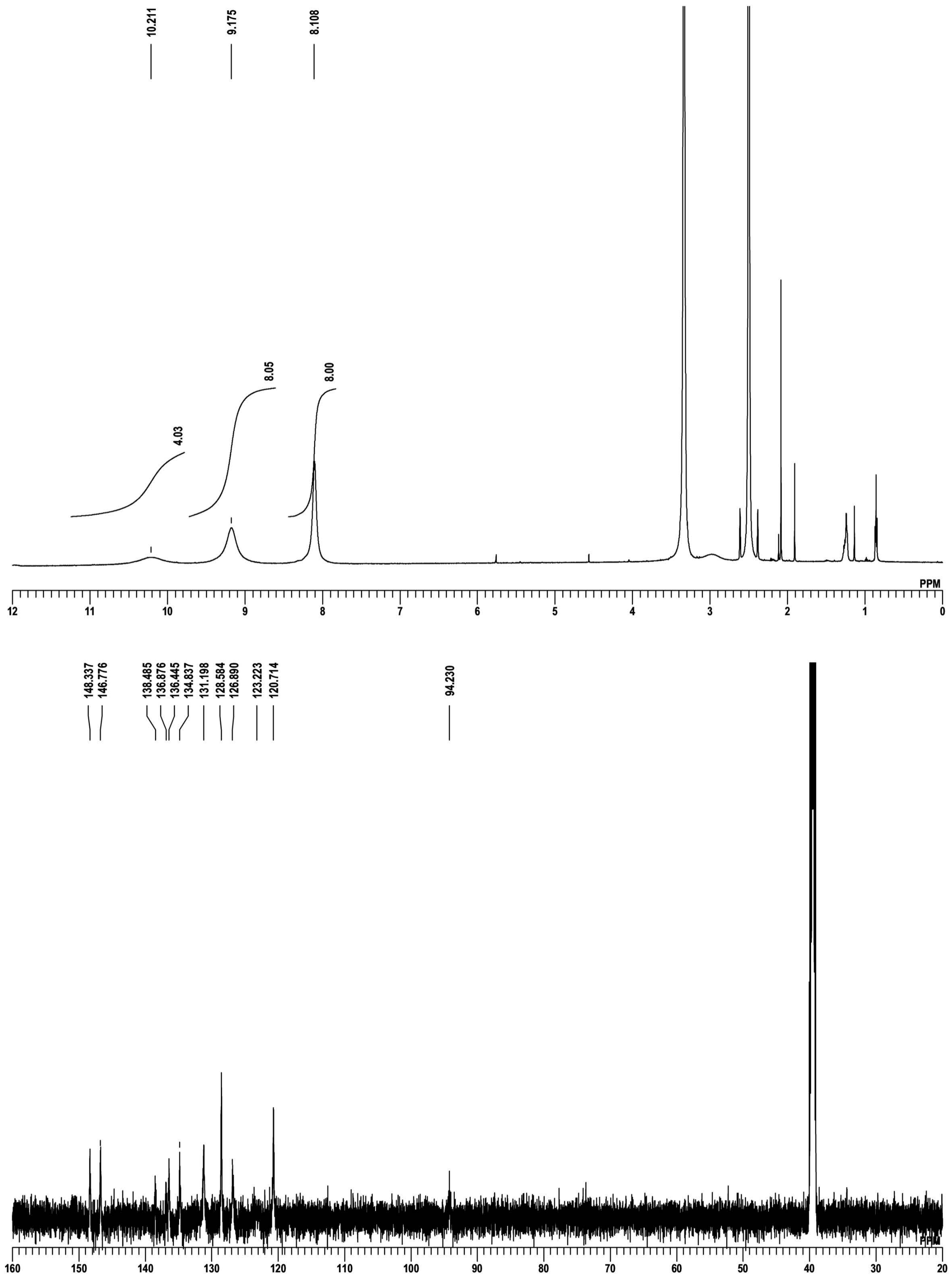

Fig. S8 ^{1}H NMR (top), ^{13}C{^{1}H} NMR (middle), and ^{19}F NMR (bottom) spectra of **1au**$^{+}$-FABA^{-} in DMSO-d_6 at 20 °C. Broad ^{1}H NMR signals are due to the equilibrium between monomers and stacked dimers (oligomers). The UV/vis absorption spectra in DMSO also suggested the formation of stacked dimers (oligomers) (Fig. S68,74).

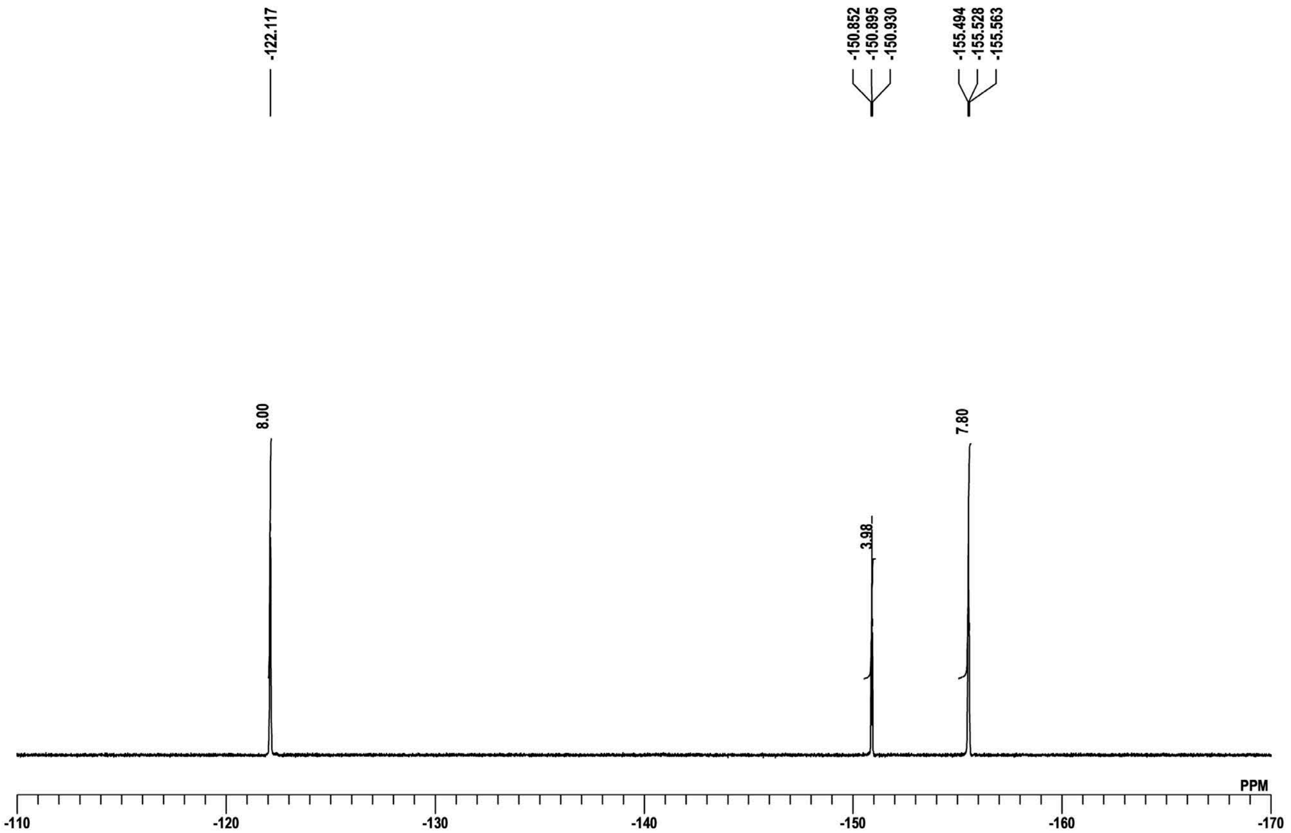

Fig. S8 (Continued)

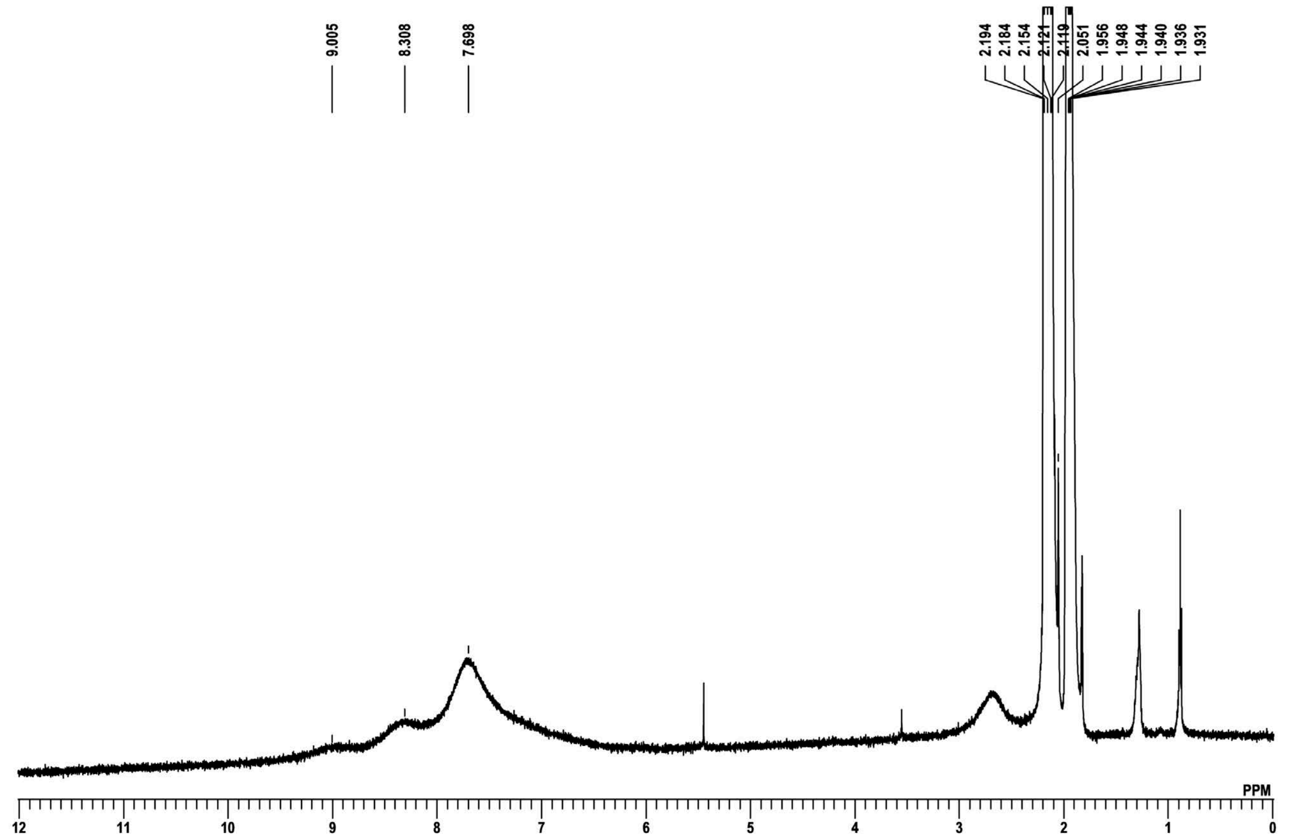

Fig. S9 ^{1}H NMR spectrum of **1au**$^{+}$-FABA^{-} in CD_3CN at 20 °C. Broad ^{1}H NMR signals are due to the equilibrium between monomers and stacked dimers (oligomers). The UV/vis absorption spectra in CH_3CN also suggested the formation of stacked dimers (oligomers) (Fig. S68,70,72).

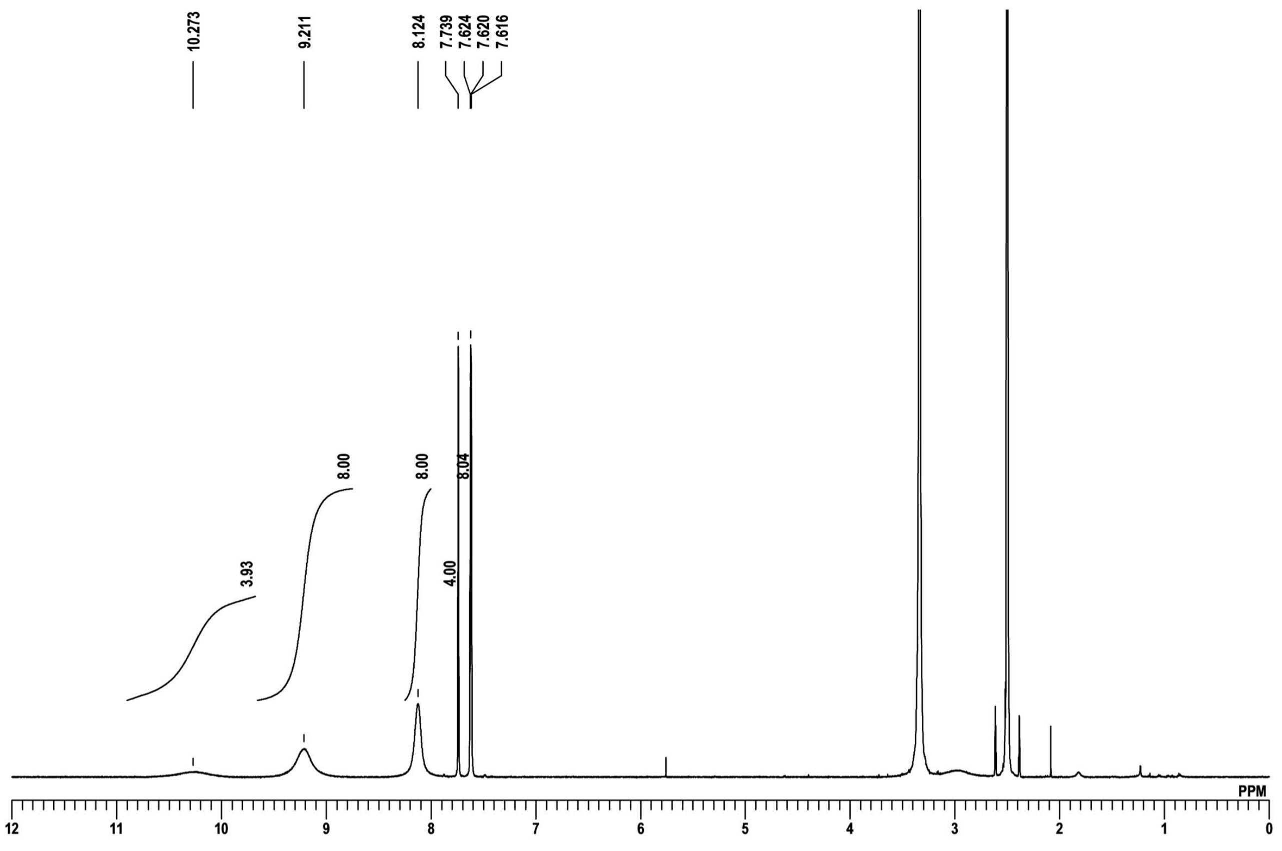

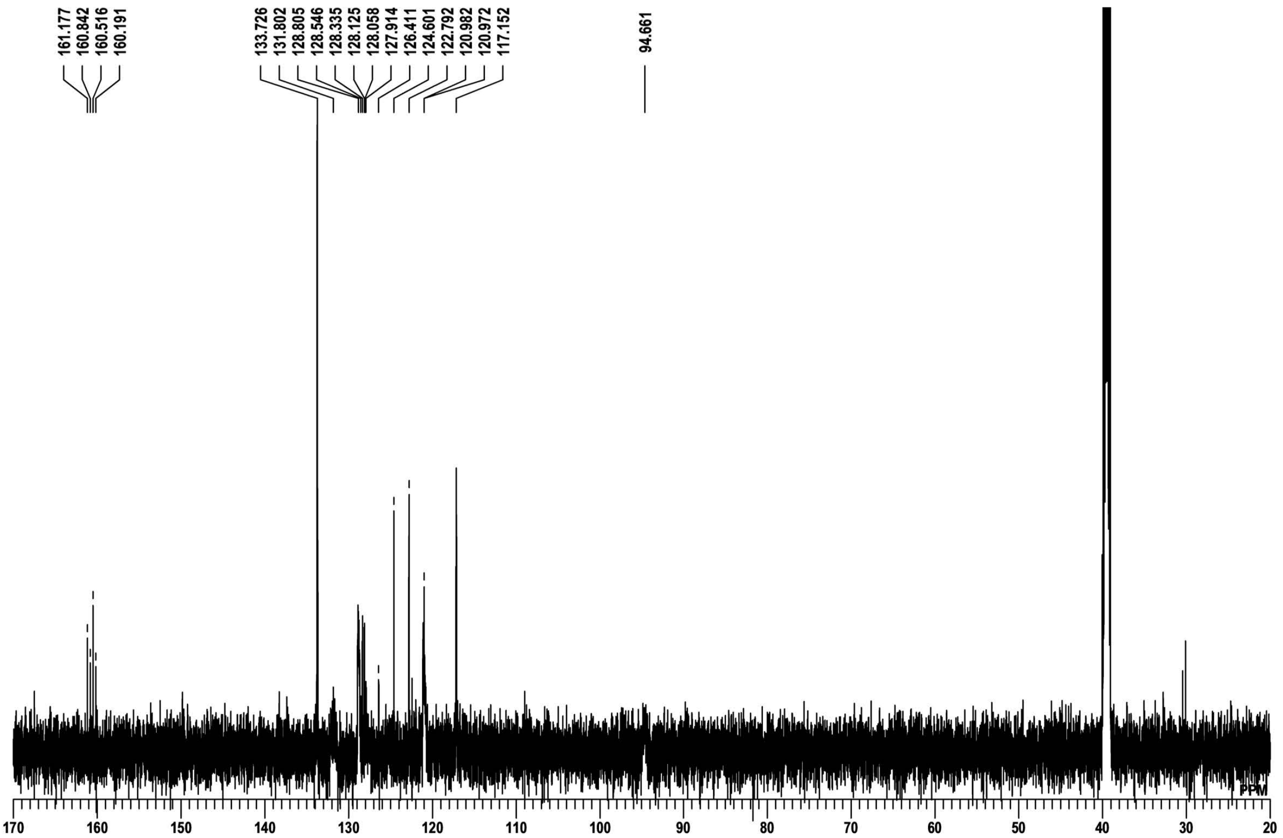

Fig. S10 ^{1}H NMR (top), ^{13}C{^{1}H} NMR (middle), and ^{19}F NMR (bottom) spectra of **1au**$^{+}$-BArF^{-} in DMSO-*d*6 at 20, 80, and 20 °C, respectively. Broad ^{1}H NMR signals are due to the equilibrium between monomers and stacked dimers (oligomers). The UV/vis absorption spectra in DMSO also suggested the formation of stacked dimers (oligomers) (Fig. S68).

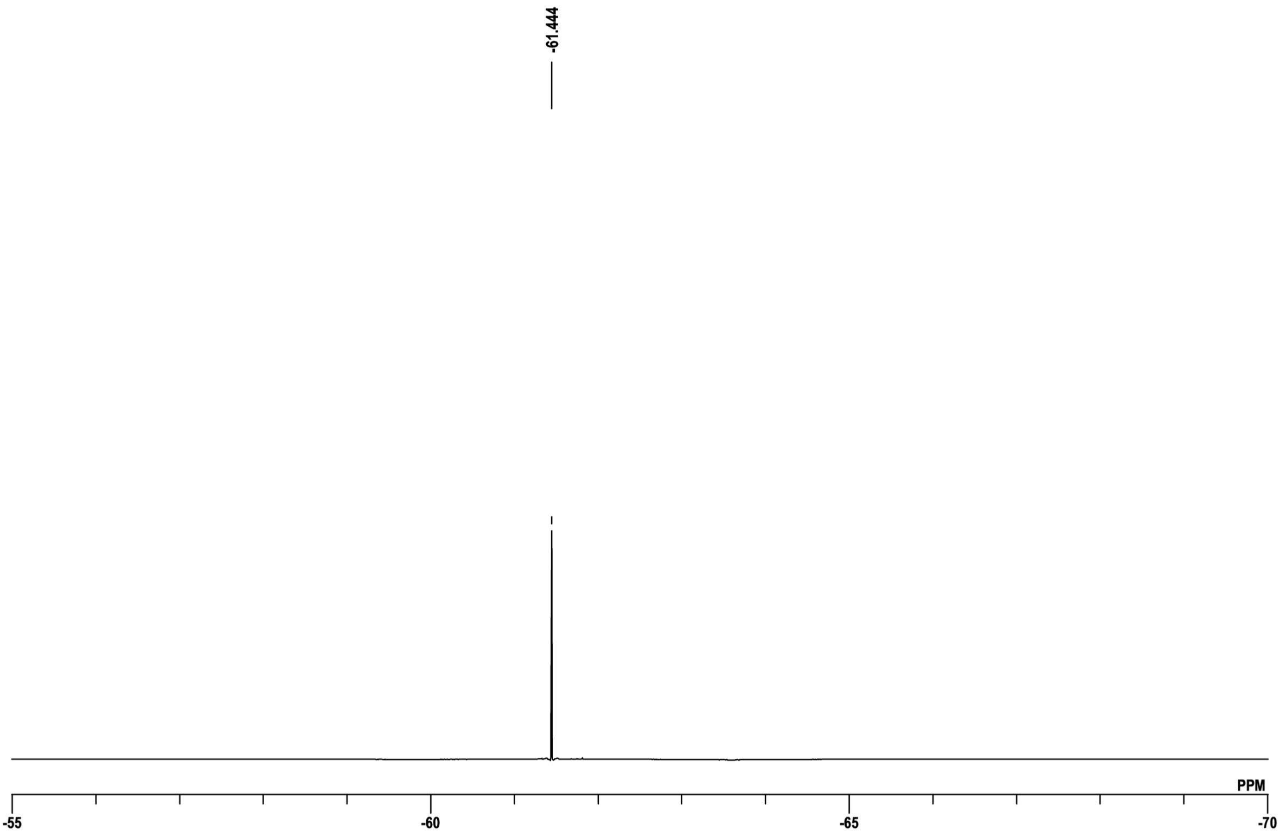

Fig. S10 (Continued)

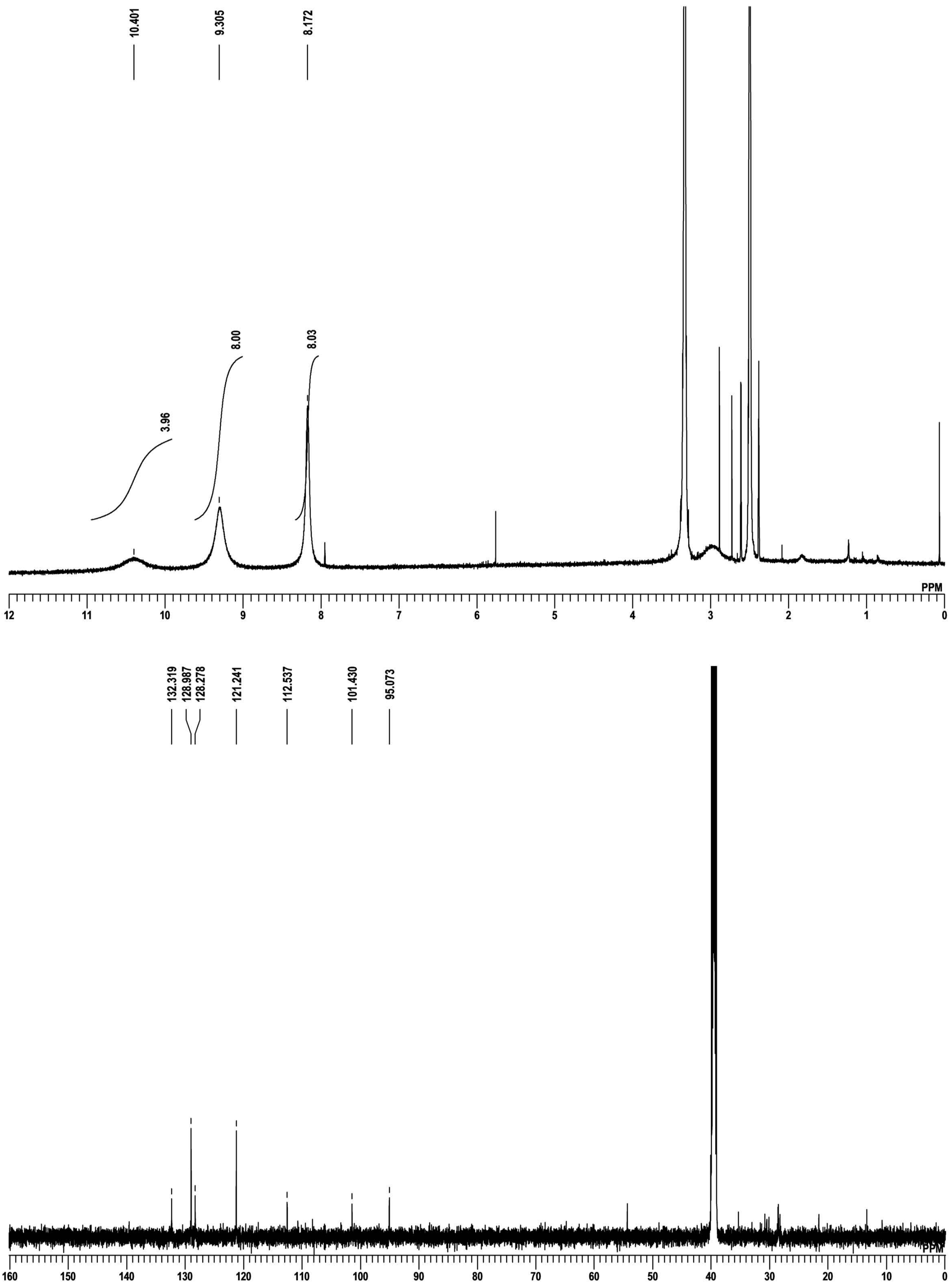

Fig. S11 ^{1}H NMR (top) and ^{13}C{^{1}H} NMR (bottom) spectra of **1au**$^{+}$-PCCp^{-} in DMSO-d_6 at 20 °C and 80 °C, respectively. Broad ^{1}H NMR signals are due to the equilibrium between monomers and stacked dimers (oligomers). The UV/vis absorption spectra in DMSO also suggested the formation of stacked dimers (oligomers) (Fig. S68).

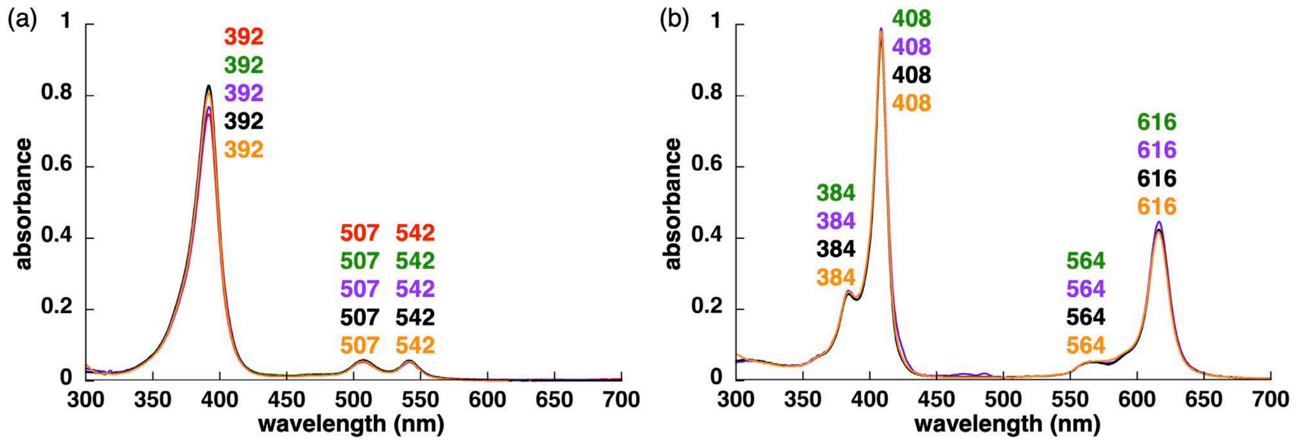

Fig. S12 UV/vis absorption spectra of (a) **2au**$^+$-Cl$^-$ (red), **2au**$^+$-PF_6^- (green), **2au**$^+$-FABA$^-$ (purple), **2au**$^+$-BArF$^-$ (black), and **2au**$^+$-PCCp$^-$ (orange) in CH_2Cl_2 (4 μM) and (b) **1au**$^+$-PF_6^- (green), **1au**$^+$-FABA$^-$ (purple), **1au**$^+$-BArF$^-$ (black), and **1au**$^+$-PCCp$^-$ (orange) in DMSO (4 μM). The ion pairs show the monomeric state as a major species in these solvents, whereas the formation of stacked dimers can be observed in other solvents (Fig. S67–74).

2. X-ray crystallographic data

Method for single-crystal X-ray analysis. Crystallographic data are summarized in Table S1. A single crystal of **2au**$^+$-FABA$^-$ was obtained by vapor diffusion of water into an CH_3CN solution. The data crystal was a red prism of approximate dimensions 0.50 mm × 0.20 mm × 0.20 mm. A single crystal of **2au**$^+$-BArF$^-$ was obtained by vapor diffusion of water into an CH_3CN solution. The data crystal was a red prism of approximate dimensions 0.03 mm × 0.03 mm × 0.01 mm. A single crystal of **1au**$^+$-FABA$^-$ was obtained by vapor diffusion of $CHCl_3$ into an CH_3CN solution. The data crystal was a blue prism of approximate dimensions 0.02 mm × 0.01 mm × 0.01 mm. A single crystal of **1au**$^+$-FABA$^-$ obtained by vapor diffusion of 1,2-dichloroethane into an acetone solution showed the packing structure similar to that of the single crystal obtained from $CH_3CN/CHCl_3$. A single crystal of **1au**$^+$-BArF$^-$ was obtained by vapor diffusion of *n*-heptane into a 1,1,1-trichloroethane solution. The data crystal was a blue prism of approximate dimensions 0.117 mm × 0.026 mm × 0.015 mm. A single crystal of **1au**$^+$-PCCp$^-$ was obtained by vapor diffusion of *o*-dichlorobenzene into a DMF solution. The data crystal was a purple prism of approximate dimensions 0.10 mm × 0.01 mm × 0.01 mm. The data of **2au**$^+$-FABA$^-$, **2au**$^+$-BArF$^-$, and **1au**$^+$-FABA$^-$ were collected at 90 K on a Bruker D8 Venture diffractometer with MoKα radiation (λ = 0.71073 Å) focused by multilayer confocal mirror, whereas that of **1au**$^+$-BArF$^-$ was collected at 90 K on a DECTRIS PILATUS3 CdTe 1M diffractometer with Si (311) monochromated synchrotron radiation (λ = 0.4136 Å) at BL02B1 (SPring-8) and that of **1au**$^+$-PCCp$^-$ was collected at 100 K on a DECTRIS EIGER X 1M diffractometer with Si (111) monochromated synchrotron radiation (λ = 0.8120 Å) at BL40XU (SPring-8).[S4] All the structures were solved by dual-space method. The structures were refined by a full-matrix least-squares method by using a SHELXL 2014[S5] (Yadokari-XG).[S6] In each structure, the non-hydrogen atoms were refined anisotropically. CIF files (CCDC-2387040–2387044) can be obtained free of charge from the Cambridge Crystallographic Data Centre via www.ccdc.cam.ac.uk/data_request/cif.

Table S1 Crystallographic details.

	**2au**$^+$-FABA$^-$	**2au**$^+$-BArF$^-$	**1au**$^+$-FABA$^-$	**1au**$^+$-BArF$^-$	**1au**$^+$-PCCp$^-$
formula	$C_{44}H_{36}N_4Au\cdot C_{24}BF_{20}\cdot 0.5CH_3CN$	$C_{44}H_{36}N_4Au\cdot C_{32}H_{12}BF_{24}\cdot 2CH_3CN$	$C_{36}H_{20}N_4Au\cdot C_{24}BF_{20}$	$C_{36}H_{20}AuN_4\cdot C_{32}H_{11}BF_{24}\cdot 1.33C_2H_3Cl_3$	$C_{36}H_{20}N_4Au\cdot C_{10}N_5$
fw	1517.31	1763.07	1384.58	1746.60	895.68
crystal size, mm	0.50× 0.20 × 0.20	0.03 × 0.03 × 0.01	0.02 × 0.01 × 0.01	0.117 × 0.026 × 0.015	0.10 × 0.01 × 0.01
crystal system	monoclinic	monoclinic	orthorhombic	monoclinic	orthorhombic
space group	$P2_1/c$ (no. 14)	*C*2/*c* (no.15)	*Pnna* (no.52)	*C*2/*c* (no.15)	*Pbca* (no.61)
a, Å	29.740(4)	35.803(6)	7.6381(3)	25.853(3)	7.2275(2)
b, Å	16.255(2)	21.342(4)	27.9614(11)	37.935(4)	29.8407(8)
c, Å	24.079(4)	20.798(3)	28.9791(13)	20.488(2)	30.6964(9)
α, °	90	90	90	90	90
β, °	98.901(6)	116.122(4)	90	90.017(6)	90
γ, °	90	90	90	90	90
V, Å^3	11500(3)	14269(4)	6189.1(4)	20094(4)	6620.4(3)
ρ_{calcd}, g cm^{-3}	1.753	1.641	1.486	1.731	1.797
Z	8	8	4	12	8
T, K	90(2)	90(2)	90(2)	90(2)	100(2)
μ, mm^{-1}	2.674 ^{a}	2.176 ^{a}	2.476 ^{a}	0.583 ^{b}	6.261 ^{b}
no. of reflns	115352	130935	103402	286290	65434
no. of unique reflns	20262	12602	5480	22604	6063
variables	1721	1065	384	1403	505
λ, Å	0.71073 ^{a}	0.71073 ^{a}	0.71073 ^{a}	0.4136 ^{b}	0.8120 ^{b}
R_1 ($I > 2\sigma(I)$)	0.0626	0.0300	0.0521	0.0583	0.0495
wR_2 ($I > 2\sigma(I)$)	0.1546	0.0766	0.1508	0.1284	0.1313
GOF	1.047	1.073	1.206	1.075	1.004

^{a} MoKα radiation. ^{b} Synchrotron radiation.

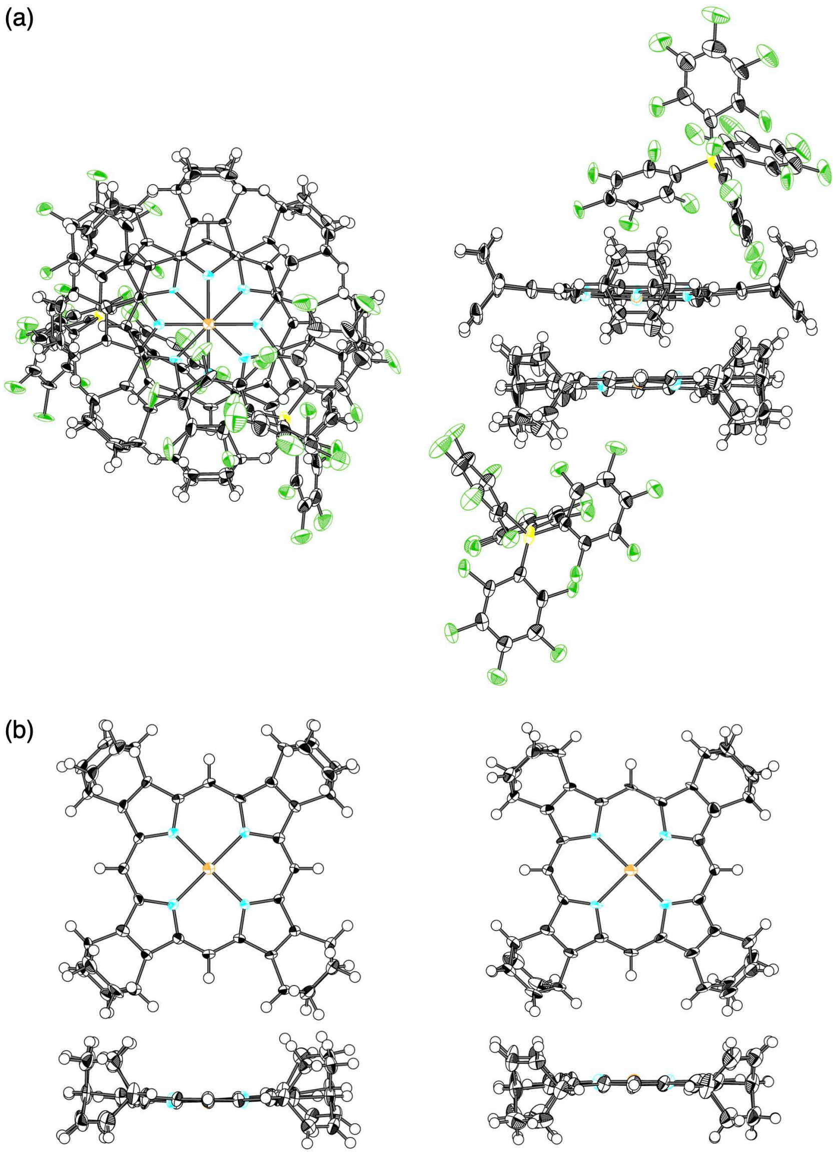

Fig. S13 Ortep drawing of single-crystal X-ray structure of **2au**$^+$-FABA$^-$: (a) stacked dimer including FABA$^-$ (top and side views) and (b) two independent structures of **2au**$^+$ (top and side views). Thermal ellipsoids are scaled to the 50% probability level. The saturated and unsaturated C–C bonds of the bicyclic units showed the distances of 1.40–1.48 Å, which are intermediate between single and double bonds, indicating the disordered structures. Stacked dimer consisted of two independent **2au**$^+$. Solvent molecules are omitted for clarity. Atom color code: black, white (sphere), yellow, blue, green, and orange refer to carbon, hydrogen, boron, nitrogen, fluorine, and gold, respectively.

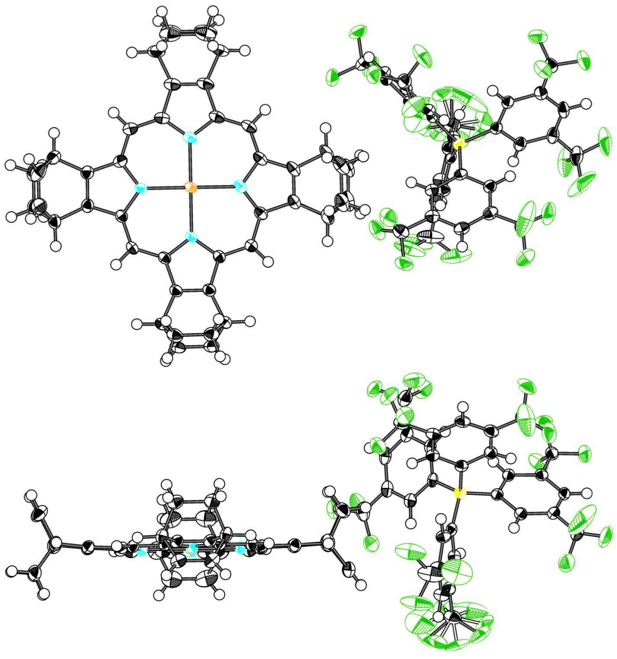

Fig. S14 Ortep drawing of single-crystal X-ray structure (top and side views) of **2au**$^{+}$-BArF^{-}. Thermal ellipsoids are scaled to the 50% probability level. A CF_3 unit has disordered structures in the ratio of 53:28:19 represented by black and white bonds for major and two minor structures, respectively. The saturated and unsaturated C–C bonds of the bicyclic units showed the distances of 1.44–1.51 Å, which are intermediate between single and double bonds, indicating the disordered structures. Solvent molecules are omitted for clarity. Atom color code: black, white (sphere), yellow, blue, green, and orange refer to carbon, hydrogen, boron, nitrogen, fluorine, and gold, respectively.

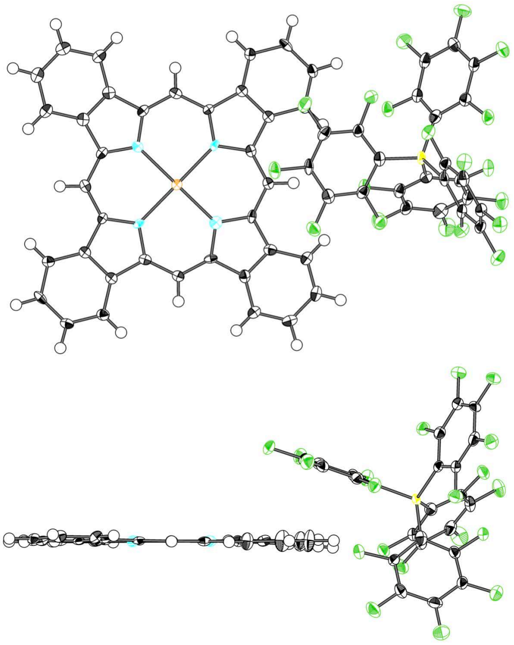

Fig. S15 Ortep drawing of single-crystal X-ray structure (top and side views) of **1au**$^{+}$-FABA^{-}. Thermal ellipsoids are scaled to the 50% probability level. Atom color code: black, white (sphere), yellow, blue, green, and orange refer to carbon, hydrogen, boron, nitrogen, fluorine, and gold, respectively.

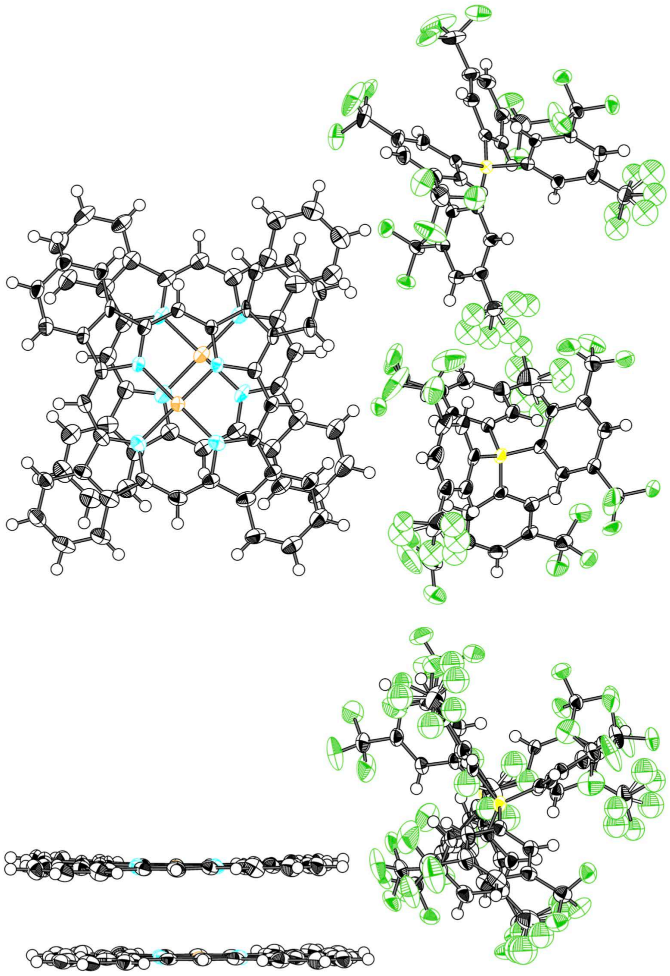

Fig. S16 Ortep drawing of single-crystal X-ray structure (top and side views in the form of the stacked dimers) of **1au**$^{+}$-BArF^{-}. Thermal ellipsoids are scaled to the 50% probability level. Three CF_3 units have disordered structures in the ratio of 50.5:49.5, 46.7:53.3, and 55:45 represented by black and white bonds for major and minor structures, respectively. Solvent molecules are omitted for clarity. Atom color code: black, white (sphere), yellow, blue, green, and orange refer to carbon, hydrogen, boron, nitrogen, fluorine, and gold, respectively.

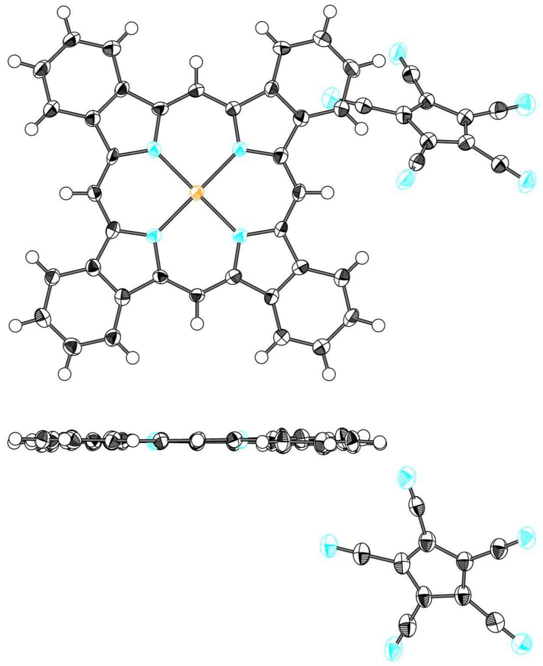

Fig. S17 Ortep drawing of single-crystal X-ray structure (top and side views) of **1au**$^+$-PCCp$^-$. Thermal ellipsoids are scaled to the 50% probability level. Atom color code: black, white (sphere), blue, and orange refer to carbon, hydrogen, nitrogen, and gold, respectively.

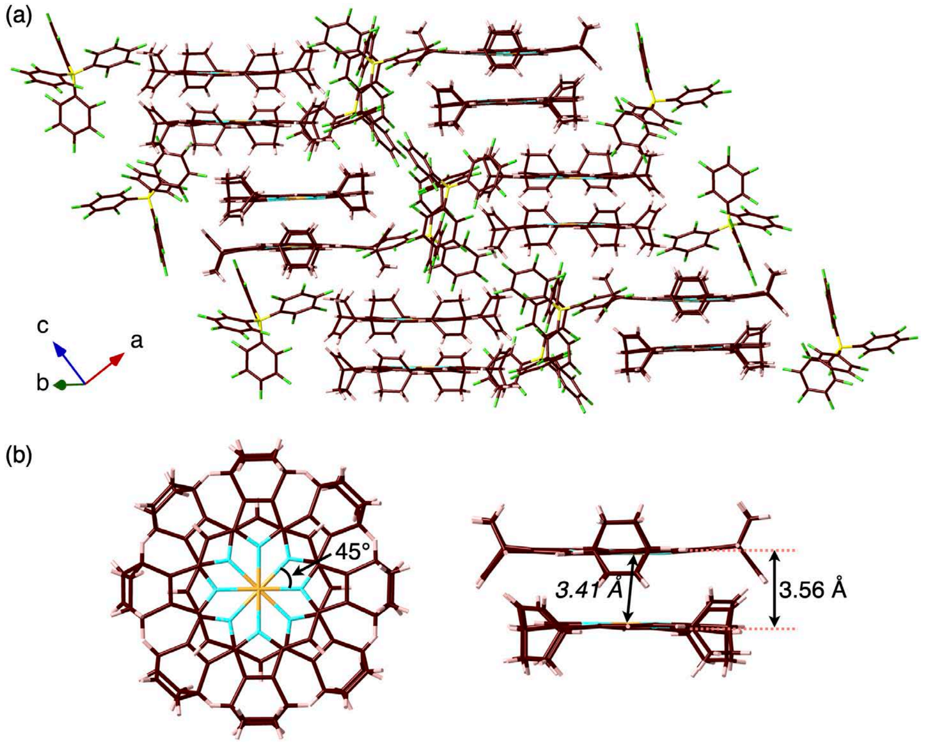

Fig. S18 (a) Packing diagram of **2au**$^+$-FABA$^-$ and (b) the stacked dimer structure as top and side views. In the stacked dimer, face-to-face (between two mean planes comprising core 25 atoms for each) and Au···Au (italic) distances are 3.56 and 3.41 Å, respectively, and rotation angle is nearly 45°. Mean-plane deviations for core 25 atoms of **2au**$^+$ are 0.052 and 0.068 Å. Solvent molecules are omitted for clarity. Atom color code: brown, pink, blue, green, and orange refer to carbon, hydrogen, nitrogen, fluorine, and gold, respectively.

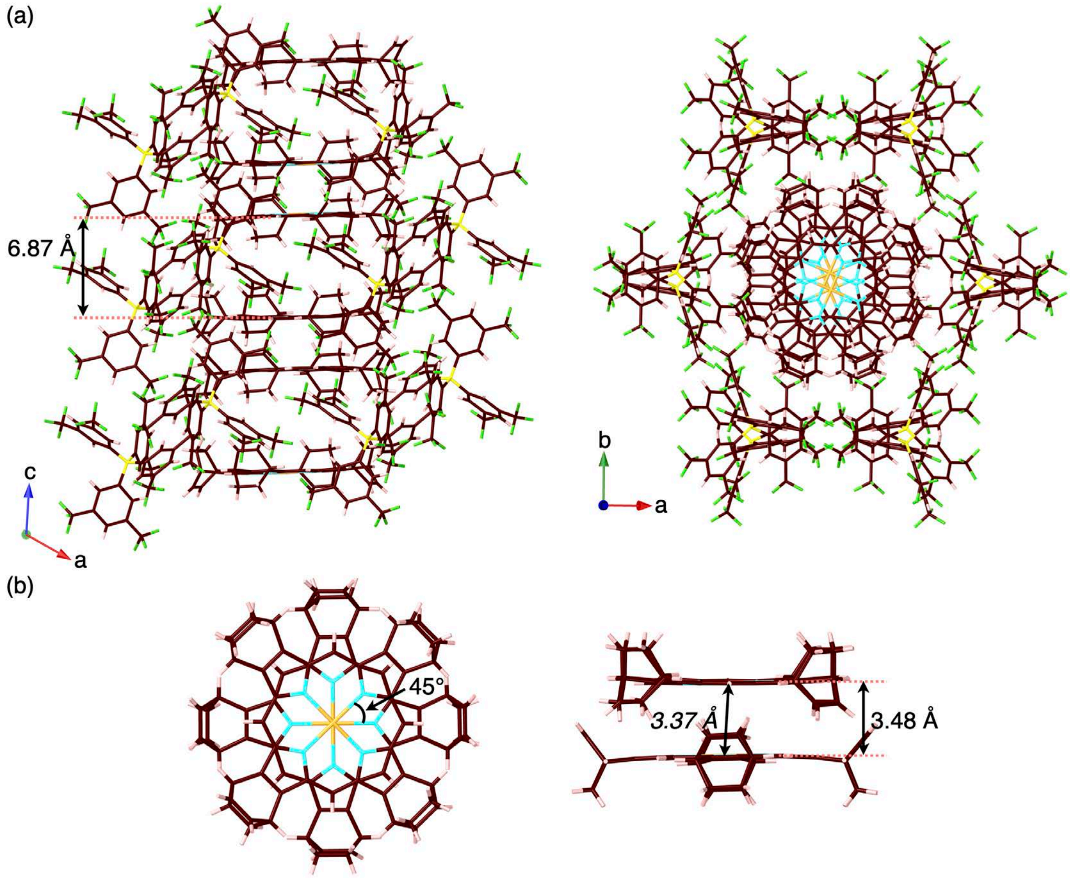

Fig. S19 (a) Top and side views of packing diagrams of **2au**$^{+}$-BArF^{-} and (b) the stacked dimer structure as top and side views. In the stacked dimer, face-to-face (between two mean planes comprising core 25 atoms for each) and Au···Au (italic) distances are 3.48 and 3.37 Å, respectively, and rotation angle is nearly 45°. Mean-plane deviation for core 25 atoms of **2au**$^{+}$ is 0.036 Å. Solvent molecules are omitted for clarity. Atom color code: brown, pink, yellow, blue, green, and orange refer to carbon, hydrogen, boron, nitrogen, fluorine, and gold, respectively.

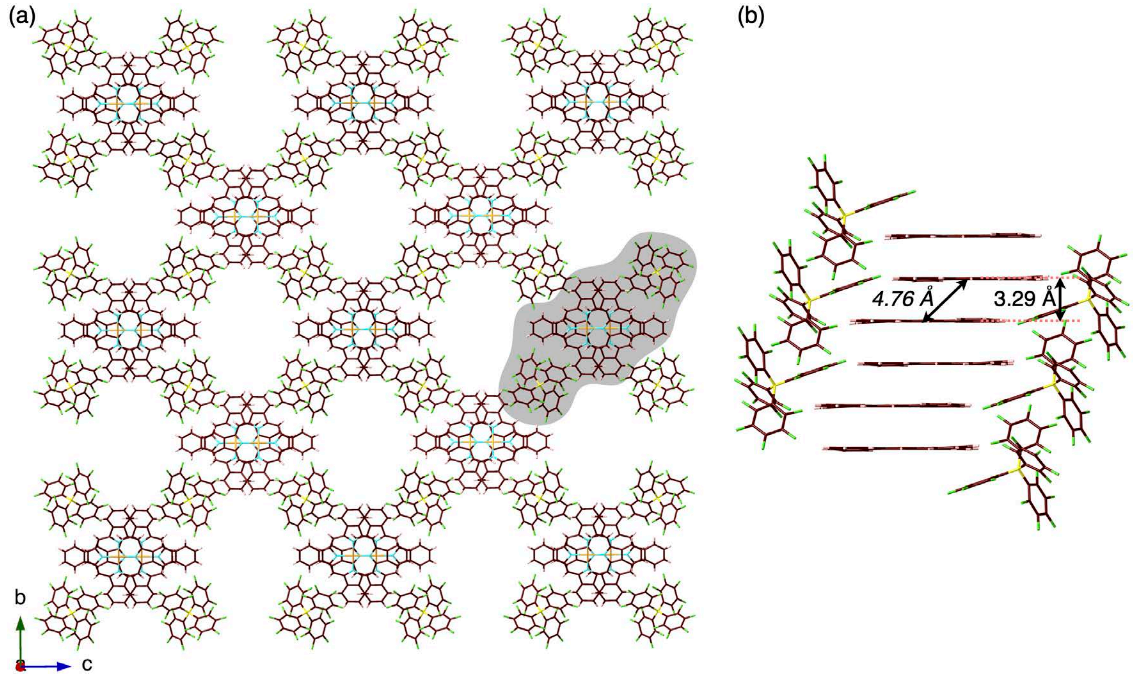

Fig. S20 (a) Packing diagram of **1au**$^+$-FABA$^-$ through *a*-axis and (b) side view of the stacked columnar structure of **1au**$^+$-FABA$^-$ at the gray part in (a). In (b), face-to-face (between two mean planes comprising core 25 atoms for each) and Au···Au (italic) distances are 3.29 and 4.76 Å, respectively. Mean-plane deviation for core 25 atoms of **1au**$^+$ is 0.016 Å. Atom color code: brown, pink, yellow, blue, green, and orange refer to carbon, hydrogen, boron, nitrogen, fluorine, and gold, respectively. The orientation of the unit cell *a*-axis for the long axis of the crystal were determined by the indexing crystal diffraction patterns (Fig. S23).

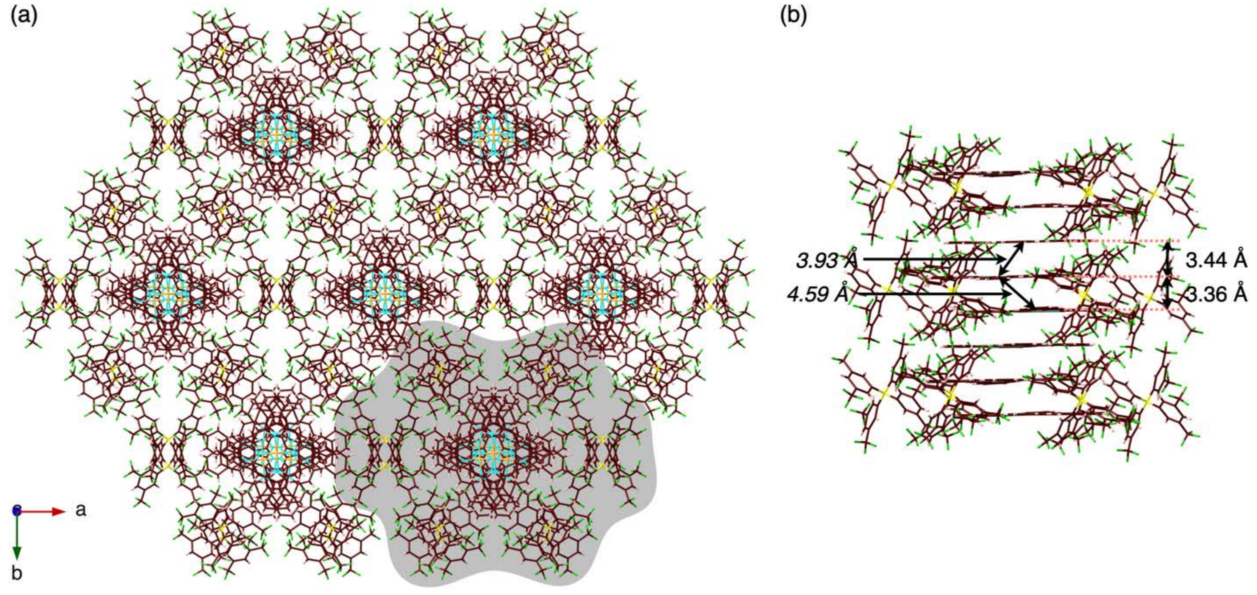

Fig. S21 (a) Packing diagram of **1au**$^+$-BArF$^-$ through *c*-axis and (b) side view of the stacked columnar structure of **1au**$^+$-BArF$^-$ at the gray part in (a). In (b), face-to-face (between two mean planes comprising core 25 atoms for each) and Au···Au (italic) distances are 3.44/3.93 and 3.36/4.59 Å, respectively. Mean-plane deviations for core 25 atoms of **1au**$^+$ are 0.031 and 0.011 Å. Solvent molecules are omitted for clarity. Atom color code: brown, pink, yellow, blue, green, and orange refer to carbon, hydrogen, boron, nitrogen, fluorine, and gold, respectively. The orientation of the unit cell *c*-axis for the long axis of the crystal were determined by the indexing crystal diffraction patterns (Fig. S23).

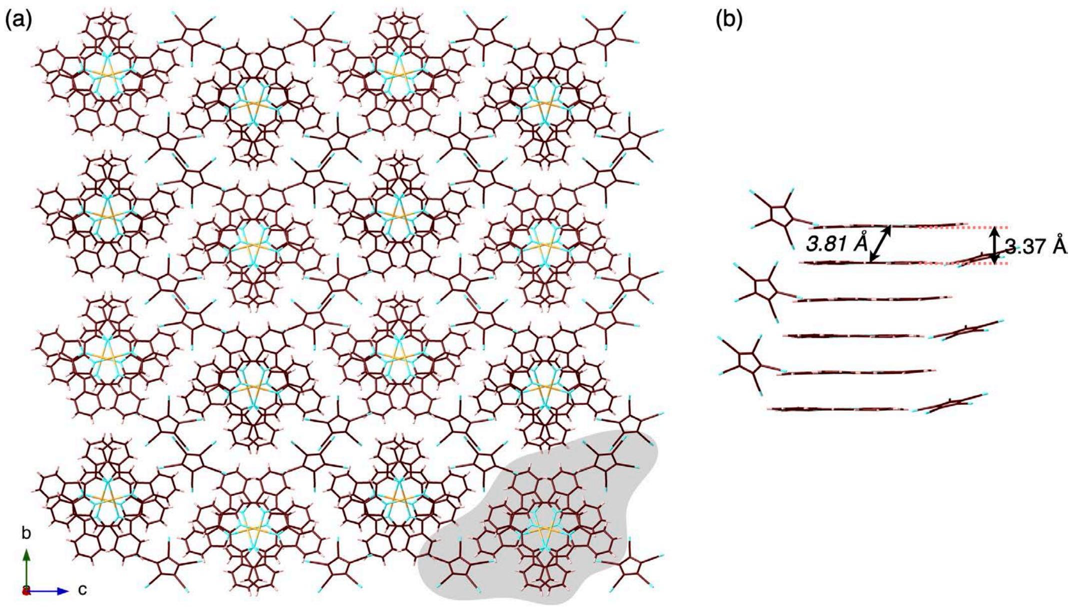

Fig. S22 (a) Packing diagram of **1au**$^{+}$-PCCp^{-} through *a*-axis and (b) side view of the stacked columnar structure of **1au**$^{+}$-PCCp^{-} at the gray part in (a). In (b), the face-to-face (between two mean planes comprising core 25 atoms for each) and Au···Au (italic) distances are 3.37 and 3.81 Å, respectively. Mean-plane deviation for core 25 atoms of **1au**$^{+}$ is 0.023 Å. Atom color code: brown, pink, blue, and orange refer to carbon, hydrogen, nitrogen, and gold, respectively. The orientation of the unit cell *a*-axis for the long axis of the crystal were determined by the indexing crystal diffraction patterns (Fig. S23).

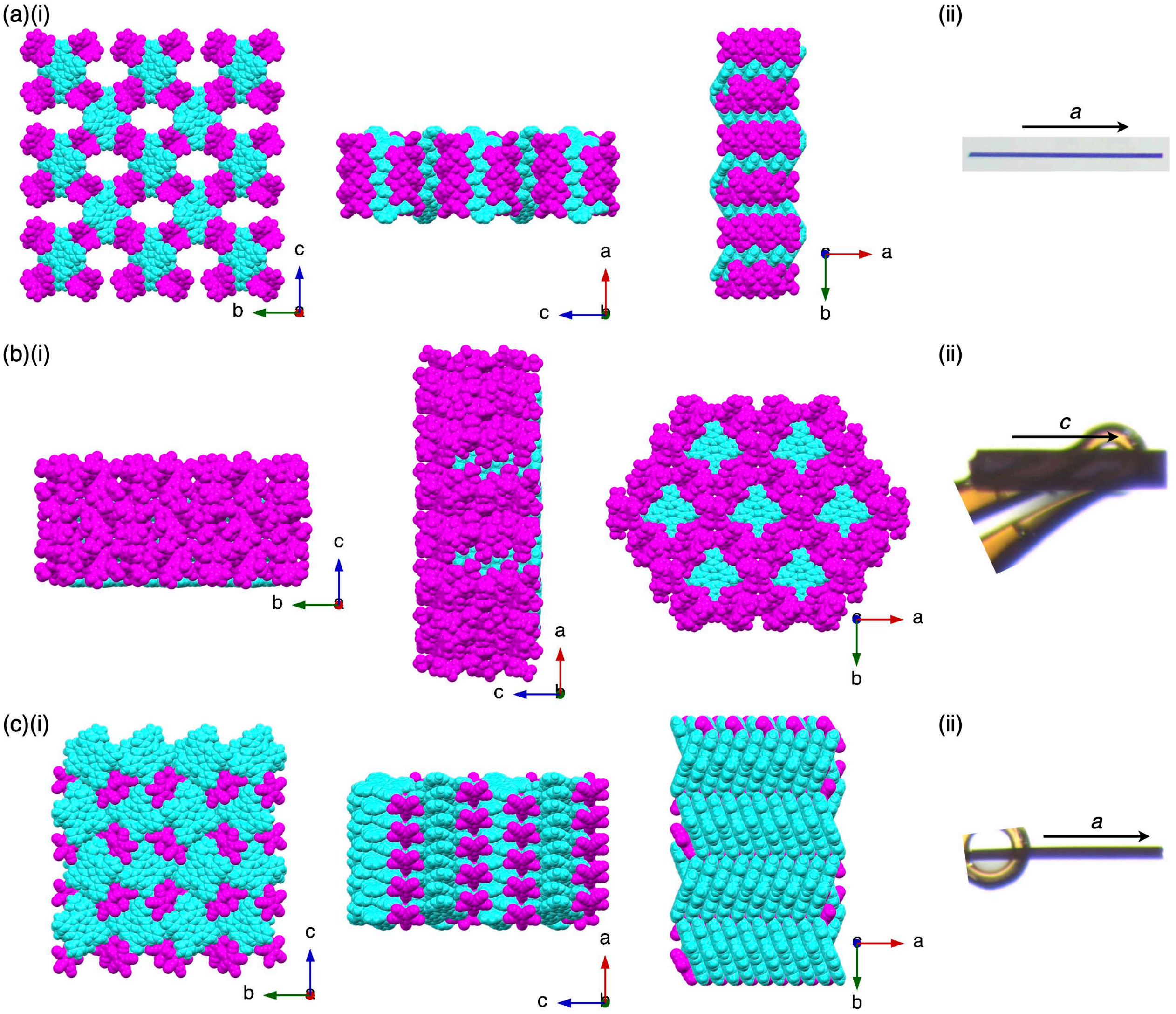

Fig. S23 Single-crystal X-ray structures of (a) **1au**$^+$-FABA$^-$, (b) **1au**$^+$-BArF$^-$, and (c) **1au**$^+$-PCCp$^-$: (i) packing diagram through *a*, *b*, and *c* axes (cyan and magenta represent cation and anion parts, respectively) and (ii) photographs of the single crystals with the axes for long axes of the crystals. The orientations of the unit cell axes for the long axes of the crystal, determined by the indexing crystal diffraction patterns, correspond to *a* axis in (a,c) and *c* axis in (b).

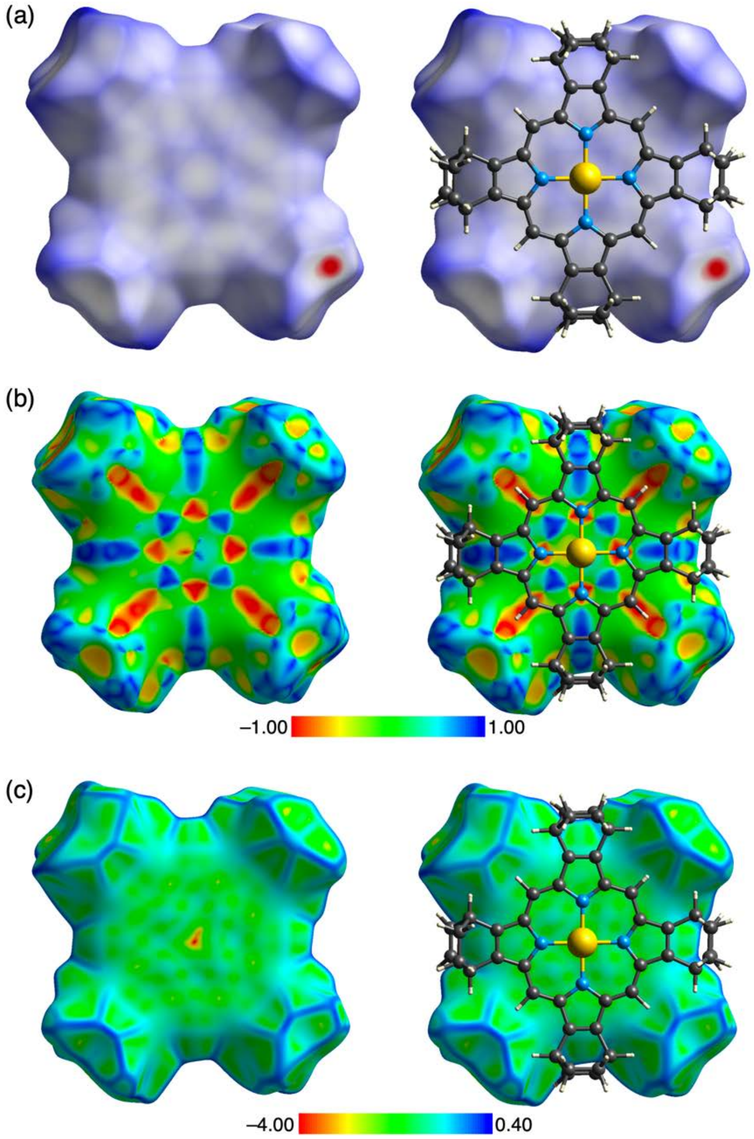

Fig. S24 Hirshfeld surface analysis[S7,8] of **2au**$^{+}$ in the crystal structure of **2au**$^{+}$-FABA^{-} mapped over (a) *d*-norm, (b) shape-index property, and (c) curvedness property: only surface (left) and surface with a ball-and-stick model of the neighboring **2au**$^{+}$ (right). The *d*-norm is normalized distance contact. Red regions indicate shorter contacts (with negative d_{norm} value), white regions indicate contacts around or close to (d_{norm} equal zero), while blue regions indicate longer contacts than the sum of the van der Waals radii (positive d_{norm}). Shape index is a qualitative measure of shape and is sensitive to subtle changes in surface shape, particularly in a flat region by differing by sign represent complementary bumps (blue) and hollows (red), whereas curvedness is a function of the root-mean-square curvature of the surface, and maps of curvedness typically show large regions of green (relatively flat) separated by dark blue edges (large positive curvature). Atom color code: gray, white, blue, and yellow refer to carbon, hydrogen, nitrogen, and gold, respectively.

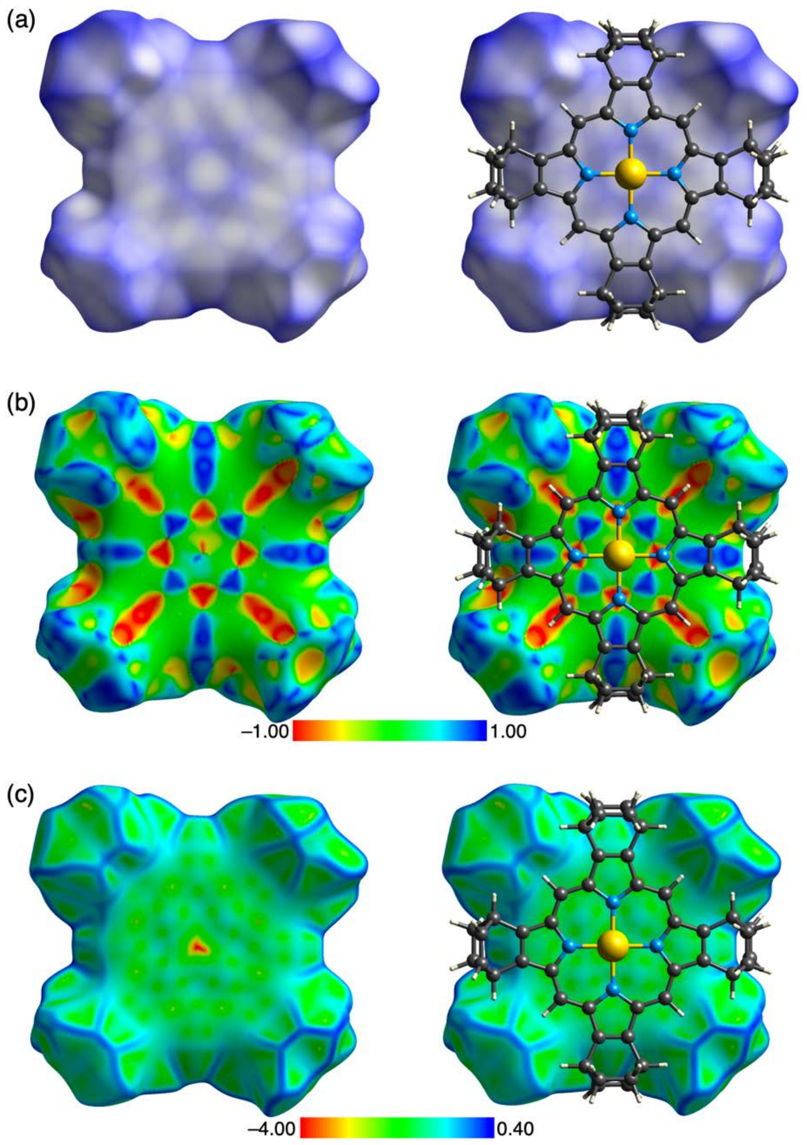

Fig. S25 Hirshfeld surface analysis[S7,8] of **2au**$^{+}$ in the crystal structure of **2au**$^{+}$-BArF^{-} mapped over (a) *d*-norm, (b) shape-index property, and (c) curvedness property: only surface (left) and surface with a ball-and-stick model of the neighboring **2au**$^{+}$ (right) (see the caption of Fig. S24 for the description of d_{norm}, shape-index, and curvedness properties). Atom color code: gray, white, blue, and yellow refer to carbon, hydrogen, nitrogen, and gold, respectively.

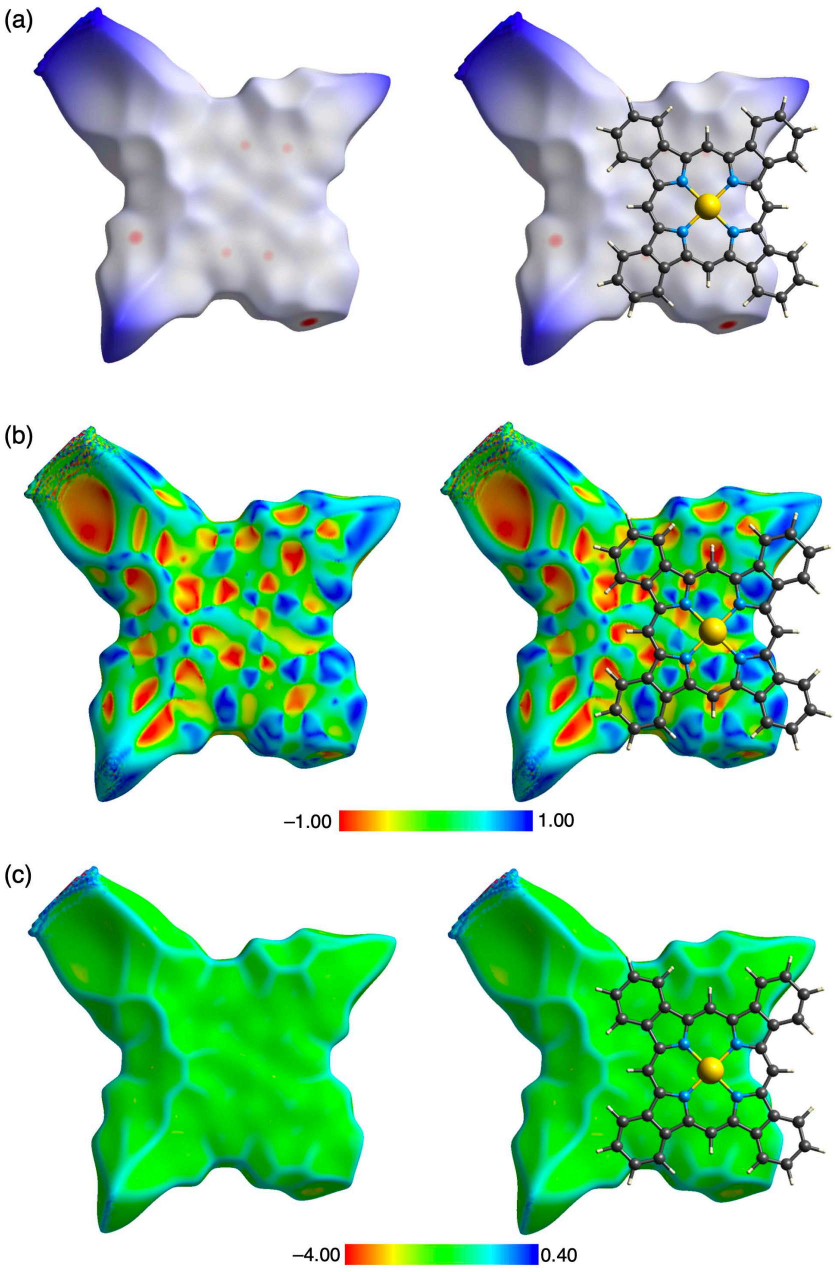

Fig. S26 Hirshfeld surface analysis[S7,8] of **1au**$^+$ in the crystal structure of **1au**$^+$-FABA$^-$ mapped over (a) *d*-norm, (b) shape-index property, and (c) curvedness property: only surface (left) and surface with a ball-and-stick model of the neighboring **1au**$^+$ (right) (see the caption of Fig. S24 for the description of d_{norm}, shape-index, and curvedness properties). Atom color code: gray, white, blue, and yellow refer to carbon, hydrogen, nitrogen, and gold, respectively.

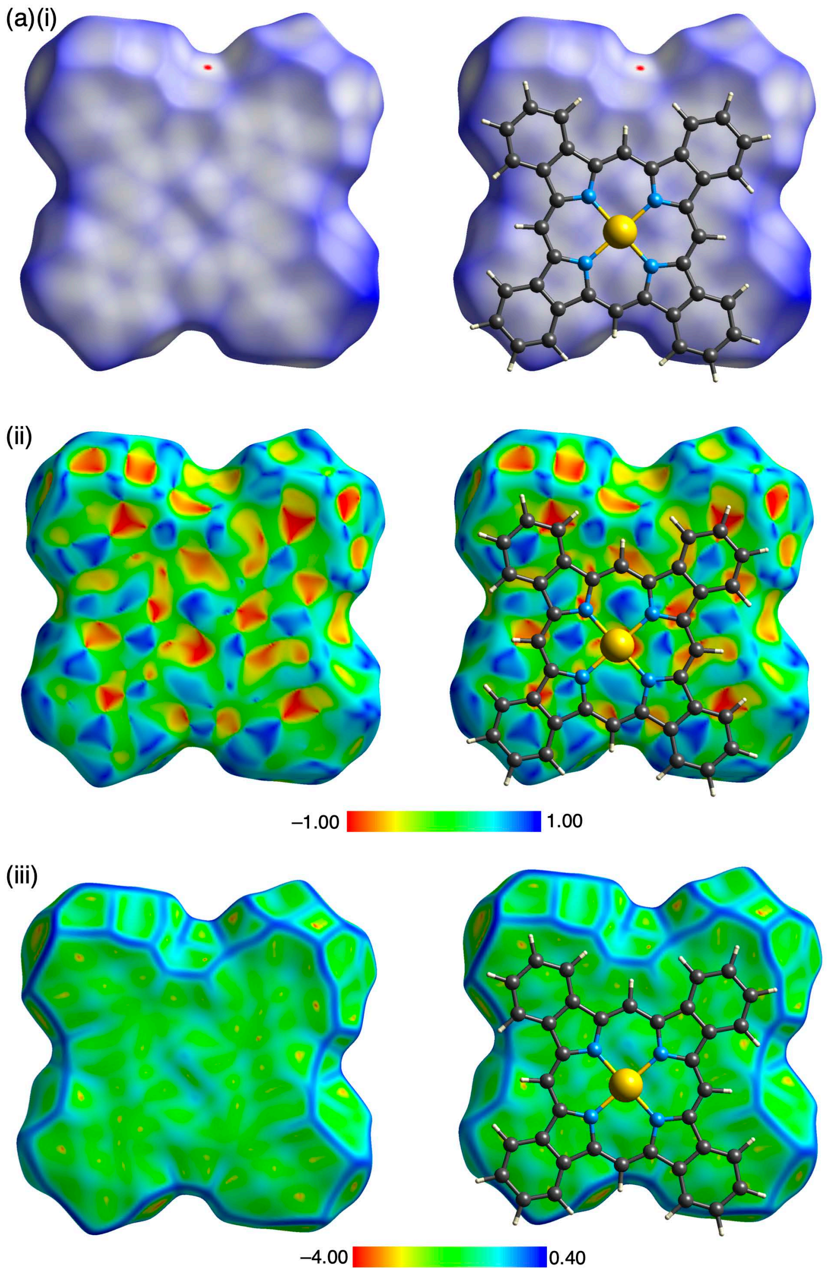

Fig. S27 Hirshfeld surface analysis[S7,8] of **1au**$^+$ in the crystal structure of **1au**$^+$-BArF$^-$ (two types of stacked dimer (a) and (b)) mapped over (i) *d*-norm, (ii) shape-index property, and (iii) curvedness property: only surface (left) and surface with a ball-and-stick model (right) of the neighboring **1au**$^+$ (see the caption of Fig. S24 for the description of d_{norm}, shape-index, and curvedness properties). Atom color code: gray, white, blue, and yellow refer to carbon, hydrogen, nitrogen, and gold, respectively.

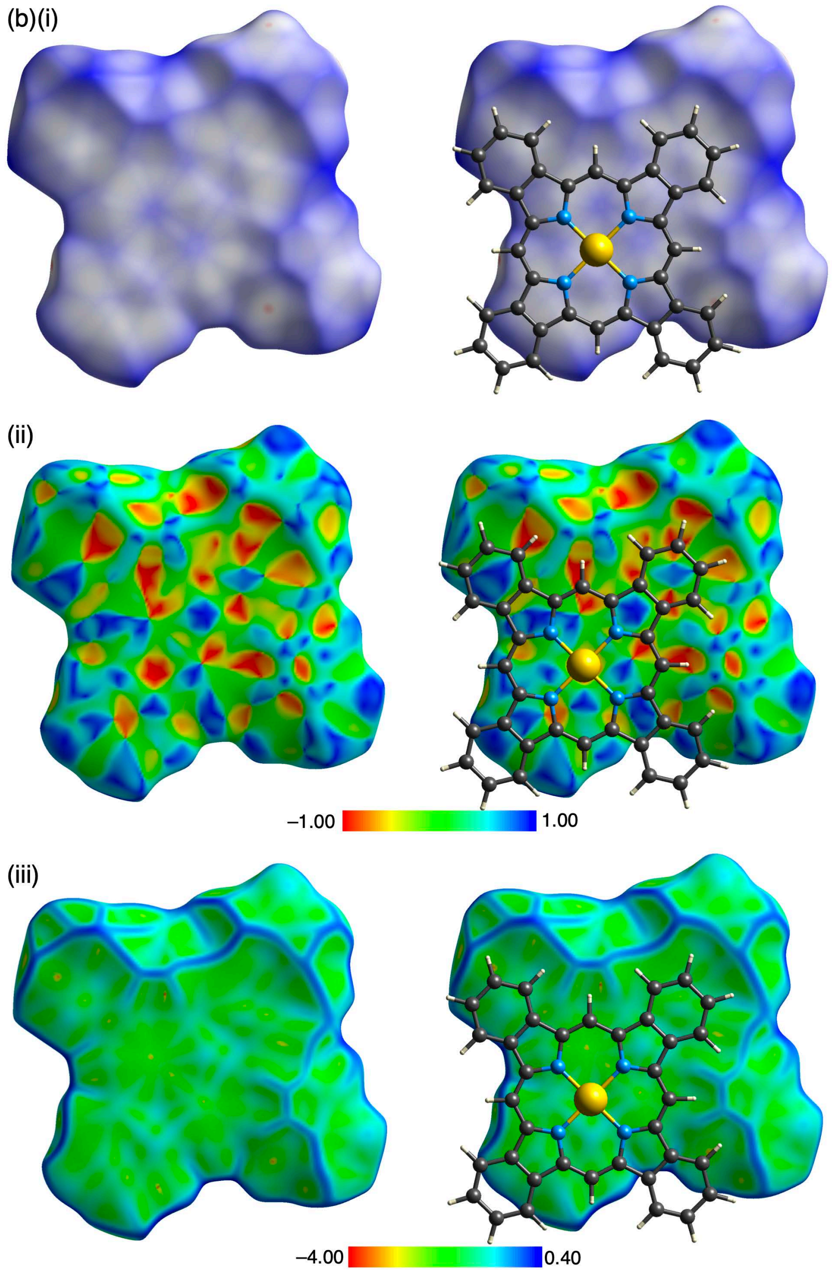

Fig. S27 (Continued)

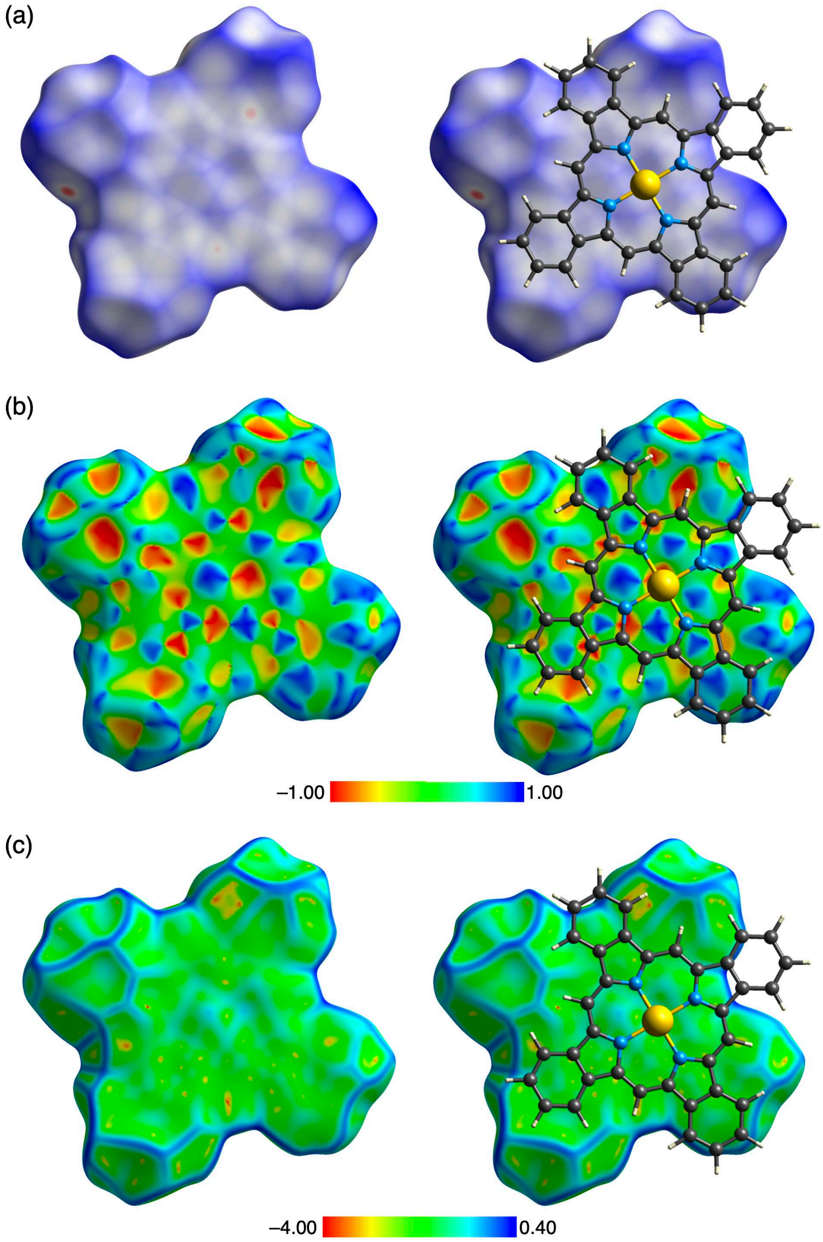

Fig. S28 Hirshfeld surface analysis[S7,8] of **1au**$^+$ in the crystal structure of **1au**$^+$-PCCp$^-$ mapped over (a) *d*-norm, (b) shape-index property, and (c) curvedness property: only surface (left) and surface with a ball-and-stick model (right) of the neighboring **1au**$^+$ (see the caption of Fig. S24 for the description of d_{norm}, shape-index, and curvedness properties). Atom color code: gray, white, blue, and yellow refer to carbon, hydrogen, nitrogen, and gold, respectively.

[S4] (a) N. Yasuda, H. Murayama, Y. Fukuyama, J. E. Kim, S. Kimura, K. Toriumi, Y. Tanaka, Y. Moritomo, Y. Kuroiwa, K. Kato, H. Tanaka and M. Takata, *J. Synchrotron Rad.*, 2009, **16**, 352–357; (b) N. Yasuda, Y. Fukuyama, K. Toriumi, S. Kimura and M. Takata, *AIP Conf. Proc.*, 2010, **1234**, 147–150; (c) K. Sugimoto, H. Ohsumi, S. Aoyagi, E. Nishibori, C. Moriyoshi, Y. Kuroiwa, H. Sawa and M. Takata, *AIP Conf. Proc.*, 2010, **1234**, 887–890.

[S5] G. M. Sheldrick, *Acta Crystallogr. Sect. A*, 2008, **64**, 112–122.

[S6] (a) K. Wakita, *Yadokari-XG*, *Software for Crystal Structure Analyses*, 2001; (b) C. Kabuto, S. Akine, T. Nemoto and E. Kwon, *J. Cryst. Soc. Jpn.*, 2009, **51**, 218–224.

[S7] P. R. Spackman, M. J. Turner, J. J. McKinnon, S. K. Wolff, D. J. Grimwood, D. Jayatilaka and M. A. Spackman, *J. Appl. Cryst.*, 2021, **54**, 1006–1011.

[S8] (a) M. A. Spackman and D. Jayatilaka, *CrystEngComm*, 2009, **11**, 19–32; (b) J. J. McKinnon, M. A. Spackman and A. S. Mitchell, *Acta Crystallogr. Sect. B*, 2004, **60**, 627–668.

3. Theoretical studies

DFT calculations. DFT calculations for the geometrical optimizations were carried out by using the *Gaussian 16* program.[S9]

MD simulation. All-atom molecular dynamics (MD) simulation was performed with the MD program *GROMACS 2016.6*, which is a free program used for fast and large-scale simulations (Fig. 9d, S66). For calculating the intra- and intermolecular interactions of the molecules, the second generation of generalized Amber force field[S10] (GAFF2) parameters were used for accurate calculations applicable to various systems.[S11,12] Note that the force field parameters for B in $FABA^{-}$ remains unsupported in the framework of the GAFF2 manner, so that the lacking parameters were optimized in this study. The van der Waals parameters were fixed to 0.35814 nm and 0.3975 kJ/mol for σ and ε, respectively, cited from the computational study of Wang et al.,[S13] and the bonding harmonic interaction around the born atom were determined by the DFT calculation of MP2/6-311+G(d,p) level for tetraphenylborate anion.[S14] The force constants were optimized to reproduce the MP2 frequency with a scaling factor of 0.9496 by Scott and Rado.[S15] For **1au**$^{+}$, the van der Waals interaction parameters of Au were set to 0.2934 nm and 0.1633 kJ/mol for σ and ε, respectively.[S16] The force field parameters of the bond, angle, and dihedral potentials with respect to Au were applied those from the universal force field[S16] parameters. The partial atomic charges of **1au**$^{+}$ and $FABA^{-}$ were obtained by applying the restrained electrostatic potential (RESP) method,[S17] based on single point DFT calculations based on the *Gaussian 16* program[S9] using the LanL2DZ basis set with the associated effective core potential for Au and the B3LYP/6-31++G(d,p) level for the other atoms. The simulated system consisted of 2592 molecules: half **1au**$^{+}$ and half $FABA^{-}$. For the initial structures of the hexagonal columnar structure, 36 columns were positioned in the hexagonal box of 12.54 nm × 10.86 nm × 12.24 nm. Pre-equilibration runs were performed for 5 ns at 298 K and 1 bar. The temperature and pressure were kept using the Berendsen thermostat and barostat,[S18] respectively, with relaxation times of 0.2 and 2.0 ps. Following the pre-equilibration runs, the equilibration run was performed for 100 ns at 298 K and 1 bar. The Nosé-Hoover thermostat[S19] and Parrinello-Rahman barostat[S20] were used to maintain the temperature and pressure constant with relaxation times of 1.0 and 5.0 ps, respectively. The time step was set to 2 fs and all covalent bonds connected to hydrogen atoms were constrained by using the LINCS algorithm.[S21] The long-range electrostatic interactions were treated by the smooth particle mesh Ewald with a real-space cutoff of 1.2 nm and a Fourier spacing of 0.30 nm. A cutoff for the van der Waals interactions was set to 1.2 nm.

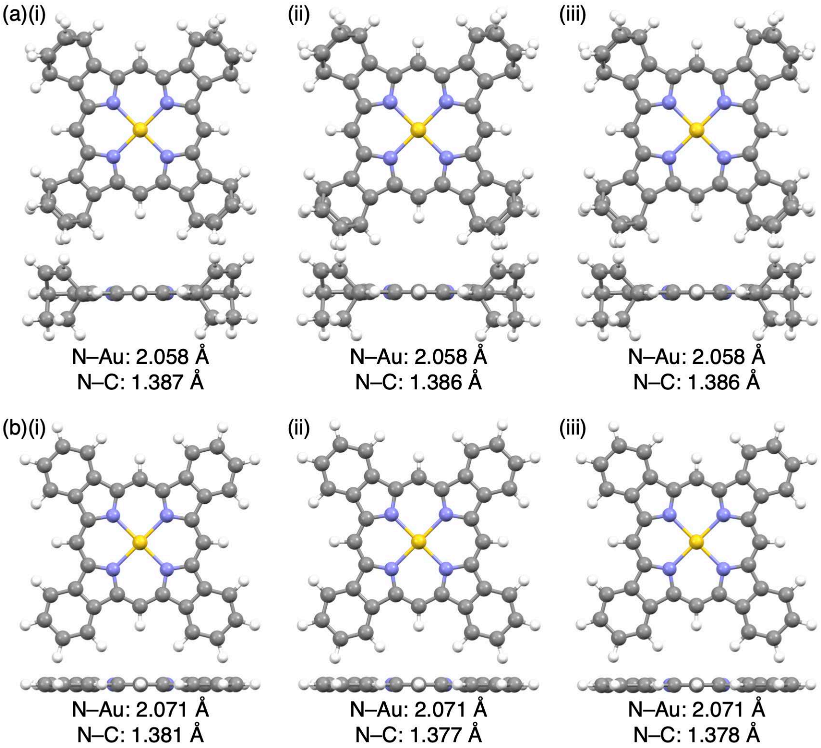

Fig. S29 Optimized structures (top and side views) of (a) **2au**$^+$ and (b) **1au**$^+$ at (i) B3LYP/6-31+G(d,p) with LanL2DZ for Au, (ii) PCM-B3LYP/6-31+G(d,p) with LanL2DZ for Au (DMSO), and (iii) PCM-B3LYP/6-31+G(d,p) with LanL2DZ for Au (CH_3CN). The mean-plane deviations for **2au**$^+$ and **1au**$^+$ (core 25 atoms) without/with PCM in (i–iii) are 0.01/0.02/0.01 and 0.00/0.00/0.00 Å, respectively. For the optimization of **2au**$^+$, all ethylene units are arranged at the same porphyrin plane side based on the crystal structure. Theoretical studies for Fig. S33–35,38–40,43–45,48–50 were examined with these optimized structures.

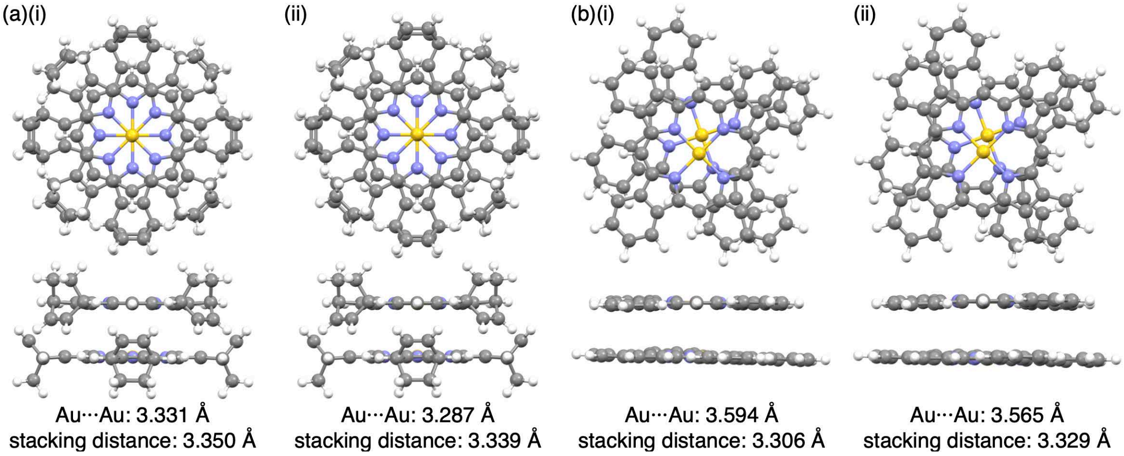

Fig. S30 Optimized structures (top and side views) of stacked dimers of (a) **2au**$^+$ and (b) **1au**$^+$ at (i) B3LYP-GD3BJ/6-31+G(d,p) with LanL2DZ for Au and (ii) PCM-B3LYP-GD3BJ/6-31+G(d,p) with LanL2DZ for Au (CH_3CN). The crystal structures of **2au**$^+$-FABA$^-$ and **1au**$^+$-FABA$^-$ were used for the initial structures of the optimization. Rotation angles in the stacked **2au**$^+$ dimers are nearly 45°. Theoretical studies for Fig. S36,41,46,51 were examined with these optimized structures.

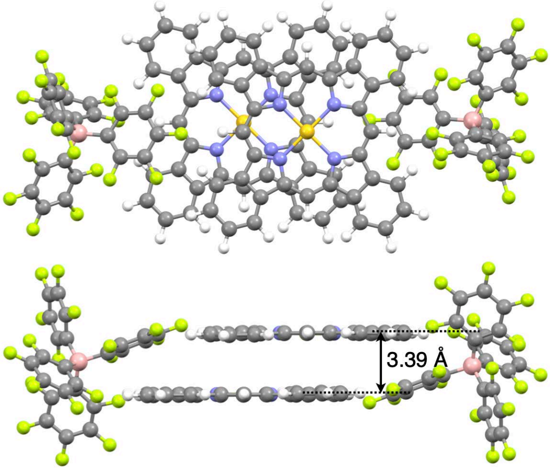

Fig. S31 Optimized structure (top and side views) of stacked dimer of **1au**$^+$-FABA$^-$ at B3LYP-GD3BJ/6-31+G(d,p) with LanL2DZ for Au based on the single-crystal structure.

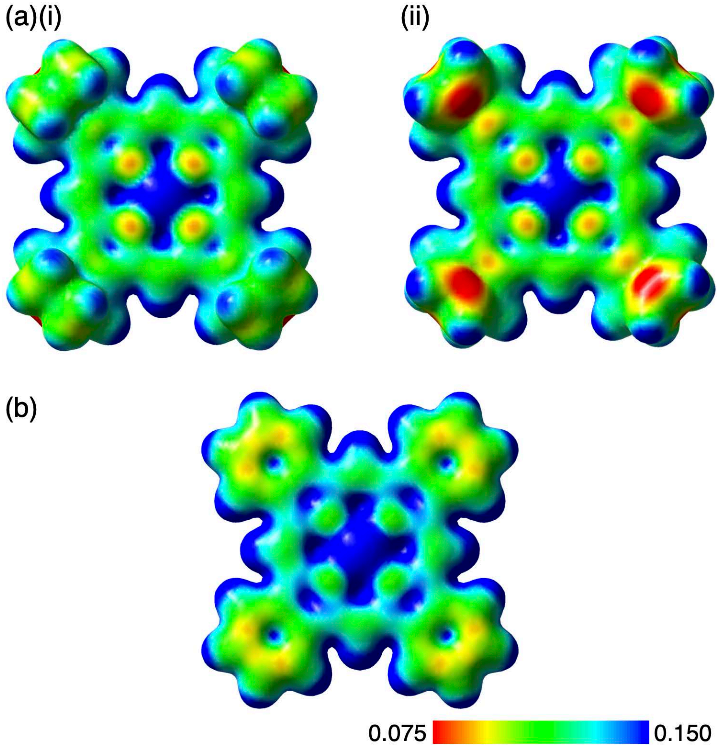

Fig. S32 Electrostatic potentials (ESP) of (a) **2au**$^+$ (views from (i) CH_2–CH_2 and (ii) CH=CH sides) and (b) **1au**$^+$ mapped onto the electron density isosurfaces (δ = 0.01) at B3LYP/6-31+G(d,p) with LanL2DZ for Au.

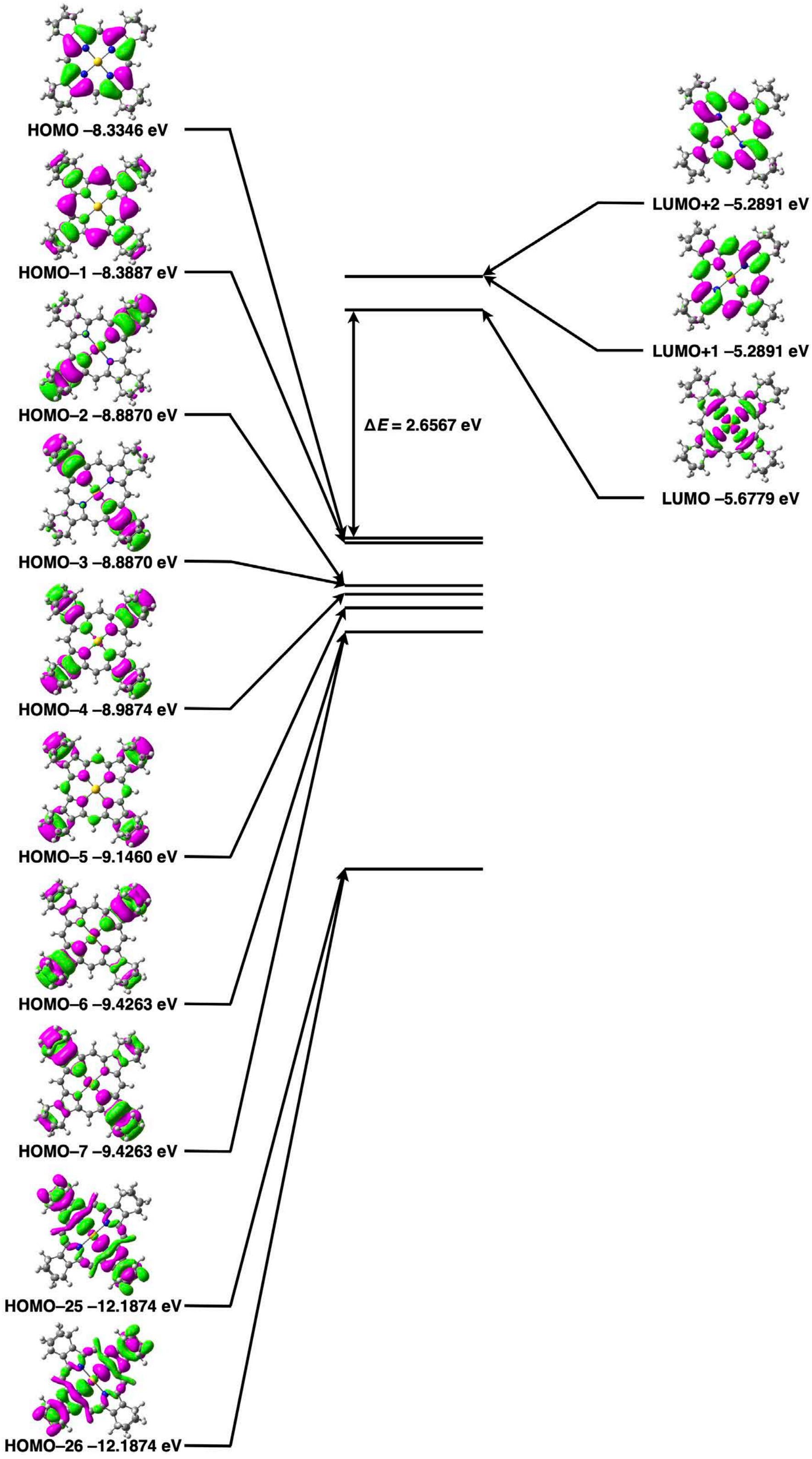

Fig. S33 Molecular orbitals (HOMO/LUMO) of **2au**$^+$ estimated at B3LYP/6-31+G(d,p) with LanL2DZ for Au based on the optimized structure (Fig. S29). Selected MOs are shown based on the TD-DFT data in Fig. S43.

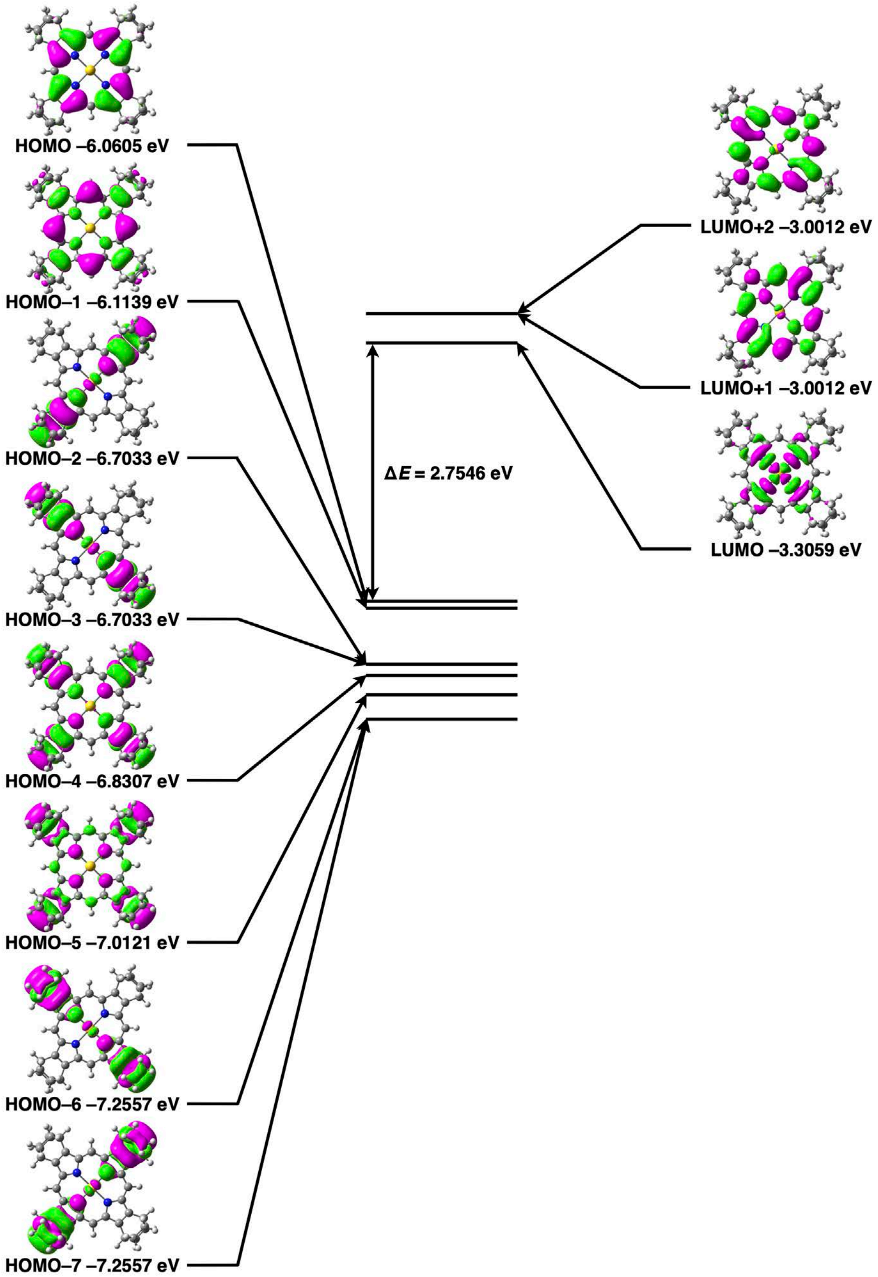

Fig. S34 Molecular orbitals (HOMO/LUMO) of **2au**$^+$ estimated at PCM-B3LYP/6-31+G(d,p) with LanL2DZ for Au (DMSO) based on the optimized structure (Fig. S29). Selected MOs are shown based on the TD-DFT data in Fig. S44.

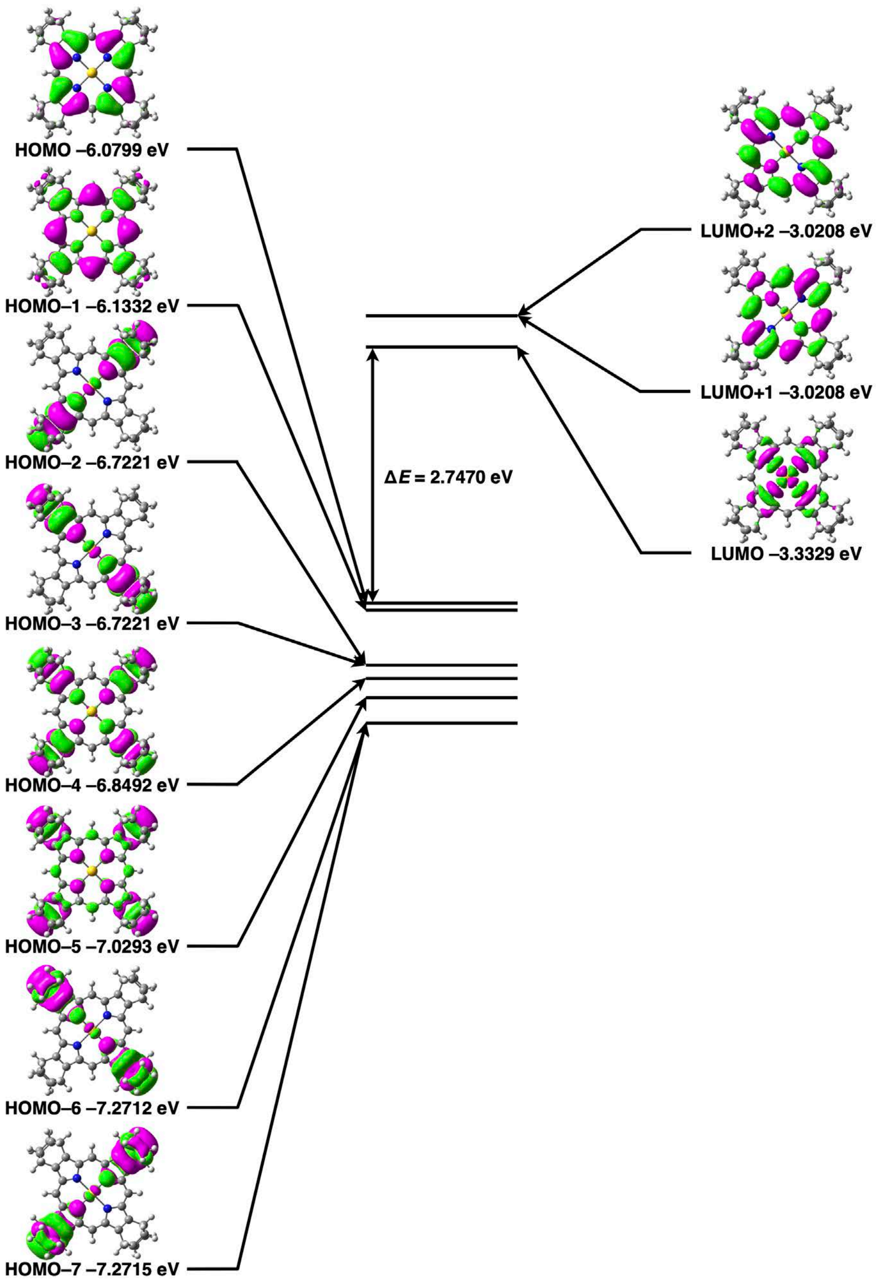

Fig. S35 Molecular orbitals (HOMO/LUMO) of **2au**$^+$ estimated at PCM-B3LYP/6-31+G(d,p) with LanL2DZ for Au (CH_3CN) based on the optimized structure (Fig. S29). Selected MOs are shown based on the TD-DFT data in Fig. S45.

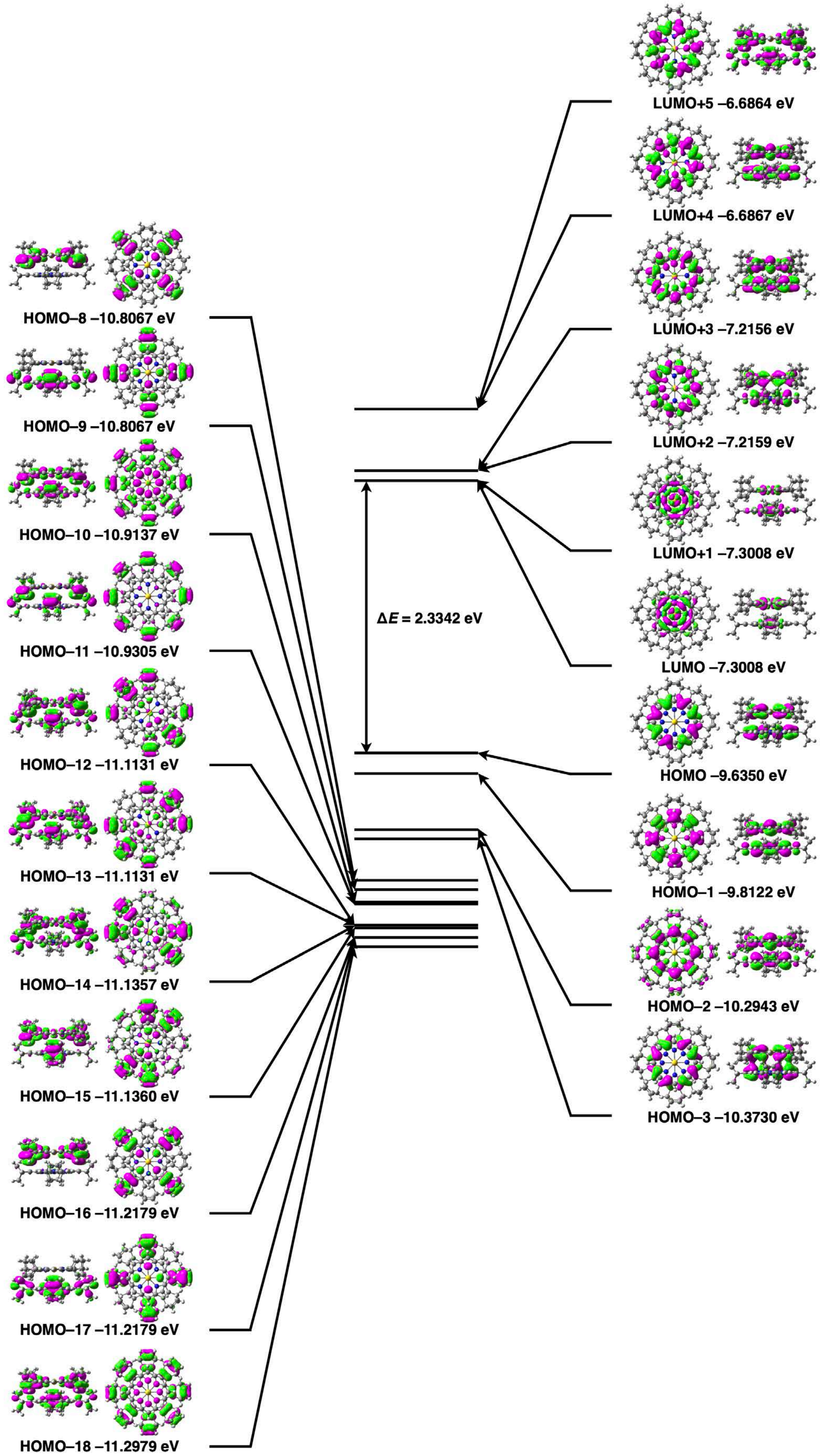

Fig. S36 Molecular orbitals (HOMO/LUMO) of **2au**$^+$ as a stacked dimer estimated at PCM-B3LYP-GD3BJ/6-31+G(d,p) with LanL2DZ for Au (CH_3CN) based on the optimized structure of stacked **2au**$^+$ dimer (Fig. S30). Selected MOs are shown based on the TD-DFT data in Fig. S46.

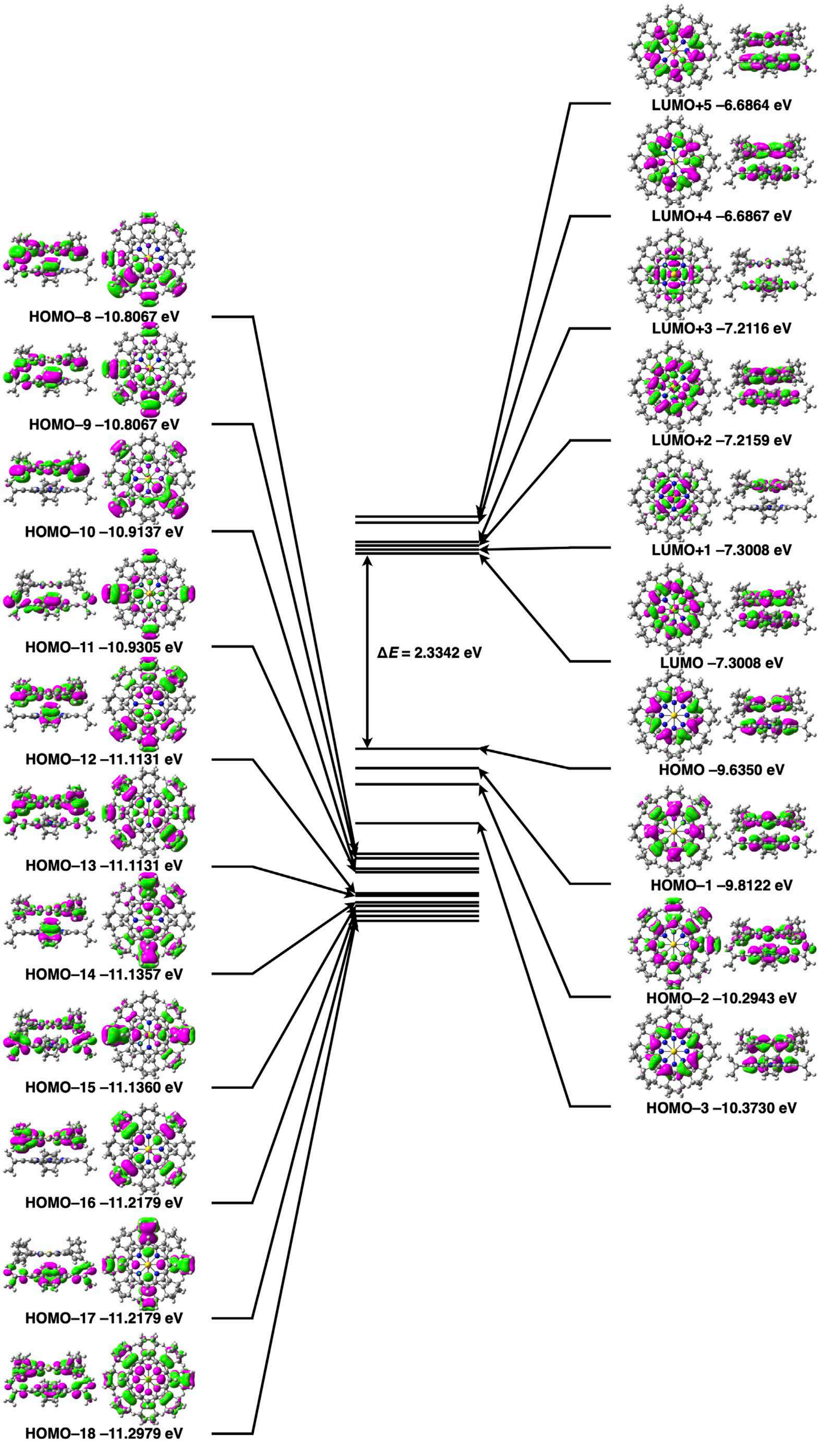

Fig. S37 Molecular orbitals (HOMO/LUMO) of **2au**$^+$ as a stacked dimer estimated at B3LYP-GD3BJ/6-31+G(d,p) with LanL2DZ for Au based on the stacked **2au**$^+$ dimer in the crystal structure of **2au**$^+$-FABA$^-$ (Fig. S18). Selected MOs are shown based on the TD-DFT data in Fig. S47.

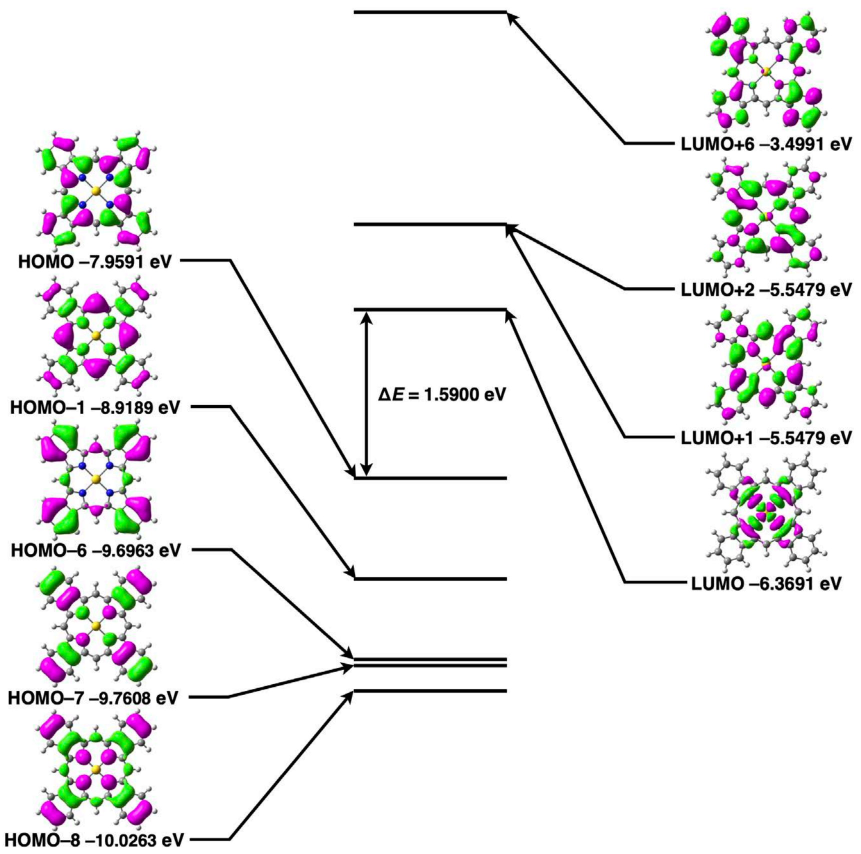

Fig. S38 Molecular orbitals (HOMO/LUMO) of **1au**$^+$ estimated at B3LYP/6-31+G(d,p) with LanL2DZ for Au based on the optimized structure (Fig. S29). Selected MOs are shown based on the TD-DFT data in Fig. S48.

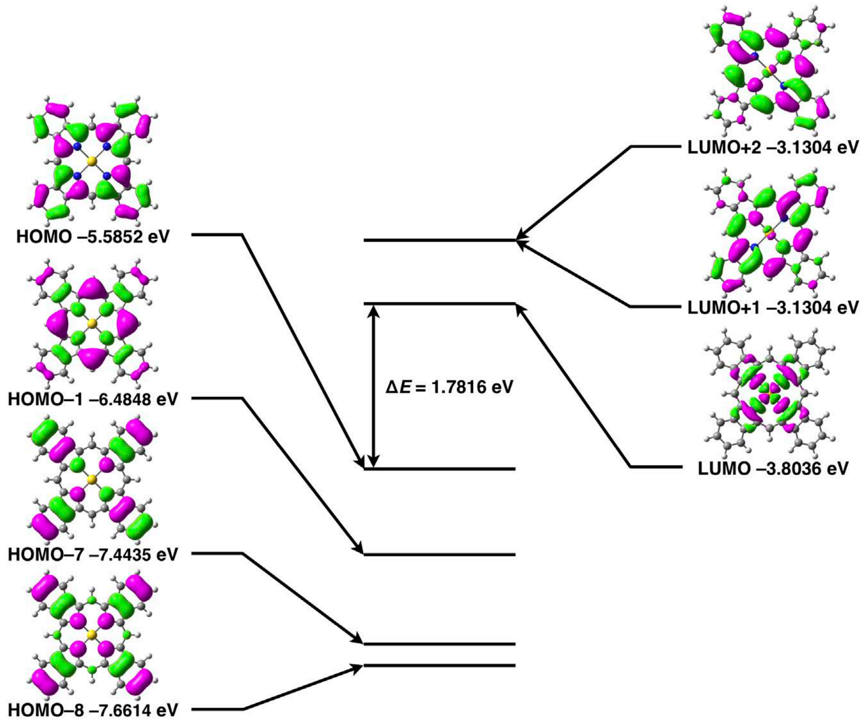

Fig. S39 Molecular orbitals (HOMO/LUMO) of **1au**$^+$ estimated at PCM-B3LYP/6-31+G(d,p) with LanL2DZ for Au (DMSO) based on the optimized structure (Fig. S29). Selected MOs are shown based on the TD-DFT data in Fig. S49.

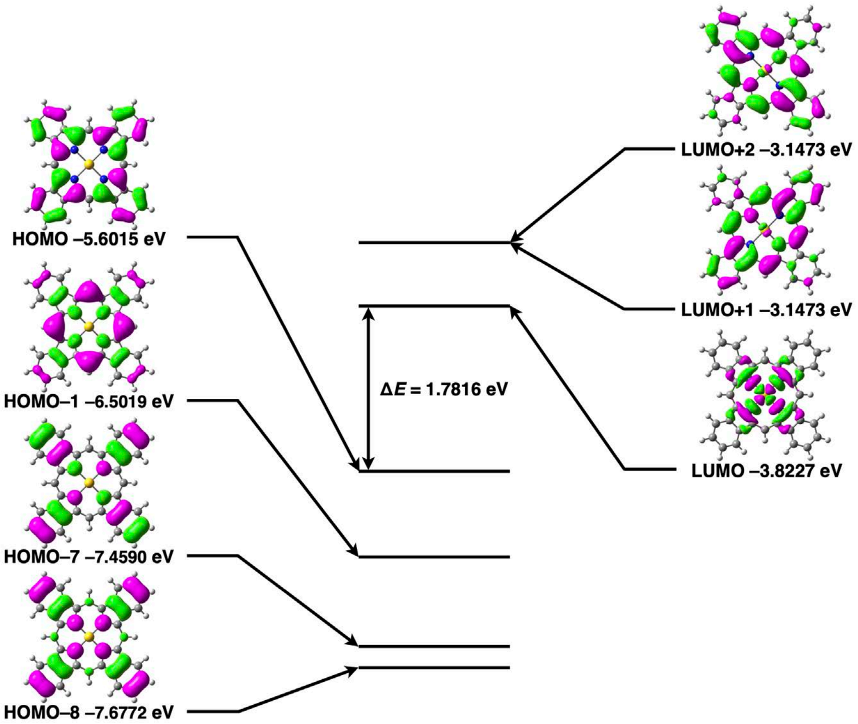

Fig. S40 Molecular orbitals (HOMO/LUMO) of **1au**$^{+}$ estimated at PCM-B3LYP/6-31+G(d,p) with LanL2DZ for Au (CH_3CN) based on the optimized structure (Fig. S29). Selected MOs are shown based on the TD-DFT data in Fig. S50.

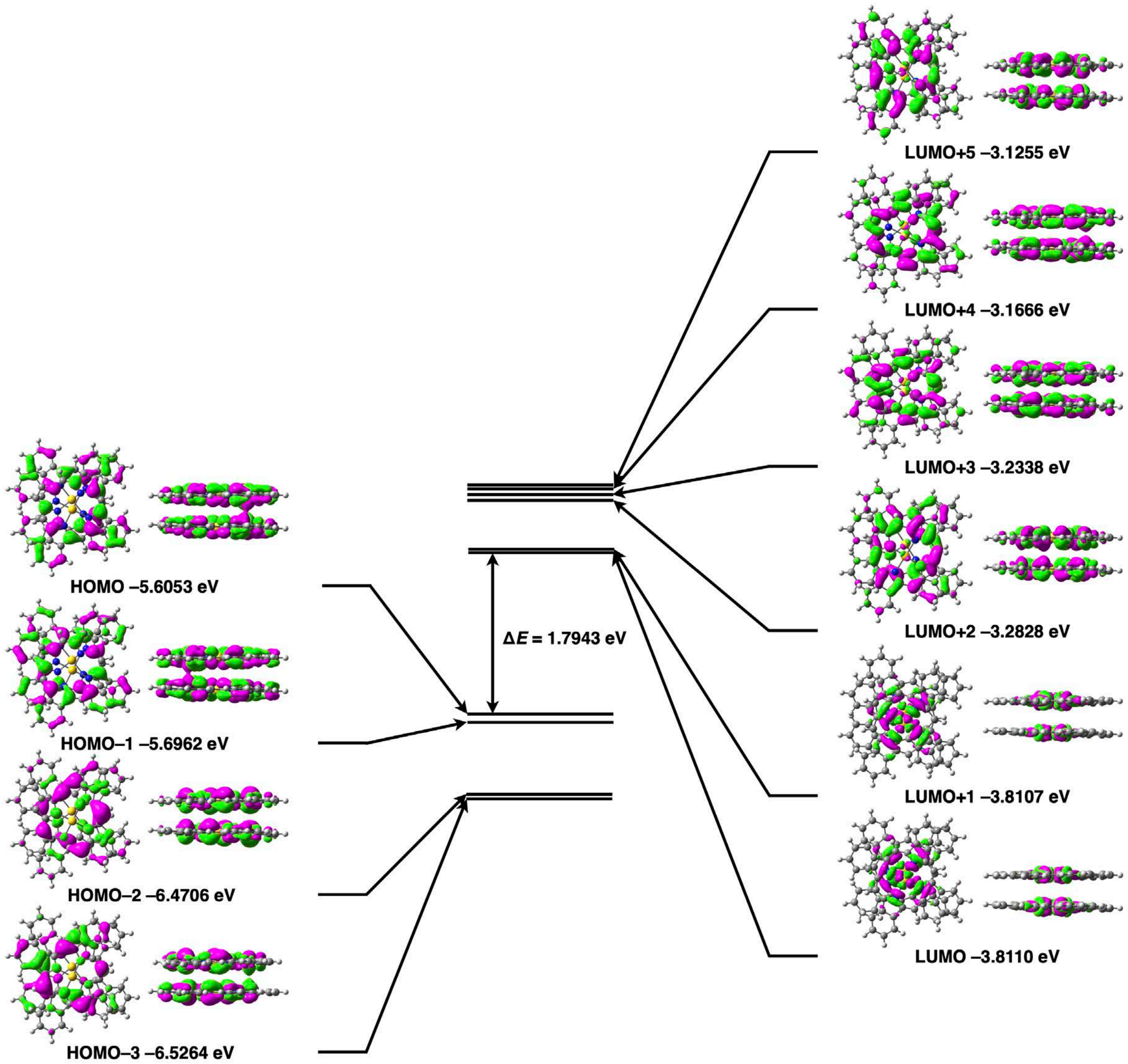

Fig. S41 Molecular orbitals (HOMO/LUMO) of **1au**$^+$ as a stacked dimer estimated at PCM-B3LYP-GD3BJ/6-31+G(d,p) with LanL2DZ for Au (CH_3CN) based on the optimized structure of stacked **1au**$^+$ dimer (Fig. S30). Selected MOs are shown based on the TD-DFT data in Fig. S51.

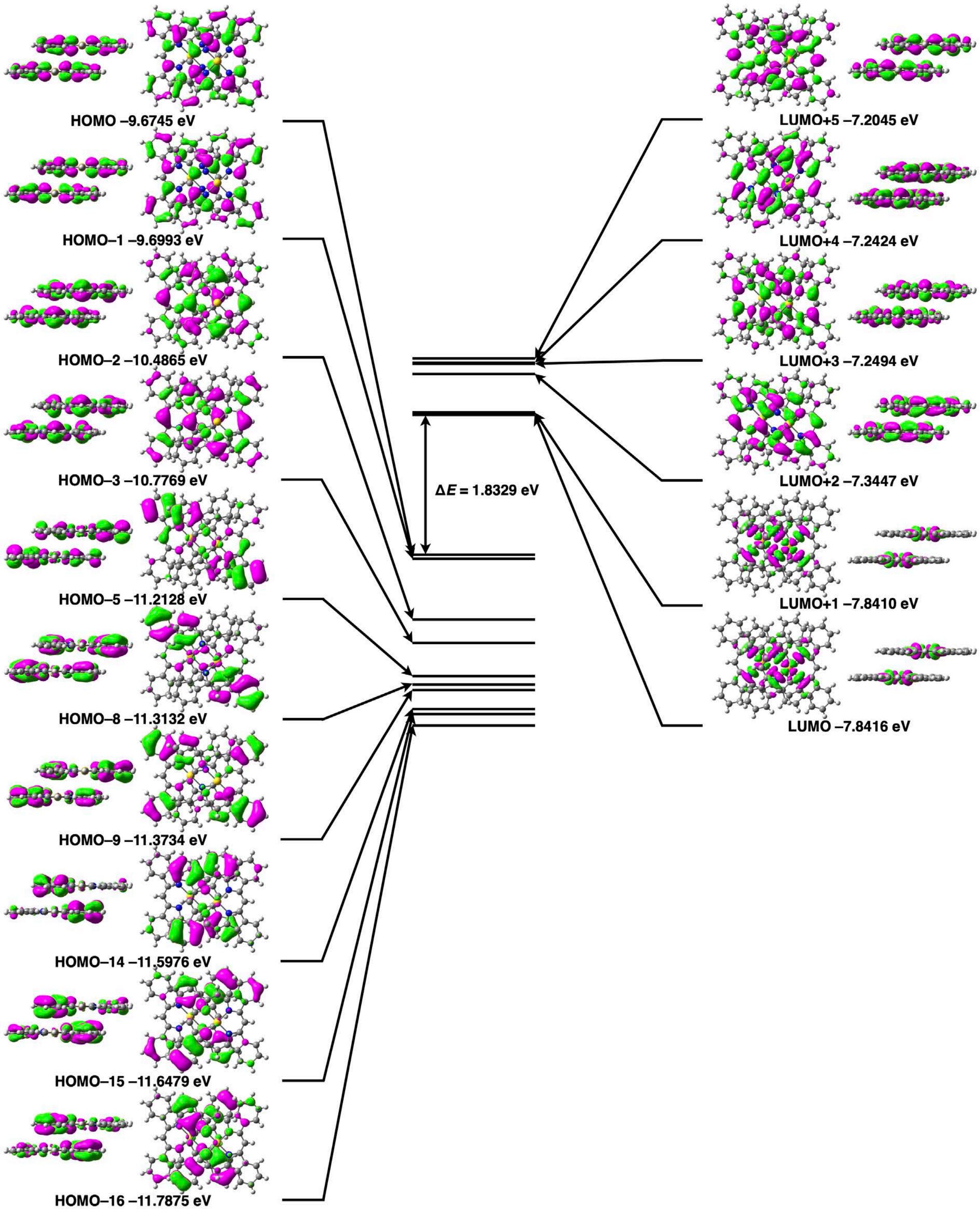

Fig. S42 Molecular orbitals (HOMO/LUMO) of **1au**$^+$ as a stacked dimer estimated at B3LYP-GD3BJ/6-31+G(d,p) with LanL2DZ for Au based on the stacked **1au**$^+$ dimer in the single-crystal structure of **1au**$^+$-FABA$^-$ (Fig. S20). Selected MOs are shown based on the TD-DFT data in Fig. S52.

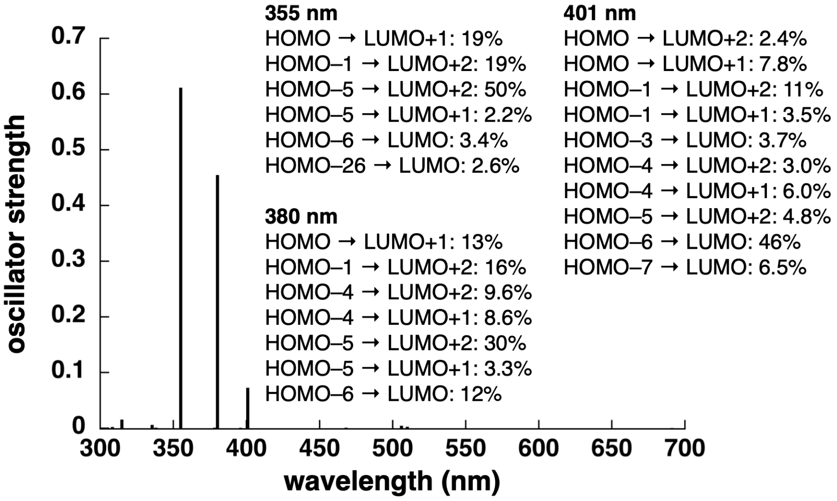

Fig. S43 TD-DFT-based UV/vis absorption stick spectrum of **2au**$^+$ with the transitions correlated with molecular orbitals (MOs) (Fig. S33) estimated at B3LYP/6-31+G(d,p) with LanL2DZ for Au based on the optimized structure (Fig. S29).

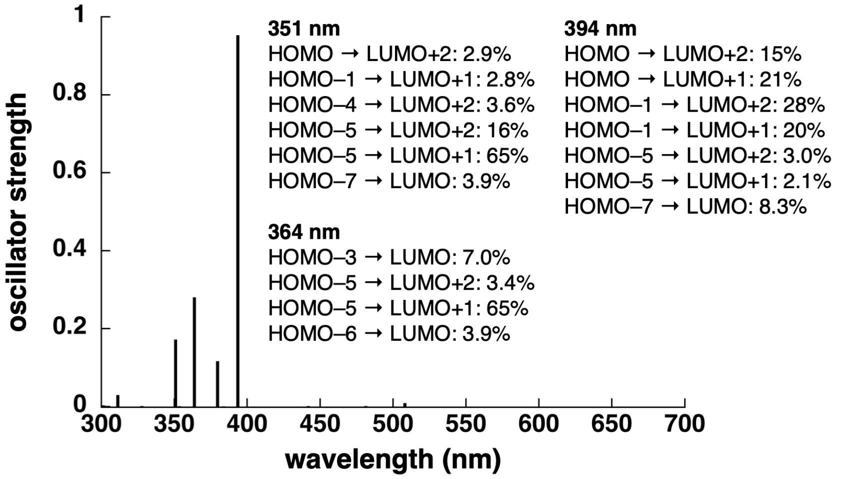

Fig. S44 TD-DFT-based UV/vis absorption stick spectrum of **2au**$^+$ with the transitions correlated with MOs (Fig. S34) estimated at PCM-B3LYP/6-31+G(d,p) with LanL2DZ for Au (DMSO) based on the optimized structure (Fig. S29).

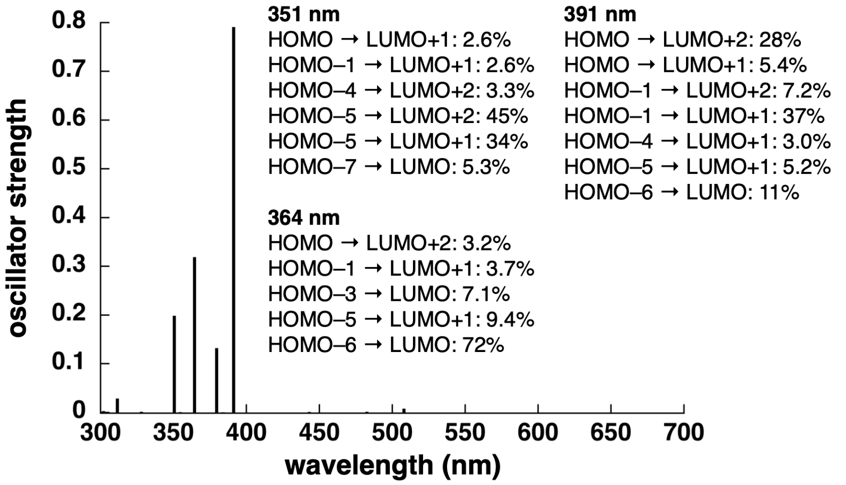

Fig. S45 TD-DFT-based UV/vis absorption stick spectrum of **2au**$^+$ with the transitions correlated with MOs (Fig. S35) estimated at PCM-B3LYP/6-31+G(d,p) with LanL2DZ for Au (CH_3CN) based on the optimized structure (Fig. S29).

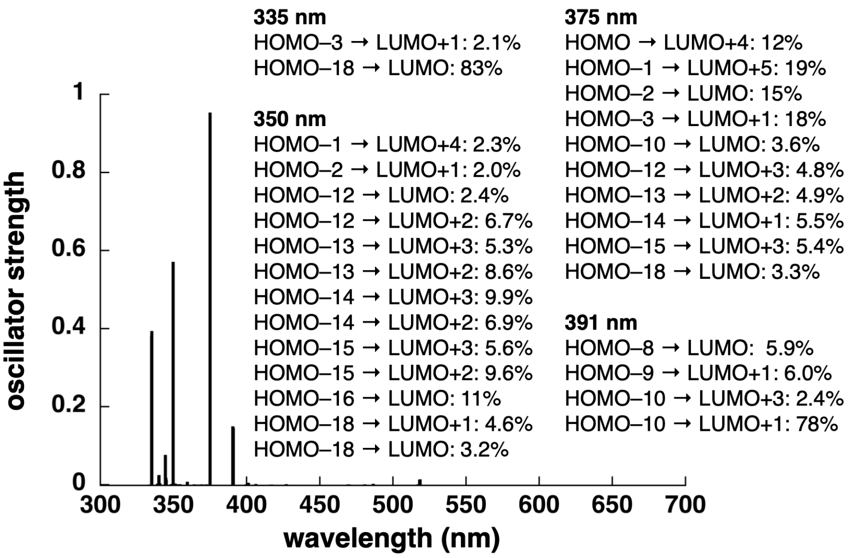

Fig. S46 TD-DFT-based UV/vis absorption stick spectrum of **2au**$^+$ as a stacked dimer with the transitions correlated with MOs (Fig. S36) estimated at PCM-B3LYP-GD3BJ/6-31+G(d,p) with LanL2DZ for Au (CH_3CN) based on the optimized structure for the stacked dimer in the single-crystal X-ray structure of **2au**$^+$-FABA$^-$ (Fig. S30).

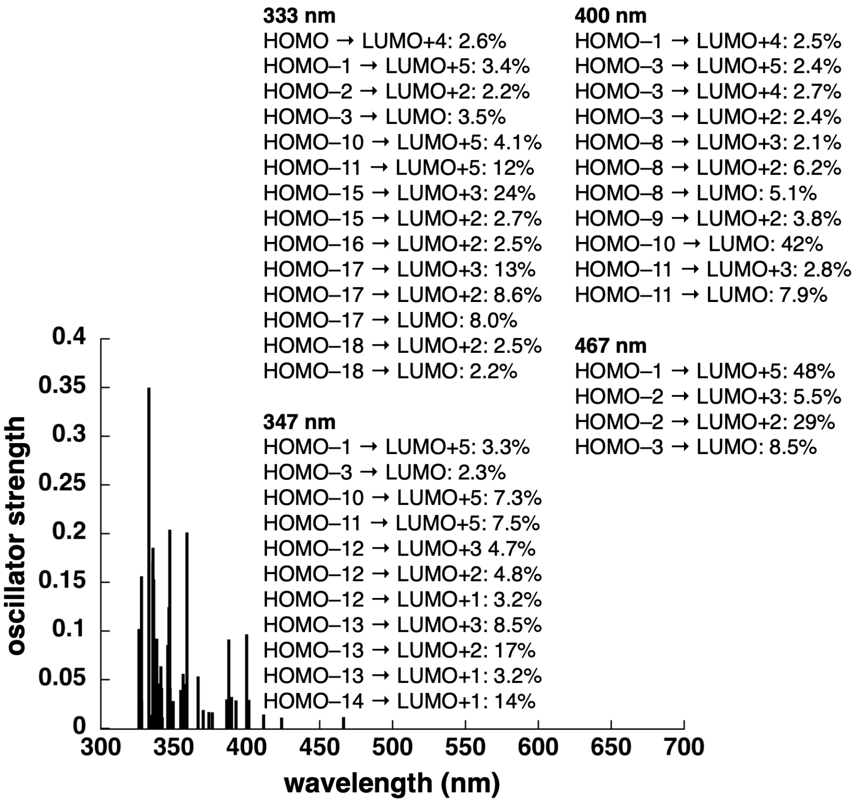

Fig. S47 TD-DFT-based UV/vis absorption stick spectrum of **2au**$^+$ as a stacked dimer in the crystal structure of **2au**$^+$-FABA$^-$ (Fig. S18) with the transitions correlated with MOs (Fig. S37) estimated at B3LYP-GD3BJ/6-31+G(d,p) with LanL2DZ for Au.

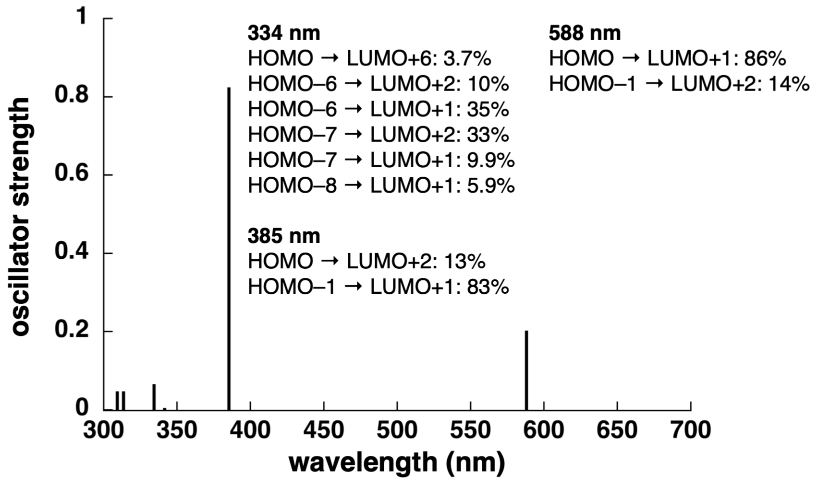

Fig. S48 TD-DFT-based UV/vis absorption stick spectrum of **1au**$^{+}$ with the transitions correlated with molecular orbitals (MOs) (Fig. S38) estimated at B3LYP/6-31+G(d,p) with B3LYP/LanL2DZ for Au based on the optimized structure (Fig. S29).

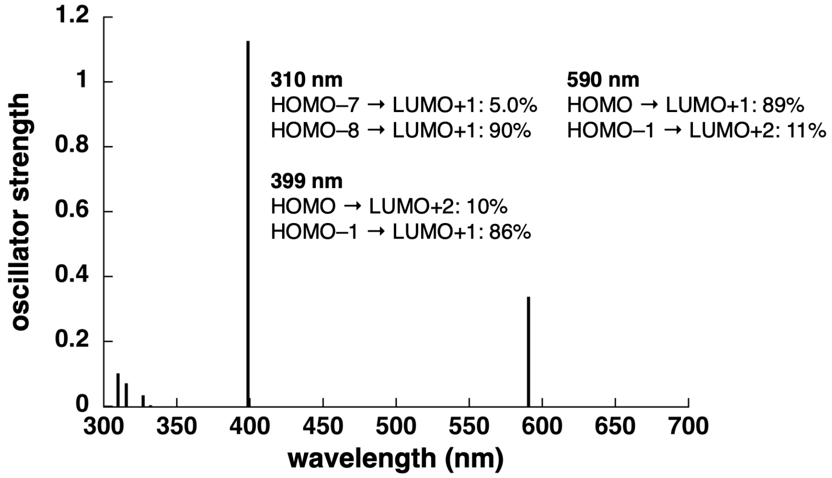

Fig. S49 TD-DFT-based UV/vis absorption stick spectrum of **1au**$^{+}$ with the transitions correlated with MOs (Fig. S39) estimated at PCM-B3LYP/6-31+G(d,p) with LanL2DZ for Au (DMSO) based on the optimized structure (Fig. S29).

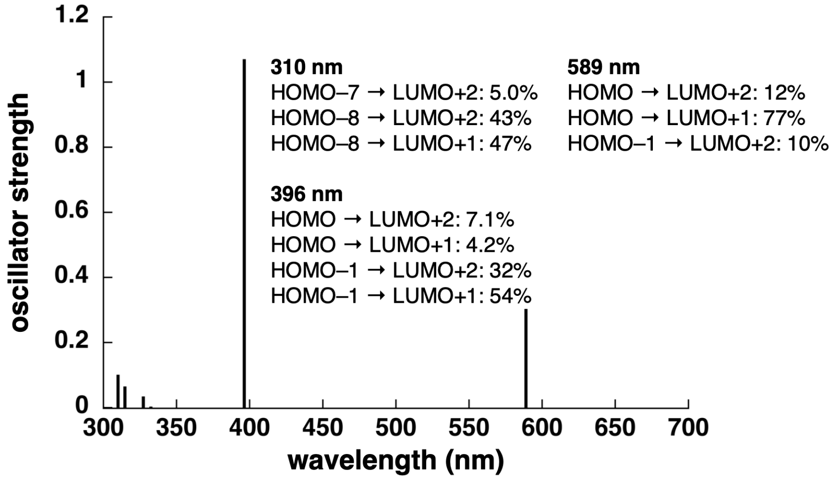

Fig. S50 TD-DFT-based UV/vis absorption stick spectrum of **1au**$^{+}$ with the transitions correlated with MOs (Fig. S40) estimated at PCM-B3LYP /6-31+G(d,p) with LanL2DZ for Au (CH_3CN) based on the optimized structure (Fig. S29).

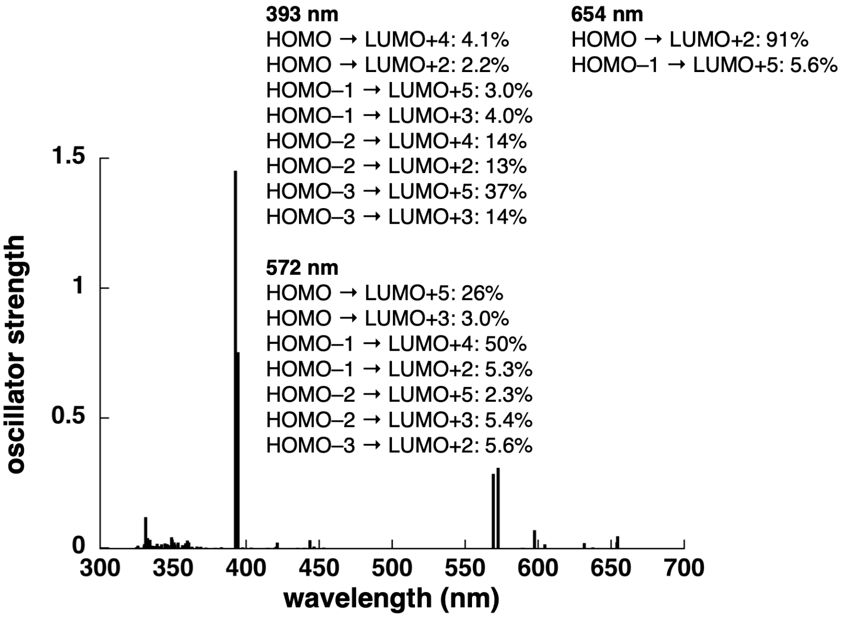

Fig. S51 TD-DFT-based UV/vis absorption stick spectrum of **1au**$^{+}$ as a stacked dimer with the transitions correlated with MOs (Fig. S41) estimated at PCM-B3LYP-GD3BJ/6-31+G(d,p) with LanL2DZ for Au (CH_3CN) based on the optimized structure for the stacked dimer in the single-crystal structure of **1au**$^{+}$-FABA^{-} (Fig. S30).

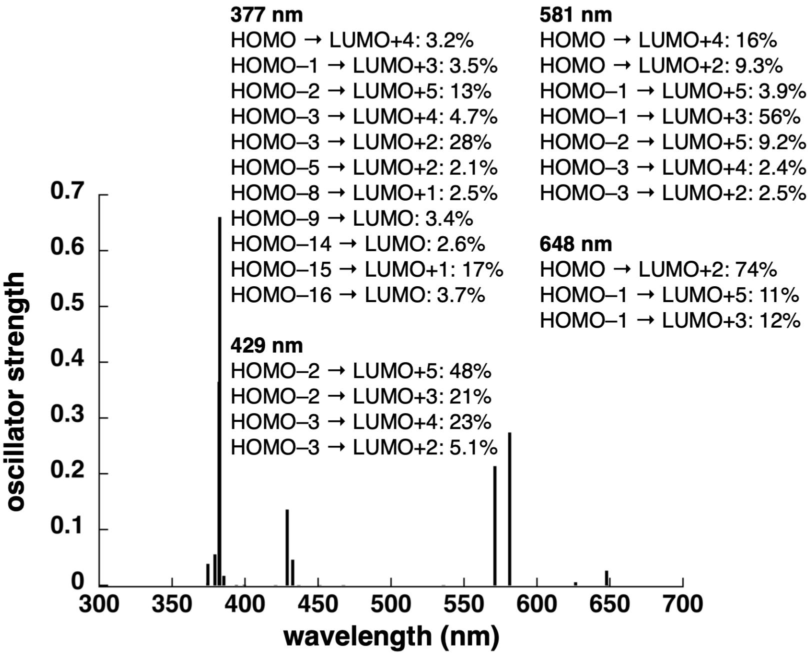

Fig. S52 TD-DFT-based UV/vis absorption stick spectrum of **1au**$^{+}$ as a stacked dimer in the crystal structure of **1au**$^{+}$-FABA^{-} (Fig. S20) with the transitions correlated with MOs (Fig. S42) estimated at B3LYP-GD3BJ/6-31+G(d,p) with LanL2DZ for Au.

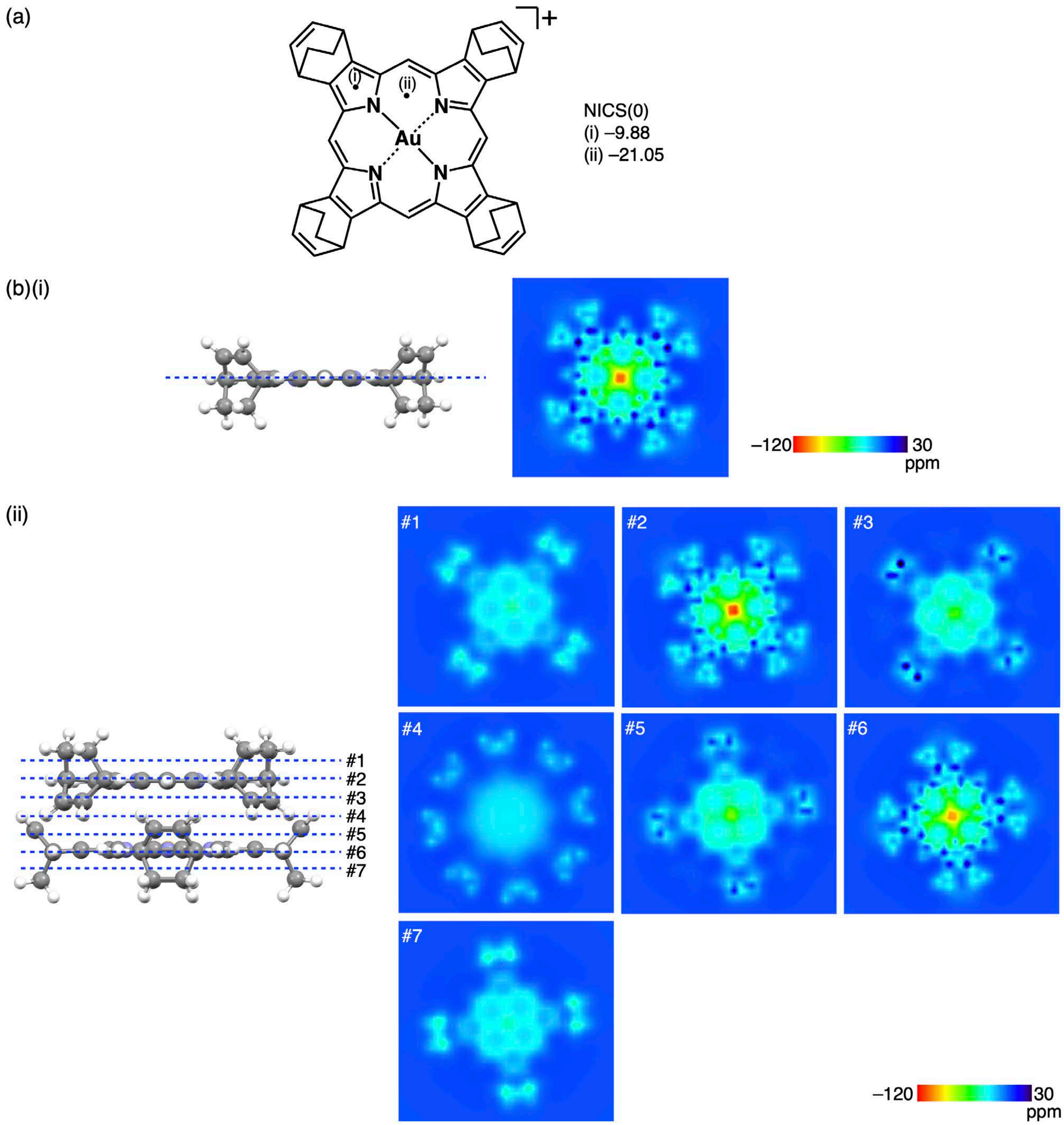

Fig. S53 (a) Nucleus-independent chemical shift (NICS(0)) values (ppm)[S22] of **2au**$^{+}$ and (b) 2D NICS[S23] of (i) **2au**$^{+}$ monomer and (ii) stacked **2au**$^{+}$ dimer based on the optimized structures at B3LYP/6-31+G(d,p) with LanL2DZ for Au (Fig. S29,30). In (i) and (ii), blue broken lines indicate the positions of cross sections (#1–7) for the 2D NICS calculations shown at right.

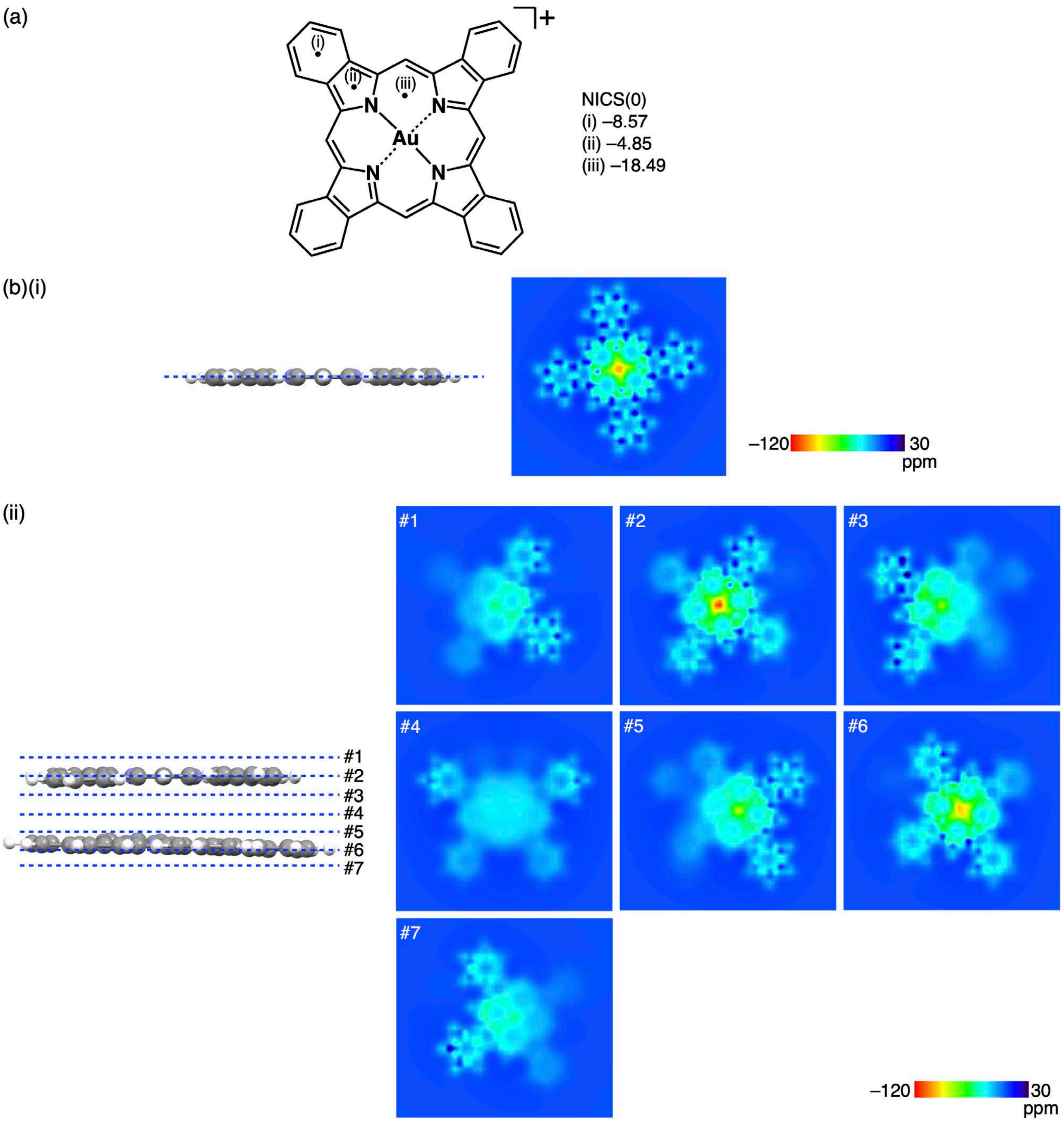

Fig. S54 (a) Nucleus-independent chemical shift (NICS(0)) values (ppm)[S22] of **1au**$^+$ and (b) 2D NICS[S23] of (i) **1au**$^+$ monomer and (ii) stacked **1au**$^+$ dimer based on the optimized structures at B3LYP/6-31+G(d,p) with LanL2DZ for Au (Fig. S29,30). In (i) and (ii), blue broken lines indicate the positions of cross sections (#1–7) for the 2D NICS calculations shown at right.

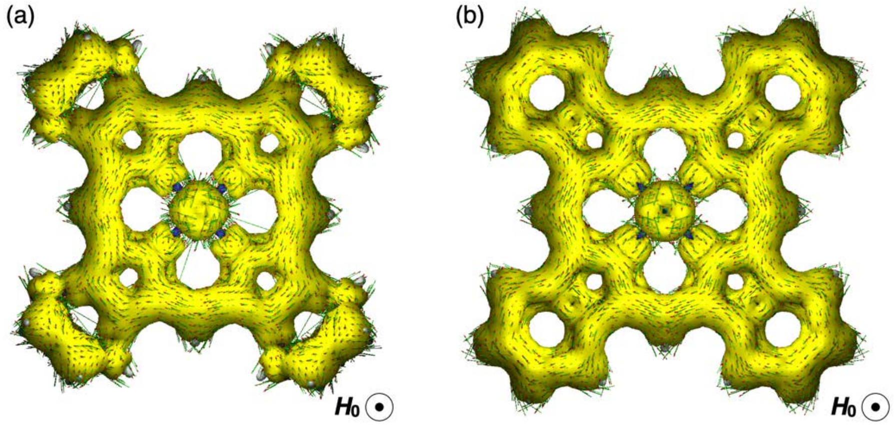

Fig. S55 Anisotropy of the current induced density (ACID)[S24] of (a) **2au**$^+$ and (b) **1au**$^+$ (top views) at the isosurface value of δ = 0.015 based on the optimized structure at B3LYP/6-31+G(d,p) with LanL2DZ for Au (Fig. S29). Current density vectors are plotted on to the ACID isosurface based on the vector of the magnetic field (H_0) which is orthogonal with respect to the molecule. In contrast to **2au**$^+$, **1au**$^+$ exhibits the ACID plots that are connected between the porphyrin and fused benzene rings.

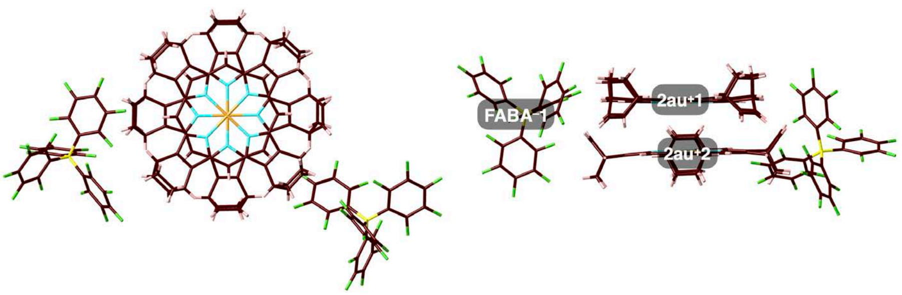

Fig. S56 Top and side views for the single-crystal X-ray structure of **2au**$^+$-FABA$^-$ for the energy decomposition analysis (EDA) calculation (Table S2).

Table S2 Energies between each fragment in **2au**$^+$-FABA$^-$ (Fig. S56) estimated by EDA calculation[S25] based on an FMO2-MP2 method using mixed basis sets using NOSeC-V-DZP with MCP with NOSeC-V-TZP with MCP for Au.[S26–28]

fragments	total interaction energy (E_{tot}) (kcal/mol)	electrostatic interaction energy (E_{es}) (kcal/mol)	dispersion interaction energy (E_{disp}) (kcal/mol)	charge-transfer interaction energy (E_{ct+mix}) (kcal/mol)	exchange repulsion interaction energy (E_{ex}) (kcal/mol)
**2au**$^+$1-**2au**$^+$2	–191.242	17.925	–230.853	–16.921	38.607
**2au**$^+$1-FABA$^-$1	–75.585	–40.260	–37.460	–3.700	5.834
**2au**$^+$2-FABA$^-$1	–58.154	–33.280	–26.122	–2.346	3.594

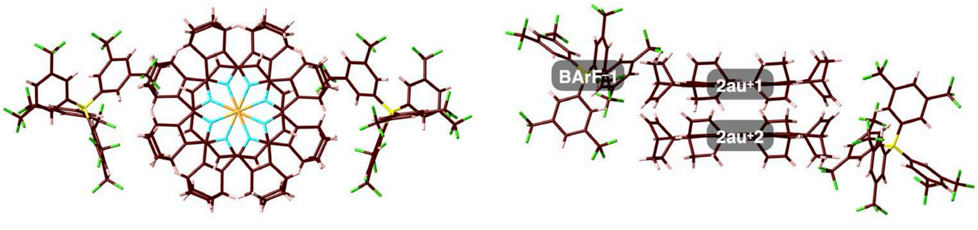

Fig. S57 Top and side views for the single-crystal X-ray structure of **2au**$^+$-BArF$^-$ for the EDA calculation (Table S3).

Table S3 Energies between each fragment in **2au**$^+$-BArF$^-$ (Fig. S57) estimated by EDA calculation[S25] based on an FMO2-MP2 method using mixed basis sets using NOSeC-V-DZP with MCP with NOSeC-V-TZP with MCP for Au.[S26–28]

fragments	total interaction energy (E_{tot}) (kcal/mol)	electrostatic interaction energy (E_{es}) (kcal/mol)	dispersion interaction energy (E_{disp}) (kcal/mol)	charge-transfer interaction energy (E_{ct+mix}) (kcal/mol)	exchange repulsion interaction energy (E_{ex}) (kcal/mol)
**2au**$^+$1-**2au**$^+$2	–206.333	14.633	–251.047	–21.142	51.223
**2au**$^+$1-BArF$^-$1	–91.161	–37.473	–57.444	–3.896	7.653
**2au**$^+$2-BArF$^-$1	–57.133	–31.488	26.175	–1.500	2.029

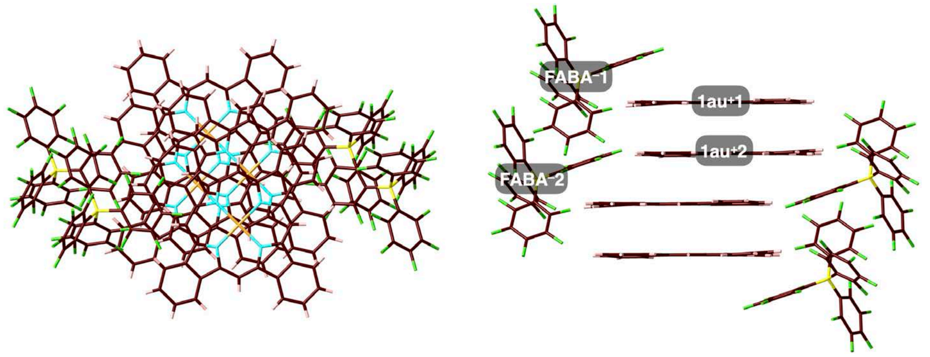

Fig. S58 Top and side views of single-crystal X-ray structure of **1au**$^{+}$-FABA^{-} for the EDA calculation (Table S4).

Table S4 Energies between each fragment in **1au**$^{+}$-FABA^{-} (Fig. S58) estimated by EDA calculation[S25] based on an FMO2-MP2 method using mixed basis sets using NOSeC-V-DZP with MCP with NOSeC-V-TZP with MCP for Au.[S26–28]

fragments	total interaction energy (E_{tot}) (kcal/mol)	electrostatic interaction energy (E_{es}) (kcal/mol)	dispersion interaction energy (E_{disp}) (kcal/mol)	charge-transfer interaction energy (E_{ct+mix}) (kcal/mol)	exchange repulsion interaction energy (E_{ex}) (kcal/mol)
**1au**$^{+}$1-**1au**$^{+}$2	–164.212	6.451	–213.973	–24.421	67.731
**1au**$^{+}$1-FABA^{-}1	–101.657	–47.747	–58.863	–5.956	10.909
**1au**$^{+}$1-FABA^{-}2	–37.555	–28.244	–9.661	–0.509	0.859
**1au**$^{+}$2-FABA^{-}1	–45.736	–34.216	–12.232	–0.888	1.600
**1au**$^{+}$2-FABA^{-}2	–56.586	–36.221	–20.184	–2.148	1.967
FABA^{-}1-FABA^{-}2	–22.644	29.091	–58.185	–5.790	12.241

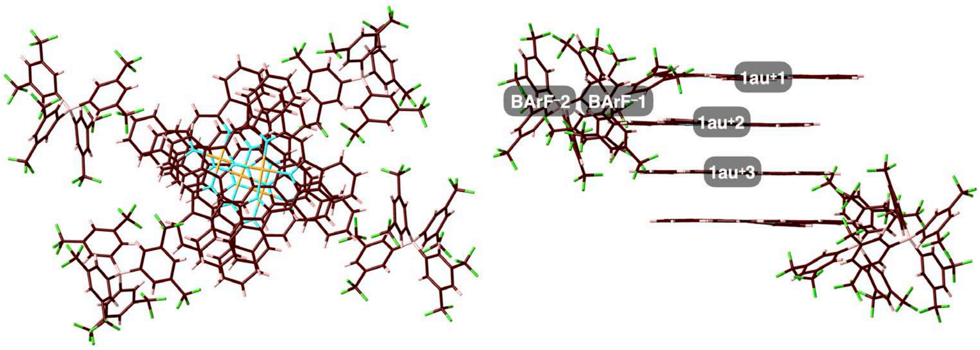

Fig. S59 Top and side views of single-crystal X-ray structure of **1au**$^{+}$-BArF^{-} for the EDA calculation (Table S5).

Table S5 Energies between each fragment in **1au**$^{+}$-BArF^{-} (Fig. S59) estimated by EDA calculation[S25] based on an FMO2-MP2 method using mixed basis sets using NOSeC-V-DZP with MCP with NOSeC-V-TZP with MCP for Au.[S26–28]

fragments	total interaction energy (E_{tot}) (kcal/mol)	electrostatic interaction energy (E_{es}) (kcal/mol)	dispersion interaction energy (E_{disp}) (kcal/mol)	charge-transfer interaction energy (E_{ct+mix}) (kcal/mol)	exchange repulsion interaction energy (E_{ex}) (kcal/mol)
**1au**$^{+}$1-**1au**$^{+}$2	–160.767	12.947	–209.110	–19.442	54.837
**1au**$^{+}$2-**1au**$^{+}$3	–181.205	14.520	231.287	–20.590	56.152
**1au**$^{+}$1-BArF^{-}1	–54.859	–33.184	–22.736	–2.223	3.284
**1au**$^{+}$1-BArF^{-}2	–35.926	–27.190	–9.066	–0.827	1.158
**1au**$^{+}$2-BArF^{-}1	–74.744	–36.075	–40.374	–2.793	4.498
**1au**$^{+}$2-BArF^{-}2	–75.735	–38.453	–40.477	–3.550	6.745
**1au**$^{+}$3-BArF^{-}1	–43.079	–30.180	–12.065	–1.001	0.166
**1au**$^{+}$3-BArF^{-}2	–35.796	–27.343	–8.259	–0.471	0.277
BArF^{-}1-BArF^{-}2	11.889	24.145	–12.677	–1.014	1.435

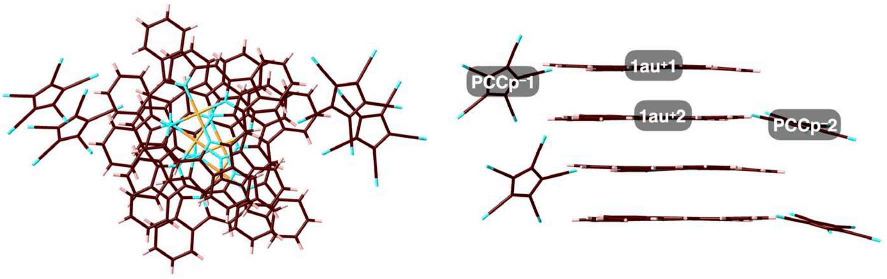

Fig. S60 Single-crystal X-ray structure of **1au**$^+$-PCCp$^-$ for the EDA calculation (Table S6).

Table S6 Energies between each fragment in **1au**$^+$-PCCp$^-$ (Fig. S60) estimated by EDA calculation[S25] based on an FMO2-MP2 method using mixed basis sets using NOSeC-V-DZP with MCP with NOSeC-V-TZP with MCP for Au.[S26–28]

fragments	total interaction energy (E_{tot}) (kcal/mol)	electrostatic interaction energy (E_{es}) (kcal/mol)	dispersion interaction energy (E_{disp}) (kcal/mol)	charge-transfer interaction energy (E_{ct+mix}) (kcal/mol)	exchange repulsion interaction energy (E_{ex}) (kcal/mol)
**1au**$^+$1-**1au**$^+$2	–171.890	12.027	–223.761	–23.600	63.444
**1au**$^+$1-PCCp$^-$1	–75.311	–48.522	–27.987	–3.392	4.589
**1au**$^+$1-PCCp$^-$2	–57.834	–39.379	–19.206	–1.128	1.880
**1au**$^+$2-PCCp$^-$1	–55.187	–37.195	–18.862	–1.196	2.066
**1au**$^+$2-PCCp$^-$2	–78.750	–53.305	–27.301	–4.151	6.007

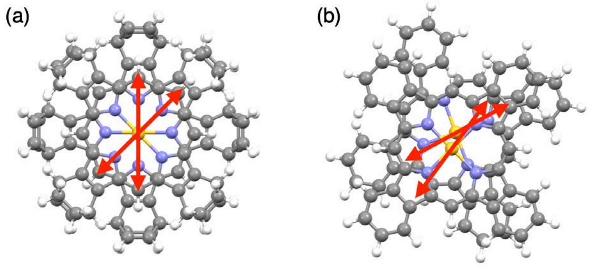

Fig. S61 Evaluation of the transition dipole moments for solution-state stacked dimers for (a) **2au**$^+$ and (b) **1au**$^+$. Representative transition dipole moments for **2au**$^+$ and **1au**$^+$ as monomers with the consideration of the D_{4h} symmetry are indicated by red arrows, which are superimposed on the stacked dimers optimized by PCM-B3LYP-GD3BJ/6-31+G(d,p) with LanL2DZ for Au (CH_3CN) (Fig. S30). Ground-to-excited-state transition dipole moment of monomers **2au**$^+$ and **1au**$^+$ are 1.43 D (364 nm (HOMO–6-to-LUMO: 72%)) and 9.49 D (396 nm (HOMO–1-to-LUMO+1: 54%)), respectively. Two transition dipole moments are arranged with rotation angle of nearly 45° and 26° for **2au**$^+$ and **1au**$^+$, respectively. Such arrangement of transition dipole moments showed blue-shifted absorption bands in the solution-state absorption spectra in the higher concentration/lower temperature conditions (Fig. S69–72).

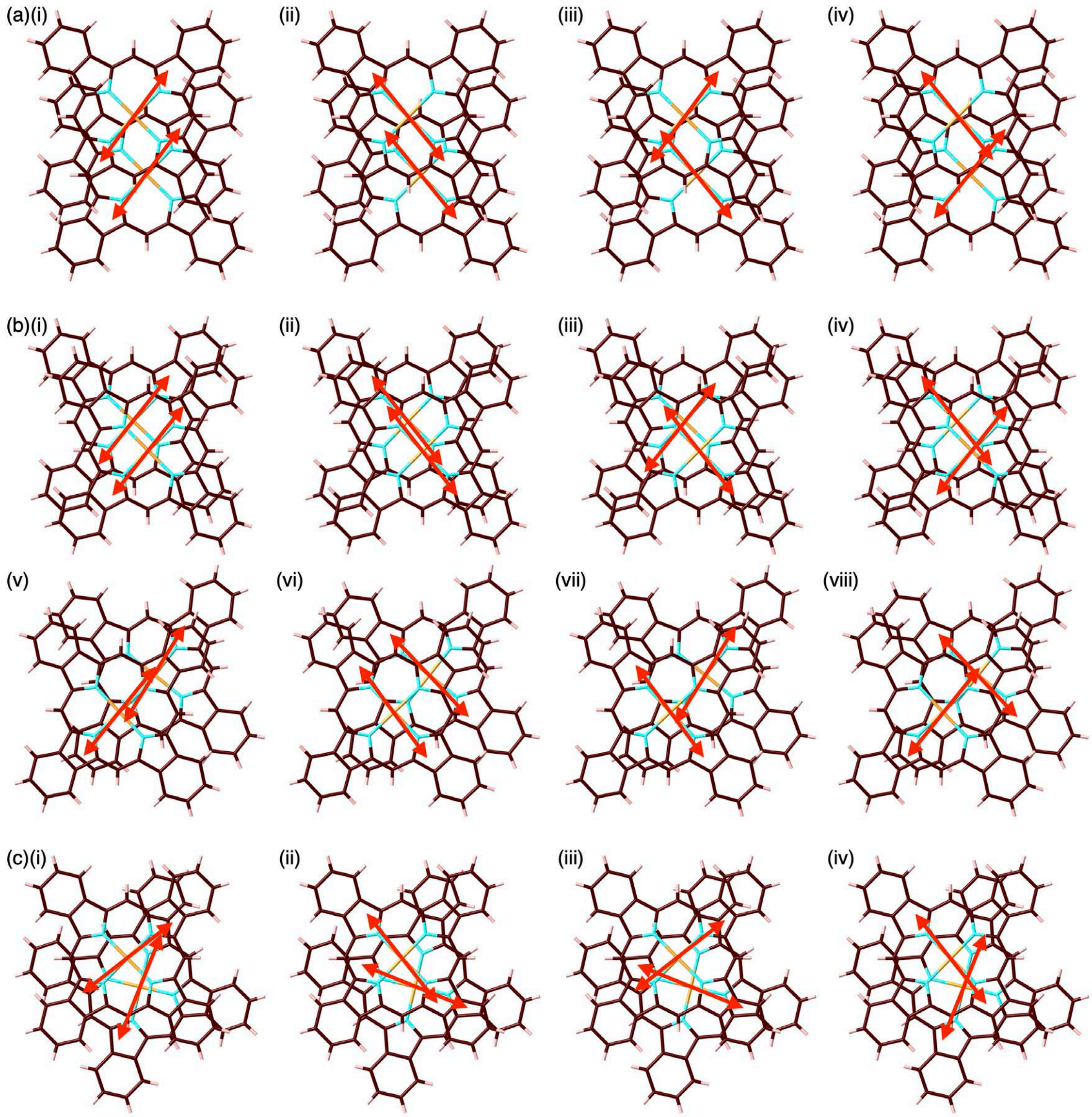

Fig. S62 Evaluation of the transition dipole moments for solid-state stacked dimers extracted from (a) **1au**$^+$-FABA$^-$, (b) **1au**$^+$-BArF$^-$ (two types of stacking modes), and (c) **1au**$^+$-PCCp$^-$ (Fig. S20–22). Representative transition dipole moments for each **1au**$^+$ with consideration of the D_{4h} symmetry is indicated by red arrows, which are superimposed on the stacked dimers. Each transition dipole moment was estimated by B3LYP/6-31+G(d,p) with LanL2DZ for Au. In (a)(i,ii), parallel-arranged transition dipoles show the angles of 49° and 61°, respectively, which showed red- and blue-shifted absorption bands (587 and 623 nm) in the crystal-state **1au**$^+$-FABA$^-$ compared to the solution-state monomer (616 nm) (Fig. S76). In (b)(i,ii,vi), the transition dipole moments are arranged in H-like arrangement, whereas (b)(v) showed J-like arrangement. The larger contribution of H-like arrangement showed a more blue-shifted absorption band (654 nm) in the solid-state absorption spectrum of **1au**$^+$-BArF$^-$ compared to the solution-state monomer (616 nm) (Fig. S76). In each case, nearly orthogonally arranged transition dipoles show very weak coupling and have less effect on the absorption spectra.

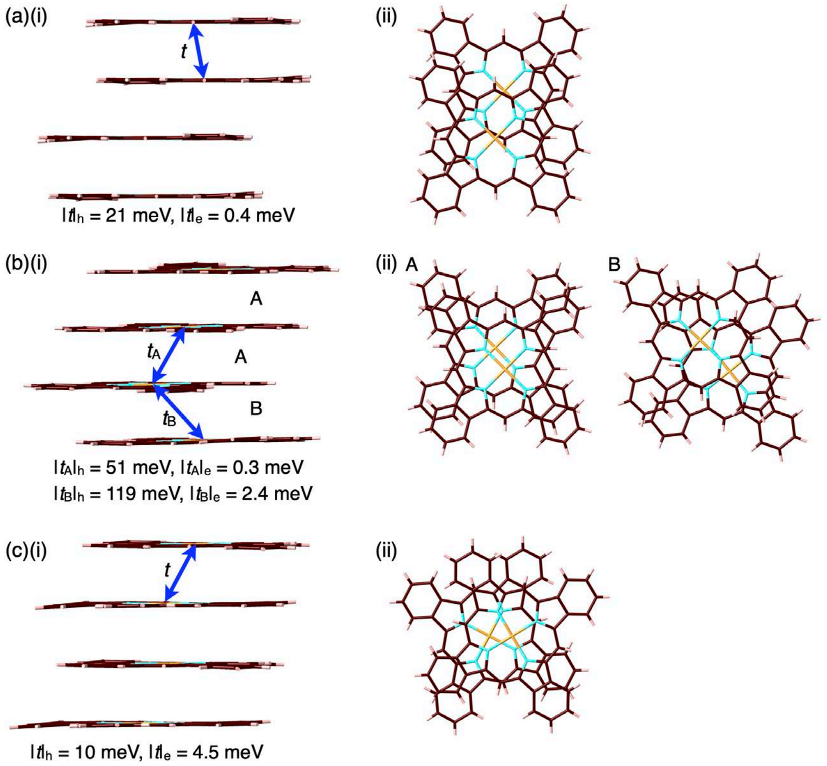

Fig. S63 Representative packing structures ((i) side views of the columns and (ii) top views of the stacked dimers) and theoretical evaluation of transfer integrals (t) at PW91/TZP for the stacked dimers in the crystal structures: (a) **1au**$^+$-FABA$^-$, (b) **1au**$^+$-BArF$^-$, and (c) **1au**$^+$-PCCp$^-$.[S29] **1au**$^+$-FABA$^-$, **1au**$^+$-BArF$^-$, and **1au**$^+$-PCCp$^-$ show hole transfer integrals ($|t|_h$) of 21, 51/119, and 10 meV, respectively, and electron transfer integrals ($|t|_e$) 0.4, 0.3/2.4, and 4.5 meV, respectively. The transfer integrals $|t|_h$ are larger than $|t|_e$ and the order of $|t|_h$ are consistent with the order of electric conductivity ($\phi\Sigma\mu$) values obtained by FP-TRMC measurements (Fig. S91).

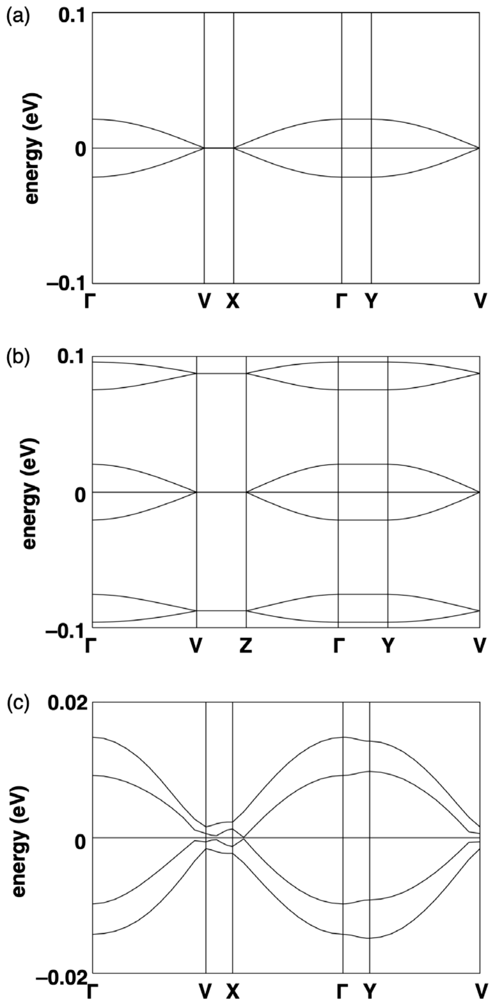

Fig. S64 Theoretical estimation of HOMO band dispersions based on the stacking of **1au**$^+$ in the crystal structures of (a) **1au**$^+$-FABA$^-$, (b) **1au**$^+$-BArF$^-$, and (c) **1au**$^+$-PCCp$^-$ using tight-binding approximation.[S30] The band dispersions exhibit typical one-dimensional band dispersion, which is consistent to the stacking structure of **1au**$^+$. The Fermi levels lie in the middle of the band gaps, suggesting semiconductive behaviors.

(a)

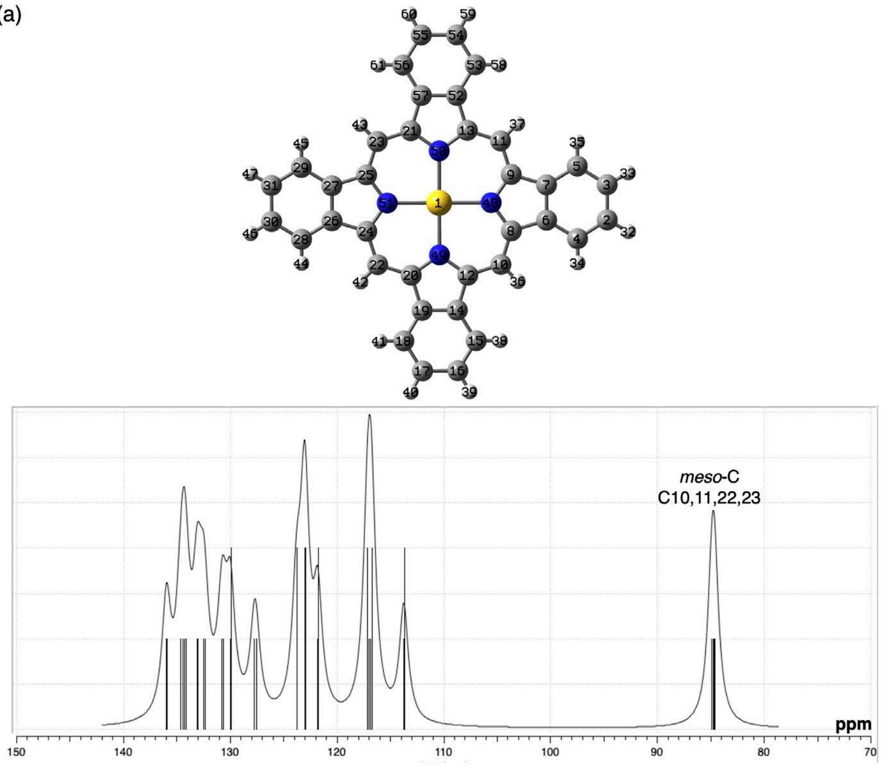

(b)

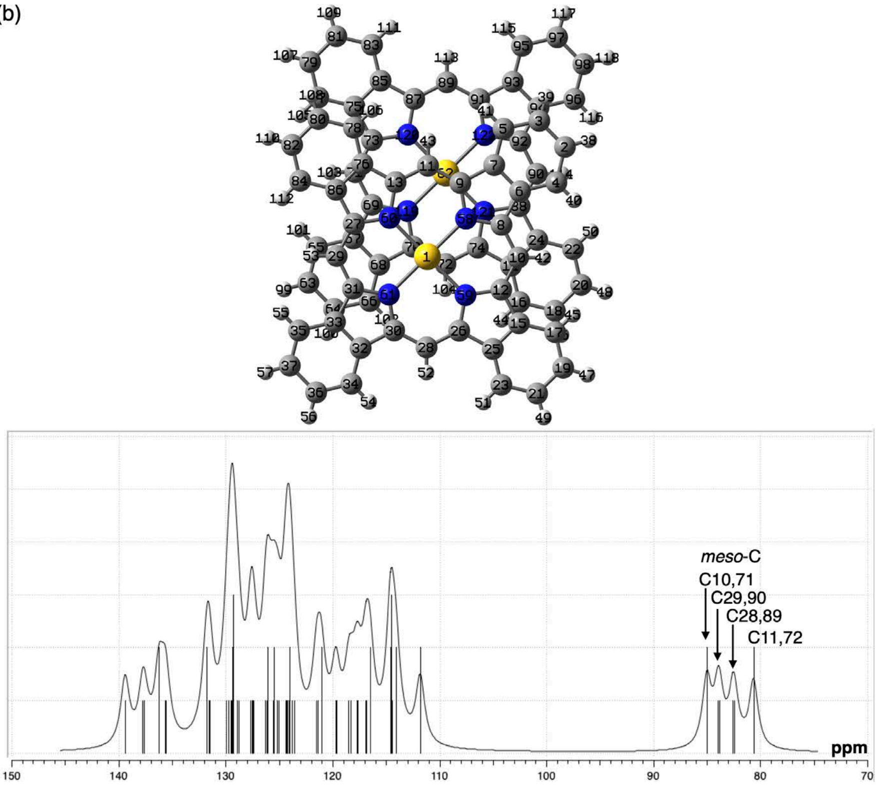

Fig. S65 Simulated ^{13}C NMR spectra for (a) **1au**$^{+}$ and (b) stacked **1au**$^{+}$ dimer at B3LYP/6-311+G(d,p) with SDD for Au based on the crystal structure of **1au**$^{+}$-FABA^{-} (Fig. S20). Theoretically estimated *meso*-^{13}C signal of the monomer **1au**$^{+}$ is observed at 84.8 ppm, whereas several *meso*-^{13}C signals of **1au**$^{+}$ as the stacked dimer are observed at the upfield-shifted region. In particular, C11 and C72 in the stacked **1au**$^{+}$ dimer, located on the top of the porphyrin core unit, are observed at 80.6 ppm, showing the more upfield shift by shielding effect. Such a signal split suggests the slipped stacking structure of **1au**$^{+}$ (Fig. S86).

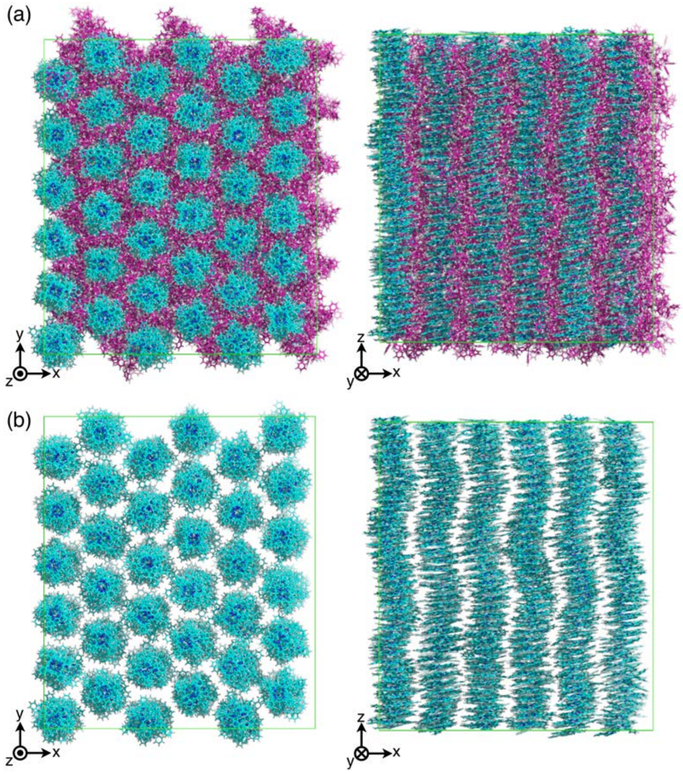

Fig. S66 Top and side views of MD simulation snapshot of **1au**$^+$-FABA^-_P as (a) whole structure and (b) **1au**$^+$ columns extracted from the whole structure after 100 ns of the equilibration run at 25 °C. The unit lattice constants after equilibration (a = 2.11 nm and c = 0.34 nm) were similar to those obtained by XRD measurements (a = 2.09 nm and c = 0.34 nm). As the initial structure for the MD simulation, the columns comprising tilted **1au**$^+$ units, as observed in the single-crystal structure, were transformed to those with barely tilted **1au**$^+$. **1au**$^+$ and FABA$^-$ are represented in cyan and magenta, respectively.

Cartesian Coordination of 2au$^+$
(B3LYP/6-31+G(d,p) with LanL2DZ for Au)
-2052.6165238 hartree
Au,0.0000023659,-0.0000121651,-0.0539466383
C,-0.2000619367,-3.4453125807,-0.0578459906
C,1.0715133076,-2.8919267472,-0.0549489069
C,2.3065092173,-3.6186239321,-0.0712706549
C,2.7490390686,-5.0562246354,-0.0452216161
C,3.5886083199,-5.1983989037,1.2874938294
C,4.7506740519,-4.1644490206,1.285590552
C,3.7599428379,-5.2323476919,-1.1775124733
C,4.7600584336,-4.3418760683,-1.1790193557
C,4.7040673599,-3.3157888411,-0.047959156
C,3.3273129198,-2.709871981,-0.0722510289
C,2.7484635872,-1.3990630939,-0.0556019659
C,3.445306967,-0.200079507,-0.0579652251
C,2.8919224729,1.071496036,-0.0549867727
C,3.618617338,2.3064930819,-0.0712548391
C,5.056218057,2.7490211963,-0.0452182435
C,5.1984356925,3.5884858505,1.2875612109
C,4.1644847892,4.7505485024,1.285784909
C,5.2323043563,3.7600161368,-1.1774328995
C,4.3418307934,4.7601302464,-1.1788319582
C,3.3157814591,4.7040488129,-0.0477429405
C,2.709863965,3.3272963378,-0.0721232646
C,1.3990564523,2.7484438859,-0.0554704417
C,0.2000724059,3.4452879063,-0.0577793991
C,-1.0715039078,2.8919043483,-0.0548610767
C,-2.3065001902,3.6186016107,-0.0711525431
C,-2.7490285608,5.0562016866,-0.0450208388
C,-3.7599818862,5.2323741989,-1.1772596227
C,-4.7600983226,4.3419033678,-1.1787612292
C,-3.5885377555,5.1983175219,1.2877367162
C,-4.7506024667,4.1643636128,1.2858414505
C,-4.7040576497,3.3157661892,-0.0477504162
C,-3.3273040674,2.7098494549,-0.0721284781
C,-2.7484546571,1.399039993,-0.0555077496
C,-3.4452961178,0.2000552297,-0.0578850781
C,-2.8919115748,-1.0715214388,-0.0549469959
C,-3.6186080819,-2.306517468,-0.071230575
C,-5.0562081689,-2.7490434833,-0.0451852367
C,-3.315775832,-4.7040749104,-0.0477812983
C,-2.7098556319,-3.3273222169,-0.0721386052
C,-1.3990476753,-2.7484717998,-0.0554971831
H,-0.2629018482,-4.5276763317,-0.0716362912
H,4.5276697992,-0.2629211765,-0.0718133101
H,0.262915495,4.5276516101,-0.0715537027
H,-4.5276593555,0.2628960657,-0.0717153098
H,1.9355417516,-5.7835411084,-0.0803991532
H,5.5205812102,-2.5919826417,-0.0855938557
H,5.7835351095,1.935528412,-0.0804825024
H,2.5919738417,5.5205654841,-0.0852875093
H,-1.9355321129,5.7835190159,-0.0802046853
H,-5.5205734554,2.5919618616,-0.0853814191

H,-5.7835242223,-1.9355485316,-0.080420461
H,-2.5919706403,-5.5205926284,-0.0853525261
H,2.9287547532,-5.0439969096,2.1467919195
H,3.9743368478,-6.2197305275,1.3463636218
H,5.7203211396,-4.6663055531,1.3428275169
H,4.6759653766,-3.4901197386,2.1442688215
H,3.6735929042,-6.0552383029,-1.8798340055
H,5.5862257526,-4.3522648491,-1.8827190899
H,5.0440650299,2.928561818,2.1468107743
H,6.2197692893,3.9742099218,1.346424714
H,4.6663404146,5.720192376,1.3430846328
H,3.4901808674,4.6757693949,2.1444768417
H,6.0551713063,3.6737229286,-1.8797891328
H,4.3521965773,5.5863536455,-1.8824662824
H,-3.6736610008,6.0552934951,-1.87955109
H,-5.5862947869,4.3523226337,-1.8824263013
H,-2.9286484282,5.0438810945,2.1470010992
H,-3.9742681282,6.2196450275,1.3466654965
H,-5.7202473053,4.666216158,1.3431500732
H,-4.6758490529,3.489993645,2.1444837732
H,-5.0440504709,-2.9286466921,2.1468427572
H,-3.4901313517,-4.6758240787,2.14443819
N,1.3685405178,-1.5372948524,-0.0462887429
N,1.5372902947,1.3685202847,-0.0462567977
N,-1.3685304381,1.5372732762,-0.0462147036
N,-1.5372803078,-1.3685480609,-0.0462488019
C,-5.1984147229,-3.5885455172,1.2875728501
C,-4.16445491,-4.7505990789,1.2857610757
C,-4.3418409781,-4.7601286002,-1.1788561634
C,-5.2323108876,-3.7600111633,-1.1774225123
H,-6.2197458205,-3.9742782774,1.3464221079
H,-4.6663007471,-5.7202479131,1.3430632813
H,-4.3522179667,-5.5863354842,-1.882509596
H,-6.0551880748,-3.6736998532,-1.8797645716

Cartesian Coordination of 2au$^+$

(PCM-B3LYP/6-31+G(d,p) with LanL2DZ for Au (DMSO))

-2052.6680848 hartree
Au,0.0000182666,0.0000054956,-0.0875699435
C,0.2094215739,3.445830734,-0.083394254
C,-1.0632403951,2.89397907,-0.082953555
C,-2.2967058534,3.6247052042,-0.0924277657
C,-2.7329592156,5.0637540803,-0.0498488299
C,-3.5597858489,5.1942828481,1.2918288562
C,-4.7245848976,4.1633208321,1.2914547936
C,-3.755450534,5.2541837669,-1.169172711
C,-4.7591566861,4.3656048236,-1.1696409521
C,-4.6949636421,3.327284132,-0.050502354
C,-3.3196490055,2.7191453472,-0.0926162896
C,-2.7439355071,1.4059677201,-0.0828609773
C,-3.4458078241,0.2094087909,-0.083250356
C,-2.8939552276,-1.0632555107,-0.0828740748
C,-3.6246747974,-2.2967251353,-0.09239236
C,-5.0637183545,-2.7329758771,-0.0497781634
C,-5.1942435286,-3.5597019595,1.2919601786
C,-4.1632096492,-4.7244833758,1.2917172969
C,-5.2540792274,-3.7555241436,-1.1690610719
C,-4.3656548793,-4.7593727356,-1.1693893769
C,-3.3272722277,-4.6950151565,-0.0502989515
C,-2.7191178525,-3.3196688402,-0.0925422495
C,-1.4059456193,-2.743950131,-0.0827971812
C,-0.2093856559,-3.4458198012,-0.0832302985
C,1.0632757398,-2.8939643772,-0.0828388953
C,2.2967469185,-3.6246762436,-0.092538747
C,2.7330130112,-5.0637125806,-0.0499789454
C,3.7557303352,-5.2540083483,-1.169121427
C,4.759609669,-4.3656174912,-1.1692167257
C,3.5595243236,-5.1943768022,1.2918978853
C,4.7242871078,-4.1632694851,1.2918990225
C,4.6950466576,-3.3272740479,-0.050117872
C,3.319697166,-2.719126948,-0.0925533838
C,2.7439732805,-1.4059569413,-0.0827703116
C,3.4458419065,-0.2093979274,-0.0832659113
C,2.8939891337,1.0632629629,-0.0827875604
C,3.6247103748,2.296730211,-0.0923600556
C,5.0637585293,2.732974276,-0.0498448621
C,3.3273090017,4.6950174449,-0.0504173653
C,2.7191543851,3.3196749532,-0.0925427935
C,1.4059803325,2.7439586013,-0.0828057082
H,0.2748648716,4.5281453031,-0.0885857816
H,-4.5281233453,0.2748510505,-0.0885466472
H,-0.2748248369,-4.5281342319,-0.0884671038
H,4.5281563195,-0.2748391216,-0.0885134695
H,-1.917307661,5.7871870833,-0.085843223
H,-5.5124512135,2.6057747313,-0.0870304035
H,-5.7871439482,-1.9173199865,-0.0858399854
H,-2.6057389704,-5.5124852477,-0.0867277488
H,1.9173607474,-5.7871350263,-0.0861576344
H,5.5125323918,-2.6057526312,-0.0863623816
H,5.7871793161,1.917312996,-0.0858813356
H,2.6057789517,5.5124898593,-0.0868549942
H,-2.889821728,5.0282062985,2.140805132
H,-3.941255704,6.2167628293,1.3657725605
H,-5.6931909693,4.6661381366,1.3653497527
H,-4.6413155718,3.4777792025,2.1401225493
H,-3.6729967894,6.0861411326,-1.8619910574
H,-5.5946702004,4.3848797562,-1.8629499494
H,-5.028193005,-2.8896654167,2.1408829553
H,-6.2167077993,-3.9412088835,1.3659344181
H,-4.666027925,-5.6930848778,1.3656957305
H,-3.4776968248,-4.6410688462,2.1403990666
H,-6.0859502478,-3.6729763107,-1.8619881711
H,-4.3849687555,-5.5949430595,-1.8624948113
H,3.673292482,-6.0858381814,-1.8621123891
H,5.5953011799,-4.3849102279,-1.8621768974
H,2.8893216073,-5.0282615148,2.1407133837
H,3.9409617275,-6.216732664,1.3658963249
H,5.6928934013,-4.6660657117,1.366016563
H,4.640759097,-3.4777468754,2.1405628003
H,5.0283250986,2.8897928085,2.1407952552
H,3.4778191458,4.6412029493,2.1402863747
N,-1.3647008653,1.5412538387,-0.0795419218
N,-1.5412312858,-1.3647130069,-0.0794118538
N,1.364735035,-1.5412418401,-0.0795152211
N,1.5412638843,1.3647230279,-0.0793691181
C,5.1943409096,3.5597879107,1.2918331302
C,4.163297599,4.7245629602,1.2915722673
C,4.3656684793,4.759309399,-1.1695376446
C,5.254072517,3.7554422614,-1.1692035209
H,6.2168078516,3.9412994876,1.3657498812

H,4.6661062586,5.6931719104,1.3655645728
H,4.384971084,5.5948433627,-1.862687658
H,6.0859164064,3.6728479314,-1.8621575489

Cartesian Coordination of 2au+
(PCM-B3LYP/6-31+G(d,p) with LanL2DZ for Au (CH_3CN))
-2052.6673777 hartree
Au,0.0000105014,0.0000093515,-0.0866288517
C,0.2088997945,3.4458386634,-0.0834367017
C,-1.06369886,2.8937007034,-0.0822799616
C,-2.2972342072,3.6243633,-0.0940875417
C,-2.732913202,5.063313988,-0.050619659
C,-3.5585448968,5.193536203,1.2920435159
C,-4.7236799463,4.1628164781,1.2920995881
C,-3.7562690714,5.2546062059,-1.1688946388
C,-4.760348285,4.3666174604,-1.1688000744
C,-4.6954814554,3.327488878,-0.05052576
C,-3.3203849014,2.7188318508,-0.0940697244
C,-2.7445146333,1.4057392994,-0.0822323084
C,-3.4458156441,0.2088926582,-0.0834190276
C,-2.8936754168,-1.0637066246,-0.0822873611
C,-3.6243370787,-2.297242146,-0.0941302076
C,-5.0632889752,-2.7329237565,-0.0507256276
C,-5.1935652232,-3.5585640525,1.2919283682
C,-4.1628576733,-4.7236969414,1.2920164523
C,-5.2545452706,-3.7562735958,-1.169008109
C,-4.3665415017,-4.7603375215,-1.1689041025
C,-3.3274566071,-4.6954828173,-0.050597829
C,-2.7188049942,-3.320393193,-0.0940862152
C,-1.4057123787,-2.7445214911,-0.082192089
C,-0.2088671913,-3.4458203695,-0.0833343006
C,1.0637304021,-2.8936792297,-0.0821884653
C,2.2972651908,-3.6243432826,-0.093929615
C,2.7329384235,-5.0632911219,-0.0503902138
C,3.7563227995,-5.2546458218,-1.1686237115
C,4.7603841152,-4.3666393292,-1.168571024
C,3.5585385308,-5.1934540093,1.2922982265
C,4.7236846443,-4.1627419435,1.2923269697
C,4.6955028559,-3.3274603007,-0.0503527691
C,3.3204148446,-2.7188111416,-0.0939274765
C,2.7445435306,-1.4057175408,-0.0821341764
C,3.445843795,-0.2088746887,-0.0833498574
C,2.8937040499,1.0637213771,-0.0822854854
C,3.6243679358,2.2972551204,-0.0941415599
C,5.0633181576,2.7329373703,-0.0506871693
C,3.3274897047,4.695497748,-0.0506722088
C,2.7188375157,3.3204063542,-0.0941561055
C,1.4057440214,2.7445365495,-0.0822820918
H,0.2735428413,4.5280692605,-0.0910574959
H,-4.5280461855,0.2735346599,-0.0910360573
H,-0.2735080066,-4.5280513353,-0.0909306021
H,4.5280747121,-0.2735166999,-0.0909336454
H,-1.9169014404,5.7866365314,-0.0870131649
H,-5.5138342168,2.6068136845,-0.0868183782
H,-5.7866136345,-1.9169156316,-0.0871499213
H,-2.6067879508,-5.5138424905,-0.086841909
H,1.9169311789,-5.7866195302,-0.0867812361
H,5.5138609305,-2.6067910779,-0.0866409658
H,5.7866439228,1.9169281363,-0.0870647752
H,2.6068220181,5.5138561548,-0.0869665609
H,-2.887935616,5.0269649963,2.1404922427
H,-3.9395721792,6.2160274219,1.3665264928
H,-5.6920254946,4.6657844826,1.3667688999
H,-4.6401790106,3.4768474407,2.1405369547
H,-3.6739616344,6.0865262786,-1.8616826093
H,-5.596090829,4.386419115,-1.8615916475
H,-5.027026437,-2.8879599048,2.1403877006
H,-6.216061565,-3.9395862335,1.3663682009
H,-4.6658194635,-5.6920469973,1.366636445
H,-3.4769001821,-4.6402079843,2.1404605261
H,-6.086455892,-3.6739608277,-1.8618063995
H,-4.3863073676,-5.5960858328,-1.8616983551
H,3.6740309643,-6.0866166563,-1.8613519923
H,5.5961504594,-4.3864662091,-1.8613418635
H,2.8879206338,-5.0268356547,2.1407255124
H,3.9395766351,-6.2159452986,1.3668281754
H,5.6920247881,-4.6657110938,1.3670173166
H,4.6401625807,-3.4767243344,2.1407182631
H,5.0269797732,2.888027618,2.1404240856
H,3.4768390407,4.6402629115,2.1403904303
N,-1.3652117695,1.5411286795,-0.0777372194
N,-1.5411023642,-1.36521968,-0.0777011568
N,1.3652408814,-1.5411045215,-0.07765293
N,1.5411308569,1.3652326606,-0.0777536281
C,5.1935437291,3.5586092832,1.2919519778
C,4.1628323272,4.7237384439,1.2919727164
C,4.3666166162,4.7603263748,-1.168941105
C,5.2546208342,3.7562632339,-1.1689851757
H,6.2160359629,3.9396378956,1.366414385
H,4.6657888416,5.6920915413,1.3666001402
H,4.3864086238,5.5960585558,-1.8617538942
H,6.0865586873,3.6739339928,-1.8617488772

Cartesian Coordination of 1au+
(B3LYP/6-31+G(d,p) with LanL2DZ for Au)
-1738.2781925 hartree
C,0.0495266742,-4.3146969886,0.001189638
C,0.5794509415,-5.6136823491,0.0013127899
H,1.6499690689,-5.7935530083,0.0012091187
C,-0.3119565882,-6.6807539148,0.0015086836
H,0.0684989517,-7.6973378062,0.0016485926
C,-1.7076980667,-6.4663400946,0.0016190422
H,-2.3757107563,-7.3218796949,0.0018466528
C,-2.237699249,-5.1809140278,0.0015331356
H,-3.3128448749,-5.0311436803,0.0016131029
C,-1.3422972324,-4.1008905819,0.0013051689
C,3.0087571699,0.6756152553,0.0009772343
C,2.6672179436,-1.547500742,0.0006278541
C,4.3147088351,0.0495414553,0.0008884808
C,4.100872397,-1.3422852201,0.0009494157
C,5.6136942605,0.5794576323,0.0011393509
C,5.1809035186,-2.2376920676,0.0010288107
H,5.7935761815,1.6499734849,0.0011723956
H,5.0311306055,-3.3128375974,0.0009136069
C,6.6807576826,-0.3119584405,0.0013475964
C,6.4663280063,-1.7077000826,0.001354188
H,7.6973431179,0.0684848325,0.001949854
H,7.321863304,-2.3757181417,0.0015871246
C,2.7785360993,2.0385817425,0.0008883178
C,2.0385434089,-2.7784895314,0.0004917335

H,3.6520410034,2.6794589684,0.0007602373
H,2.6794357786,-3.6519872298,0.0003280171
C,1.5475322196,2.6672224108,0.0010552336
C,0.675600316,-3.0087643141,0.0008355176
C,1.3422972324,4.1008905819,0.0013051689
C,2.237699249,5.1809140278,0.0015331356
H,3.3128448749,5.0311436803,0.0016131029
C,1.7076980667,6.4663400946,0.0016190422
H,2.3757107563,7.3218796949,0.0018466528
C,0.3119565882,6.6807539148,0.0015086836
H,-0.0684989517,7.6973378062,0.0016485926
C,-0.5794509415,5.6136823491,0.0013127899
H,-1.6499690689,5.7935530083,0.0012091187
C,-0.0495266742,4.3146969886,0.001189638
C,-0.675600316,3.0087643141,0.0008355176
C,-1.5475322196,-2.6672224108,0.0010552336
C,-2.0385434089,2.7784895314,0.0004917335
C,-2.7785360993,-2.0385817425,0.0008883178
H,-2.6794357786,3.6519872298,0.0003280171
H,-3.6520410034,-2.6794589684,0.0007602373
C,-2.6672179436,1.547500742,0.0006278541
C,-3.0087571699,-0.6756152553,0.0009772343
C,-4.100872397,1.3422852201,0.0009494157
C,-4.3147088351,-0.0495414553,0.0008884808
C,-5.1809035186,2.2376920676,0.0010288107
C,-5.6136942605,-0.5794576323,0.0011393509
H,-5.0311306055,3.3128375974,0.0009136069
H,-5.7935761815,-1.6499734849,0.0011723956
C,-6.4663280063,1.7077000826,0.001354188
C,-6.6807576826,0.3119584405,0.0013475964
H,-7.321863304,2.3757181417,0.0015871246
H,-7.6973431179,-0.0684848325,0.001949854
N,2.0469526171,-0.31436156,0.0004538736
N,0.3144253985,2.0469406031,0.0006728979
N,-0.3144253985,-2.0469406031,0.0006728979
N,-2.0469526171,0.31436156,0.0004538736
Au,0.0000000000,0.0000000000,0.0006856517

Cartesian Coordination of 1au+
(PCM-B3LYP/6-31+G(d,p) with LanL2DZ for Au (DMSO))
-1738.3361371 hartree
C,5.5116648891,-1.2093407456,0.0000355123
H,5.5667722917,-2.293297939,0.0000248633
C,4.2257840087,0.8724815524,0.0000276069
C,5.399882406,1.6412195539,0.0000656381
C,6.6180105132,0.9684230984,0.0000867816
C,6.673337665,-0.4433573615,0.0000717919
C,4.2812132309,-0.5347236146,0.0000127629
H,5.3706899081,2.7262169247,0.0000776956
H,7.5434492645,1.5360317722,0.0001157589
H,7.6402806238,-0.9369525419,0.0000897296
C,-5.3998751051,-1.6412117102,-0.0000067803
C,-2.3380757132,-2.5318577703,-0.0000373641
C,-1.0085836591,-2.9114649041,-0.0000394133
C,-0.5347046815,-4.2812004181,-0.0000147281
C,-1.2093244725,-5.5116495204,0.0000106693
C,-0.4433427279,-6.6733243527,0.0000342945
H,-5.370581172,-2.7262063166,0.0000055686
H,-3.0726693858,-3.3279676747,-0.0000112089
H,-2.2932817383,-5.5667544939,0.0000131058
H,-0.9369394239,-7.6402664884,0.0000544305
C,-1.2367591836,2.8225624078,-0.0000531936
C,-0.8724690761,4.2257624854,-0.0000062619
C,-1.6412112364,5.399857312,0.0000160469
C,-0.9684183647,6.6179881589,0.0000440078
C,-2.5318389225,2.3380846105,-0.0000636341
C,-2.9114422378,1.0085967954,-0.0000676654
C,-4.2811819833,0.5347164929,-0.0000477093
C,-5.5116331012,1.2093447575,-0.000033443
C,-6.6733176828,0.4433823199,-0.0000062147
C,-6.6179668738,-0.9683950848,0.0000070833
C,-4.2257311144,-0.8724876245,-0.0000347278
C,-2.8225398408,-1.23677685,-0.0000507683
H,-2.7262084774,5.3706613677,0.0000477074
H,-1.5360299986,7.5434250604,0.0000463887
H,-3.3279527257,3.0726742848,-0.000052424
H,-5.5667657619,2.2933009277,-0.0000414675
H,-7.6402399878,0.9370176037,0.0000065186
H,-7.5434038298,-1.536020039,0.0000296938
N,-2.0699098775,-0.0824934453,-0.0000826425
C,2.5318726242,-2.3380964446,-0.0000294518
C,1.2367981509,-2.8225739535,-0.0000395825
C,0.8725000715,-4.2257724361,-0.0000149541
C,1.6412357774,-5.3998713524,0.0000097654
C,0.9684369736,-6.6179989931,0.000033434
H,3.3279877187,-3.0726847226,-0.0000084789
H,2.7262332404,-5.3706810873,0.0000101334
H,1.536044927,-7.5434382197,0.0000525682
N,0.0825074939,-2.0699308158,-0.0000659068
Au,0.0000195217,-0.000002036,-0.0001409567
C,1.008622213,2.9114622663,-0.0000210371
C,0.5347356257,4.2811952688,-0.0000204487
C,1.2093487802,5.5116482491,0.0000033993
C,0.4433612725,6.6733191691,0.0000200522
C,2.3381099362,2.531854226,0.0000021213
C,2.8225862592,1.2367759205,-0.0000035279
C,2.9114800299,-1.0086052334,-0.0000229604
H,2.2933057969,5.5667595386,-0.0000282777
H,0.9369541629,7.6402633272,0.0000536032
H,3.0727021272,3.3279656386,0.00004201
N,-0.0824740003,2.0699243623,-0.0000641071
N,2.0699448519,0.0824887377,-0.0000414066

Cartesian Coordination of 1au+
(PCM-B3LYP/6-31+G(d,p) with LanL2DZ for Au (CH_3CN))
-1738.335651 hartree
C,5.5116754928,-1.2093077343,0.0000359066
H,5.5668042908,-2.293246022,0.000025139
C,4.2257797731,0.8724613244,0.0000264114
C,5.3998715914,1.6411926133,0.0000667977
C,6.6179886563,0.9684262392,0.0000897607
C,6.6733336478,-0.4433420672,0.0000743773
C,4.2812142599,-0.534736311,0.0000112237
H,5.3706714695,2.7261866468,0.0000793044
H,7.543420093,1.5360399828,0.0001205935
H,7.6402855474,-0.9369046906,0.0000938826
C,-5.3998598701,-1.6411855021,-0.0000047978
C,-2.3381260209,-2.5318581694,-0.0000398176
C,-1.0086445463,-2.9115152851,-0.0000425979
C,-0.5347176319,-4.2812010679,-0.0000158932

C,-1.2092917077,-5.5116597643,0.0000117446
C,-0.4433276906,-6.6733199705,0.0000372887
H,-5.3705678771,-2.726176944,0.0000077718
H,-3.0727909205,-3.3279422831,-0.000011533
H,-2.2932300701,-5.5667861482,0.0000144533
H,-0.936891836,-7.6402710438,0.0000591782
C,-1.2367709625,2.8226192944,-0.0000565645
C,-0.8724484068,4.2257586519,-0.0000096551
C,-1.6411838175,5.3998469272,0.0000125144
C,-0.9684210042,6.6179667143,0.0000439503
C,-2.5318395426,2.3381353287,-0.000066387
C,-2.9114931063,1.0086581675,-0.0000704869
C,-4.2811831358,0.5347299517,-0.0000482257
C,-5.5116434285,1.2093108911,-0.0000317766
C,-6.6733132773,0.4433640921,-0.000002249
C,-6.6179457726,-0.968400216,0.0000112222
C,-4.2257282911,-0.8724672343,-0.0000351369
C,-2.8225966243,-1.2367886624,-0.0000535182
H,-2.7261777224,5.3706433797,0.0000384047
H,-1.5360376827,7.5433963159,0.0000476657
H,-3.3279273012,3.0727964073,-0.0000533823
H,-5.5667974909,2.2932481549,-0.0000398256
H,-7.6402451682,0.9369655376,0.0000122551
H,-7.5433744115,-1.5360311309,0.0000357318
N,-2.0699033454,-0.0824724018,-0.0000877106
C,2.5318733342,-2.3381463829,-0.0000327621
C,1.2368099967,-2.8226300947,-0.0000431582
C,0.8724795935,-4.2257678502,-0.0000163365
C,1.6412085902,-5.3998601856,0.0000104172
C,0.9684398658,-6.6179767796,0.0000362231
H,3.3279624646,-3.072806039,-0.0000099142
H,2.7262027141,-5.3706622793,0.0000107628
H,1.5360528756,-7.5434086996,0.0000569701
N,0.0824866864,-2.0699237678,-0.0000714699
Au,0.0000194492,-0.0000017202,-0.0001528001
C,1.0086831611,2.9115133535,-0.0000240492
C,0.5347487576,4.2811966721,-0.0000192853
C,1.2093162348,5.5116592123,0.0000085319
C,0.4433464782,6.6733155323,0.0000255835
C,2.3381602179,2.5318552787,-0.0000003061
C,2.8226427365,1.2367880834,-0.0000067498
C,2.9115307674,-1.0086658192,-0.0000265856
H,2.2932543453,5.5667918755,-0.0000176965
H,0.9369068245,7.6402686232,0.0000608004
H,3.072823739,3.3279407665,0.000042297
N,-0.0824531169,2.0699180141,-0.00006947
N,2.0699381253,0.0824682343,-0.0000470225

Cartesian Coordination of 2au$^{+}_{2}$
(B3LYP/6-31+G(d,p) with LanL2DZ for Au)
-4105.7647402 hartree
Au,-0.0409493885,0.0115463953,1.6656603574
C,-1.5820546894,2.6078055615,-1.6921121403
C,-2.9377199025,3.070799597,-1.7019982192
C,-4.7440151242,4.4026778695,-0.7424995638
C,-5.5460110656,3.3297985725,-0.7429109297
C,-4.432162777,4.3042613408,-3.1831372202
C,-5.3626717286,3.0601139248,-3.1835919497
C,-5.2211411649,2.2883556741,-1.8095186451
C,-3.7569298548,1.9752133501,-1.7029213336
C,-2.929670505,0.8057812098,-1.6946716015
C,-3.3754080406,-0.5034727183,-1.6885132868
C,-2.5780053989,-1.6337263699,-1.6878363152
C,-3.0415523706,-2.9889799583,-1.6869194279
C,-4.3584498082,-3.7031313977,-1.7825240769
C,-3.2990751252,-5.589555911,-0.7073893963
C,-4.3710420863,-4.7862846835,-0.707904692
C,-2.1086325301,-2.7519227349,1.6671041163
C,-0.7460506316,-2.988030411,1.6816223248
C,-0.1149013276,-4.2740652336,1.6844297439
C,-0.539231232,-5.7108590114,1.7803065287
C,0.2189821578,-6.4845856623,0.7058935004
C,0.0728672931,-6.2154830638,3.1493969283
C,1.6106137491,-5.9943378298,3.1488312357
C,2.0534119579,-5.3361418819,1.7798661505
C,1.2391447765,-4.0786175904,1.6857327891
C,1.4811907644,-2.666879035,1.6871310759
C,2.7224956144,-2.0565466601,1.6879298536
C,2.9583491021,-0.6937223993,1.6953362874
C,4.2445905839,-0.0631730499,1.7041481281
C,5.6800478501,-0.4893189486,1.8102640073
C,-1.5629410849,2.6903903369,1.6675124702
C,-2.8038557548,2.0797317414,1.6574376197
C,-3.0398995552,0.7168256152,1.6613691788
C,-4.3256235073,0.0860286312,1.6439440951
C,-5.7638944388,0.5106063464,1.712809811
C,-6.2964225173,-0.1049567336,3.0695102894
C,-6.0751123036,-1.6427201587,3.069272967
C,-6.515797797,-0.2442696806,0.6204277517
C,-6.3243961637,-1.5701205893,0.6201505133
C,-5.3899607549,-2.0821014025,1.7126108823
C,-4.1304437305,-1.268259075,1.6445819593
C,-2.7191018753,-1.5105681928,1.6639772457
H,-4.4473818808,-0.6576534472,-1.6607694285
H,-2.7571007358,-3.6189494516,1.630891383
H,3.5895878985,-2.7054411425,1.6594631141
H,-3.6704389884,2.7285233779,1.6178816428
H,-5.8724142102,1.4149962818,-1.7731737139
H,-5.2295997717,-3.0487853204,-1.7465683431
H,-1.6176196569,-5.8655958517,1.7450338792
H,3.1314750741,-5.1789301027,1.7426578026
H,-5.9175995197,1.5891894088,1.6768779001
H,-5.232574469,-3.1601771674,1.6757821895
H,-7.3573049055,0.1372614976,3.1667508304
H,-5.7684828739,0.3660836616,3.9030863294
H,-5.4360820025,-1.946072236,3.9029819718
H,-7.0246597047,-2.1743523683,3.1658562258
H,-7.2159860994,0.2676321487,-0.0314608058
H,-6.851236291,-2.2588992094,-0.0320209746
H,-0.2908964446,-7.1978575454,0.0668353244
H,-0.1697450282,-7.2741470416,3.2676538479
H,-0.4004065596,-5.6706028698,3.9707201832
H,1.9119240544,-5.3390013926,3.9705487734
H,2.1419011712,-6.9418336191,3.2654634416
H,-6.420984917,3.2263012972,-0.1097384073
H,-3.7062662266,4.2501864138,-3.9989857787
H,-6.407466902,3.3534444741,-3.309968499
H,-5.1056259279,2.3796504856,-3.9999054442
H,-3.1935557938,-6.4598799934,-0.068238573
H,-5.2365530086,-4.9295730434,-0.0694178722

N,-1.6037914545,1.2190075975,-1.6850036541
N,-1.1895189273,-1.6558649193,-1.6808530318
N,0.2516204632,-2.0215161159,1.68100075
N,1.9923093863,0.3039565982,1.6864905166
N,-0.3332441173,2.0444301665,1.6739385843
N,-2.0736623515,-0.2809325137,1.6689652942
Au,0.0409180273,-0.0114349361,-1.6646898618
C,0.5326413967,-3.4275942302,-1.6681826972
C,1.6630248944,-2.6302468689,-1.6648754414
C,3.0181037941,-3.0933131351,-1.6453207517
C,3.7345984797,-4.4106246747,-1.7154286906
C,4.5476573539,-4.3500221836,-3.0713159755
C,5.4781259307,-3.1058750431,-3.0683755181
C,4.7990527036,-4.410427644,-0.6220289906
C,5.6017613786,-3.337950267,-0.6196715601
C,5.3038714933,-2.3131666128,-1.7103595465
C,3.837774137,-1.9977162961,-1.6423695649
C,3.0110781413,-0.8283570903,-1.6594834407
C,3.456655098,0.4811231888,-1.6550700162
C,-4.2832186215,-4.4931093671,-3.1514382157
C,-3.0391891085,-5.4237278477,-3.1505381821
C,-2.2617582358,-5.2734867826,-1.7806571255
C,-1.9464007145,-3.8089184938,-1.6848088169
C,-0.7765570985,-2.9821752893,-1.6817631108
C,1.5447002361,-6.2926310075,0.7053122043
H,0.686538569,-4.4993055848,-1.6326373926
H,3.0804473983,-5.2820149419,-1.6812681763
H,5.9550472244,-1.4397916113,-1.6720824165
H,-1.3891691262,-5.9256445641,-1.7437579321
H,5.8338074868,-1.5678657309,1.7736122492
H,2.2354634903,-6.8322116974,0.0658007902
H,3.8420690871,-4.308811677,-3.9055165382
H,5.1269100782,-5.2711476652,-3.1693108234
H,6.5255747419,-3.4009609655,-3.1651391057
H,5.2409100036,-2.4380336028,-3.900938967
H,4.9315086278,-5.2684940431,0.0287150841
H,6.4608737214,-3.2247999254,0.0333035711
H,-4.2328769221,-3.7734877694,-3.9730691825
H,-5.2032655417,-5.0704014381,-3.2692209323
H,-3.33316467,-6.4692864637,-3.2688104808
H,-2.3619210565,-5.1722627709,-3.9712255203
N,1.6850513743,-1.2415917022,-1.6687491784
N,1.2708136758,1.633165499,-1.6727629758
C,3.3671939717,5.56077354,-0.6585659968
C,2.3404885094,5.2517821774,-1.7443113646
C,0.8575065415,2.9589268916,-1.6751842557
C,-0.4519559108,3.404872437,-1.6801754024
C,-3.6526603263,4.3864807426,-1.8085696535
C,6.1742515842,0.1198924131,3.1844462443
C,5.9529825956,1.6576190196,3.184743091
C,5.3075588414,2.1036586931,1.8105662356
C,4.0499643276,1.2909262429,1.7039873976
C,2.6381300991,1.5336202715,1.6939067421
C,2.0276304423,2.7744865641,1.6816057082
C,0.6645294294,3.0102259257,1.6761231498
C,0.0343496272,4.2962698016,1.6629044845
C,0.4605586844,5.7333866671,1.7430624038
C,-0.1555193115,6.2556694841,3.1035225567
C,-1.6932786863,6.034623159,3.1003476426
C,-0.2926497162,6.4949594731,0.6562686354
C,-1.6187154036,6.3051703376,0.6535367998
C,-2.1325093316,5.3618620079,1.7372996532
C,-1.3199799952,4.1019724654,1.6590247161
H,-0.6067101581,4.4766749544,-1.6497154056
H,2.6767767681,3.6412716196,1.6511985869
H,1.4664923862,5.9024510941,-1.7101254706
H,-2.9996750425,5.2584762404,-1.7715780541
H,5.1518403635,3.1818839575,1.7745243475
H,1.5394185797,5.886075124,1.7089082234
H,-3.2106504012,5.2060056851,1.6989013691
H,7.2319900589,-0.1229189999,3.3103566174
H,5.6230705642,-0.3548473723,4.0007149417
H,5.2899904437,1.9573345967,4.0008782157
H,6.8993137088,2.1887208984,3.3112747446
H,0.0868499084,7.3157278551,3.2090502541
H,0.3148067383,5.7212133522,3.9333496294
H,-1.997253988,5.388134916,3.9280537489
H,-2.2247734783,6.983364917,3.2052581566
H,0.2205308325,7.1998489969,0.0104484199
H,-2.3063535391,6.8380004615,0.0051349203
H,5.299771316,4.8983624067,-0.0082760723
H,3.25468529,6.4259934838,-0.013549579
H,-4.8927255546,5.270851994,-0.1090120864
H,-5.0086753725,5.2236271336,-3.309716552
C,2.6597288998,1.6114388571,-1.6661467267
C,3.1224488112,2.9670113694,-1.6586180011
C,4.439010065,3.6839096577,-1.7384629673
C,4.3746109652,4.4835229546,-3.1022575524
C,3.1305059062,5.4140587286,-3.1054397917
C,4.4404455443,4.7591643289,-0.655840088
C,2.0265662228,3.786125764,-1.6627409049
C,6.4635034717,0.2699366617,0.7436888901
C,6.2731203761,1.5958568703,0.7439355546
H,4.5280649851,0.6356458533,-1.6144320922
H,5.3108910652,3.0308353531,-1.6999968972
H,7.181889364,-0.2395457723,0.1099979109
H,6.8189881598,2.2871175977,0.1104292948
H,4.3312741275,3.7696317641,-3.9292559029
H,5.2954866919,5.0617378308,-3.2083998231
H,3.4254144992,6.4606003137,-3.2120879608
H,2.4611384667,5.1693893623,-3.9346228074

Cartesian Coordination of 2au$^{+}_{2}$

(PCM-B3LYP/6-31+G(d,p) with LanL2DZ for Au (CH_3CN))

-4105.9202962 hartree

C,2.9752924128,0.7690348764,-1.667405343
C,4.2799851159,0.1771013739,-1.6706137082
C,5.7030521606,0.6473903168,-1.7605289936
C,6.2302348583,0.0510982394,-3.1263650568
C,6.0560531646,-1.4932228737,-3.1260483517
C,6.4934803803,-0.0853864366,-0.6806643362
C,4.1257510666,-1.1815373897,-1.6707845114
C,4.6479064671,4.554406734,0.6972013075
C,5.4772724442,3.5021101991,0.6882585027
H,3.5499944836,2.7982654734,-1.6342296303
H,5.8238482791,1.7300218453,-1.7265517909
H,4.7463429366,5.4129139425,0.0412748186
H,6.3279085311,3.401558834,0.022384973
H,5.6716584841,0.5075949919,-3.9482230022
H,7.2817034084,0.3267467353,-3.2413476929

H,7.0197506569,-1.9962499025,-3.2404963813
H,5.4100207457,-1.814237821,-3.9479134744
C,6.3431375845,-1.4166864749,-0.68047637
C,5.4091534644,-1.9562207999,-1.7600650127
C,2.7207659025,-1.4651770344,-1.6695537016
C,2.1464821143,-2.7236018589,-1.6686213486
C,0.6647749112,-5.7262771367,-1.7637192896
C,4.378320604,4.4591548894,3.1444813251
C,5.3415712737,3.2395871428,3.1344887025
C,5.1997039454,2.4594589856,1.7672238299
C,3.7423923887,2.1080930449,1.6842758733
C,2.9461493281,0.9172978966,1.6786440648
C,3.4282640883,-0.379608044,1.6719811861
C,2.6613712386,-1.5306455928,1.6710403528
C,3.1607806643,-2.8741516995,1.6744528288
C,4.4952357356,-3.5558138163,1.7726396279
C,4.4352089933,-4.345566728,3.140737202
C,3.2146160482,-5.3077031355,3.1371801377
C,4.5355226452,-4.6373540276,0.6968398949
C,3.4835424318,-5.4669239139,0.6936723981
C,2.4377786057,-5.178018347,1.7665415728
C,2.087526845,-3.7211891899,1.6708473462
H,2.8195763601,-3.5718054004,-1.6422068219
H,4.5040197741,-0.5046168494,1.6485880828
H,5.2872442876,-3.0382863699,-1.7240303293
H,1.7466485655,-5.8488827157,-1.7221656668
H,5.873343945,1.603982731,1.7263530395
H,5.3503114162,-2.881478599,1.7387296631
H,1.5819232886,-5.8514293766,1.7286438628
H,4.9318627658,5.3946540686,3.2595120641
H,3.6649864756,4.3874987695,3.9702571439
H,5.113670954,2.5543470256,3.9557006528
H,6.3808317367,3.5602721886,3.2436652149
H,5.3699504262,-4.8990555739,3.2620069306
H,4.3629230432,-3.6249494242,3.9601037968
H,2.527678134,-5.071264121,3.9545381655
H,3.5340045302,-6.3462123728,3.2569119292
H,5.3954580784,-4.7419991632,0.043796105
H,3.3867511072,-6.325900989,0.0381366702
H,7.1669578644,0.4522023446,-0.0216849341
H,6.880242984,-2.0900531973,-0.020966378
H,0.4561866523,-7.1845683563,-0.0212337957
H,0.3500131483,-7.3104364784,-3.2398602883
Au,-0.0005037724,-0.0227428297,-1.6430225453
C,-2.1461620347,2.6811520635,-1.6528081072
C,-0.7903816179,2.9549243362,-1.6545762816
C,-0.1974443543,4.2596643767,-1.653200589
C,-0.6653406769,5.6836151599,-1.7424328764
C,-0.0720321624,6.208895158,-3.1102913537
C,1.4721112111,6.0320040068,-3.114087082
C,0.0719513225,6.4739351628,-0.6655514148
C,1.4030035009,6.3218024037,-0.6689746131
C,1.9377933094,5.3855452565,-1.7486153386
C,1.1608058779,4.1040535078,-1.6564049524
C,1.4433142804,2.6989375314,-1.6588638012
C,2.7017464295,2.1251121844,-1.6600529114
C,-6.2249488144,-0.0673247946,-3.1567937945
C,-6.0481535459,1.4767042261,-3.1352017383
C,-5.4053885711,1.9199375627,-1.7608214625
C,-4.1248394546,1.140526968,-1.6764790134
C,-2.7200105953,1.4223974384,-1.6652423268
C,-3.4721844831,5.509547281,0.697797445
H,-2.8193473547,3.5292961745,-1.6237423907
H,-1.7473377523,5.8062928567,-1.7043005182
H,3.0197199255,5.2608457589,-1.7157703112
H,-5.2800894223,3.0012206528,-1.7100862956
H,2.9009372263,5.3706159289,1.7553088392
H,-3.366865456,6.3705152554,0.0461670658
H,-0.5317044911,5.6506150771,-3.9306018005
H,-0.3461614532,7.2607749016,-3.2251918734
H,1.976475516,6.9947365151,-3.2308213215
H,1.7897562482,5.384661155,-3.9362271064
H,-0.4622197271,7.1508268309,-0.0072074619
H,2.0793490282,6.8605154004,-0.0138484154
H,-5.6633503517,-0.5137896762,-3.9821036882
H,-7.2763486604,-0.3394907136,-3.2804227098
H,-7.0105632502,1.9828910229,-3.2464664614
H,-5.3985627958,1.8075270673,-3.950353957
N,0.2339938503,2.0191164372,-1.6541522748
N,2.0403007632,-0.2557016393,-1.6633706175
Au,0.0005681512,0.022536333,1.642926547
C,0.7902721903,-2.997649548,-1.6761959574
C,0.1970072608,-4.3018734417,-1.6812234126
C,-0.0759199498,-6.5112037943,-0.6849187119
C,-1.406980017,-6.3585823997,-0.6929909391
C,0.0756946165,-6.2579843685,-3.1307341808
C,-1.4683985941,-6.0810362358,-3.1401607004
C,-1.9382506701,-5.4280144881,-1.7795263647
C,-1.1615853666,-4.1460669436,-1.689790017
C,-1.4440161189,-2.7414502547,-1.6897479817
C,-2.7025935044,-2.1671391043,-1.6948096776
C,-2.9768432588,-0.8113503576,-1.6901224551
C,-4.2811901001,-0.2177342175,-1.6938248298
C,-5.7053489275,-0.682623233,-1.7966748179
C,-6.3451384049,1.3689837329,-0.6921497281
C,-6.4990896499,0.0382466621,-0.7110354454
C,-3.4277008942,0.4212947634,1.6570461908
C,-2.6602459057,1.5725422102,1.6682199387
C,-3.1603283418,2.9153152616,1.6753579652
C,-4.4970677737,3.5942650155,1.7565006161
C,-4.5228934313,4.6783713929,0.6826927109
C,-4.458893116,4.3803140962,3.1273674734
C,-3.239485847,5.3438229953,3.1449043767
C,-2.4421850245,5.2195247497,1.7857421789
C,-2.0877472884,3.7635456903,1.6897131601
C,-0.8964371927,2.9676022229,1.6892554171
C,0.4005768418,3.4488228055,1.6941422079
C,1.5518172794,2.6817409662,1.6909385451
C,2.8948395126,3.1812259501,1.692459249
C,3.5765193388,4.5161674548,1.7835115863
C,0.8968216816,-2.9242275792,1.6634663576
C,-0.3998504925,-3.4060568125,1.6529086114
C,-1.5507274462,-2.638713333,1.6514647742
C,-2.8934443483,-3.1394366509,1.6481914534
C,-3.5714461947,-4.4761693656,1.7363800592
C,-4.3618771744,-4.4280106576,3.104594885
C,-5.326305478,-3.2092360558,3.1095298467
C,-4.6523782964,-4.5113434976,0.6599949968
C,-5.484074934,-3.4610394569,0.6640934467
C,-5.198084681,-2.4221931664,1.7445372872

C,-3.7419887114,-2.0674152605,1.6514814084
C,-2.9461099777,-0.8754475404,1.6549861268
H,-3.550946812,-2.8404204594,-1.6802438192
H,-4.5033017933,0.546546859,1.6300675808
H,0.5261637316,4.5245861276,1.6786472962
H,-0.5250661839,-4.481725599,1.6263105862
H,-3.0203912942,-5.3034397824,-1.7508660107
H,-5.8294482835,-1.7649201552,-1.7766050495
H,-5.3503063964,2.918129088,1.7087561398
H,-1.5872188745,5.8947541664,1.7630262128
H,-2.8940844812,-5.3288318922,1.6972704214
H,-5.873712518,-1.567956808,1.7130121272
H,-4.9135922132,-5.364713945,3.2186637006
H,-3.6417543988,-4.3604465637,3.9248119478
H,-5.0913357976,-2.5277703526,3.9318948594
H,-6.364214771,-3.531452362,3.2268133592
H,-4.7558279692,-5.367544884,0.0018017579
H,-6.3431449283,-3.3616058915,0.0090779304
H,-5.373587758,4.7836477047,0.0175764655
H,-5.3960092179,4.9323962734,3.2360723651
H,-4.3981848628,3.657569342,3.9458174987
H,-2.5645491773,5.1055858834,3.9717003552
H,-3.5618327923,6.3815734951,3.2632580982
H,-2.0855434203,-6.8932695156,-0.0367713733
H,0.5379312477,-5.7036964689,-3.9522929606
H,-1.9724572637,-7.0442515023,-3.2540613148
H,-1.7833240423,-5.4375892427,-3.9664129165
H,-6.8846725625,2.0354434154,-0.0275177493
H,-7.1787706726,-0.5057339404,-0.0637320985
N,-0.2342464503,-2.0620559472,-1.676183343
N,-2.040715607,0.2129280453,-1.6686111741
N,-1.274315285,1.6324600364,1.670163318
N,1.6113326294,1.2953866478,1.6777583233
N,1.2749666991,-1.589562099,1.6604892218
N,-1.6107858796,-1.2527816575,1.6502670466

Cartesian Coordination of 1au$^{+}_{2}$
(B3LYP/6-31+G(d,p) with LanL2DZ for Au)
-3476.9490833 hartree
C,-0.6863199433,3.981652355,1.7631624237
C,-0.7102789931,5.3817900187,1.7307868535
C,-1.935341724,6.0147612646,1.9180417173
C,-3.1155280517,5.2711791198,2.1355434405
C,-4.7514985373,4.0868672384,-0.8088860203
C,-1.8672360249,3.239369461,1.9700503507
C,-1.7119287542,3.3515533203,-1.3744144814
C,-0.3899966579,3.0280807569,-1.596338946
C,0.6833240035,3.9828526138,-1.7627933582
C,0.7061397836,5.3830123421,-1.7307241408
C,1.9306667959,6.0169602461,-1.9182157609
H,0.1922447443,5.9624946273,1.5719864308
H,-1.986112158,7.0987162446,1.9089051973
H,-4.0527768288,5.7965097613,2.2864058037
H,-4.1961970583,5.01335591,-0.9058025425
H,-1.9514935684,4.4051705212,-1.3055970312
H,-0.1968371139,5.9629643714,-1.5717799226
H,1.9805517796,7.1009542018,-1.9093109865
C,-3.390570424,-1.8406863897,-1.1818226776
C,-3.7633254073,-3.2380985922,-1.152391884
C,-5.0002123107,-3.8762745557,-1.0001208375
C,-5.0197574975,-5.2665851445,-1.00443966
C,-4.2616240856,-0.7838215497,-1.0166810841
C,-3.9313584786,0.5542443878,-1.0301732475
C,-4.8627247052,1.643955077,-0.8227632054
C,-6.236735056,1.6668571043,-0.5467383125
C,-6.8501788335,2.9072994623,-0.4036589682
C,-6.1149789291,4.1053551649,-0.536357467
C,-3.0969132479,3.8810013337,2.1628212516
C,-4.12832384,2.8401471124,-0.9482033294
C,-2.7557999916,2.4658148364,-1.2129059108
H,-5.9201564706,-3.3134073968,-0.8835771634
H,-5.9636646779,-5.7903846521,-0.8952499507
H,-5.3027562319,-1.0306356219,-0.8516755844
H,-6.815248697,0.7534123658,-0.4535622299
H,-7.9135824593,2.9587069956,-0.1945440293
H,-6.6278227588,5.0553740188,-0.4277169894
H,-4.0130863712,3.3222742373,2.320995536
N,-2.6768855585,1.0877446791,-1.2546074441
C,2.3754245123,0.7838169138,-2.0400588234
C,1.4989331505,1.8416939503,-1.9122129427
C,1.8648145323,3.2414890668,-1.96960564
C,3.0939611541,3.8840884385,-2.162545729
C,3.1114543991,5.2742917076,-2.1356162784
H,3.4180221507,1.0301405341,-2.1984902845
H,4.0105444468,3.3260514519,-2.3207159606
H,4.0482500304,5.8003659158,-2.2867341716
N,0.137913553,1.7542414807,-1.6893357819
Au,-0.9457312433,0.0000832288,-1.5281569973
C,-1.4980956945,-3.0285378127,-1.4573503573
C,-2.5771757631,-3.9821037415,-1.3128127662
C,-2.6003601362,-5.3827033825,-1.3096692105
C,-3.8311399882,-6.0125575845,-1.1529382308
C,-0.1727403136,-3.3527696654,-1.657028958
C,0.8754752779,-2.4696190133,-1.8083293364
C,2.0531983049,-0.5568631343,-1.985857257
H,-1.6957131253,-5.9685352951,-1.4381548223
H,-3.8830285296,-7.0964100066,-1.1558749111
H,0.0704021986,-4.407292027,-1.7004169889
N,-2.0257169689,-1.753524585,-1.369411134
N,0.7919377752,-1.0893329361,-1.7928032526
C,2.7539698785,2.4672450264,1.2133008621
C,4.1262494828,2.8426404748,0.9488813381
C,4.7484698868,4.0898607967,0.8098709237
C,6.1120468186,4.1094798815,0.537901072
C,1.7094196613,3.3521838799,1.3747858524
C,0.3877328817,3.0277171138,1.596707267
C,-1.5002712558,1.8398544146,1.9125747943
H,4.1923541964,5.0158689373,0.906602301
H,6.6241757895,5.0599163055,0.4295513159
H,1.9481982951,4.4059791362,1.3059015582
N,-0.1392022988,1.7534685638,1.6896228588
C,-2.3759425406,0.7812962301,2.0403884935
C,-2.0527263648,-0.5591330999,1.9859314241
C,-2.9925068636,-1.6497934447,2.1263476982
C,-4.3779454616,-1.6743579756,2.3372452161
C,-4.9972061015,-2.9144238988,2.4402925918
H,-3.4187084736,1.0268166077,2.1989556119
H,-4.9545973912,-0.7596289215,2.4290708467
H,-6.0670414286,-2.9674926318,2.6142973427
Au,0.9457103313,0.0001152356,1.5279661832

C,3.9310241616,0.5565895051,1.0307070309
C,4.8616287674,1.6470296365,0.823649247
C,6.2357348682,1.6710585936,0.5481960135
C,6.8482506172,2.9120069881,0.4054644476
C,4.2622779196,-0.7812297903,1.017078459
C,3.3919685326,-1.8387688964,1.1818311756
C,3.7657596316,-3.2358977967,1.152099456
C,5.0031455388,-3.873115762,0.9998760949
C,5.0237191473,-5.2634130833,1.0038021633
C,3.8356229248,-6.0103036269,1.151856695
C,4.3792883232,-1.6703289537,-2.3370215307
C,2.5801303393,-3.9808250836,1.3120765925
C,1.5003242712,-3.0280981712,1.456728263
H,6.8150196587,0.7580851179,0.455184609
H,7.9117042251,2.9642683824,0.1968165479
H,5.3036216661,-1.027248596,0.8522200081
H,5.9226935256,-3.309535731,0.8836510381
H,5.968033477,-5.7864838006,0.8946358684
H,3.8883112144,-7.0941185807,1.1545030548
H,4.9553094316,-0.7551668491,-2.4284972439
N,2.6760784657,1.0891161087,1.2548080433
N,2.0270162076,-1.7526753992,1.369198186
C,2.2569938585,-2.8433432317,-2.0178015601
C,2.8881146799,-4.0907298235,-2.1209628556
C,4.2602479538,-4.1086031915,-2.3338017793
C,4.9994512889,-2.9099305512,-2.440272299
C,2.6043465874,-5.3814047002,1.3085269063
C,2.9937973058,-1.6468105832,-2.1263440848
C,0.1752018568,-3.3533568492,1.6562787206
C,-0.8736426866,-2.4709991758,1.8078433069
C,-2.2548630121,-2.8457665089,2.0174065046
C,-2.8850789356,-4.0936321743,2.1203160369
C,-4.2571663419,-4.1125432387,2.3333442969
H,2.3331442385,-5.0186867834,-2.0367484535
H,4.7766599766,-5.0585261115,-2.4182673054
H,6.069356751,-2.9621838093,-2.6140914116
H,1.7001081774,-5.9679428869,1.436658205
H,-0.067164185,-4.4080638212,1.6994795896
H,-2.3294526394,-5.0211713206,2.0357906309
H,-4.7728929696,-5.0628556227,2.4176113586
N,-0.7911065646,-1.0906515305,1.7925689368

Cartesian Coordination of 1au$^{+}_{2}$
(PCM-B3LYP/6-31+G(d,p) with LanL2DZ for Au (CH_3CN))
-3477.1231749 hartree
C,-1.4669005283,-3.0161342358,1.4468994812
C,-2.5584697679,-3.9531518227,1.2817208562
C,-2.6005199896,-5.3531673135,1.2601746908
C,-3.8398367423,-5.9622225542,1.0851956501
C,-0.1494679366,-3.3641265814,1.6607906864
C,0.9068991723,-2.4961983401,1.8413980723
C,2.1103519751,-0.6061959057,2.0511960251
H,-1.7024060722,-5.9511230697,1.3747956271
H,-3.9050734499,-7.0452873219,1.0631845733
H,0.0799608127,-4.4220901155,1.6828670179
N,0.8454101093,-1.1171833166,1.8465656788
C,2.4554108191,0.7287984925,2.1078295698
C,1.5959225495,1.7995200402,1.9706281182
C,1.9877494301,3.1935082072,2.0288319715
C,3.2279640853,3.8130321728,2.225018091
C,3.2711998254,5.2031051945,2.192291147
H,3.500656627,0.9583971785,2.2715519644
H,4.1336250213,3.2360104336,2.3780774901
H,4.220122316,5.7109990965,2.3290442245
Au,-0.86872574,0.0009954745,1.5567049467
C,-3.3363324067,-1.7977450166,1.1594699119
C,-3.7316161987,-3.1896028561,1.1164631383
C,-4.9768432241,-3.8066880025,0.9446129451
C,-5.0163219514,-5.1970840928,0.9351494665
C,-4.190190105,-0.727700029,0.9882143128
C,-3.8381784239,0.60500313,1.015173343
C,-4.7511427079,1.7102119371,0.804361995
C,-6.1179255773,1.7553570744,0.4973247267
C,-6.7086600641,3.0071863424,0.351699779
C,-5.9585302817,4.1931556444,0.5109007923
C,-3.2277471267,3.8117567391,-2.2280511753
C,-4.0013227743,2.8938587636,0.9529313394
C,-2.637058802,2.4951016039,1.2283812882
H,-5.8840324435,-3.2268686863,0.8131991522
H,-5.9656780134,-5.7047042019,0.7988746699
H,-5.2312256899,-0.9577778892,0.8013423572
H,-6.703233479,0.8502074369,0.3732901333
H,-7.7648010489,3.0757450531,0.1117320665
H,-6.4506154132,5.1526229853,0.3888990025
H,-4.1335706785,3.2347338862,-2.3801092836
N,-1.9737891656,-1.7347622358,1.3638635749
N,-2.5809313704,1.1177138868,1.2608229163
C,-0.8207723738,3.9542297786,-1.8171207307
C,-0.8690143581,5.3535745393,-1.7797451783
C,-2.1050520906,5.965034766,-1.9709335765
C,-3.2707156485,5.2018879517,-2.1970604889
C,-4.6012120489,4.151537074,0.8114869989
C,-1.9876329572,3.192230089,-2.0312475129
C,-1.5815453311,3.3647845632,1.4057946746
C,-0.2675809727,3.0172578352,1.6402046388
C,0.8210147304,3.9554367767,1.8137844379
C,0.8695098521,5.3547326327,1.7747980417
C,2.1056713522,5.9661859963,1.9652017946
H,0.0207809602,5.9485599105,-1.6030511193
H,-2.1764306597,7.0475617727,-1.9441708575
H,-4.219543612,5.7097831634,-2.3344702678
H,-4.0297579843,5.067071797,0.9163849233
H,-1.8034177773,4.4221534138,1.3374795434
H,-0.0201758885,5.9496796462,1.5974173878
H,2.1772558916,7.0486648075,1.9370854905
N,0.2375440795,1.7367542885,1.7415357437
C,2.2819226592,-2.895068588,2.0568592755
C,2.8936230466,-4.1533873761,2.1374836892
C,4.2674733969,-4.1935557805,2.3422193493
C,5.0253109389,-3.0076875141,2.4593148346
C,2.6003387831,-5.3540884856,-1.2560283886
C,3.0363348065,-1.7114806829,2.1827328257
C,0.1492817079,-3.3652555284,-1.6577855101
C,-0.9070723093,-2.4974247558,-1.8389799077
C,-2.2821112372,-2.8964002354,-2.0540892198
C,-2.8938521576,-4.1547584013,-2.13383503
C,-4.2677055441,-4.1950260654,-2.3385041933
H,2.324122995,-5.0697684693,2.0290952443
H,4.7722257122,-5.1521101572,2.3948427063
H,6.0983419331,-3.076952716,2.6053156743

H,1.7022347869,-5.952109804,-1.3703770725
H,-0.0801685758,-4.4232312833,-1.6791163988
H,-2.3243674989,-5.0710782492,-2.0248563448
H,-4.7724865123,-5.15360029,-2.3904730858
C,3.8381807985,0.6042326851,-1.0153065768
C,4.7512211964,1.7096128873,-0.8056522577
C,6.118041298,1.7550243616,-0.4988323879
C,6.708848626,3.0069817553,-0.3546025874
C,4.1901091485,-0.7284663372,-0.9871163122
C,3.3361872734,-1.7986098456,-1.1574797653
C,3.7314285815,-3.1904461602,-1.1135036764
C,4.9766488896,-3.8074323949,-0.9412452728
C,5.016127628,-5.1978211962,-0.9309870929
C,3.8396491433,-5.9630439149,-1.0806583702
C,4.4229781956,-1.7566483732,2.3806308371
C,2.5582849436,-3.9540859314,-1.2783572869
C,1.4667276608,-3.0171595118,-1.4441415922
H,6.7033380914,0.8499900947,-0.3739248155
H,7.7650222802,3.0757477617,-0.1148378555
H,5.2311352731,-0.9584636402,-0.8000737359
H,5.8838308927,-3.2275244751,-0.8101704594
H,5.9654782313,-5.7053673003,-0.7943978339
H,3.9048894716,-7.0460959426,-1.0580496195
H,5.0146418735,-0.8512332192,2.4654381697
N,1.9736512859,-1.7357435451,-1.3619748254
C,-2.4555184398,0.7274349661,-2.1078002056
C,-2.1104723535,-0.6075248372,-2.0500422639
C,-3.0364939922,-1.7128766301,-2.180738097
C,-4.4231456447,-1.7581399479,-2.3785554923
C,-5.025515392,-3.0092133228,-2.4563761043
H,-3.500815674,0.9568951011,-2.2714381271
H,-5.0147749504,-0.8527617608,-2.463976769
H,-6.0985512894,-3.0785531304,-2.6023020162
N,-0.845538151,-1.1184047827,-1.8450891046
Au,0.8686789317,-0.0001510468,-1.5562803932
C,2.6371475039,2.4941785129,-1.2303092937
C,4.0014525293,2.8931447924,-0.9553777878
C,4.6014090046,4.1509551656,-0.8153776777
C,5.9587693906,4.1928207116,-0.5150044231
C,1.5817022435,3.3637539747,-1.4085788876
C,0.2677067909,3.0160936822,-1.6426212117
C,-1.5959621673,1.7982478306,-1.9716767975
H,4.0300480798,5.0664308065,-0.9213541666
H,6.4509349614,5.1523896073,-0.3941295671
H,1.803652246,4.4211735123,-1.3413003356
N,2.5809242328,1.1167580074,-1.2612978062
N,-0.2375563077,1.7355574242,-1.7426348813

Cartesian Coordination of 1au$^{+}_{2}$-2FABA^{-} (B3LYP/6-31+G(d,p) with LanL2DZ for Au)
-9350.0465247 hartree
F,13.1882526827,5.0940698328,-1.1111949824
F,11.4861006979,-2.5553679763,-4.8711620329
F,-13.1870899335,-5.095684201,1.1063479201
F,-11.485927344,2.552836171,4.8696032266
Au,-1.7202493277,0.3380820622,1.6069492014
B,-10.5882364303,-0.0451540792,-0.2968675808
C,2.3545982712,-4.9521426373,1.7640530404
C,3.3753236217,-3.9761236923,1.7611692545
C,1.0114260355,-4.5921450463,1.7517035893
C,3.0718514627,-2.6206520984,1.747079908
C,0.6989376627,-3.2255275155,1.7352434978
C,1.7198033183,-2.2529529135,1.7338879319
C,-0.5602552643,-2.5150223271,1.7040823155
C,1.0705304395,-0.9612383405,1.7026377931
C,-1.8083398648,-3.1054924937,1.7008556404
C,1.7246704959,0.2554495983,1.6857194063
C,-3.021425856,-2.4545639386,1.663904506
C,1.1333481136,1.501014169,1.6472622799
C,-1.8451447537,-2.7591362683,-1.614209069
C,-4.3161902632,-3.1010812037,1.6682228754
C,-3.2118045398,-3.063749364,-1.6140349345
C,-4.6962170187,-4.448234635,1.7023925168
C,-3.5796300299,-4.4014824233,-1.5358242136
C,-6.0570808302,-4.7379186268,1.6929777294
C,-2.6075727935,-5.4219204781,-1.4570519114
C,-7.0264044324,-3.7126551512,1.6567740559
C,-1.2477953354,-5.1249688963,-1.4664975354
C,-6.6539165367,-2.3731048956,1.6262797681
C,-0.8720379219,-3.7775377377,-1.5512414895
C,-5.2849362899,-2.0794881748,1.6233897746
C,-4.5698864723,-0.8211591914,1.5878142689
C,-0.4205165197,3.1289482314,1.5701783811
C,-5.1655748867,0.4234109762,1.5458986116
C,-1.6372515487,3.7803192956,1.5465617215
C,-4.5112285336,1.6379854094,1.5384436141
C,-2.880749643,3.1899127578,1.5447383606
C,-5.1606631577,2.9327042808,1.5072914676
C,-4.1399603074,3.9026948888,1.5089717484
C,-6.5109611238,3.3002280071,1.4634237829
C,-4.4453995098,5.2681288843,1.4580980809
C,-6.8093199816,4.6569872123,1.4072867607
C,-5.7879807875,5.6308255294,1.4051270224
C,-11.3537891485,-1.4764662646,-0.0004295342
C,-10.7531244096,0.7458256264,1.1432165225
C,-10.8417306936,-2.3316183998,0.9761326934
C,-12.032615407,1.1132555711,1.5762231467
C,-11.4142005552,-3.5371873679,1.3553032941
C,-12.3017714945,1.7158661964,2.8000538842
C,-12.6068611699,-3.9354623276,0.7605967192
C,-11.2547045522,1.9691841065,3.6830701387
C,-13.1881323913,-3.1062213821,-0.1906757647
C,-9.9681187788,1.597421331,3.3174797685
C,-12.5658843115,-1.9071487577,-0.5447538546
C,-9.7561185346,0.9927985432,2.0806444362
C,-11.1958959356,0.7780477532,-1.5909058138
C,-8.9992324873,-0.2167061422,-0.7519730156
C,-11.2039015518,0.1500480906,-2.8406489363
C,-8.2310548288,0.9405747117,-0.9288760875
C,-11.6051000059,0.7587800949,-4.0230130781
C,-6.9258706546,0.9506847067,-1.3990542707
C,-12.0149140905,2.0886904141,-3.9951856568
C,-6.3151892874,-0.2540912312,-1.7208350686
C,-12.0025228609,2.7705174676,-2.785212766
C,-7.0297055383,-1.4324518738,-1.5851434022
C,-11.5904446505,2.1157753044,-1.6240539242
C,-8.3468074735,-1.3932010504,-1.1226864427
F,-9.687135421,-1.9993523977,1.6184059933
F,-13.095467816,0.8834383309,0.7763240348
F,-10.8221700704,-4.3211050903,2.2815733419

F,-13.555486667,2.0572904927,3.1404659473
F,-14.3505776627,-3.4653349953,-0.7618586225
F,-8.9352460378,1.8140292062,4.1583660498
F,-13.2292601573,-1.167342498,-1.4577093058
F,-8.4553487365,0.6326560538,1.8444289907
F,-10.7960729994,-1.1395040474,-2.9409345097
F,-8.7528532346,2.1501673352,-0.6206635174
F,-11.595455795,0.0841741111,-5.1874971402
F,-6.2376267176,2.1053387191,-1.5389571773
F,-12.4036227783,2.7056111871,-5.1229356982
F,-5.0326137992,-0.2694249649,-2.1604907561
F,-12.3720143246,4.0644222796,-2.7451657081
F,-6.4407575493,-2.6062659129,-1.8956627372
F,-11.5723462253,2.8842324956,-0.5093227274
F,-8.9538129132,-2.5954259248,-1.0505146816
H,2.6281638769,-6.0025255407,1.7776901863
H,4.4141225898,-4.2877788557,1.7610109372
H,0.2345249446,-5.3500269549,1.7522403195
H,3.8642980353,-1.8833404955,1.7444636933
H,-1.8370193895,-4.1877237725,1.714643683
H,2.8059227888,0.2284553424,1.7124650287
H,-3.9679975779,-2.292214882,-1.6704923414
H,-3.9603674382,-5.2451273052,1.7302015062
H,-4.6324993462,-4.6605993536,-1.5263176731
H,-6.3846070659,-5.7724292486,1.7179209536
H,-2.9294561497,-6.4568680185,-1.3963224208
H,-8.0795347103,-3.9681324385,1.6590852081
H,-0.5082750652,-5.9177615229,-1.4138966339
H,-7.4063096509,-1.5954684787,1.6081832372
H,-6.2467198224,0.4486743423,1.5382847415
H,-1.6109828269,4.8618484913,1.5158828998
H,-7.3011346292,2.5616301425,1.4662823031
H,-3.6676078035,6.0250299066,1.4561135618
H,-7.8468578238,4.9709125949,1.3620008647
H,-6.0577946753,6.6812622572,1.3625707142
N,-0.2963824788,-1.1594032174,1.6795551235
N,-3.2148107263,-1.0854036769,1.610244523
N,-0.2234915019,1.7639896565,1.6098132945
N,-3.1443541953,1.8332494941,1.5605725651
Au,1.7199001879,-0.3376710554,-1.6038232324
B,10.5886703754,0.0446173472,0.2943815943
C,-2.3541682937,4.9532145943,-1.7591548133
C,-3.3750208604,3.977316568,-1.7577667167
C,-1.0110552774,4.5930402802,-1.7460259025
C,-3.0717252859,2.6218066275,-1.744308465
C,-0.6987483825,3.2263656238,-1.730252528
C,-1.7197462369,2.2539373852,-1.7302905937
C,0.5603451403,2.5156870342,-1.6990328258
C,-1.0706643596,0.9621347639,-1.6993725502
C,1.8085272571,3.105963704,-1.6953137554
C,-1.7250111098,-0.2544246181,-1.6827242569
C,3.0215383212,2.4547884111,-1.65993003
C,-1.133922383,-1.5000709413,-1.6436619837
C,4.3164362399,3.1010635615,-1.6652622289
C,1.8443171007,2.7602519545,1.6184567043
C,4.6966976552,4.4481924912,-1.6980381384
C,3.2109266446,3.0651361207,1.617940836
C,6.0576378388,4.7375709677,-1.6904999179
C,3.5784582153,4.4029845626,1.5402330275
C,7.0268060997,3.7120533734,-1.6575393531
C,2.6061693946,5.4232898433,1.4623965477
C,6.654098084,2.3725388507,-1.6285153841
C,1.2464567385,5.1260585548,1.4722496843
C,5.2850519312,2.0792013873,-1.6236727915
C,0.8710004041,3.778505687,1.5564371923
C,4.5698011923,0.8209844181,-1.5883204287
C,0.4196181458,-3.1282619967,-1.5652100587
C,5.1652956407,-0.4237292077,-1.5480180007
C,1.6362294642,-3.7798706219,-1.5414487178
C,4.5106912669,-1.6381476901,-1.5389992205
C,2.8798640981,-3.1897310316,-1.541219004
C,5.1598922292,-2.9329716815,-1.5078419244
C,4.1389855717,-3.9027466794,-1.5066578224
C,6.5101962661,-3.3007060434,-1.4659470532
C,4.4442420735,-5.2681870102,-1.4547990872
C,6.8083824681,-4.657463891,-1.4088692786
C,5.7868444571,-5.6310907895,-1.4037895961
C,11.3544497114,1.4756592143,-0.0026770483
C,10.7534423095,-0.7468836318,-1.1454536807
C,10.8424271851,2.3304757745,-0.9795571425
C,12.032889751,-1.1147191479,-1.5782612209
C,11.4150703577,3.5357809243,-1.359312978
C,12.3020083729,-1.7177829712,-2.8018778065
C,12.6078608815,3.9340989195,-0.7648819906
C,11.2549401125,-1.9712252933,-3.6848627553
C,13.1890689068,3.1051814398,0.1867184463
C,9.9684009593,-1.5991247601,-3.319448919
C,12.5666629413,1.9063523739,0.5413543252
C,9.7564391072,-0.9940277481,-2.0828401898
C,11.1962303666,-0.7782562295,1.588654039
C,8.9997144557,0.216647473,0.7493847657
C,11.2045490384,-0.1498166065,2.8381695231
C,8.2311398867,-0.9403762957,0.9262450228
C,11.6057377424,-0.7582402436,4.0206974301
C,6.9257353142,-0.9499509607,1.3957792581
C,12.0151861726,-2.0882718355,3.993289736
C,6.3153143625,0.2550962667,1.716944631
C,12.0024926886,-2.7705162435,2.7835532409
C,7.0302804037,1.4331855188,1.5815802461
C,11.5904537212,-2.1160683691,1.6222124213
C,8.3475752213,1.3934259529,1.1197524999
F,9.6876302624,1.9981416079,-1.6214523802
F,13.0957460973,-0.8848650801,-0.7783766276
F,10.823076153,4.3194118753,-2.2858432236
F,13.5556810765,-2.0595543005,-3.1420901612
F,14.3516130045,3.4643401114,0.7576711425
F,8.9355071015,-1.8159216143,-4.1602731873
F,13.2299367962,1.1668377237,1.4546273179
F,8.4557005063,-0.6336682148,-1.846768382
F,10.7970689999,1.1398769479,2.9380371424
F,8.7526102446,-2.150195997,0.6184242005
F,11.5964016256,-0.0832252795,5.1849491601
F,6.2369731966,-2.10431782,1.5354648012
F,12.4038813358,-2.7048942669,5.1212084197
F,5.0322773087,0.2711226419,2.1552984473
F,12.3716883438,-4.0645175955,2.7439046733
F,6.4414572513,2.6071747947,1.8916683473
F,11.5721416275,-2.8848688847,0.5077187545
F,8.9550501495,2.595412734,1.0477340384
H,-2.6275947701,6.003639166,-1.7722977554

H,-4.413779002,4.2891075126,-1.7582884784
H,-0.2340571557,5.3508221215,-1.7454565622
H,-3.8642693509,1.8845714434,-1.7429084046
H,1.8373798309,4.1882051462,-1.707949822
H,-2.8062551839,-0.2272399755,-1.7098469031
H,3.9609699808,5.2452782419,-1.7232814005
H,3.9673471094,2.2937699619,1.6736981806
H,6.385347208,5.7720511302,-1.7142603968
H,4.6312770536,4.662304457,1.5304089581
H,8.0799825416,3.9673285421,-1.6610517472
H,2.9278197932,6.4583379887,1.402110655
H,7.4063785363,1.5947270648,-1.6127900793
H,0.5067583056,5.9187348958,1.4203821629
H,6.2464540384,-0.4492292287,-1.5423227306
H,1.609757276,-4.8613672055,-1.5098622423
H,7.3004652639,-2.5622033837,-1.4710717982
H,3.6662951217,-6.0249215891,-1.45055109
H,7.8459331952,-4.9715685969,-1.3651111429
H,6.0565313143,-6.6815301086,-1.360513114
N,0.2962665791,1.1600907706,-1.6756057499
N,3.2147363341,1.0855266579,-1.6081935285
N,0.2228634051,-1.7632855072,-1.6057693376
N,3.1437354266,-1.8331349927,-1.5586298384

[S9] M. J. Frisch, G. W. Trucks, H. B. Schlegel, G. E. Scuseria, M. A. Robb, J. R. Cheeseman, G. Scalmani, V. Barone, G. A. Petersson, H. Nakatsuji, X. Li, M. Caricato, A. V. Marenich, J. Bloino, B. G. Janesko, R. Gomperts, B. Mennucci, H. P. Hratchian, J. V. Ortiz, A. F. Izmaylov, J. L. Sonnenberg, D. Williams-Young, F. Ding, F. Lipparini, F. Egidi, J. Goings, B. Peng, A. Petrone, T. Henderson, D. Ranasinghe, V. G. Zakrzewski, J. Gao, N. Rega, G. Zheng, W. Liang, M. Hada, M. Ehara, K. Toyota, R. Fukuda, J. Hasegawa, M. Ishida, T. Nakajima, Y. Honda, O. Kitao, H. Nakai, T. Vreven, K. Throssell, J. A. Montgomery, Jr., J. E. Peralta, F. Ogliaro, M. J. Bearpark, J. J. Heyd, E. N. Brothers, K. N. Kudin, V. N. Staroverov, T. A. Keith, R. Kobayashi, J. Normand, K. Raghavachari, A. P. Rendell, J. C. Burant, S. S. Iyengar, J. Tomasi, M. Cossi, J. M. Millam, M. Klene, C. Adamo, R. Cammi, J. W. Ochterski, R. L. Martin, K. Morokuma, O. Farkas, J. B. Foresman and D. J. Fox, *Gaussian 16*, Revision C.01, Gaussian, Inc., Wallingford CT, 2016.

[S10] J. Wang, R. M. Wolf, J. W. Caldwell, P. A. Kollman and D. A. Case, *J. Comput. Chem.*, 2004, **25**, 1157–1174.

[S11] J. Yoshida, S. Tamura, K. Hoshino, H. Yuge, H. Sato, A. Yamazaki, S. Yoneda and G. Watanabe, *J. Phys. Chem. B*, 2018, **122**, 10615–10626.

[S12] G. Watanabe, H. Watanabe, K. Suzuki, H. Yuge, S. Yoshida, T. Mandai, S. Yoneda, H. Sato, M. Hara and J. Yoshida, *Chem. Commun.*, 2020, **56**, 12134–12137.

[S13] X. Wu, Z. Liu, S. Huang and W. Wang, *Phys. Chem. Chem. Phys.*, 2005, **7**, 2771–2779.

[S14] X. Dong, X. Yuan, Z. Song and Q. Wang, *Phys. Chem. Chem. Phys.*, 2021, **23**, 12582–12591.

[S15] A. P. Scott and L. Radom, *J. Phys. Chem.*, 1996, **100**, 16502–16513.

[S16] A. K. Rappe, C. J. Casewit, K. S. Colwell, W. A. Goddard III and W. M. Skiff, *J. Am. Chem. Soc.*, 1992, **114**, 10024–10035.

[S17] C. I. Bayly, P. Cieplak, W. Cornell and P. A. Kollman, *J. Phys. Chem.*, 1993, **97**, 10269–10280.

[S18] H. J. C. Berendsen, J. P. M. Postma, W. F. van Gunsteren, A. Dinola and J. R. Haak, *J. Chem. Phys.*, 1984, **81**, 3684–3690.

[S19] S. Nosé, *Mol. Phys.*, 1983, **52**, 255–268.

[S20] M. Parrinello and A. Rahman, *J. Appl. Phys.*, 1981, **52**, 7182–7190.

[S21] B. Hess, H. Bekker, H. J. C. Berendsen and J. G. E. M. Fraaije, *J. Comput. Chem.*, 1997, **18**, 1463–1472.

[S22] Z. Chen, C. S. Wannere, C. Corminboeuf, R. Puchta and P. v. R. Schleyer, *Chem. Rev.*, 2005, **105**, 3842–3888.

[S23] T. Lu and F. Chen, *J. Comput. Chem.*, 2012, **33**, 580–592.

[S24] R. Herges and D. Geuenich, *J. Phys. Chem. A*, 2001, **105**, 3214–3220.

[S25] M. J. S. Phipps, T. Fox, C. S. Tautermann and C.-K. Skylaris, *Chem. Soc. Rev.*, 2015, **44**, 3177–3211.

[S26] Articles for *GAMESS*: (a) M. W. Schmidt, K. K. Baldridge, J. A. Boatz, S. T. Elbert, M. S. Gordon, J. H. Jensen, S. Koseki, N. Matsunaga, K. A. Nguyen, S. Su, T. L. Windus, M. Dupuis and J. A. Montgomery, *J. Comput. Chem.*, 1993, **14**, 1347−1363; (b) M. S. Gordon and M. W. Schmidt, In *Theory and Applications of Computational Chemistry: The First Forty Years*, ed: C. E. Dykstra, G. Frenking, K. S. Kim and G. E. Scuseria, Elsevier, 2005; pp. 1167–1189.

[S27] Report for FMO: K. Kitaura, E. Ikeo, T. Asada, T. Nakano and M. Uebayasi, *Chem. Phys. Lett.*, 1999, **313**, 701–706.

[S28] Report for pair interaction energy decomposition analysis (PIEDA): D. G. Fedorov and K. Kitaura, *J. Comput. Chem.*, 2007, **28**, 222–237.

[S29] Program and report for calculation of transfer integrals: (a) ADF (2024.1), SCM, Theoretical Chemistry, Vrije Universiteit, Amsterdam, 2024; (b) G. te Velde, F. M. Bickelhaupt, E. J. Baerends, C. Fonseca Guerra, S. J. A. van Gisbergen, J. G. Snijders and T. Ziegler. *J. Comput. Chem.*, 2001, **22**, 931–967.

[S30] T. Mori, A. Kobayashi, Y. Sasaki, H. Kobayashi, G. Saito and H. Inokuchi, *Bull. Chem. Soc. Jpn.*, 1984, **57**, 627–633.

4. Solution-state assembled behaviors

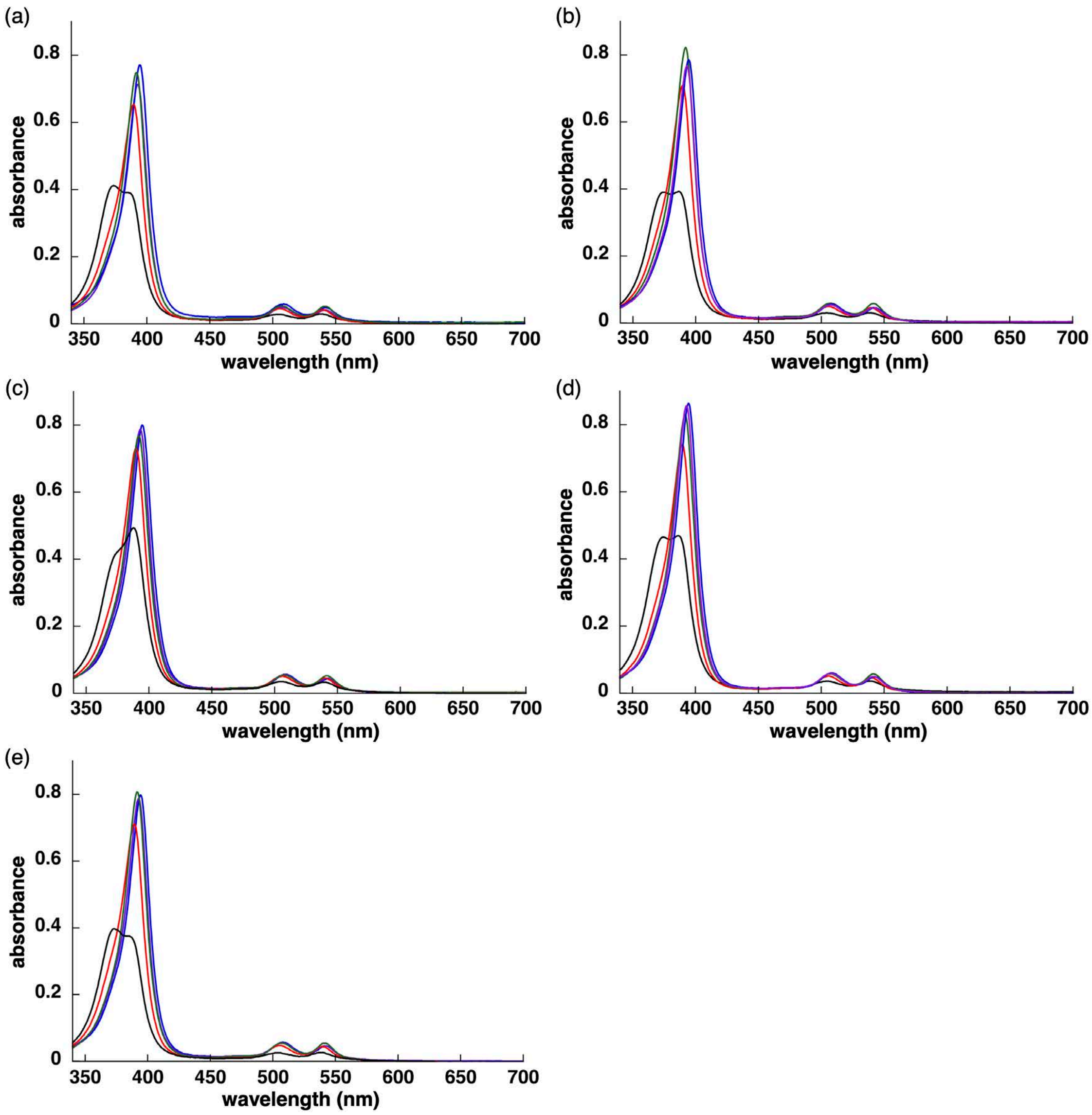

Fig. S67 UV/vis absorption spectra of (a) **2au**$^+$-Cl$^-$, (b) **2au**$^+$-PF_6^-, (c) **2au**$^+$-FABA$^-$, (d) **2au**$^+$-BArF$^-$, and (e) **2au**$^+$-PCCp$^-$ in several solvents (4 μM): CH_3CN (black), acetone (red), CH_2Cl_2 (green), DMSO (blue), and DMF (purple). In CH_3CN, the Soret band was blue-shifted, suggesting the dimer formation.

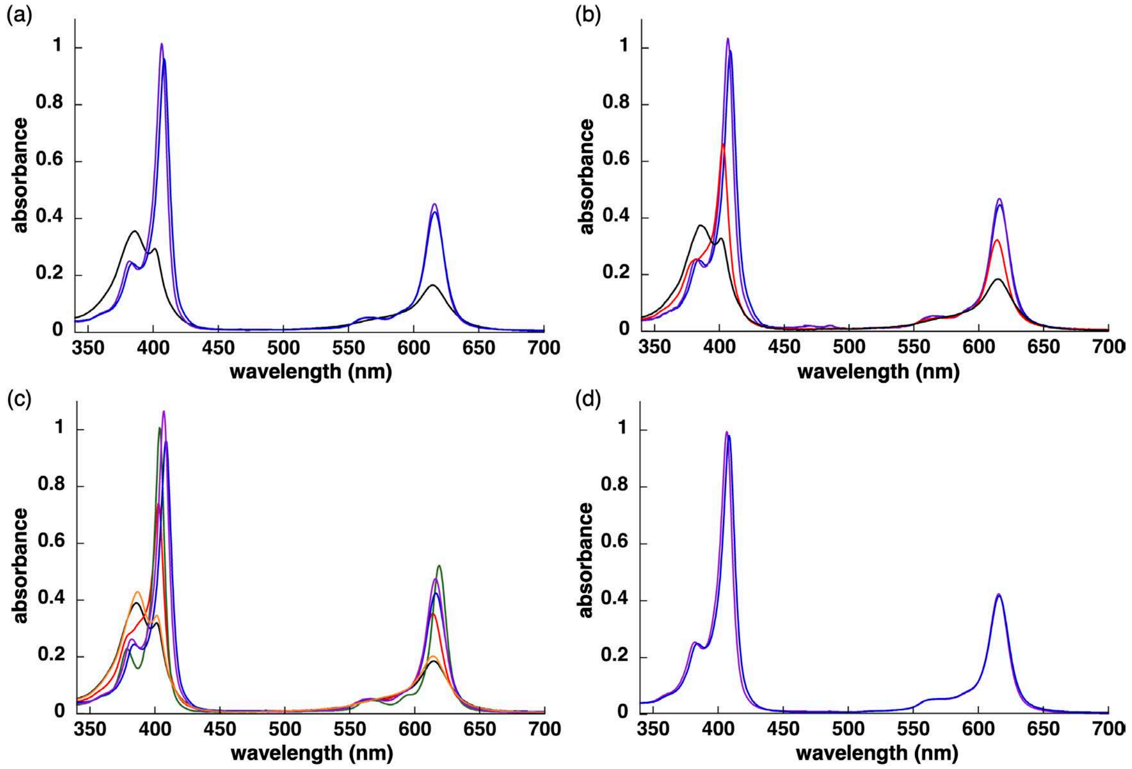

Fig. S68 UV/vis absorption spectra of (a) **1au**$^+$-PF_6^-, (b) **1au**$^+$-FABA$^-$, (c) **1au**$^+$-BArF$^-$, and (d) **1au**$^+$-PCCp$^-$ in several solvents (4 μM): CH_3CN (black), acetone (red), DMSO (blue), DMF (purple), MeOH (orange), and CH_2Cl_2 (green). In CH_3CN, the Soret band was blue-shifted, suggesting the dimer formation. **1au**$^+$-BArF$^-$ showed moderate solubility even in less polar CH_2Cl_2.

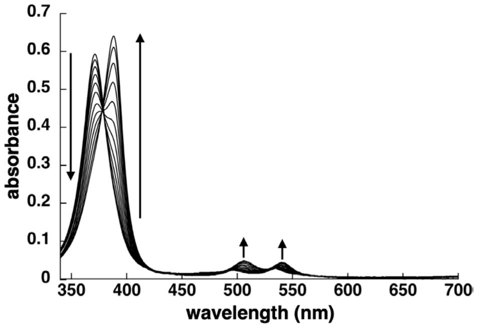

Fig. S69 VT UV/vis absorption spectra of **2au**$^+$-FABA$^-$ in CH_3CN from –40 °C to 70 °C. Red shift of the Soret band from 371 nm to 388 nm by heating suggests the disaggregation of the stacked dimer.

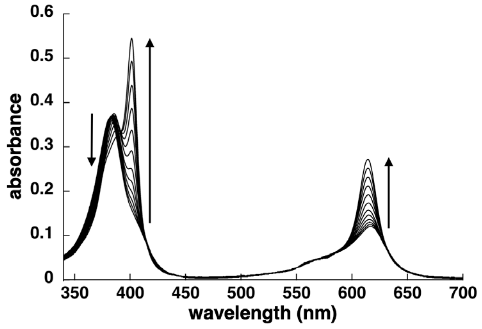

Fig. S70 VT UV/vis absorption spectra of **1au**$^+$-FABA$^-$ in CH_3CN from –40 °C to 70 °C. Red shift of the Soret band from 382 nm to 401 nm by heating suggests the disaggregation of the stacked dimers.

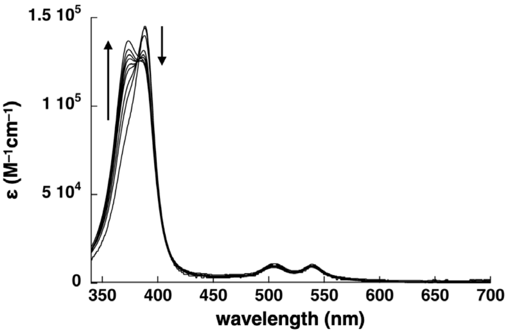

Fig. S71 Concentration-dependent UV/vis absorption spectra of **2au**$^+$-FABA$^-$ at 1.0×10^{-6} to 1.0×10^{-5} M in CH_3CN at 20 °C. Blue shift of the Soret band from 388 nm to 373 nm suggests the formation of the stacked **2au**$^+$ dimer at higher concentration.

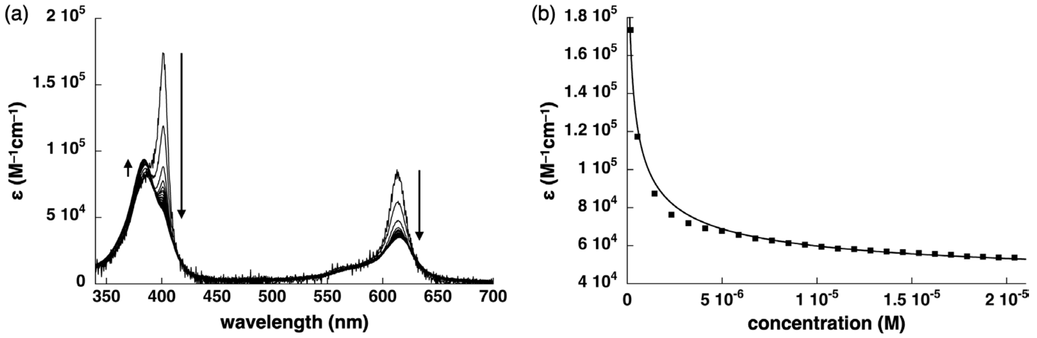

Fig. S72 Concentration-dependent UV/vis absorption spectra of **1au**$^+$-FABA$^-$ at (a) 1.8×10^{-7} to 2.0×10^{-5} M in CH_3CN at 20 °C and (b) concentration-dependent plots of the ε ($M^{-1}cm^{-1}$) at 402 nm and fitting curves. Blue shift of the Soret band from 402 nm to 384 nm suggests the formation of the stacked **1au**$^+$ dimer at higher concentration. Assuming that only isolated monomer and stacked dimer exist, dimerization constants were estimated by least-square curve fitting using the following formula

$$\varepsilon_{obs} = \varepsilon_{mono} + \frac{1 + 4K_{dim}C - \sqrt{1 + 8K_{dim}C}}{4K_{dim}C}(\varepsilon_{dim} - \varepsilon_{mono})$$

for the equilibrium between the monomer and the dimer showing their summed absorptions (ε_{obs}: observed ε values, ε_{mono}: ideal monomer ε, ε_{dim}: ideal dimer ε, K_{dim}: dimerization constant, C: total concentrations of the ion pair). Fitting curves are consistent with the formation of the stacked dimer. The K_{dim} was roughly estimated to be 5×10^6 M^{-1}, which is higher than that of phthalocyanine Cu^{II} complexes bearing alkyl side chains in *n*-dodecane ($K_{dim} = 1.5 \times 10^6$ M^{-1}).[S31]

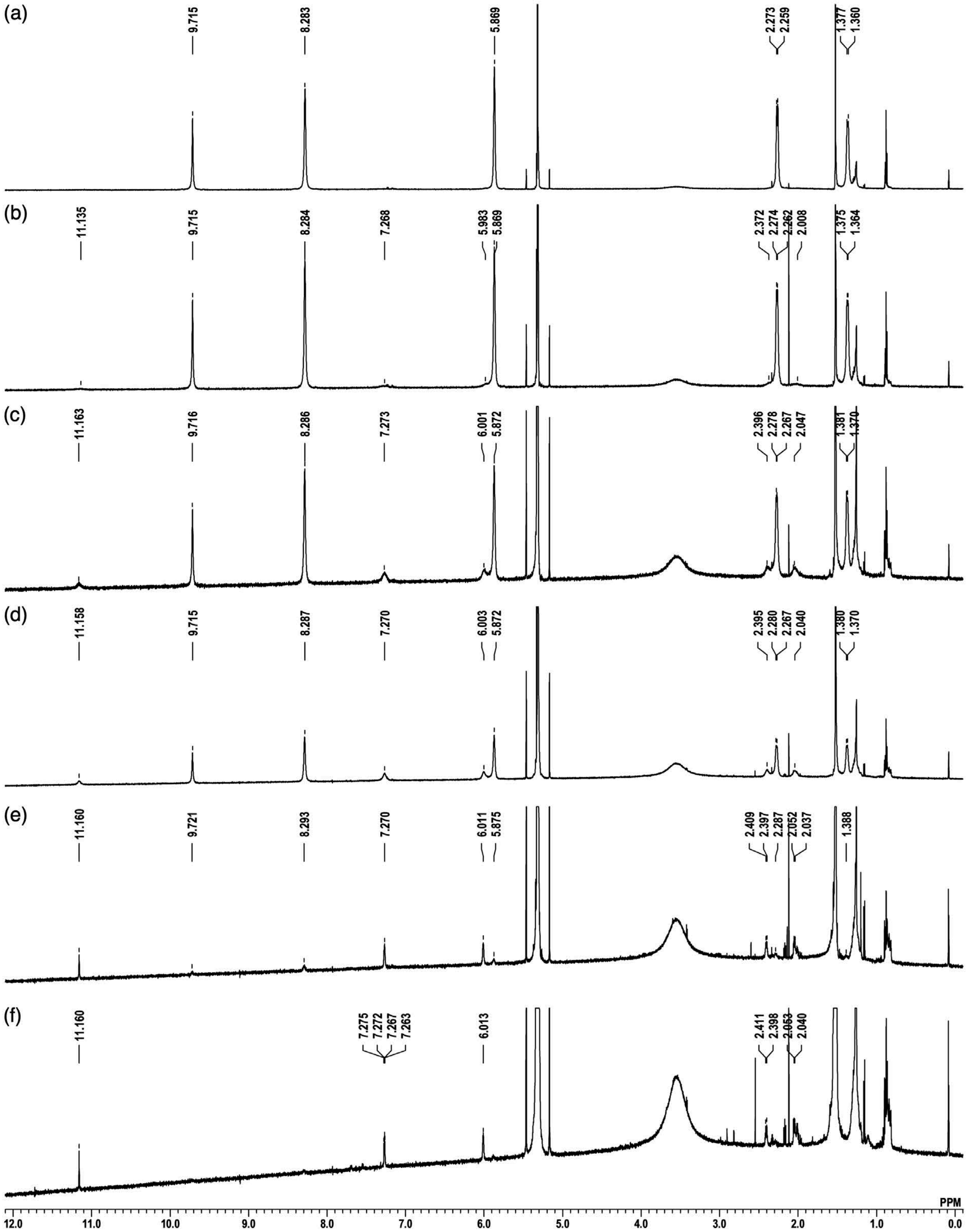

Fig. S73 Variable concentration ^{1}H NMR of **2au**$^{+}$-FABA^{-} at (a) 1 mM, (b) 0.5 mM, (c) 0.1 mM, (d) 0.05 mM, (e) 0.01 mM, and (f) 5 μM in CD_2Cl_2 at 20 °C. The dimerization constant (K_{dim}) for **2au**$^{+}$ was estimated to be 1.1 × 10^{5} M^{-1} from the monomer/dimer ratio in (d).

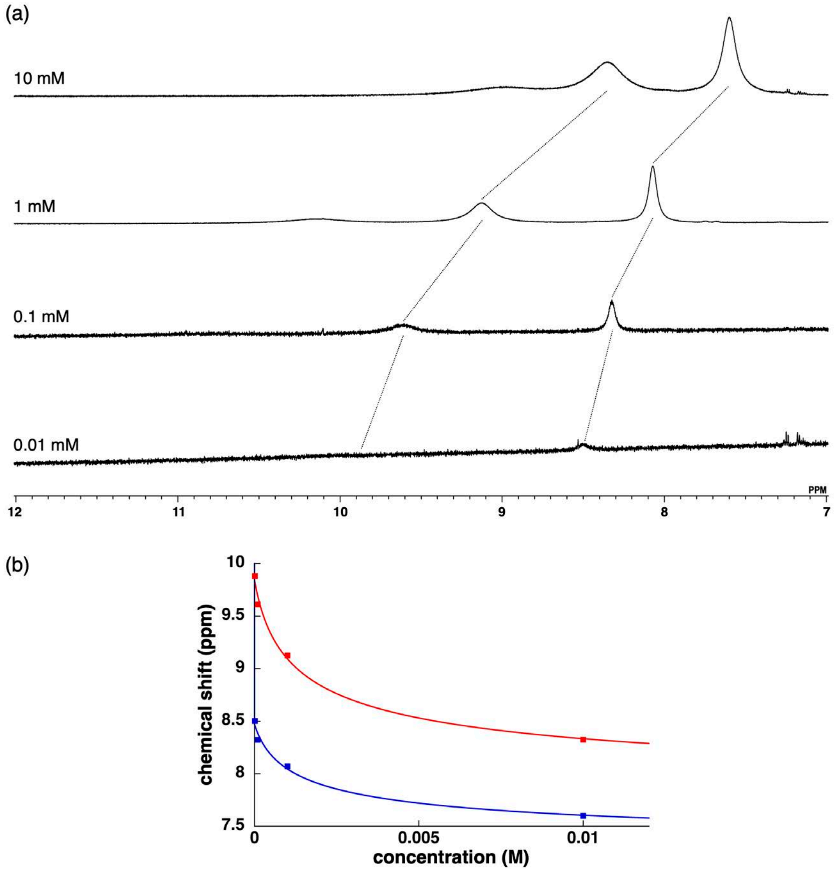

Fig. S74 Concentration-dependent ^{1}H NMR of **1au**$^+$-FABA$^-$ at (a) 10–0.01 mM in DMSO-d_6 at 20 °C and (b) concentration-dependent plots of the ^{1}H NMR chemical shifts and fitting curves (red and blue plots correspond to the benzo-CH). The upfield shift of the benzo-CHs at higher concentrations suggest the dimerization **1au**$^+$. These shifts are attributed to the effect of the ring current of the neighbor molecule in a stacked dimer. Assuming that only isolated monomer and stacked dimer exist, dimerization constants were estimated by least-square curve fitting using the following formula

$$\delta_{obs} = \delta_{mono} + \frac{1 + 4K_{dim}C - \sqrt{1 + 8K_{dim}C}}{4K_{dim}C}(\delta_{dim} - \delta_{mono})$$

for the equilibrium between the monomer and the dimer showing their averaged signals (δ_{obs}: observed chemical shifts, δ_{mono}: ideal monomer chemical shift, δ_{dim}: ideal dimer chemical shift, K_{dim}: dimerization constant, C: total concentrations of the ion pair). Fitting curves are consistent with the formation of the stacked dimer. The K_{dim} was roughly estimated to be 400 M^{-1}, which is higher than phthalocyanine Cu^{II} complexes bearing alkyl side chains in THF as a polar solvent (K_{dim} = 140 M^{-1}).[S31]

[S31] A. R. Monahan, J. A. Brado and A. F. DeLuca, *J. Phys. Chem.*, 1972, **76**, 446–449.

5. Solid-state assembled behaviors

Absorption measurements of single crystals. An inverted optical microscope (IX71, Olympus) was used for steady-state absorption measurements of single crystals. The halogen lamp placed above the sample stage was employed as the incident light, and focused on the sample microcrystal with the objective lens (×40, NA 0.40). The transmitted light was collected with the objective lens (×60, NA 0.70), and then passed through a confocal pinhole (1 µm in diameter) to select the monitoring position. The intensity was detected with a CCD camera (DU970P, Andor) coupled with a polychrometer (250is, Chromex). The absorption spectra were calculated by using reference light, transmitted only through the coverslip.

Differential scanning calorimetry (DSC). The phase transitions were measured on a differential scanning calorimetry (Shimadzu DSC-60).

Synchrotron X-ray diffraction analysis (XRD). High-resolution XRD analyses were carried out using a synchrotron radiation X-ray beam with a wavelength of 1.00 Å on BL40B2 at SPring-8 (Hyogo, Japan). A DECTRIS PILATUS3S 2M with camera lengths of 427.4 mm for **1au**$^+$-FABA^-_P, 429.7 mm for **1au**$^+$-FABA^-_H, and 428.1 mm for **1au**$^+$-BArF$^-$ and **1au**$^+$-PCCp$^-$. An exposure time of the X-ray beam was 10 sec.

Solid-state nuclear magnetic resonance (SSNMR). Solid-state NMR measurements were conducted using JEOL JNM-ECZ800WB with spinning frequency of 20 kHz at r.t.

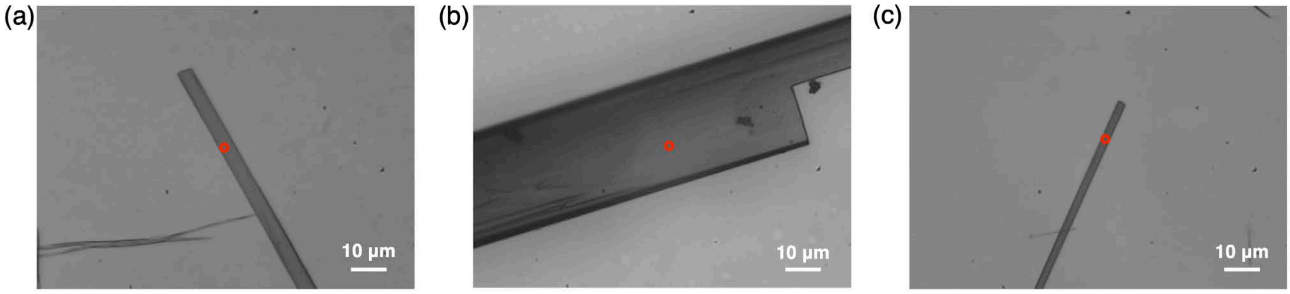

Fig. S75 Optical microscopic image of single crystal of (a) **1au**$^+$-FABA$^-$, (b) **1au**$^+$-BArF$^-$, and (c) **1au**$^+$-PCCp$^-$ obtained by recrystallization from $CH_3CN/CHCl_3$, 1,1,1-trichloroethane/*n*-heptane, and *o*-dichlorobenzene/DMF, respectively. Red spots indicate the position where the UV/vis absorption measurements were conducted (Fig. S76).

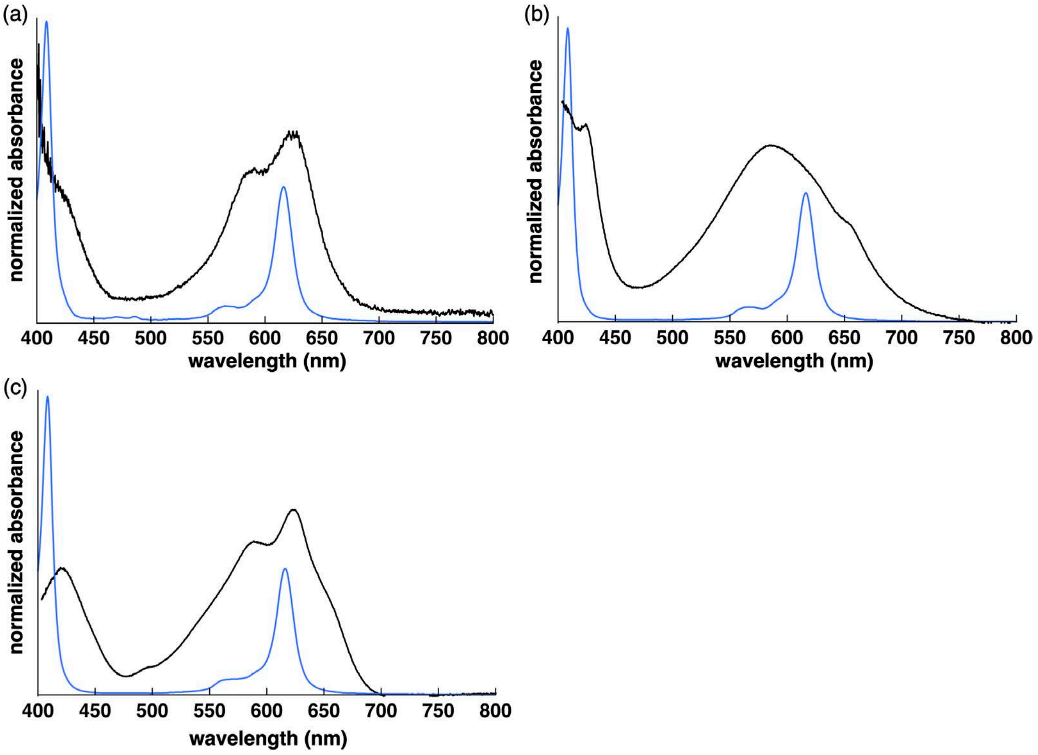

Fig. S76 UV/vis absorption spectra of (a) **1au**$^+$-FABA$^-$, (b) **1au**$^+$-BArF$^-$, and (c) **1au**$^+$-PCCp$^-$ in the form of a single crystal (black) and in DMSO (4 μM) (blue) (Fig. S68). UV/vis absorption spectrum of the single crystal of **1au**$^+$-FABA$^-$, with charge-segregated assembly, showed broad absorption bands with the λ_{max} at 587 and 623 nm. These bands are blue- and red-shifted absorption compared to the absorption band of the monomer at 616 nm in DMSO. Similarly, **1au**$^+$-PCCp$^-$ showed broad absorption bands at 588 and 623 nm. These absorption maxima in the solid state are ascribable to the exciton coupling in J-like arrangement and very weak coupling for the orthogonally arranged transition dipole moments (Fig. S62). On the other hand, **1au**$^+$-BArF$^-$ showed a broad absorption band at 585 nm with a shoulder at 654 nm, the former of which is blue-shifted compared to the absorption band of the monomer at 616 nm in DMSO. The blue-shifted band can be ascribed to H-like arrangement in the stacked **1au**$^+$ (Fig. S62).

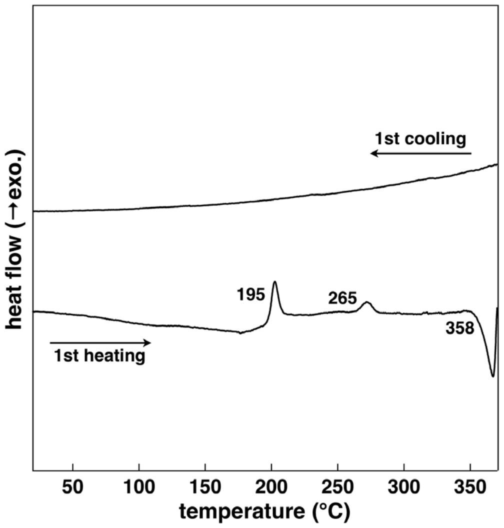

Fig. S77 DSC thermogram of **1au**$^+$-FABA$^-{}_H$ obtained by heating **2au**$^+$-FABA$^-$ at 190 °C without precipitation step. Onset temperatures (°C) of phase transitions are represented. The Col$_h$ structure, maintained up to 195 °C, was converted to the complicated crystalline state at higher temperature with the decomposition at 358 °C. Although the packing structure at >195 °C could not be determined, the metastable Col$_h$ state is changed to the stable crystalline state. No peaks were observed upon cooling after heating at >358 °C because of the decomposition of the ion pair. DSC thermogram for **1au**$^+$-BArF$^-$ obtained by heating **2au**$^+$-BArF$^-$ is not shown here because the conversion from **2au**$^+$-BArF$^-$ to **1au**$^+$-BArF$^-$ at 180 °C induces the complicated crystalline state as revealed by XRD analysis (Fig. S84).

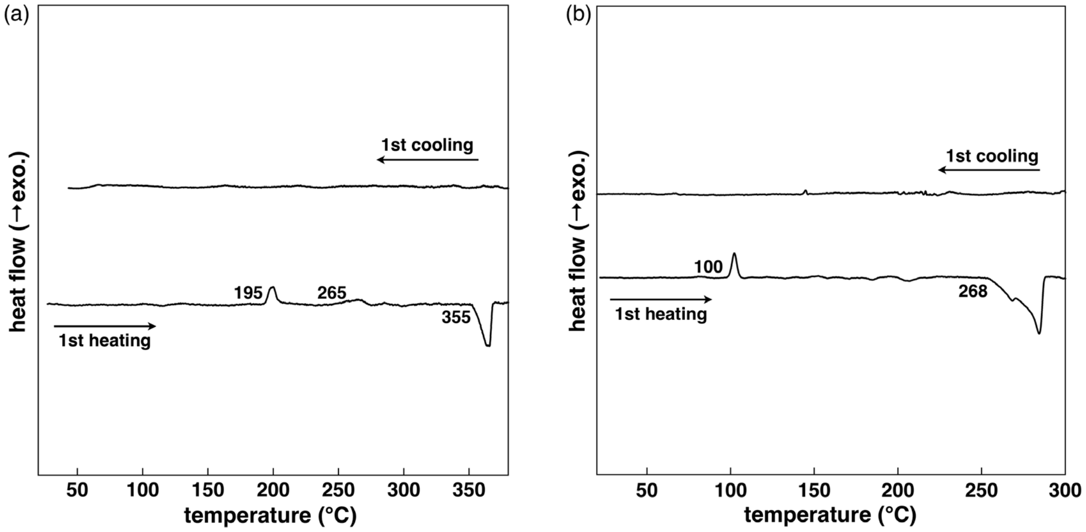

Fig. S78 DSC thermogram of (a) **1au**$^+$-FABA^-_P and (b) **1au**$^+$-BArF^-_P as precipitates prepared from acetone/*n*-hexane. Onset temperatures (°C) of phase transitions are represented. The peaks at 195 and 100 °C for **1au**$^+$-FABA$^-$ and **1au**$^+$-BArF$^-$, respectively, indicate the transformation to complicated crystalline states as revealed by XRD analysis (Fig. S81,84). The transitions at 355 and 268 °C for **1au**$^+$-FABA^-_P and **1au**$^+$-BArF^-_P, respectively, suggest the decomposition of the ion pairs.

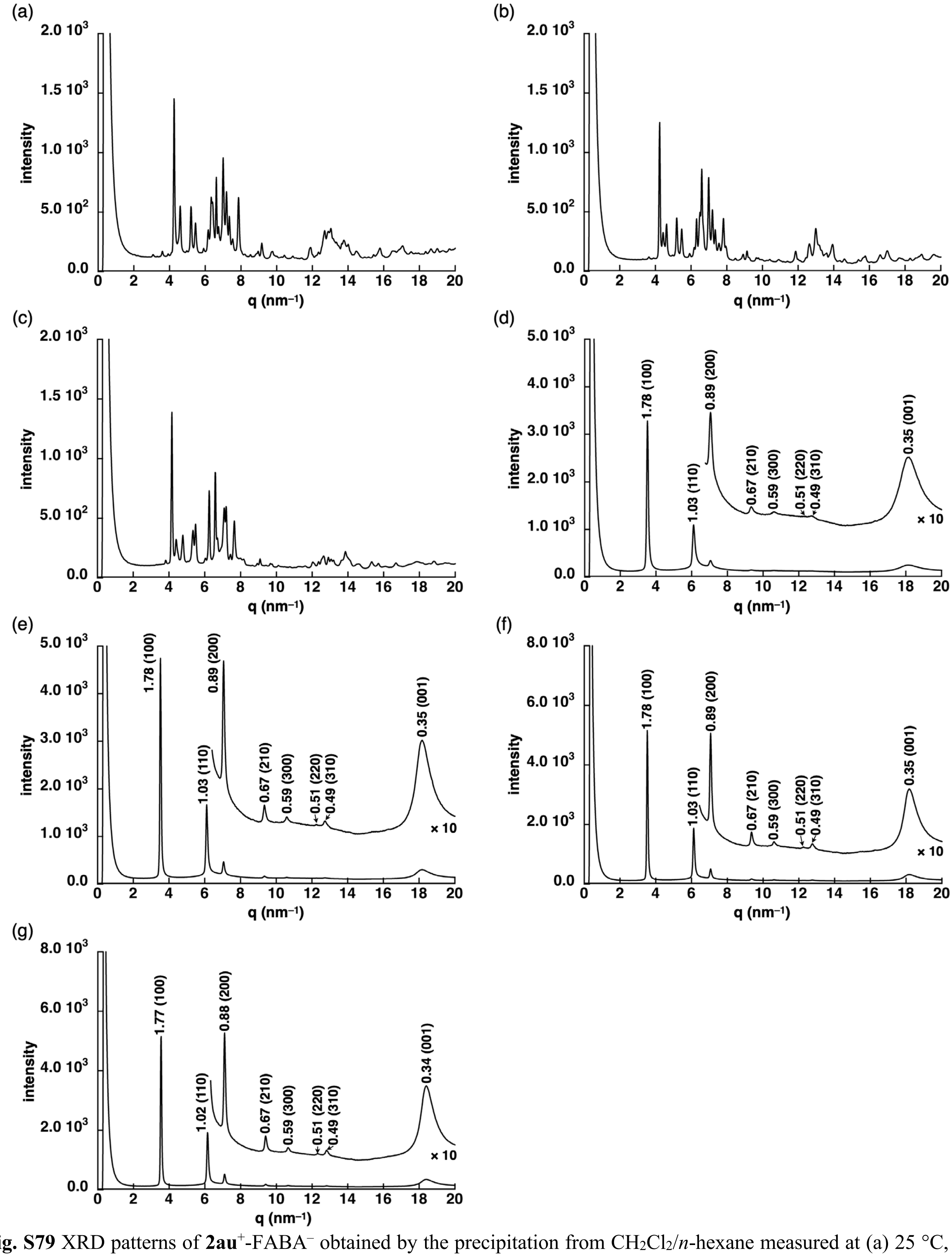

Fig. S79 XRD patterns of **2au**$^+$-FABA$^-$ obtained by the precipitation from CH_2Cl_2/*n*-hexane measured at (a) 25 °C, (b) 140 °C, (c) 150 °C, (d) 150 °C (after maintaining for 30 min), (e) 160 °C, and (f) 160 °C (after maintaining for 30 min) upon heating and (g) 25 °C upon cooling. At 150 °C, conversion from **2au**$^+$-FABA$^-$ to **1au**$^+$-FABA^-_H was observed. Diffraction pattern of the Col$_h$ packing appeared at 25 °C was identical to that of **1au**$^+$-FABA^-_P obtained by precipitation from acetone/*n*-hexane (Fig. S81).

Table S7 Summary of XRD data of **1au**$^+$-FABA^-_H obtained by heating of **2au**$^+$-FABA$^-$ (Fig. S79). The peaks which can be indexed are represented.

	q (nm^{-1})	*d*-spacing (nm)	ratio	ratio (calc.)	*hkl*
	3.52	1.78	1.000	1.000	100
	6.11	1.03	0.577	0.577	110
(d) 150 °C (after 30 min)	7.05	0.89	0.500	0.500	200
Col_h	9.33	0.67	0.377	0.378	210
a = 2.06 nm, c = 0.35 nm	10.62	0.59	0.332	0.333	300
Z = 1 (ρ = 1.81)	12.23	0.51	0.288	0.289	220
	12.75	0.49	0.276	0.277	310
	18.19	0.35	–	–	001
	3.52	1.78	1.000	1.000	100
	6.11	1.03	0.577	0.577	110
(e) 160 °C	7.05	0.89	0.500	0.500	200
Col_h	9.33	0.67	0.377	0.378	210
a = 2.06 nm, c = 0.35 nm	10.59	0.59	0.333	0.333	300
Z = 1 (ρ = 1.81)	12.24	0.51	0.288	0.289	220
	12.75	0.49	0.276	0.277	310
	18.16	0.35	–	–	001
	3.52	1.78	1.000	1.000	100
	6.12	1.03	0.576	0.577	110
(f) 160 °C (after 30 min)	7.07	0.89	0.499	0.500	200
Col_h	9.35	0.67	0.377	0.378	210
a = 2.06 nm, c = 0.35 nm	10.61	0.59	0.332	0.333	300
Z = 1 (ρ = 1.81)	12.25	0.51	0.288	0.289	220
	12.75	0.49	0.276	0.277	310
	18.18	0.35	–	–	001
	3.55	1.77	1.000	1.000	100
	6.15	1.02	0.577	0.577	110
(g) 25 °C	7.10	0.88	0.500	0.500	200
Col_h	9.40	0.67	0.378	0.378	210
a = 2.04 nm, c = 0.34 nm	10.66	0.59	0.333	0.333	300
Z = 1 (ρ = 1.86)	12.31	0.51	0.288	0.289	220
	12.81	0.49	0.277	0.277	310
	18.39	0.34	–	–	001

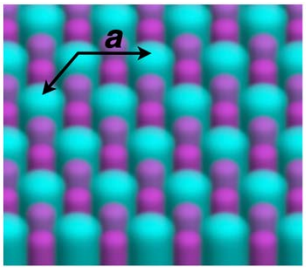

Fig. S80 Possible packing model of **1au**$^+$-FABA^-_H as a Col_h structure. **1au**$^+$ and FABA$^-$ are represented in cyan and magenta, respectively.

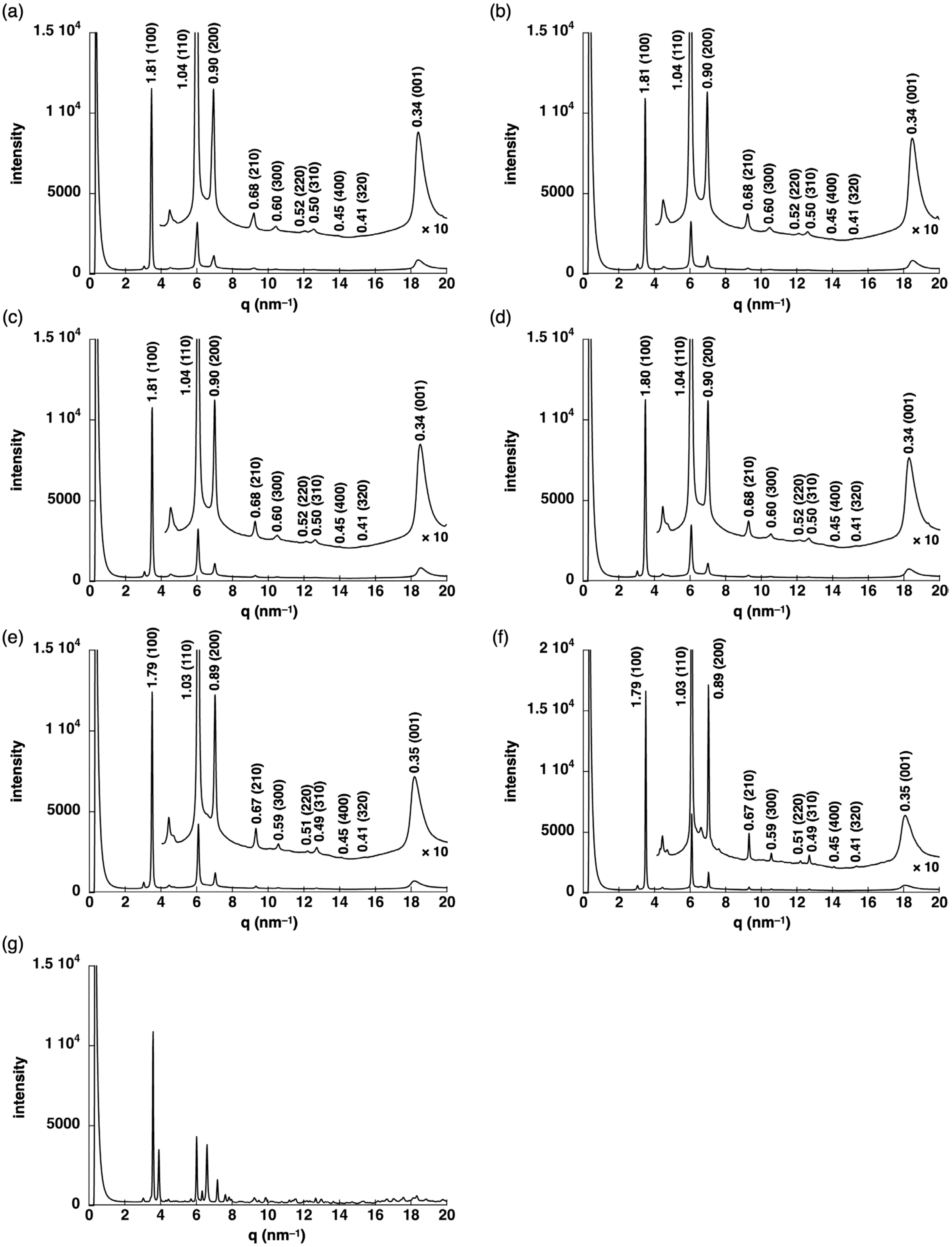

Fig. S81 XRD patterns of **1au**$^{+}$-FABA^{-}$_{\mathrm{P}}$ obtained by precipitation from acetone/*n*-hexane at (a) 25 °C, (b) 30 °C, (c) 60 °C, (d) 100 °C, (e) 150 °C, (f) 190 °C, and (g) 230 °C upon heating. In (a)–(f), some small diffraction peaks could be derived from minor species that was formed through the precipitation process.

Table S8 Summary of XRD data of **1au**$^+$-FABA^-_P (Fig. S81). The peaks which can be indexed are represented.

	q (nm^{-1})	*d*-spacing (nm)	ratio	ratio (calc.)	*hkl*
(a) 25 °C<br>Col$_h$<br>a = 2.09 nm, c = 0.34 nm<br>Z = 1 (ρ = 1.79)	3.47	1.81	1.000	1.000	100
	6.03	1.04	0.575	0.577	110
	6.96	0.90	0.497	0.500	200
	9.21	0.68	0.376	0.378	210
	10.45	0.60	0.331	0.333	300
	12.10	0.52	0.287	0.289	220
	12.58	0.50	0.276	0.277	310
	13.92	0.45	0.249	0.250	400
	15.16	0.41	0.227	0.229	320
	18.41	0.34	–	–	001
(b) 30 °C<br>Col$_h$<br>a = 2.08 nm, c = 0.34 nm<br>Z = 1 (ρ = 1.81)	3.48	1.80	1.000	1.000	100
	6.05	1.04	0.578	0.577	110
	7.00	0.90	0.500	0.500	200
	9.26	0.68	0.378	0.378	210
	10.52	0.60	0.333	0.333	300
	12.14	0.52	0.289	0.289	220
	12.63	0.50	0.278	0.277	310
	13.96	0.45	0.249	0.250	400
	15.30	0.41	0.227	0.229	320
	18.50	0.34	–	–	001
(c) 60 °C<br>Col$_h$<br>a = 2.08 nm, c = 0.34 nm<br>Z = 1 (ρ = 1.81)	3.49	1.80	1.000	1.000	100
	6.06	1.04	0.578	0.577	110
	7.00	0.90	0.500	0.500	200
	9.28	0.68	0.378	0.378	210
	10.52	0.60	0.333	0.333	300
	12.16	0.52	0.289	0.289	220
	12.65	0.50	0.278	0.277	310
	13.99	0.45	0.249	0.250	400
	15.30	0.41	0.227	0.229	320
	18.53	0.34	–	–	001
(d) 100 °C<br>Col$_h$<br>a = 2.08 nm, c = 0.34 nm<br>Z = 1 (ρ = 1.81)	3.49	1.80	1.000	1.000	100
	6.06	1.04	0.578	0.577	110
	7.01	0.90	0.500	0.500	200
	9.28	0.68	0.378	0.378	210
	10.53	0.60	0.333	0.333	300
	12.16	0.52	0.289	0.289	220
	12.66	0.50	0.278	0.277	310
	13.93	0.45	0.249	0.250	400
	15.28	0.41	0.227	0.229	320
	18.28	0.34	–	–	001
(e) 150 °C<br>Col$_h$<br>a = 2.07 nm, c = 0.35 nm<br>Z = 1 (ρ = 1.77)	3.51	1.79	1.000	1.000	100
	6.10	1.03	0.575	0.577	110
	7.04	0.89	0.497	0.500	200
	9.32	0.67	0.374	0.378	210
	10.58	0.59	0.330	0.333	300
	12.22	0.51	0.285	0.289	220
	12.72	0.49	0.274	0.277	310
	14.09	0.45	0.249	0.250	400
	15.39	0.41	0.227	0.229	320
	18.17	0.35	–	–	001

Table S8 (Continued)

	q (nm^{-1})	*d*-spacing (nm)	ratio	ratio (calc.)	*hkl*
	3.51	1.79	1.000	1.000	100
	6.09	1.03	0.575	0.577	110
	7.03	0.89	0.497	0.500	200
(f) 190 °C	9.31	0.67	0.374	0.378	210
Col_h	10.57	0.59	0.330	0.333	300
a = 2.07 nm, c = 0.35 nm	12.20	0.51	0.285	0.289	220
Z = 1 (ρ = 1.77)	12.70	0.49	0.274	0.277	310
	14.08	0.45	0.249	0.250	400
	15.35	0.41	0.227	0.229	320
	18.08	0.35	–	–	001

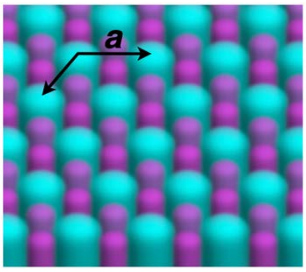

Fig. S82 Possible packing model of **1au**$^+$-FABA^-_P as a Col_h structure. **1au**$^+$ and FABA$^-$ are represented in cyan and magenta, respectively.

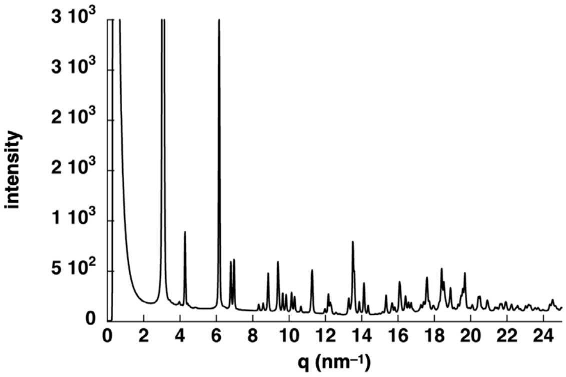

Fig. S83 XRD pattern of **1au**$^+$-FABA$^-$ obtained by precipitation from $CH_3CN/CHCl_3$ at 25 °C. The XRD pattern is identical to that of the single crystal of **1au**$^+$-FABA$^-$ (Fig. S20).

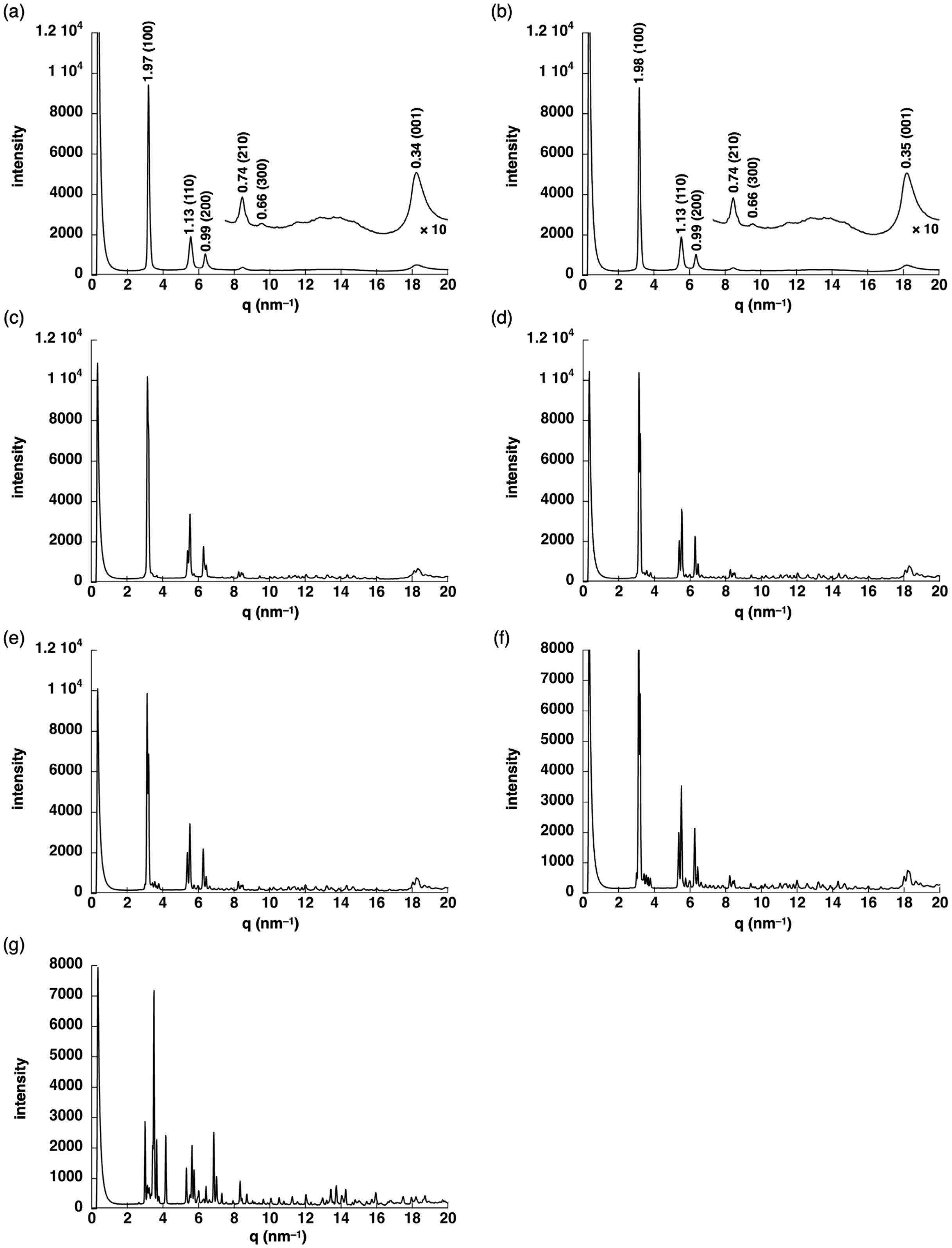

Fig. S84 XRD patterns of **1au**$^+$-BArF^-_P obtained by precipitation from acetone/*n*-hexane at (a) 25 °C, (b) 60 °C, (c) 100 °C, (d) 120 °C, (e) 150 °C, (f) 170 °C, and (g) 200 °C upon heating. In (a) and (b), small diffraction peaks could be derived from higher crystallinity compared to the Col$_h$ of **1au**$^+$-FABA$^-$ (Fig. S81).

Table S9 Summary of XRD data of **1au**$^+$-BArF$^-$ (Fig. S84). The peaks which can be indexed are represented.

	q (nm^{-1})	*d*-spacing (nm)	ratio	ratio (calc.)	*hkl*
	3.18	1.97	1.000	1.000	100
(a) 25 °C	5.56	1.13	0.573	0.577	110
Col$_h$	6.37	0.99	0.503	0.500	200
a = 2.27 nm, c = 0.34 nm	8.48	0.74	0.376	0.378	210
Z = 1 (ρ = 1.71)	9.58	0.66	0.335	0.333	300
	18.24	0.34	–	–	001
	3.17	1.98	1.000	1.000	100
(b) 60 °C	5.54	1.13	0.571	0.577	110
Col$_h$	6.36	0.99	0.500	0.500	200
a = 2.29 nm, c = 0.35 nm	8.46	0.74	0.374	0.378	210
Z = 1 (ρ = 1.64)	9.58	0.66	0.333	0.333	300
	18.19	0.35	–	–	001

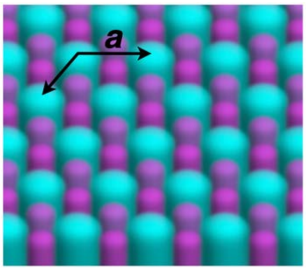

Fig. S85 Possible packing model of **1au**$^+$-BArF$^-$ as a Col$_h$ structure. **1au**$^+$ and BArF$^-$ are represented in cyan and magenta, respectively.

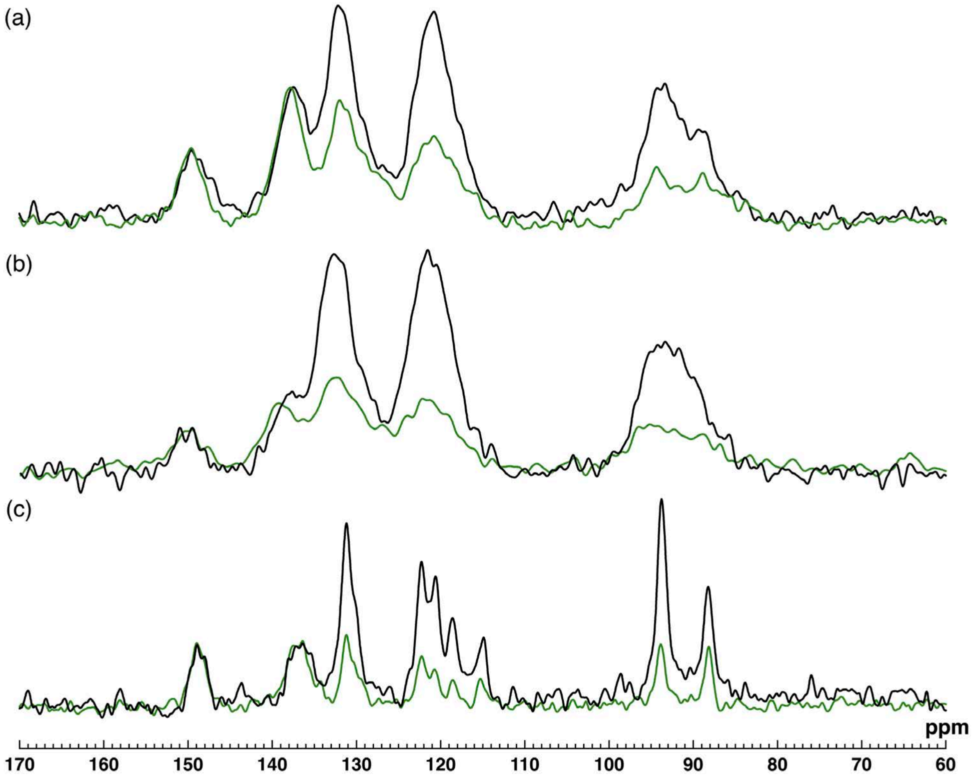

Fig. S86 ^{13}C cross-polarization magic-angle spinning (CP-MAS) NMR spectra (black lines) and dipolar-dephased ^{13}C MAS spectra (dephasing time: 400 μs) (green lines) of (a) **1au**$^+$-FABA$^-_{\rm P}$, (b) **1au**$^+$-FABA$^-_{\rm H}$, and (c) **1au**$^+$-FABA$^-$ as single crystals. ^{13}C NMR signals were enhanced by CP from protons. Dipolar-dephased ^{13}C MAS NMR measurements were used for the assignments of the signals for **1au**$^+$ and FABA$^-$. For example, in **1au**$^+$-FABA$^-_{\rm P}$, the broad signals at 132.2, 120.8, 93.4, and 89.3 ppm were assigned to **1au**$^+$, whereas the signals at 149.7, 137.7, and 126.0 ppm were assigned to FABA$^-$. **1au**$^+$-FABA$^-_{\rm H}$ showed slightly more broader signals than those of **1au**$^+$-FABA$^-_{\rm P}$, suggesting the formation of more disordered arrangement of constituting ions. On the other hand, ^{13}C CPMAS NMR for **1au**$^+$-FABA$^-$ as single crystals afforded narrower signals than those of **1au**$^+$-FABA$^-_{\rm P}$ and **1au**$^+$-FABA$^-_{\rm H}$, suggesting that the broader signals of **1au**$^+$-FABA$^-_{\rm P}$ and **1au**$^+$-FABA$^-_{\rm H}$ suggested the less-ordered arrangement of ions that form the Col$_{\rm h}$ structures. Furthermore, characteristic signal splitting for the *meso*-^{13}C signals at 93.5 and 88.0 ppm in (c) indicates the slipped stacking structure of **1au**$^+$ as supported by the theoretical simulation of ^{13}C NMR (Fig. S65). Although it is difficult to fully assign the signals, such signal splitting is also observed in (a), whereas splitting is almost disappeared in (b). These data with the support of the theoretical simulation suggest that **1au**$^+$-FABA$^-_{\rm P}$ forms an LeC state based on slipped stacking structures similar to **1au**$^+$-FABA$^-$ as observed in the crystal structure.

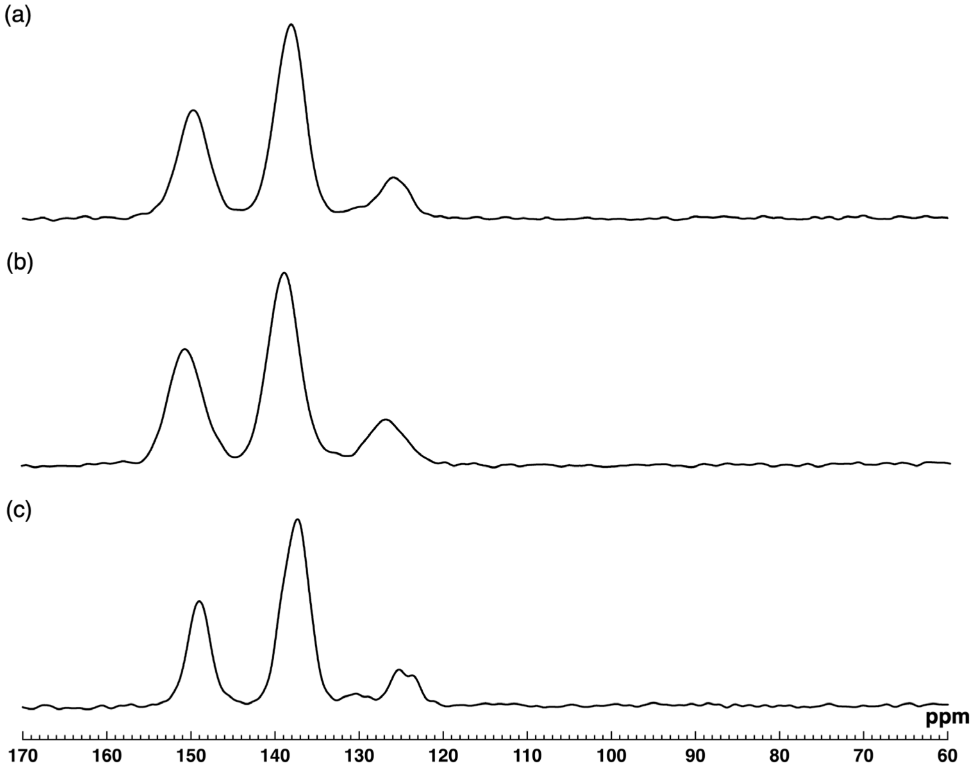

Fig. S87 ^{13}C CP-MAS NMR spectra of (a) **1au**$^{+}$-FABA$^{-}_{P}$, (b) **1au**$^{+}$-FABA$^{-}_{H}$, and (c) **1au**$^{+}$-FABA^{-} as single crystals. ^{13}C NMR signals were enhanced by the CP from ^{19}F.

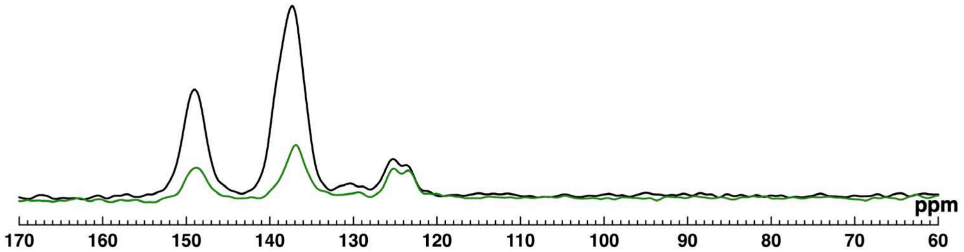

Fig. S88 Dipolar-dephased ^{13}C MAS spectra (green line) and ^{13}C CP-MAS NMR spectra (black line) as a reference of **1au**$^{+}$-FABA^{-} as single crystals. ^{13}C NMR signals were enhanced by the CP from ^{19}F.

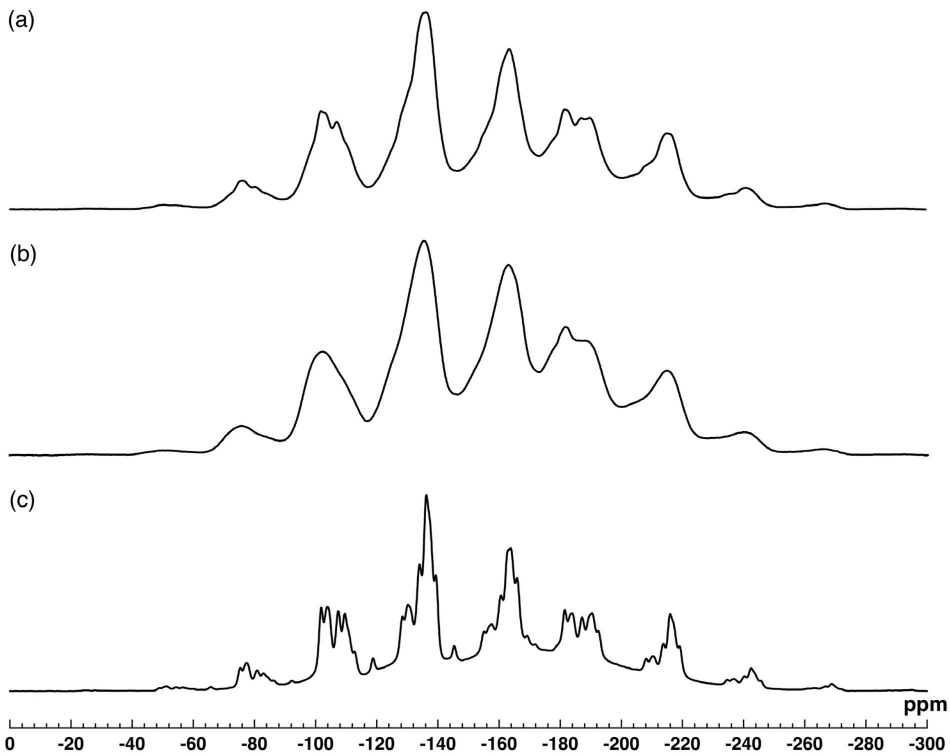

Fig. S89 ^{19}F MAS NMR spectra of (a) **1au**$^{+}$-FABA$^{-}_{P}$, (b) **1au**$^{+}$-FABA$^{-}_{H}$, and (c) **1au**$^{+}$-FABA^{-} as single crystals.

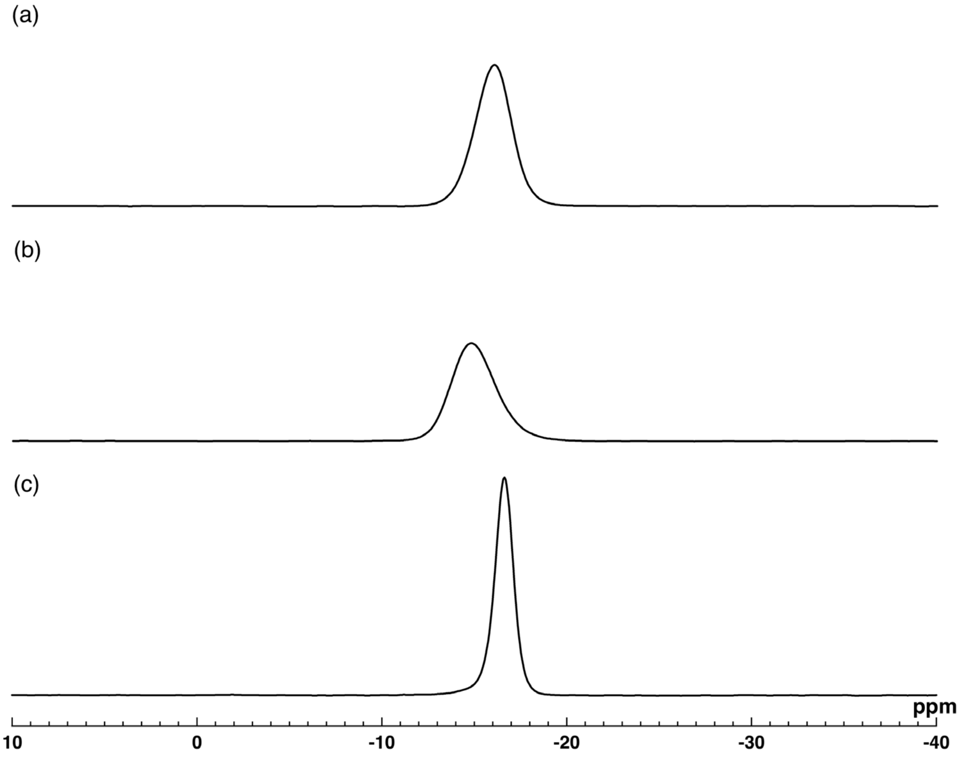

Fig. S90 ^{11}B MAS NMR spectra of (a) **1au**$^{+}$-FABA$^{-}_{P}$, (b) **1au**$^{+}$-FABA$^{-}_{H}$, and (c) **1au**$^{+}$-FABA^{-} as single crystals.

6. Electric conductivity properties

Method for Time-Resolved Microwave Conductivity and Electric Conductivity Measurements. All the single/poly-crystals of the compounds were placed onto quartz substrates (for quantitative analysis) and/or columnar quarts rods (8 mmϕ for anisotropic measurements) and were overcoated by Cytop®. The overcoated crystals were dried in vacuo for 30 min at 30 °C (Fig. S91,93) and at 60 °C (Fig. S92) prior to the measurements at 25 °C. Crystals on the quartz plate or rods were inserted into a TE-102 mode microwave cavity at Q-value of 2500 (quartz plates) or of 1300 (quartz rods) and were fixed at the position of electric field maximum. Excitation of the crystals was carried out through the quartz at 355 nm by third harmonic generation from a Spectra-Physics INDI Nd:YAG laser. The power of probing microwave was set at 3 mW. The excitation light intensity (I_0) through the crystal and quartz was monitored by an Ophir VEGA power meter with a PE-25 head. Microwave reflection signals (P_R and $\Delta P_R(t)$) from the cavity were evolved through a Schottky diode, amplified, and monitored by a Tektronics TDS3054 digital oscilloscope. Inside of the cavity was filled with dry N_2, and the measurements were performed at 25 °C. The evolved microwave reflection signal from the diode reflecting the power of microwave was converted into pseudo transient conductivity ($\phi\Sigma\mu$) as

$$\phi\sum\mu(t)=\frac{\Delta P_{\mathrm{R}}(t)}{eI_0AF_{\mathrm{L}}P_{\mathrm{R}}},$$

where e, A, and F_L are elementary charge, sensitivity factor, and filling factor, respectively. The latter two were estimated by numerical calculation from the overlap of excitation light absorption profile in the sample with transmittance of 0.28–0.41, and electric-field strength distribution in the cavity.

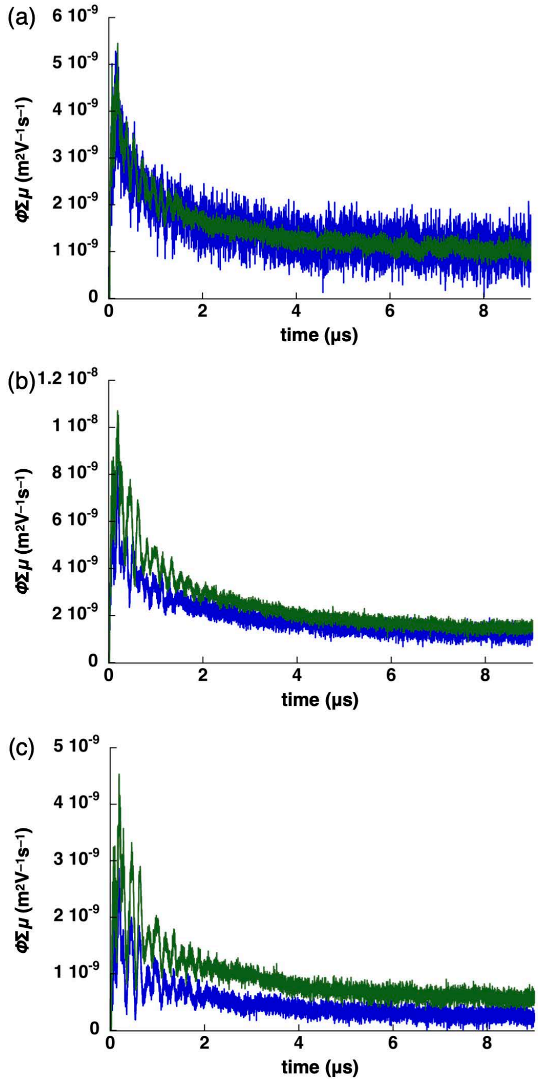

Fig. S91 FP-TRMC photoconductivity transients recorded for the single crystals of (a) **1au**$^+$-FABA$^-$, (b) **1au**$^+$-BArF$^-$, and (c) **1au**$^+$-PCCp$^-$, which were obtained by recrystallization from $CH_3CN/CHCl_3$, 1,1,1-trichloroethane/*n*-heptane, and DMF/*o*-dichlorobenzene, respectively (green and blue lines for long- and short-axis directions of the crystal, respectively), upon excitation at 355 nm, 9.1×10^{15} photons cm^{-2} pulse^{-1}, at r.t. Crystals of the compounds were fixed onto quartz substrate and were overcoated with Cytop® thin film, back-excited with the excitation light pulses. The electric conductivity ($\phi\Sigma\mu$) values for long-/short-axis directions of the crystals were $4.6 \times 10^{-9}/3.9 \times 10^{-9}$, $9.2 \times 10^{-9}/7.9 \times 10^{-9}$, and $2.9 \times 10^{-9}/2.9 \times 10^{-9}$ m^2 V^{-1} s^{-1} for **1au**$^+$-FABA$^-$, **1au**$^+$-BArF$^-$, and **1au**$^+$-PCCp$^-$, respectively. The order of the $\phi\Sigma\mu$ are consistent with the theoretically calculated transfer integrals (Fig. S63).

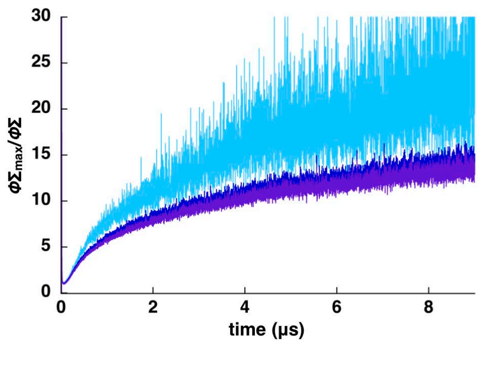

Fig. S92 Reciprocal conductivity transients recorded for the single crystals of **1au**$^+$-FABA$^-$ using microwave power of 2.1×10^{15}, 5.3×10^{15}, and 31×10^{15} photons cm^{-2} pulse^{-1} for light blue, blue, and purple lines, respectively, at r.t. under SF_6 atmosphere.

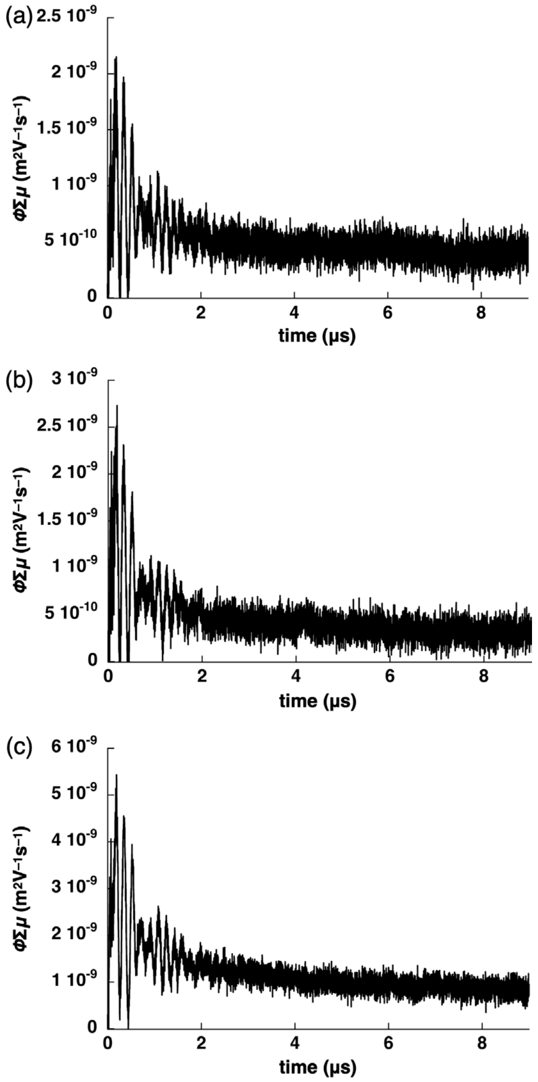

Fig. S93 FP-TRMC photoconductivity transients recorded for the LeC states of (a) **1au**$^+$-FABA$^-_{\mathrm{P}}$, (b) **1au**$^+$-FABA$^-_{\mathrm{H}}$, and (c) **1au**$^+$-BArF$^-_{\mathrm{P}}$, upon excitation at 355 nm, 9.1×10^{15} photons cm^{-2} pulse^{-1}, at r.t. Solid-state samples were fixed onto quartz substrate and were overcoated with Cytop® thin film, back-excited with the excitation light pulses. The electric conductivity ($\phi\Sigma\mu$) values were 1.2×10^{-9}, 2.0×10^{-9}, and 3.4×10^{-9} m^2 V^{-1} s^{-1} for **1au**$^+$-FABA$^-_{\mathrm{P}}$, **1au**$^+$-FABA$^-_{\mathrm{H}}$, and **1au**$^+$-BArF$^-_{\mathrm{P}}$, respectively.
